# User-Centered Counseling and Male Involvement in Contraceptive Decision Making: Protocol for a Randomized Controlled Trial

**DOI:** 10.2196/24884

**Published:** 2021-04-05

**Authors:** Mahesh Karra, Kexin Zhang

**Affiliations:** 1 Frederick S Pardee School of Global Studies Boston University Boston, MA United States; 2 Department of Economics Boston University Boston, MA United States

**Keywords:** user-centered counseling, male involvement, contraceptive use, family planning, randomized controlled trial, Malawi, Sub-Saharan Africa

## Abstract

**Background:**

To achieve informed choice within the framework of reproductive autonomy, family planning programs have begun to adopt user-centered approaches to service provision, which highlight the individual client as the focal point of interaction and key decision maker. However, little is known about how user-centered approaches to family planning, particularly family planning counseling, shape contraceptive preferences and choices.

**Objective:**

We conducted a multiarmed randomized controlled trial to identify the causal impact of user-centered approaches to family planning counseling on women’s contraceptive decision making in urban Malawi. This study aims to determine how a tailored, preference-driven approach to family planning counseling and the involvement of male partners during the counseling process may contribute to shaping women’s contraceptive preferences and choices.

**Methods:**

Married women aged 18-35 years were recruited and randomly assigned to 1 of the 3 intervention arms or a control arm characterized by the following two interventions: an intervention arm in which women were encouraged to invite their husbands to family planning counseling (husband invitation arm) and an intervention arm in which women received targeted, tailored counseling on up to five contraceptive methods (as opposed to up to 13 contraceptive methods) that reflected women’s stated preferences for contraceptive methods. Women were randomized into a control arm, T0 (no husband invitation, standard counseling); T1 (husband invitation, standard counseling); T2 (no husband invitation, targeted counseling); and T3 (husband invitation, targeted counseling). Following counseling, all women received a package of family planning services, which included free transportation to a local family planning clinic and financial reimbursement for family planning services. Follow-up surveys were conducted with women 1 month after counseling.

**Results:**

A total of 785 women completed the baseline survey, and 782 eligible respondents were randomized to 1 of the 3 intervention groups or the control group (T1, n=223; T2, n=225; T3, n=228; T0, n=108). Furthermore, 98.1% (767/782) of women were contacted for follow-up. Among the 767 women who were contacted, 95.3% (731/767) completed the follow-up survey. The analysis of the primary outcomes is ongoing and is expected to be completed by the end of 2021.

**Conclusions:**

The results from this trial will fill knowledge gaps on the effectiveness of tailored family planning counseling and male involvement in family planning on women’s stated and realized contraceptive preferences. More generally, the study will provide evidence on how user-centered counseling may affect women’s willingness to use and continue contraception to realize their contraceptive preferences.

**Trial Registration:**

American Economics Association’s Registry for Randomized Controlled Trials AEARCTR-0004194; https://www.socialscienceregistry.org/trials/4194/history/46808. Registry for International Development Impact Evaluations RIDIE-STUDY-ID-5ce4f42bbc2bf; https://ridie.3ieimpact.org/index.php?r=search/detailView&id=823.

**International Registered Report Identifier (IRRID):**

DERR1-10.2196/24884

## Introduction

### Choice Architecture in Family Planning

Unlike other domains in women’s health, delivering high-quality family planning services is reflected by the achievement of good reproductive health outcomes and the objective of helping women maximize a complex set of preferences around future fertility outcomes. In family planning, the role of the client as the key actor in her receipt of care is distinct from most other contexts in health-related decision making, wherein providers often play a leading role in determining which course of treatment is best for a patient. Indeed, family planning programs typically consider women to have the right to full information about contraceptive method options. For this reason, family planning programs dedicate significant resources to providing complete and accurate information so that women are informed of the full range of contraceptive methods. Clients typically do not receive modern contraceptive methods without receiving a comprehensive consultation session with a family planning counselor (typically a health care professional or social worker), during which time they are informed about the range of available methods. Due to the high value placed on fully informed choice, counselors may discuss as many as 13 different methods and describe as many as 10 method attributes (eg, effectiveness at preventing pregnancy, the ease of use, the risk of side effects, and the duration of effectiveness, among others) for each method with a client. This information-intensive approach to counseling, which compels a client to interpret a large volume of information across several dimensions (attributes and methods), may reduce the salience of counseling while simultaneously increasing the potential for choice overload.

Several studies have examined how family planning counseling affects women’s contraceptive uptake [1]. However, little is known about how the choice architecture for family planning—the structures and processes by which contraceptive methods and information are presented to women—actually shapes women’s preferences. Studies have shown that a woman’s fertility intentions, which affect her contraceptive preferences, are likely to be unstable over her reproductive lifetime [2-4] and are sensitive to relatively small changes in her environment. A number of studies have examined the effect of choice architecture on people’s decision-making processes [5-7] and demonstrated that increased cognitive load leads to more risk-averse behaviors, higher levels of impatience, and a higher likelihood of deferring decision making. In addition, studies have revealed that choice architecture–based approaches can be used to nudge an individual to make better choices without forcing certain outcomes [8-12]. For example, when faced with a small number of well-understood alternatives, a person tends to examine all the attributes of all the alternatives and then make trade-offs when necessary. However, when the choice set becomes large, alternative strategies (eg, structuring complex choices into a certain order) are employed [13]. These studies suggest that the complexity of options may adversely affect an individual’s choice. In the context of family planning, Delavande [14] showed that the extent to which a contraceptive method is effective, the extent to which such a method protects against sexually transmitted infections (STIs), and the extent to which the method is approved (or disapproved) by a woman’s partner are the three most important method-related attributes that drive a woman’s choice of contraception. As a woman makes her contraceptive choice based on the attributes of a certain method, information about methods that are tailored to a woman’s most valued attributes may, in turn, allow her to reinforce and better realize her preferences for a contraceptive method.

### Male Involvement in Family Planning Counseling

In addition to the role of choice architecture, the involvement of men in family planning and reproductive health decision making, specifically partner involvement in contraceptive counseling, also plays a crucial role in shaping women’s preferences. In most family planning counseling programs however, male partners are often not required to be counseled. Often, male partners do not participate in the counseling process unless they are actively brought by women themselves. A wide range of studies have shown that including men in family planning counseling might increase the use of contraceptive methods through two channels [15-20]. First, counseling may provide men with more detailed information on the benefits of family planning and contraception [15,16]. Second, couples counseling provides a platform for increased spousal communication and offers couples the opportunity to discuss fertility and method preferences more openly. This result is attested in a series of cross-sectional studies that found a positive link between spousal communication and contraceptive use [21-25]. A small number of impact evaluations of male involvement in counseling have been conducted in a handful of countries, including China [26], Ethiopia [27], Malawi [15], Zambia [28], Tanzania [21], and Jordan [29]. To date however, experimental evidence on spousal concordance and the role of men in family planning decision making remains limited and mixed, particularly in low- and middle-income settings. Moreover, existing family planning programs cannot compel women to invite their husbands or male partners to be counseled; therefore, couples who were enrolled for counseling were, by construction, selected because they were willing to be counseled together. These couples and, in particular, the male partners who were selected and participated in counseling are not likely to be representative of typical couples and men. To test the impact of male involvement more effectively, it would be necessary to examine the extent to which giving women the choice to invite or involve their husbands impacts male involvement and subsequent outcomes.

### User-Centered Approaches to Family Planning

To achieve full, free, and informed choices within the framework of reproductive autonomy and rights, programs have increasingly begun to adopt user-centered approaches to family planning counseling and service provision. These approaches have stressed the role of the individual client as the focal point of interaction and the key decision maker. Recently, user-centered counseling approaches to family planning, such as the Population Council’s Balanced Counseling Strategy (BCS) [30,31], IDEO’s human-centered design methods [32,33], and others, have been developed to allow counselors to elicit women’s contraceptive preferences along various dimensions more effectively. In developed countries, user-centered counseling strategies on family planning have also been implemented. A decision support tool named *My Birth Control*—which has educational and interactive modules and produces a provider printout with the patient’s preferences—has been proven to improve patient centeredness of contraceptive counseling [34]. These strategies were designed to allow for the identification of a more tailored range of contraceptive methods that better match women’s preferences. Such counseling approaches, although promising, have some limitations. For instance, many of them are found to be time-consuming and difficult to scale up to larger client bases. A recent report on the BCS shows that the BCS toolkit includes (1) an algorithm that summarizes 11 steps needed to implement the strategy; (2) counseling cards with basic information about 15 family planning methods, plus a card with the checklist to ensure that a woman is not pregnant; and (3) brochures on each of the methods for clients. In addition, there is little evidence on the effectiveness of these approaches in meeting women’s fertility desires [35].

### Family Planning Counseling in Malawi

Counseling with a service provider is often the first step for women to learn about, choose, and receive family planning services in Malawi. As stated in the National Reproductive Health Service Delivery Guidelines 2014-2019, counseling is intended to be an interactive process in which the provider seeks to identify the client’s needs, elicit the client’s concerns, and offer relevant information and guidance to enable the client to make an informed decision about methods. During the counseling session, women often receive a range of information, such as the cost of procuring contraceptives (although many methods are provided for free in public facilities), side effects or contraindications associated with methods, and method effectiveness [36]. In public health facilities in Malawi, women typically receive a group counseling session with a nurse or family planning counselor, followed by a short (an estimated 3- to 5-min) individual counseling session, at which time they may choose to receive a contraceptive method. According to the guidelines set by the Ministry of Health (MOH) and the Malawi Reproductive Health Directorate (RHD), a family planning counseling session is typically administered to women with a family planning flip chart, which describes 13 contraceptive methods that are organized in order of method effectiveness to preventing pregnancy, starting with female and male sterilization, and concluding with traditional methods of contraception (Multimedia Appendix 1). Although the flip chart is comprehensive in the information provided to each woman about each method, this counseling procedure does not prioritize women’s preferences for either method attributes or methods themselves. Moreover, counseling in this manner may likely anchor women’s preferences to methods based on a default top attribute (method effectiveness), which may not be the most preferred. Given the limited time for individual counseling, there is also little opportunity for women to receive clarification or follow-up that they may seek, and service providers may not be able to fully elicit a woman’s family planning and fertility preferences before providing them with the most suitable services. Finally, most counseling sessions, particularly those involving group counseling, are exclusively targeted to women clients, with few opportunities for men to participate in the service provision and decision-making processes for family planning.

### Study Objectives

In this study, we identified the causal impact of user-centered approaches to family planning counseling on women’s contraceptive preferences and decision making by means of a randomized controlled trial. In particular, we investigated two channels that have been hypothesized to play a role in contraceptive decision making: (1) a preference-based, targeted, user-centered approach to family planning counseling and (2) male involvement in family planning counseling. To this end, we tested the following 2 hypotheses:

1. A preference-driven, tailored, user-centered approach to counseling would allow women to express and realize their contraceptive preferences more effectively.
2. The involvement of male partners in family planning counseling may allow women to express their contraceptive preferences and, in turn, translate their preferences into behavior more effectively.

The study population comprised married women aged 18-35 years living in Lilongwe, Malawi. As part of the trial, each woman in the study was randomly assigned to 1 of the 3 treatment arms or a control arm. A woman assigned to one of the intervention arms received one tailored, user-centered counseling session, followed by a package of family planning services that was designed to reduce the key barriers to accessing family planning in urban Malawi. The primary objective of this study is to evaluate the impact of user-centered counseling on a woman’s contraceptive use, intention to change stated ideal methods, switching of the current method, and realization of the stated ideal method. Primary outcomes include changes to women’s contraceptive method preferences (both stated and realized) over time and changes to women’s contraceptive concordance (whether a woman’s stated choice of contraceptive method, her ideal method, is the method that she is, in fact, using). Secondary outcomes include changes to male partners’ fertility and family planning preferences, women’s sexual and marital well-being, and changes in women’s decision-making in the household.

This study seeks to fill the current knowledge gaps in the evidence on the causal impact of user-centered counseling on women’s contraceptive decision making and behavior. Specifically, the study documents how women’s preferences for family planning may change over time and investigates the extent to which women’s stated contraceptive preferences are realized and translated into behavior. A downstream objective of this study is to investigate the extent to which improved counseling and outreach may contribute to women’s empowerment and autonomy, more generally.

## Methods

### Study Approval

Human subject approvals were received from the Boston University (BU) Institutional Review Board (IRB; IRB Protocol Number: IRB5162E), the Malawi National Health Sciences Research Committee (NHSRC; NHSRC Approval Number: 2350), the Lilongwe District Council, the Malawi Police Service, and the Malawi MOH to conduct the study. A Memorandum of Understanding was established with the Good Health Kauma Clinic.

### Study Setting

Our study was conducted in urban areas of Lilongwe, the capital of Malawi. Estimates from the 2015-2016 Malawi Demographics and Health Survey (MDHS) demonstrate that the contraceptive prevalence rate in Malawi was 45.2% among all women of reproductive age (aged 15-49 years) and 59.2% among married women of reproductive age [37]. These estimated contraceptive prevalence rates exhibit a significant increase from the 2010 MDHS; nevertheless, the unmet need for family planning has remained high, with an estimated 18.7% of women in Malawi reporting to have an unmet need for spacing or limiting births. Injectable contraceptives were the most popular method in Malawi in 2010 and were used by 22.5% of women, followed by intrauterine devices and female sterilization at 9% and 8.3%, respectively [38]. The distribution of contraceptive users by method, or contraceptive method mix, has not changed significantly over time among married women in Malawi, as injectable contraceptives, intrauterine devices, and female sterilization remain the most popular methods used by 30%, 11.5%, and 10.9%, respectively. In Malawi, nearly 8 in 10 (79%) modern contraceptive users aged 19-45 years procure their method from public sector providers (government hospitals or clinics), whereas 8% of users procure their method from nongovernmental providers such as Banja la Mtsogolo (BLM), 6% of users procure their method from the private medical sector, and 4% of users procure their method from the Christian Health Association of Malawi and other faith-based service providers [37].

Although the contraceptive prevalence rate in Malawi has continued to rise over the past decade, the contraception discontinuation rate in Malawi has also remained high, with more than 37% of women reporting that they discontinued their family planning method within the last year, among which half of them discontinued because of nonfertility-related reasons (eg, method-related reasons, such as side effects). This high rate of contraceptive discontinuation suggests that barriers may exist in a woman’s decision-making process that prevent her from choosing the *right* method that caters to her specific preferences. Although an increasing number of family planning programs have been successful in increasing the contraceptive uptake, it is crucial to be reminded that a woman’s family planning preferences are not realized simply from an increased use of contraceptive methods, which has been reiterated in numerous settings by reproductive rights researchers, policy makers, and advocates alike [39]. Family planning programs have mainly focused on the extensive margin of family planning utilization (ie, increasing contraceptive uptake); however, few existing studies have focused on the intensive margin to determine whether the increase in utilization implies that women’s preferences, specifically her choice of contraceptive method, are, in fact, being met.

### Study Sample and Inclusion Criteria

**Figure 1:**
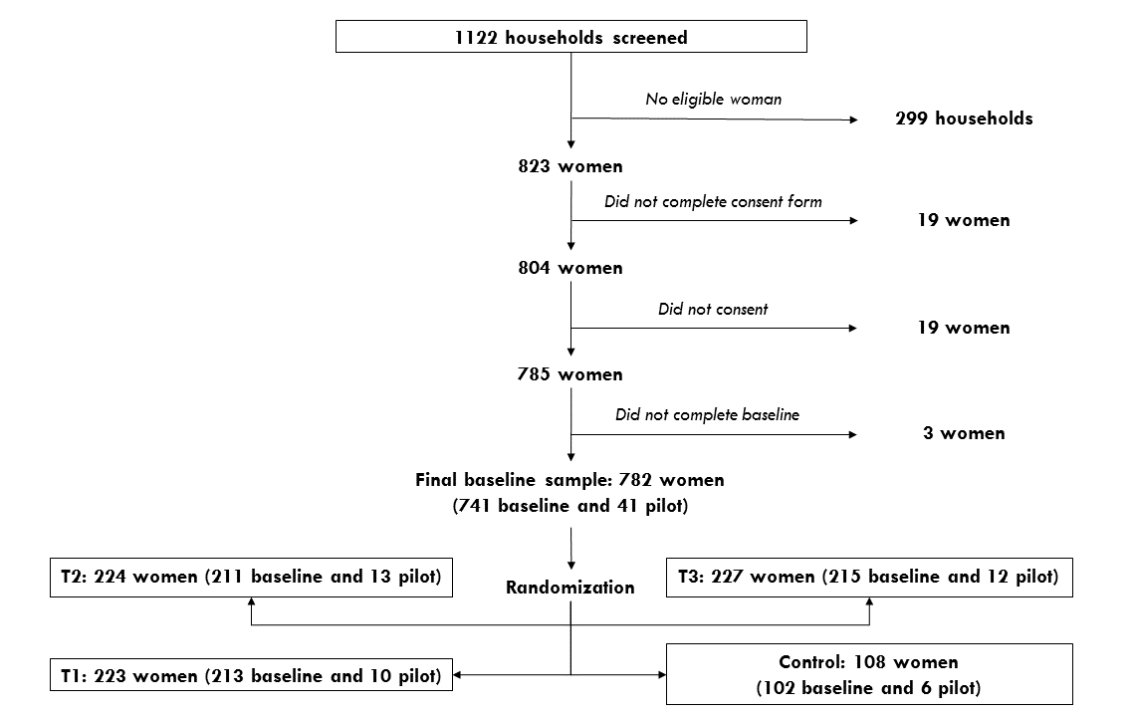
Experimental framework and flowchart.

This study is a multiarmed randomized controlled trial that was conducted with a sample of women of reproductive age from Lilongwe, Malawi. The study comprised a baseline survey, randomization into 1 of 3 treatment arms or 1 control arm, and the implementation of a 2-month intervention. One follow-up survey was conducted after the intervention and was administered to women either at the clinic or on the phone. If participants could not be reached in either way, then a home visit was paid to the households. Figure 1 outlines the general framework of the entire field experiment.

For this study, we recruited 782 women who, at the time of the baseline survey, (1) were married, (2) were aged between 18 and 35 years, (3) lived in the city of Lilongwe (permanent residents), (4) were not pregnant and did not gave birth in the 6 months before the initial screening, (5) had neither been sterilized nor had a hysterectomy, (6) had given birth to at least one child (one live birth) in their lifetime, and (7) lived with their husbands at the time of the screening. These criteria were designed to identify women who were most likely to seek and use a contraceptive method if given the opportunity and means. Women who successfully met these seven inclusion criteria and who consented to participate in the main part of our study were recruited. In addition, no two eligible women were enrolled from the same household. If multiple women from the same household were potentially eligible to be recruited, then we chose the youngest eligible woman from the household to participate. Given that randomization was to be administered at the individual woman level, it was necessary to select only one eligible woman from a household to minimize any possible contamination across the intervention and control arms. We also ensured that eligible women who were selected for the study were sufficiently distant (at least five households apart) from each other, which also served to reduce any spillover effects between treated and control women who lived in the same neighborhood.

In addition, we interviewed the husbands of women who were invited to the counseling session by their wives. The men who were selected to be interviewed were husbands who (1) were assigned to 1 of the 2 intervention arms that encouraged women to invite them and (2) consented to participate in the counseling session with their wives.

### Recruitment and Study Timeline

Our study recruitment design closely follows the recruitment protocols of Karra and Canning [40]. Using the most recent Demographics and Health Survey (DHS) and census maps of Lilongwe’s enumeration areas and listings of households and neighborhoods, we employed a 2-stage sample selection procedure that is based on the sampling strategy used by the DHS. In the first stage, we randomly selected defined census areas in Lilongwe to be screened until we have selected enough enumeration areas to contain at least 5000 households in total. In the second stage, our enumerators proceeded door-to-door to screen households in each selected enumeration area for potentially eligible women. Enumerators screened each household that they approached to determine if any woman who was living in that household (1) met our inclusion criteria listed in the section above and (2) consented to participate in our study. To make this determination, enumerators used a recruitment script to verify eligibility and presented the eligible woman with a consent form to participate in the study. Multimedia Appendix 2 presents the recruitment script and the consent form. Written (or verbal) informed consent was obtained from all participating women before proceeding to administering the survey. On the basis of our knowledge of participation refusal rates and the estimated proportion of eligible women in Lilongwe, we estimated the need to screen at least 940 households across our randomly selected enumeration areas to attain a desired sample size of at least 700 women. We also needed to choose enough enumeration areas to have at least 5000 households in total (940×5=4700 households among the women who made up our sample and who were at least five households apart, plus an additional 300 households that were screened, but where women either did not meet the eligibility criteria or refused to participate). Recruitment from the selected enumeration areas ceased after at least 700 women were found who met the eligibility conditions, consented to participate in the study, and were administered the baseline survey.

This study spanned a total of 14 months from January 2019 to February 2020. Table 1 presents the study timeline. Before recruiting and conducting the study with the main study sample, a small sample of women was recruited to pilot new survey instruments and intervention activities over the study period. All research activities, including recruitment, consent, study instrument administration, and intervention administration, were administered to the pilot sample using the same study protocols as those used for the main sample. For this reason, the final analytic data sets for the study comprised data from both the pilot and main samples.

**Table 1:** Study timeline.

Activity	Year						
	2019					2020	
	Months 1-5	Months 5-6	Months 6-7	Months 7-9	Months 9-12	Months 12-13	Months 13-14
Preparation of survey instruments, consent forms, recruitment materials, and Boston University Institutional Review Board protocols	✓^a^	—^b^	—	—	—	—	—
Hiring of study staff and interventionists, obtaining local institutional review board approval in Malawi, and identifying sampling strategy	—	✓	—	—	—	—	—
Training of local staff, lay interventionists, and enumerators	—	—	✓	—	—	—	—
Baseline survey administration, randomization, and intervention administration	—	—	—	✓	—	—	—
Intervention period—counseling	—	—	—	—	✓	—	—
Follow-up surveys at the clinic through phone calls and through visits	—	—	—	—	—	✓	—
Final reports, publications, and dissemination	—	—	—	—	—	—	✓

^a^The specified study activity was conducted in the time period.

^b^Not available. The specified study activity was not conducted in the time period.

### Informed Consent and Participant Privacy

Our consent and privacy processes closely follow the consent protocols established by Karra and Canning [40]. The process of obtaining consent was consistent for all potential participants: women and eligible husbands. Written consent to participate was obtained from all the participants before administering the surveys. Once participants agreed to join the study, a copy of the consent form script was read to them, and they were given opportunities to ask questions and express concerns. Surveyors checked for comprehension throughout the consent process, which took 5-10 minutes on average. Multimedia Appendix 1 presents the consent documents and details of the consent process. After completing the consent script, potential participants were asked if they would like to participate. If the participant wished to take further time to reflect, then the surveyor and the participant would determine the time and method of reconnecting. If the potential participant agreed to participate in the study, then consent was obtained and documented by obtaining the signatures of both the participant and the study staff member who provided consent. Upon providing consent, the surveyor would then conduct the survey.

For follow-up surveys, written consent to participate was obtained from women who were followed up either at home or at the clinic, and verbal consent to participate was obtained over the phone from participants who were originally recruited at baseline but who did not visit the clinic over the intervention period. Respondents who had previously indicated that they were no longer interested in participating in the study were not contacted. All survey responses were collected on Android (Google LLC)-based tablets, and data were securely transferred to the CommCare-supported server daily.

Throughout the consent process, each participant was informed that even if she decides to participate and sign the consent form, she could decide at any time to end her participation. If the prospective study participant was not literate, then a witness who did not work for the study signed the consent form. In the absence of witnesses, the participant could confirm their consent by placing a thumbprint on their consent form, and a photo of the thumbprint was taken as a record of consent. Moreover, participants were encouraged to contact the researchers with further questions at any time during the study. Consent was obtained before any surveys were conducted, and all participants were informed that their participation was completely voluntary.

All recruitment scripts, consent forms, and survey instruments were translated by a certified translator from English into Chichewa and were back-translated into English by a second certified translator to ensure accuracy.

When presented with the intervention, women were offered each intervention component (counseling and family planning package) by an interventionist from the study team. Women were informed that they could take up and stop any or all components of the intervention at any time. Counselors asked for a woman’s consent to participate before each counseling session.

All surveys and administration of the intervention were conducted in a private room. To maintain a respondent’s privacy during an attempt to reach her by phone, the field enumerator conducting the call left no indication of the reason for the phone attempt (eg, voicemail and text message) on the respondent’s phone should there be no response to a call. To ensure that the respondent’s participation remains private, the field enumerator only continued with the phone call once she received assurance from the woman that she was able to speak on the phone without being overheard or interrupted. Any disruption or interruption during the phone call resulted in postponement or termination of the call.

### Randomization

Following the baseline survey, women who consented to participate were randomized into 1 of the following 4 experimental arms:

1. A control group (T0), in which a woman received a private counseling session on the full range of 13 contraceptive methods following a standard counseling process (n=100).
2. An intervention group (T1), in which a woman was encouraged (but not compelled) to invite her husband to a joint counseling session. A woman (and her husband if she chose to invite him) then received a private counseling session on a full range of 13 contraceptive methods (n=200).
3. An intervention group (T2), in which a woman received a private counseling session on 5 targeted contraceptive methods based on her baseline preferences for family planning (n=200).
4. An intervention group (T3), in which a woman was encouraged to invite her husband to a joint counseling session. A woman (and her husband if she chose to invite him) was then counseled on 5 targeted contraceptive methods based on her baseline preferences for family planning (n=200).

Upon the completion of baseline data collection, women were randomized to 1 of the 3 intervention arms or the control arm, such that the intervention assignment was balanced along the following baseline characteristics: the use of family planning, most preferred method attributes, and age. As part of the balancing process, strata by each combination of characteristic values were created, and observations were assigned to their respective strata. Observations within each stratum were then individually randomized to each of the 4 groups by the principal investigator (PI) in a 2:2:2:1 allocation using computer-generated randomization using STATA, version 14 (StataCorp LLC). Following individual randomization, the Innovations for Poverty Action (IPA) Malawi intervention team implemented the intervention in the participants randomized into the intervention arms. Although the implementation team was not involved in the actual randomization process, counselors, who were responsible for administering the correct counseling and follow-up procedures to each respondent, were not blinded to respondent assignments to each of the intervention arms.

### The Intervention

#### Intervention Protocols

All women who consented to participate in the study (T0-T3) were offered a free, private family planning counseling session, which included a risk assessment for contraceptive methods and detailed information on methods switching, side effects associated with each method, and the benefits of contraception and birth spacing. A precounseling survey was administered to each woman, where they were asked to confirm their personal information, including, but not limited to, pregnancy status, current contraceptive use, and preferences for fertility and contraception. During counseling sessions, counselors provided women with information on the range of modern and natural family planning methods based on their respective intervention assignments. Strategies on how to communicate family planning messages with partners and on how to increase partner awareness were conveyed during the sessions. The counseling session lasted no more than 1 hour and was administered in a private room by a counselor who was trained to provide family planning and reproductive health services. To facilitate an effective and open dialog with women during the counseling session, only female counselors were hired. Counselors were hired by IPA Malawi, held a nursing degree, and were trained on the range of counseling protocols over a week. We enlisted the support of the Malawi RHD and several international nongovernmental organizations who work on family planning, including Population Services International (PSI), BLM, the Family Planning Association of Malawi, and FHI360, to help us develop training materials and counseling resources. We also collaborated with the Malawi RHD, BLM, and PSI to assist with counselor training.

#### Counseling for Control Group T0

Following the introductory risk assessment and discussion on the benefits of contraception and birth spacing, women who were assigned to the control arm were counseled on the full range of 13 available family planning methods. Counselors employed the standard-of-care contraceptive method flip chart that is provided by the MOH and RHD and counseled women on each method following the order of methods in the flip chart.

#### Counseling for Intervention Group T1

Before receiving the counseling session, women who were assigned to the intervention arm T1 were encouraged by the counselor to invite their husbands or male partners to participate in a joint family planning counseling session. Following the invitation, women and their husbands (if they chose to invite them) jointly received an introductory risk assessment and discussion on the benefits of contraception and birth spacing. Women and their husbands were then jointly counseled on the full range of 13 available family planning methods using the standard counseling flip chart, as described for intervention arm T0.

#### Counseling for Intervention Group T2

Following the introductory risk assessment and discussion on the benefits of contraception and birth spacing, women who were assigned to the intervention arm T2 were counseled on a targeted number of methods that were chosen based on the respondents’ reported contraceptive preferences at baseline. The objective of this intervention arm was to minimize the choice overload and increase the salience of a woman’s most preferred method attribute (eg, method effectiveness in preventing pregnancy, duration of use, and likelihood of method-related side effects). At baseline, each woman was asked to assign a relative rank to her top 3 most valued method-specific attributes (eg, does she prefer that a method has a lower incidence of side effects over a method that is more effective at preventing pregnancy?). On the basis of her ranking of method attributes, the counselor confirmed the attribute that the woman revealed to be most important (eg, methods with low incidence of side effects) and used a predesigned tailored flip chart (an abbreviated version of the full flip chart) to present a subset of 5 methods that ranked highest along that revealed attribute. Particular emphasis was placed on making the order of presentation salient, in which women were reminded and primed to consider the relative ranking of methods along the stated attribute. Counselors then counseled women on each of the 5 methods following the standard family planning counseling procedure described above. Additional details on each of the predesigned tailored flip charts are presented in Table 2.

**Table 2:** Attribute-method flip chart correspondence.

Flip chart color and methods		Attributes
**Blue**		Effective at preventing pregnancy Duration of effect or lasts long
	Sterilization	
	IUD^a^	
	Implants	
	Injectables	
	Pill	
**Purple**		
	LAM^b^	No risk of harming health
	Two-day method	No effect on monthly bleeding
	Rhythm method	No unpleasant side effects
	Standard days method	Low cost
	Condoms	No risk of infertility
	N/A^c^	Nonhormonal
	N/A	No need to go to the clinic to procure the method
**Pink**		Immediate return to fertility
	Condoms	
	Two-day method	
	Rhythm method	
	Standard days method	
	IUD	
**Yellow**		
	Condoms	Protects against HIV or STId
**Gray**		
	IUD	Want to try something new or tired of old method
	Implants	My doctor recommended it to me
	Sterilization	My husband wanted me to use this method
	Pills	Other women in my family have used this method
	Injectables	Friends have used this method
	N/A	Easily available at clinic
**Orange**		No need to remember to use
	Sterilization	
	IUD	
	Implants	
	Injectables	
**White**		No need to go to the clinic to resupply
	Two-day method	
	Rhythm method	
	Standard days method	
	Sterilization	
	IUD	
	Implants	
**Red**		Concealable
	LAM	
	Two-day method	
	Rhythm method	
	Standard days method	
	Injectables	
**Black**		Does not interfere with sex
	Sterilization	
	IUD	
	LAM	
	Implants	
	Injectables	
	Pills	
**Green**		Control group only
	IUD	
	Implants	
	Sterilization	
	Pills	
	Injectables	
	Condoms	
	LAM	
	Two-day method	
	Rhythm	
	Standard days method	

^a^IUD: intrauterine device.

^b^LAM: lactational amenorrhea method.

^c^N/A: not applicable.

^d^STI: sexually transmitted infection.

#### Counseling for Intervention Group T3

Before receiving the counseling session, women who were assigned to the intervention arm T3 were encouraged by the counselor to invite their husbands or male partners to participate in a joint family planning counseling session. Following the invitation, women and their husbands (if they chose to invite them) received an introductory risk assessment and discussion on the benefits of contraception and birth spacing. In following the counseling protocols of the T2 intervention arm above, a woman and her husband were jointly counseled on a targeted range of 5 family planning methods based on the woman’s highest ranked preferred method attribute she identified at baseline. Before counseling, counselors confirmed the woman’s highest ranked attribute and jointly counseled the woman and her husband on the targeted subset of 5 contraceptive methods that most closely aligned with her most preferred attribute using a tailored, condensed flip chart specified in Table 2.

### Postcounseling Survey

#### Survey Instrument and Rollout

Following the counseling session, counselors conducted a brief survey with all women to assess their experiences with the counseling service. Multimedia Appendix 2 presents the postcounseling survey instrument. A particular aim of this follow-up survey was to elicit women’s preferred choice of contraceptive method and the cited reasons for choosing this method immediately after counseling. Interviews with women and husbands who were present took no more than 15 minutes and were conducted privately in accordance with the standard interviewing procedures that were established for the baseline survey.

#### Postcounseling Package of Services

Following the postcounseling survey, all women (and husbands who participated in the counseling session) were offered the following package of services for a 1-month period:

1. Transportation service: women (and applicable husbands) were offered a free transportation service from their homes to the Good Health Kauma Clinic for a period of 1 month. The transportation service was provided by a driver who was hired and trained by IPA Malawi. Respondents received the phone number of a female field manager and were instructed to let the field manager contact the driver to transport them to the Good Health Kauma Clinic during the clinic’s normal working hours, that is, from 8 AM to 5 PM from Monday to Saturday. The field manager maintained a daily schedule of respondents who requested the driver’s services, and the respondents were instructed to notify the field manager at least one day before they wish to go to the clinic to ensure that the driver would be able to transport them. The field manager also provided 1 day’s advanced notice to the Good Health Kauma Clinic to inform them of the number of clients who could be expected to attend the clinic on the following day. Each woman who visited the clinic was always accompanied by the female field manager; the presence of another woman in the vehicle acted to minimize potential stigma associated with a woman traveling alone in the company of a man and also provided comfort to the participant.
2. Financial reimbursement for family planning services: finally, women and participating husbands were financially reimbursed for any out-of-pocket expenditures that they incurred for receiving family planning care at the Good Health Kauma Clinic for the 1-month service period. Costs that were eligible for reimbursement included the procurement costs of family planning medications and contraceptive methods, family planning consultation fees, lab test fees, treatment costs for any contraceptive-related side effects and contraindications, expenses associated with switching and discontinuation of methods, and exam fees. Each couple was allowed a maximum reimbursement amount of MWK 17,500 (US $25.00), which could be redeemed by the couple over multiple visits at the Good Health Kauma Clinic. The extent to which a given expenditure at the clinic met the criteria for being reimbursed was determined by the clinician. For every family planning service that was eligible for reimbursement, the cost of the service was deducted from her reimbursement allowance.

In addition, women and participating husbands who experienced any side effects because of contraceptive use over the course of the service period were entitled to free treatment. If a woman or her husband experienced a side effect or contraindication, then she or he may seek care at her nearest public clinic, public hospital, or the Good Health Kauma Clinic. The woman was asked to keep receipts of any costs incurred at the health facility so that she could be reimbursed later. The reimbursement covered the cost of side effects treatment for all family planning methods used by the woman, regardless of where the method or treatment was procured. All reimbursements for incurred costs were distributed as closely as possible to the time that the reimbursable cost was incurred, most likely within 2-3 business days by the field manager.

At the end of the counseling session, the family planning counselor presented a terms of service document, which specified the terms and conditions for each of the abovementioned services to the woman. Multimedia Appendix 2 presents the terms of service documents for each of the 4 interventions. Counselors answered any questions from the woman about the package of services and then asked for the woman’s (and her husband’s) understanding of these terms. Each respondent also received a paper copy of the terms of the service document.

### Follow-up

#### Follow-Up Phased Rollout

Following the 1-month service period, the entire study sample of women was resurveyed with an abbreviated version of the baseline survey questionnaire. Resurveying participants in this manner enables us to create a panel of individual women where each woman is observed at 3 time points. The same protocols regarding data security and confidentiality that were enforced at baseline were implemented once again for each follow-up survey round.

Follow-up surveys were administered in 3 phases: (1) a clinic-based survey that was administered to women (and participating husbands) who visited the Good Health Kauma Clinic, (2) a phone follow-up survey that was administered to women who did not visit the Good Health Kauma Clinic, and (3) an in-person home-based follow-up survey that was administered to women who neither visited the Good Health Kauma Clinic nor were available for a phone follow-up survey.

#### Clinic-Based Survey

The 1-month clinic-based follow-up survey was administered by the local field team and in person to all women (and husbands) who visited the Good Health Kauma Clinic over the 1-month service period. The clinic-based survey, which is an abbreviated version of the main baseline survey instrument, took no more than 20 minutes and was administered by the female field manager who accompanied the woman to the clinic.

#### Phone Follow-Up Survey

For women who either moved out of Lilongwe or did not visit the Good Health Kauma Clinic, the field team attempted to contact them by phone up to a maximum of 3 times. Women who were reachable by phone were invited to participate in a short phone follow-up survey. To maintain a respondent’s privacy during an attempt to reach her by phone, the field enumerator conducting the call left no indication of the reason for the phone attempt (eg, voicemail, SMS text message) on the respondent’s phone if there was no response to a call. The phone follow-up survey instrument took no more than 20 minutes and was identical to the clinic-based follow-up survey instrument.

#### Home-Based Survey

For women who did not visit the Good Health Kauma Clinic and who were also uncontactable by phone, the field team attempted to contact them at their homes up to a maximum of one time. Women who were reachable at their homes were invited to participate in a home-based follow-up survey, which was administered in person by a field enumerator. The home-based follow-up survey instrument took no more than 20 minutes in total and was identical to the clinic-based and phone-based follow-up survey instruments. The survey was administered in a private room at a woman’s home.

All protocols related to the consent process and in-person survey administration followed during the baseline survey were also followed for the clinic, phone, and home surveys. Before administering each survey, the field enumerators clearly explained the purpose of the follow-up to the respondents and asked for their consent to participate. For in-person clinic-based and home-based surveys, written consent from respondents was solicited, whereas verbal consent was obtained from participants who were contacted for the phone survey. Following the receipt of consent, the enumerators would then ensure the confidentiality and privacy of responses by asking the respondent to find a private room or space in their household (for the baseline, counseling, phone follow-up, and home follow-up surveys) or at the clinic (for clinic-based surveys) where their responses could not be overheard. For each woman, survey data were collected up to a total of 3 time points (at baseline, at the counseling stage, and at the follow-up stage).

### Participant Compensation

All women who participated in the study received a small token of appreciation (3 bars of soap, a monetary equivalent of MWK 500 [US $0.66]) after completing the baseline survey. No monetary compensation was provided to participants for participating in the study, which served to minimize coercion. Participants were not informed of these tokens until the baseline survey had been administered.

All participants received a mobile phone credit of MWK 100 (US $0.14) by means of a mobile airtime transfer. To avoid coercion into participation, women were not informed of this airtime transfer until after the counseling session had been administered.

### Primary Outcomes

Key primary outcome variables include the following:

- Attitude or knowledge of family planning, including knowledge of family planning and perceptions toward contraception (eg, intentions to use)
- Contraceptive use, including changes in contraceptive use, changes in method mix, and adherence to methods (compliance, switching, and discontinuation)
- Contraceptive preferences, including the ideal contraceptive method and the most valued contraceptive method attribute
- Pregnancy and fertility outcomes, including pregnancy status, parity, delivery in a facility, months since last birth, wantedness of last birth, and intentions to become pregnant in the future
- Use of local health facilities, including the frequency of visits to local clinics and frequency of receipt of family planning counseling services
- Husband’s preferences, including the approval of family planning and fertility preferences
- Women’s autonomy, empowerment, and decision making

### Secondary Outcomes

A range of secondary outcomes were collected in each survey wave: (1) women’s education, employment, work, and income; (2) household assets; (3) postpartum treatment from last birth; (4) sexual and marital well-being; (5) husband’s employment, work, education, and earnings; (6) household bargaining and women’s autonomy and empowerment; and (7) trust in local health facilities. Outcomes were collected according to the schedule outlined in Table 3. All survey instruments (baseline, counseling, and follow-up) and intervention monitoring tools to track participants and intervention uptake over the course of the study are available upon request.

**Table 3:** Outcome measures and instruments.

Outcome or instrument	Baseline	Counseling	Follow-up
Attitude or knowledge of family planning	✓^a^	✓	✓
Contraceptive use	✓	✓	✓
Pregnancy and fertility outcomes	✓	✓	✓
Contraceptive preferences	✓	✓	✓
Use of local health facilities	✓	—^b^	✓
Husband’s preferences, approval of family planning, and fertility preferences	✓	✓	✓
Women’s autonomy, empowerment, decision making, and household bargaining	✓	✓	✓
Women’s education, work, income, and employment	✓	✓	—
Household assets	✓	—	—
Postpartum treatment from last birth	✓	—	—
Sexual and marital well-being	✓	—	—
Husband’s employment, work, education, and earnings	✓	✓	—
Trust in local facilities	✓	—	✓
Spillover of the intervention between treatment groups	—	—	✓

^a^The outcome was collected in the survey wave.

^b^Not available. The outcome was not collected in the survey wave.

### Survey Instruments and Monitoring Tools

A paper version of the baseline survey instrument, the counseling questionnaire, the clinic-based survey instrument, the home-based survey instrument, and the phone survey instrument are provided in Multimedia Appendix 2. Baseline surveys were administered in a private room in the woman’s place of residence and lasted approximately 2 hours. Short breaks of 5 to 10 minutes were given to the respondents at the end of each section, and additional breaks were taken at the request of the participant and at other scheduled times (eg, mealtime and picking up children from school) as needed. Surveys were conducted in Chichewa, the local language, and strictly follow the format of the questionnaires, which were electronically programmed into Android tablets. To successfully track our participants over time, we collected identifiable data, including names and contact information, names and background characteristics of other household members, household addresses, contact phone numbers and emails, and GPS coordinates of the household. For the purposes of minimizing loss to follow-up, we took photographs of the household and of the survey respondents, and we asked respondents to provide the names and contact information of 2 contacts who did not live in the household and who could be contacted if the respondents could not be directly located in the follow-up period. To protect participant privacy and ensure confidentiality, all identifiable data are appropriately stored and secured in accordance with the data security measures described in the Data Safety and Monitoring section below.

### Analysis Plan

The analysis of quantitative study data will be conducted using STATA and R (R Foundation), where appropriate. Descriptive analysis will be performed for all variables, and unadjusted comparisons between experimental arms will be conducted. Descriptive statistics, including frequencies, means, and SDs, will also be performed. Chi-square tests and one-tailed *t* tests will be used to examine associations in the data. A probability value of less than .05 will be considered statistically significant for all the statistical tests that are conducted. Continuous variables will be tested for normality, and nonnormal values will be categorized or appropriately transformed. Given our hypotheses regarding the impact of our intervention on our key outcomes, one-sided hypothesis tests will be conducted for all our main analyses.

Our main econometric specifications will (1) estimate the intent-to-treat (ITT) effects of our family planning intervention on fertility intentions and preferences, contraceptive intentions and preferences, intention to switch methods, and other outcomes related to contraceptive behaviors by directly regressing our outcomes of interest on a binary variable indicating receipt of the intervention and (2) estimate the effect of interventions on downstream behavioral outcomes (such as women’s uptake of contraceptive options and the concordance between their revealed preference and their stated preference) using a 2-stage least squares model, in which the intermediate counseling variable will be instrumented by the treatment. The main econometric specification for estimating the ITT effect of our family planning intervention is defined as follows:

*Y_it_* = *β_0_* + *β_1_T_it_* + *X_it_ζ* + *Y_i0_* + *η_i_* + *ε_it_*

where *Y_it_* is the outcome variable of interest for woman 𝑖 in time period 𝑡=0, 1, 2 for baseline (time period 0) and follow-up (time periods 1 and 2), respectively; *T_it_* is an indicator of assignment to 1 of the 3 treatment arms; *X_it_* is a vector of individual-level covariates that are controlled for in the analysis; η_i_ is the individual-specific fixed effect; *Y_i0_* is the baseline level of the outcome; and *ε_it_* is the error term. Here, the outcome variables of interest include immediate, intermediate, and long-term outcomes described in the previous sections.

We will conduct several subgroup analyses to examine how our family planning intervention effects vary across key subpopulations. Subgroups of interest include women who have or do not have their husbands’ approval for accessing contraceptive methods, women who have or have not previously used family planning methods, women whose most valued method attribute is or is not effective, working or nonworking women, higher or lower educated women, counseling sessions administered in the morning or afternoon, women who have more or less than 2 children, and counseling sessions of above or below the median length.

Finally, robustness checks (5% and 10% sample truncations and coarsening of independent variables) and falsification tests, which include placebo regression, simulation, and resampling methods, will be conducted to ascertain the strength and significance of our estimates. We will also conduct attrition-adjusted analyses to assess the extent of differential loss-to-follow-up on outcomes. In handling missing data, we will conduct a complete case analysis if the potential impact of the missing data is negligible, and we can conduct a series of sensitivity analyses, including *best-worst* case analyses, to generate plausible bounds of estimates across different scenarios of missingness. If we are able to assume that any missing data are missing at random, then we will conduct an ITT analysis using multiple imputation to adjust for missing data. We will then compare the ITT estimates from our multiple imputation analysis with our estimates from a complete case analysis, which would allow us to infer the extent to which missingness might impact our inference.

### Sample Size and Power Calculations

#### Target Sample and Key Outcomes

The sample size of this study was powered to primarily identify the independent effects of tailored family counseling and male involvement on women’s contraceptive uptake and switching of methods. On the basis of the preliminary power calculations for the primary outcomes of interest, our target baseline sample consists of 700 eligible women who consent to participate in the study. Among these 700 women, 100 women were assigned to the control arm T0, and 200 women were assigned to each of the 3 intervention arms, namely, T1, T2, and T3.

Previous studies have found that one-fifth of women of reproductive age were either pregnant or had given birth to a child in the past 6 months [40]. To meet our target sample size of 700 women who would be eligible to participate in our study, we need to recruit a total of 940 women if we conservatively assumed a combined ineligibility or refusal rate of 25%.

We have powered our study to detect effects on the following outcomes: (1) contraceptive uptake and (2) intention to switch from the current contraceptive method.

#### Contraceptive Prevalence: Partner Invitation Interventions

Using modern contraceptive prevalence estimates for urban area of Malawi from the 2010 Malawi DHS, we assume a modern contraceptive prevalence rate of 50% among women aged 18-35 years who are currently not pregnant and who did not give birth in the 6 months before the baseline. To infer a potential effect size for our intervention, we look to evidence to a study from Zambia [28], which found that male involvement in family planning care-seeking decreased the probability of receiving family planning services by 19% over a 2-year study period. Our study differs from this study in that we do not require husbands to participate in the counseling session; instead, we leave it up to the woman to decide whether she would like to invite her husband to counseling or not. In designing our invitation in this way, a selection effect might exist, in which husbands who are more supportive of contraceptive use are more likely to comply with the intervention and participate in a joint counseling session. As a result, our male partner invitation intervention would likely lead to an increase in contraceptive prevalence.

Assuming an attrition-adjusted sample of 700 women, with 400 women assigned to partner invitation arms, T1 and T3, compared with 300 women assigned to noninvitation arms, T0 and T2, we will have 75% power to detect a percentage point increase of 10 in the contraceptive prevalence rate from 50% to 60% (α=.05, two-sided). If, instead, we use a one-sided test, then we estimate that we would have 85% power to detect the same 10 percentage point increase in contraceptive prevalence.

Table 4 presents the levels of power (1−β) that could be achieved for various minimum effect sizes for the modern contraceptive prevalence use, assuming a baseline contraceptive prevalence rate of 50% in both the intervention and control arms and a fixed endline sample size of 700 women.

**Table 4:** Power calculations for contraceptive prevalence rate, partner invitation intervention.

Control CPR^a^, n (%)	Intervention CPR, n (%)	Significance level, α	Control sample size, n	Intervention sample size, n	Power
150 (50)	236 (59)	.05	300	400	0.66
150 (50)	240 (60)	.05	300	400	0.75
150 (50)	244 (61)	.05	300	400	0.83
150 (50)	248 (62)	.05	300	400	0.89

^a^CPR: contraceptive prevalence rate.

#### Intention to Switch Methods: Partner Invitation Interventions

Our measure of a woman’s intention to switch to another contraceptive method is proxied by her answer to the question, “if you had the choice and ability to switch to another family planning method, would you choose to switch?” An intervention to encourage women to invite their husbands or male partners to counseling could have 2 potential effects on women’s stated preferences for family planning methods. On the one hand, male involvement in counseling may encourage women to change their contraceptive preferences by allowing women to more actively retain and interpret counseling messaging [15,16] and through increased communication between partners [20]. On the other hand, male involvement in counseling might discourage women from changing their stated preferences if they feel compelled to hide their true preferences from their partners [41]. On the basis of the existing evidence on male involvement, we hypothesize that male involvement in counseling is likely to increase a woman’s intention to switch methods. Assuming an attrition-adjusted sample of 700 women, with 400 women assigned to partner invitation arms, T1 and T3, compared with 300 women assigned to noninvitation arms, T0 and T2, we will have 86% power to detect a 10 percentage point increase in women’s intention to switch methods in the intervention arm from 20% to 30% (α=.05, two-sided). If, instead, we use a one-sided test, then we estimate that we would have 92% power to detect the same 10 percentage point increase in intentions to switch.

Table 5 presents the levels of power (1−β) that could be achieved for various minimum effect sizes for the likelihood of women’s intention to switch contraceptive methods, assuming a baseline switching rate of 20% in both the intervention and control arms and a fixed endline sample size of 700 women.

**Table 5:** Power calculations for intentions to switch methods, partner invitation intervention.

Control likelihood, n (%)	Intervention likelihood, n (%)	Significance level, α	Control sample size, n	Intervention sample size, n	Power
60 (20)	112 (28)	.05	300	400	0.69
60 (20)	116 (29)	.05	300	400	0.78
60 (20)	120 (30)	.05	300	400	0.86
60 (20)	124 (31)	.05	300	400	0.91

#### Contraceptive Prevalence: Short, Targeted Counseling

Following a similar calculation to Table 4, we assume a modern contraceptive prevalence rate of 50% among women of reproductive age who are neither currently pregnant nor have given birth in the past 6 months at baseline. Given the lack of evidence on the role of information quantity during counseling on women’s contraceptive choices, we made some assumptions on the expected minimum detectable effect size of our targeted counseling intervention relative to the standard counseling process. Assuming an attrition-adjusted sample of 700 women, with 400 women assigned to the targeted counseling arms, T2 and T3, compared with 300 women assigned to the standard counseling arms, T0 and T1, we will have 75% power to detect a 10 percentage point increase in the modern contraceptive prevalence rate in the intervention arm from 50% to 60% (α=.05, two-sided). If, instead, we use a one-sided test, then we estimate that we would have 85% power to detect the same 10 percentage point increase in contraceptive prevalence.

Table 6 presents the levels of power (1−β) that could be achieved for various minimum effect sizes for modern contraceptive prevalence use, assuming a baseline contraceptive prevalence rate of 50% and a fixed endline sample size of 700 women.

**Table 6:** Power calculations for contraceptive prevalence rate, targeted counseling intervention.

Control CPR^a^, n (%)	Intervention CPR, n (%)	Significance level, α	Control sample size, n	Intervention sample size, n	Power
150 (50)	236 (59)	.05	300	400	0.66
150 (50)	240 (60)	.05	300	400	0.75
150 (50)	244 (61)	.05	300	400	0.83
150 (50)	248 (62)	.05	300	400	0.89

^a^CPR: contraceptive prevalence rate.

#### Intention to Switch Methods: Short, Targeted Counseling

Following a similar calculation to Table 5, we assume an attrition-adjusted sample of 700 women, with 400 women assigned to the targeted counseling arms, T2 and T3, compared with 300 women assigned to the standard counseling arms, T0 and T1, and we calculate that we will have 86% power to detect a 10 percentage point increase in women’s intention to switch methods in the intervention arm from 20% to 30% (α=.05, two-sided). If, instead, we use a one-sided test, then we estimate that we would have 92% power to detect the same 10 percentage point increase in intentions to switch.

Table 7 presents the levels of power 1−β that could be achieved for various minimum effect sizes for intention to switch contraceptive methods, assuming a baseline switching rate of 20% and a fixed endline sample size of 700 women.

**Table 7:** Power calculations for intentions to switch methods, targeted counseling intervention.

Control likelihood, n (%)	Intervention likelihood, n (%)	Significance level, α	Control sample size, n	Intervention sample size, n	Power
60 (20)	112 (28)	.05	300	400	0.69
60 (20)	116 (29)	.05	300	400	0.78
60 (20)	120 (30)	.05	300	400	0.86
60 (20)	124 (31)	.05	300	400	0.91

### Dissemination Plan

In a similar fashion to Karra and Canning [40], we formed a partnership with IPA Malawi, a US-based nonprofit research organization with operations in 42 different countries. Using rigorous research techniques, IPA works to develop and test solutions to real-world problems faced by the poor in developing countries. IPA comprises a group of leading academic researchers in development economics, behavioral economics, and psychology, based both in the United States and in developing countries. IPA Malawi has provided extensive technical and research assistance in health and development to many governmental and nongovernmental organizations in Malawi, including the MOH and the World Bank. For these reasons, we chose to partner with IPA Malawi for the study and worked with the IPA Malawi management team and a hired team of surveyors, field managers, intervention staff (family planning counselors and driver), and other support staff to conduct the fieldwork. IPA Malawi’s primary role in the study was to conduct the local field research activities, including (1) hiring, training, and management of the local field staff; (2) data collection, monitoring, and evaluation; (3) implementation of the intervention; and (4) assisting the investigators with the dissemination of results in Malawi.

In addition, we worked closely with the Malawi MOH and the Malawi RHD on dissemination and outreach activities for this study. Flip charts used for counseling were provided by the RHD for the duration of the study. Neither the MOH nor the RHD were directly involved in any of the data collection, intervention implementation activities, or other research-related activities. Finally, we collaborated with PSI Malawi, a nonprofit global health organization with programs targeting malaria, child survival, HIV, and reproductive health. PSI provided our team with family planning methods (male or female condoms) and other training resources to facilitate the administration of the counseling process.

Aggregate summary statistics and final peer-reviewed publications will be shared with participants, key partners (IPA Malawi, Dimagi, Good Health Kauma Clinic), the Malawi MOH, the Malawi RHD, and the Malawi National Statistics Office. Individual survey responses will not be shared among participants verbally, by recording, or in writing. Each interviewee’s responses will remain confidential, as per the terms of their consent to study participation.

We will produce output in peer-reviewed journals, working papers, and policy briefs that are accessible to academics, policy makers, and practitioners and contribute to policy changes in this area. Aggregate results and final publications will be disseminated to the community and local institutions where the research was conducted (University of Malawi, among others). The research team will also present intervention findings at local and national venues, including annual meetings of professional organizations, community gatherings, and meetings with local service providers. These meetings and conferences will be valuable opportunities for us to see the rigor of work that our contemporaries are working toward and will allow us to contribute our own progress in the field of economic outcomes of reproductive health.

Our work will also be effectively disseminated to practitioners via local partnerships with FHI360, BLM, PSI Malawi, the World Bank, the RHD, and the MOH in Malawi. We have worked with these organizations during the study design phase to ensure that the interventions are appropriate for the country setting. Upon the completion of the intervention, the local partners can leverage the study findings to expand or tailor their services to help women achieve their family planning goals within a specific country context. We will also share our descriptive and analytical findings with members of the community who are engaged in advocacy efforts.

Our dissemination efforts are engrained in our interventions from the outset. We will tailor our information packs and materials for women in Malawi based on knowledge gained from preliminary studies of the family planning environment. Consulting practitioners and local agencies will develop the information packs and other counseling components of the intervention with wording that has integrity and clarity, that uses language as commonly used by local women, that is pitched at a layperson level, and that is sensitive to local culture and practices. This dissemination of information as a part of the intervention will help us learn how to further disseminate the results of our research to the women in the communities we are working in.

### Protocol Amendments and Modifications

All protocol amendments and modifications were submitted to both the BU IRB and the Malawi NHSRC for approval. Before the implementation of any modified protocols, the approval of the modification had to be received by both ethics committees. Any determination of the extent to which modifications were communicated to the study participants was made by both ethics committees at the time of review.

### Data Confidentiality

Our data confidentiality protocols follow those established by Karra and Canning [40]. To effectively monitor participants over the study period, identifiable data on participants’ demographic backgrounds and personal contact information (household addresses, phone numbers, emails, and GPS locations) were collected. In addition, photographs of participants and of the households were taken to facilitate identification at the follow-up stage. All identifiable data collected from surveys (both baseline and follow-up surveys) and from the intervention were administered in an electronic computer-assisted personal interview format using the CommCare survey management system. Electronic survey data were collected by interviewers on Android-based tablets, and data were securely transferred from the Android tablets onto a CommCare-supported secure cloud server at the end of each working day. All Android tablets were used for data collection only, and tablet settings were adjusted so that field staff was blocked from accessing apps that were not relevant for data collection (eg, internet browsing, social media, and email). The CommCare cloud server was Health Insurance Portability and Accountability Act (HIPAA)–compliant and met all the necessary security requirements for storing level 4 identifiable data. Once the data were securely transferred to the cloud server, the survey record on the Android tablet was immediately erased. A technical overview of the CommCare system, including descriptions of the data transfer process and the HIPAA-compliant storage system, can be found on the Dimagi website [42]. An electronic version of the CommCare Terms of Us or End User License Agreement can also be found on the Dimagi website [43].

All data uploaded to the CommCare cloud server were encrypted and password-protected in accordance with level 4 data security and storage regulations. For each collected data case, which comprised a woman’s (and her husband’s) data records, all personal identifiable data were separated from the other nonidentifiable data. The deidentified data were uploaded to an encrypted password-protected File Transfer Protocol (FTP) site on a daily or weekly basis and circulated to the project PIs for analysis purposes. The identified data were stored on the CommCare secure encrypted server and could only be accessed for the purpose of revisiting the households at the 1-month follow-up period. After the study ended, the IPA Malawi research site maintained the identified data in an encrypted file on a secure server, and only deidentified data sets remained available for analysis purposes after the end of the study.

Every effort will be made to ensure that participation in this study and all records about participation remain confidential. As previously stated, all confidential identifiable data were secured by trained study personnel upon collection. Data were collected by trained staff and fully deidentified as soon as possible. We will work with Dimagi to set up a data management system that meets the following requirements:

1. Raw electronic survey data will immediately be transferred to the CommCare secure cloud server once it has been collected on the Android-based tablets. Following the transfer, the data from the Android tablets will be automatically erased.
2. All identifying information will be separated from the raw electronic survey data immediately after collection and secure transfer to the cloud server, and a unique computer-generated ID number, which is created when a respondent is registered in the study database, will be assigned to each case. Coded, deidentified data files will be stored separately from the code list and the identified data files. Following the completion of all field research activities, all respondents will only be referred to by their ID number in the deidentified data set. Only the PIs will have access to the linkages to the underlying identifiable files. Identifiable electronic data will be encrypted, password-protected, and securely stored on the CommCare-protected cloud server, and one copy of the data will be stored on a password-protected target computer.
3. Restrictions will be placed on nonauthorized users from accessing certain data or features by assigning them permission levels. This includes restricting access to any identifying data that would violate HIPAA or other privacy standards. Each study team member will be assigned 1 of 3 permission levels, which will provide them with varying levels of access, from no access (level 0) to full access (level 2).

Identifiable hard-copy data, including signed consent forms, were stored in locked cabinets in access-limited rooms at the IPA Malawi office. All study computers that are used for descriptive analysis of the deidentified data are password-protected, and only study staff who are cleared to view the data will have the password. All electronic data, both on the CommCare secure cloud server and on any study computer, will be encrypted and password-protected. This information will be accessible only to the research team.

All staff members of the study were required to sign a data confidentiality agreement. The data were stored in a relational database. Usernames and passwords are required to access the data. A security policy is used to ensure that the passwords are updated on a regular basis.

Data sharing of deidentified data between the immediate research team will be conducted in person—a USB key will be used to transfer the deidentified data from one secure hard drive to the next, and data will then be deleted from the USB key. The USB key will be used only for storing and transferring research material between the research team members mentioned above, and it will not be used for storing or transferring other files that are unrelated to the study. Four sets of deidentified data will be stored, one for each of the immediate research team members.

Deidentified data will be transmitted from Malawi to BU via secure file transfer (through the BU system). Colleagues in Malawi will be offered guest access to transfer the data. All hard-copy data and electronic data will be retained for 7 years after study closure, after which it will be destroyed (shredding hard copies and permanently deleting all electronic files).

### Data Safety and Monitoring

The PI, MK, will take the overall responsibility for the safety, monitoring, and review of the data. He and KZ will oversee the weekly review of all data collected in the study and were present during the administration of the baseline, intervention, and follow-up surveys. He and the coinvestigators of the study will ensure that the data are treated as confidential and stored in a secure location, as detailed above.

For the proposed research, the local project manager at IPA Malawi; the local coinvestigators, Bagrey Ngwira and Abiba Longwe; and the PI, MK, will review adverse events and protocol deviations. This information will then be provided to the institutional review boards at the BU and the NHSRC in Lilongwe. Unanticipated adverse events and protocol deviations will be immediately reported to both the BU IRB and the NHSRC in writing within 5 business days. As per our stated reporting protocols, we will also inform local community leaders of any adverse events related to participant safety, domestic violence, and abuse within the household, and we will refer participants and other members of the household to their nearest victim support unit and to the Department of Social Welfare at the Lilongwe District Council Office. In addition to reporting any events from the baseline and follow-up surveys, we will make quarterly reports on consultations and reimbursements for family planning services for women.

We do not anticipate that there will be any research-related injuries. Bagrey Ngwira, Abiba Longwe, and the IPA Malawi project manager trained the field staff (surveyors, field managers, and interventionists) in first aid and will be on call via mobile phone throughout the entire duration of the study. The field team was trained to recognize basic signs of physical and psychological injuries and were instructed to immediately report any injuries that were incurred during the interview with the IPA Malawi project manager. With the help of Bagrey Ngwira, Abiba Longwe, and the IPA Malawi project manager, we established a link with a local primary clinic in Lilongwe and will refer respondents to this clinic if they experience research-related injuries. We will identify the emergency care facility or hospital that is nearest to the interview location before each interview, and we will refer respondents to this facility in case of a medical emergency.

### Regulatory Compliance

MK, KZ, Bagrey Ngwira, and the IPA Malawi project manager will be the immediate supervisors of the study staff in the field. They will communicate with the study staff on a weekly basis and will ensure that the study protocol and IRB regulations are being followed. On-site supervision will allow the management team to provide support for staff as well as quality assurance and confidentiality of study data. In addition, the local team and Boston-based team will be in regular contact via email in the interim to discuss the progress of the study protocol procedures. MK and KZ will travel regularly to Malawi over the course of the study period and particularly during the data collection phases to monitor field activities. All regulatory documentation will be maintained for 7 years after IRB study closure.

### Authorship Eligibility Guidelines

A publication committee comprising MK, the overall PI, and KZ has been established to address and decide on all matters related to access to project data and publications using such data. All guidelines for data access and publications are outlined in the publications committee terms of reference document (available upon request).

### Availability of Data and Material

Following our own use and analysis of the data (a minimum 1-year period), we hope to open access to deidentified baseline and follow-up survey data at no cost to authorized users. Only deidentified data will be available for download via a secure website, through which authorized users can download deidentified survey data files for legitimate academic research. To access the data, prospective users must first register on the secure website and must then create a new research project request. The request must include a project title and a description of the analysis that the user proposes to perform with the data. The requested data should only be used for research or study purposes. To request the same data for another purpose, a new research project request needs to be submitted. Requests for data access will then be reviewed by the PI, who can then grant or deny access to the user. All publications that users produce from the data set must appropriately acknowledge the data source and project from which the data were collected. Once downloaded, the data sets must not be passed on to other researchers without written consent from the PI. All reports and publications based on the requested data must be sent via email to the BCS in a portable document format or as a printed hard copy.

## Results

### Recruitment, Study Sample, and Randomization

Field activities for the baseline survey began with field staff hires, training, and piloting of the survey instrument in June 2019 and continued through September 2019. During the 3-month baseline survey period, 1122 women were approached and screened using the eligibility criteria. On the basis of the eligibility screening, of the 1122 women, 823 (73.4%) were found to be eligible to participate in the study. Of the 823 eligible women, 801 agreed to go through the consent form with the enumerator, and 785 (95.3% of the eligible sample) further consented to participate and were subsequently enrolled in the study. Of these 785 women, 782 (99.6%) completed the baseline survey and were eligible to be randomized into 1 of the 3 intervention groups or the control group. From this baseline sample, 223 women were randomly assigned to treatment (T1) with long counseling and husband invitations, 225 women were assigned to treatment (T2) with short counseling and no husband invitations, 228 women were assigned to treatment (T3) with short counseling and husband invitations, and 108 women were assigned to treatment (T0) with long counseling and no husband invitations. Among the 782 women in the final sample, 41 were interviewed as a part of a preliminary pilot study to test the feasibility of the survey instruments and implementation of the intervention. As part of the intervention rollout, these 41 respondents were also randomized into the T1 (n=10), T2 (n=13), T3 (n=12), and control T0 (n=6) arms. The final analytic sample for the baseline survey comprised 782 eligible women, of which 223 were assigned to T1, 225 were assigned to T2, 228 were assigned to T3, and 108 were assigned to T0 (control). The experimental framework is illustrated in Figure 1.

### Intervention Activities

The rollout of the counseling intervention began shortly after randomization in September 2019. Four family planning counselors (registered nurses and midwives with previous counseling experience in family planning) were identified in August 2019 and were trained through September 2019 to administer the range of counseling services over a 3-month intervention period. Counseling in the intervention groups began in September 2019.

In addition to hiring 4 counselors, the study management team hired and trained a licensed taxi driver in March 2019 to assist with the implementation of the transportation component of the intervention. In March 2019, the management team also identified an obstetrician at the Kamuzu College of Medicine as a medical doctor on call. The obstetrician was asked to be responsible for (1) answering any calls from clients; (2) providing consultation services and information on the desired family planning methods for any woman who visited the clinic during the service period; (3) providing any support or consultation services over the phone; and (4) referring any client who may be experiencing health concerns, particularly those related to their use of family planning, to the management team for follow-up.

Counseling activities with women in the intervention group concluded in December 2019; however, other intervention activities (providing transportation to women to visit the Kauma Clinic for services and providing financial reimbursements to women for any family planning services that they obtain) continued until February 2020.

### Follow-Up Surveys

Follow-up surveys began in November, 2 months after the conclusion of the baseline survey. Field activities for the follow-up surveys began with hiring field staff, training, and piloting the follow-up survey instruments in November 2019, which continued through February 2020. During the 4-month follow-up survey period from November 2019 to February 2020, 767 women (including 727 women from the main study and an additional 40 women from the pilot phase of the study, but not including the 15 women who withdrew from the study before the start of the follow-up survey) were reached. Of the 767 women who were reached at the follow-up stage, 731 (95% of women who were eligible for follow-up) successfully completed the follow-up survey. In total, 51 women who were interviewed at baseline are estimated to be lost to follow-up.

### Analysis

All study-related field activities for the follow-up survey were completed on February 15, 2020. The cleaning of baseline, precounseling, postcounseling, and follow-up survey data are ongoing. A complete, cleaned survey wave includes the following: a recoded and indexed data set, a data codebook, a recode map and variable list, a final survey questionnaire, a final report and user manual for the survey wave, and data analysis files and templates. Analyses of the primary outcomes are ongoing and are expected to be completed by 2021.

## Discussion

### Principal Findings

The primary objective of this study is to investigate the extent to which user-centered approaches to counseling, by exploiting choice architectural elements around family planning and promoting male involvement, can affect a woman’s immediate stated preferences and realized preferences in a context where her preferences may be sensitive to a range of behavioral biases. Our main outcomes of interest include women’s stated preferred method of contraception, intention to switch to other contraception methods, uptake of their ideal contraceptive method, and realization of their ideal contraceptive preferences. In addition, by observing whether women will seek family planning following their counseling visit, we will examine the short-run stability of their stated preferences and the extent to which these stated preferences are subsequently realized in the face of barriers to use. Data examining the realization of women’s eventual contraceptive preferences (eg, contraceptive uptake, eventual method choice) were collected during clinic visits and at (phone or home) follow-ups across the four arms.

### Study Limitations

Our study has several limitations. Although the study is powered to independently test the effects of 2 user-centered counseling procedures on women’s stated and realized preferences, the sample size of this study may be too small to allow for the examination of interaction effects across treatment arms. As an exploratory exercise, we will examine these effects and present them as supplementary findings. Moreover, although our study can infer the manner in which women realize their preferences in response to counseling, it is not as straightforward to be able to disentangle a woman’s true individual preferences for family planning from her stated preferences in the presence of her partner, who is likely to influence her eventually realized choice (or lack of choice) of method. To this end, additional research is warranted to better understand how women’s true preferences are expressed and can be documented, even in cases where their partner is present. Similarly, it would be equally necessary to further explore the trade-off that women face between (1) making independent decisions to reflect their individual preferences and (2) incorporating their husband’s/family’s preferences to make jointly/socially better-off, but not necessarily individually better-off, decisions.

### Conclusions

Our study findings seek to inform women and communities of the local family planning environment. In addition, our findings will inform both clients and service providers of the role of user-centered, tailored family planning counseling in improving concordance between a woman’s preferences and her contraceptive use, which in turn would help her realize her family planning goals. The results may also help the MOH and other service providers to develop family planning programs that internalize the preferences of women and their partners for contraceptive methods, thereby achieving the goal of improving reproductive health outcomes in Malawi. More generally, our findings may also demonstrate to policy makers that the benefits of tailoring family planning services to women’s preferences are likely to extend beyond the health domain by improving empowerment outcomes. Such findings can be used to develop policies, programs, and interventions that aim to improve the health and well-being of women, couples, and households.

## Appendix

Multimedia Appendix 1 Survey instruments, recruitment scripts, and consent forms.

Multimedia Appendix 2 Family planning flip charts.

## Abbreviations

BCS: Balanced Counseling Strategy
BLM: Banja La Mtsogolo
BU: Boston University
DHS: Demographics and Health Survey
HIPAA: Health Insurance Portability and Accountability Act
IPA: Innovations for Poverty Action
IRB: institutional review board
ITT: intent-to-treat
MDHS: Malawi Demographics and Health Survey
MOH: Ministry of Health
NHSRC: National Health Sciences Research Committee
PI: principal investigator
PSI: Population Services International
RHD: Reproductive Health Directorate
