# Supplementary material for: User-Centered Counseling and Male Involvement in Contraceptive Decision Making: Protocol for a Randomized Controlled Trial
Source: JMIR Res Protoc. 2021 Apr 5;10(4):e24884. doi: 10.2196/24884 (PMC8056297; doi:10.2196/24884)
Supplement: Multimedia Appendix 1 [file resprot_v10i4e24884_app1.pdf]

MALAWI BEHAVIORAL BIASES STUDY

BASELINE SURVEY

Survey ID Generation

1. Field Manager ID Number ☐
2. Enumerator Identification Number (Enter 01-10): ☐☐
3. Survey Wave (Enter 1-3): ☐
4. District (Enter 1 for Lilongwe): ☐
5. Area Code (2 digits): ☐☐
6. Sector Code (2 digits): ☐☐
7. Cluster Number (2 digits): ☐☐
8. Household ID Number - 2 digits describing the number of the household that the enumerator is completing for that particular day. For example, if the household is the fifth being interviewed by that particular enumerator that day, the household ID number is '05'. ☐☐

The **survey ID number** is a 13-digit number that is created by joining the 8 numerical responses recorded above in order, from response 1 to response 8. An example of a complete respondent ID number is given below.

Example: For the 13th household that is interviewed by Enumerator 04 in Field Manager 1's team for the baseline survey (Wave 1) in Lilongwe (District 1) Area 47 (code 47), Sector 6 (code 06), Cluster 12, the **survey ID number** will be created as:

1	0	4	1	1	4	7	0	6	1	2	1	3

SURVEY ID NUMBER: ☐☐☐☐☐☐☐☐☐☐☐☐☐

Enter assigned number above and in the Interviewer Log Book.

PROCEED TO WOMAN RECRUITMENT SCRIPT – PAGE 2

WOMAN Recruitment Script – FIRST VISIT (Verbal, In Person)

INTRODUCTION

Hello. My name is ________________ and I am from Innovations for Poverty Action (IPA) Malawi based in Lilongwe. I would like to invite you to participate in a study in which we are trying to understand use of family planning and reproductive health services in Lilongwe, Malawi. We are interested in trying to understand some of the issues that married women face concerning family planning, maternal and child health, and childbearing. We are also collecting information about households. You may participate if:

- you are a married woman;
- you are between the ages of 18 to 35;
- you live in Lilongwe;
- you are neither pregnant now nor have given birth within the past 6 months;
- you have neither been sterilized nor had a hysterectomy;
- you have given birth to at least one child (one live birth) in your lifetime;
- you live with your husband

If you do not meet all of the requirements that I mentioned, please tell me now. (GO TO QUESTION 1)

DETERMINING ELIGIBILITY

No.	Question	Response	Skip Rules
1	Do you meet all of these requirements?	YES NO	IF NO, GO TO 2A<br>IF YES, GO TO 2B
2A	Thank you. Are there any other women who live in this household?	YES NO	IF NO, GO TO **END**<br>IF YES, GO TO 3
2B	Thank you. Are there any other women who live in this household?	YES NO	IF NO, GO TO **PURPOSE**.<br>IF YES, GO TO 3.
3	With your permission, I would meet them to see if they are eligible for this study.<br>MEET OTHER WOMEN IN THE HOUSEHOLD.<br>REPEAT INTRODUCTION TO EACH WOMAN.<br>IDENTIFY THE **YOUNGEST** ELIGIBLE WOMAN IN THE HOUSEHOLD.	YES NO	IF **YOUNGEST** ELIGIBLE WOMAN IS IDENTIFIED, GO TO **PURPOSE**.<br>IF **NO** ELIGIBLE WOMAN IS IDENTIFIED, GO TO **END**
**END**	Thank you for your time. I hope that you have a good day.		END VISIT.

PURPOSE

(TALK TO THE YOUNGEST ELIGIBLE WOMAN IN THE HOUSEHOLD)

NAME OF YOUNGEST ELIGIBLE WOMAN: ____

We want to talk to women like you who live in Lilongwe so that we can identify the best ways to help our community access family planning. We feel that perspectives from people like you can help to inform the Ministry of Health's approach to identifying potential barriers to use of family planning services so that we can then improve those services.

If you decide to participate, you will be asked to participate in two individual surveys over a two-month period. The first survey will take approximately 75 minutes respectively, and the second survey will take 20 minutes. In each survey, you will be asked several questions. Some of them will be about marriage, family planning, pregnancy, and your children. Others will be about education and employment. If you agree, you will participate in the first survey today, and we will return after two months to conduct the second survey with you. By returning after some time, we can learn more about how life changes for Malawian families.

I do not anticipate any physical risks to participating. Your responses to survey questions will be kept confidential, and at no time will your actual identity be revealed. Your participation may help to inform the community of the local family planning environment and may also help to improve access to family planning services in Lilongwe. The data you give me will be used for academic publications and may be used as the basis for articles or presentations in the future. That said, we won't use your name or information that would identify you in any publications or presentations. (GO TO QUESTION 5)

No.	Question	Response	Skip Rules
5	If you would like to participate in this study, please let me know now.	YES NO	IF NO, GO TO **END**<br>IF YES, GO TO 6
6	Thank you for your participation. Is now a good time to begin the first survey?	YES NO	IF NO, GO TO **END**<br>IF YES, GO TO **CONTACT**
**END**	Thank you for your time. I hope that you have a good day.		END VISIT.

CONTACT

Thank you. I will begin the survey shortly. Do you have any questions now? If you have questions later, you can contact one of the researchers, Mr. Patrick Baxter, at:

Mr. Patrick Baxter
Innovations for Poverty Action (IPA) Malawi
E-mail: pbaxter@poverty-action.org
Telephone: +265 1762424
Availability: Monday to Friday, 9 AM to 5 PM

Thank you for your time. DATE: ________________________________

PROCEED TO WOMAN CONSENT FORM – APPENDIX G1

Protocol Title: **The Malawi Behavioral Biases Study 2019, Wave I**	
Principal Investigator: **Mahesh Karra, Sc.D.**	
Description of Study Population: **Married women aged 18-35, living in Lilongwe, are neither currently pregnant nor have given birth within past 6 months, and living with husband**	
Version Date: **April 15, 2019**	

Malawi Behavioral Biases Study (MBBS) 2019
Field Research Informed Consent Form

Study Summary

The purpose of this research study is: 1) to understand use of family planning and reproductive health services among married women in Lilongwe, Malawi; 2) to learn about issues concerning family planning, maternal and child health, and childbearing; 3) to investigate how women make decisions in health, family planning, and childbearing; and 4) to understand the role of men in women's reproductive health decision-making.

Participants who take part in this study will participate in this study for five (5) months, from June 2019 to October 2019. During this time, participants will receive up to two (2) study visits to their homes by the MBBS field team.

Participants taking part in this study will receive a survey will ask about their marriage, pregnancy status, fertility preferences, and views on family planning, maternal and child health, and reproductive health. Participants will then receive a second survey after two months at a local clinic, by phone, or at the participant's home.

The risks of taking part in this research study are a potential loss of privacy. We will protect your privacy by labeling your information with a code and keeping the key to the code in a locked cabinet. If you are interested in learning more about this study, please read the rest of this form.

About this consent form

Please read this form carefully. This form provides important information about participating in research. You have the right to take your time in making decisions about participating in this research. You may discuss your decision with your family, your friends and/or your doctor. If you have any questions about the research or any portion of this form, please ask us. If you decide to participate in this research, you will be asked to sign this form. A copy of the signed form will be provided to you for your record.

Participation is voluntary

You are invited to take part in this research because:

- you are a married woman;
- you are between the ages of 18 to 35;
- you live in Lilongwe;
- you are neither pregnant now nor have you given birth within the past 6 months;
- you have neither been sterilized nor had a hysterectomy;
- you have given birth to at least one child (one live birth) in your lifetime;
- you live with your husband

It is your choice whether or not to participate. If you choose to participate, you may change your mind and leave the study at any time. Refusal to participate or stopping your participation will involve no penalty.

What is the purpose of this research?

The purpose of this research is:

1. To understand use of family planning and reproductive health services among married women in Lilongwe, Malawi.
2. To learn about issues concerning family planning, maternal and child health, and childbearing.
3. To investigate how women make decisions in health, family planning, and childbearing
4. To understand the role of men in women's reproductive health decision-making.

How many people will take part in this research?

About 700 women from Lilongwe will take part in this research.

How long will I take part in this research?

It will take about five (5) months to complete the entire study. During this time, you will be asked to participate in two (2) individual surveys.

What can I expect if I take part in this research?

As a participant, you will be asked to participate in two (2) individual surveys over a five-month period. The first survey will take approximately 75 minutes, and the second survey will be conducted in two months and will take about 20 minutes. In each survey, you will be asked several questions. Some of them will be about marriage, family planning, pregnancy, and your children. Others will be about education and employment. Finally, we will also collect information that will help us to contact you for future interviews. This information includes: your household address and location, your mobile phone number, and the contact information of two people whom you know and who do not live in your household, but would know how to reach you. With your permission, we would also like to take your photo, which will help us to identify and contact you in the future.

If you agree, you will participate in the first survey today, and we will return after two months to conduct the follow-up survey with you. By returning after some time, we can learn more about how life changes for Malawian families. The information that we gather from you and from other community members will help us better understand key issues in women's health in Malawi. You may choose, without any penalty, to skip any questions, to discontinue the survey at any time, or to exclude use of your responses.

What are the risks and possible discomforts?

I do not anticipate any major physical risks to participating. However, some of the questions may cause discomfort or embarrassment. The main risk of allowing us to use and store your information for research is a potential loss of privacy. We will protect your privacy by labeling your information with a code and keeping the key to the code in a locked cabinet.

Are there any benefits from being in this research study?

There are no direct benefits from participating in this study. However, this is a chance for you to share your thoughts and experiences regarding marriage, children, fertility, and family planning. Your participation will help to inform the community of the local family planning environment and may also help to improve access to family planning services in Lilongwe. You may also be eligible to receive additional health information and/or services that are available in Lilongwe.

Will I be paid to participate in this study?

All eligible participants will receive a small token of appreciation (three bars of soap) for taking part in our study.

Can my taking part in the research end early?

You may decide not to continue in the study at any time without it being held against you. You may withdraw by informing me that you no longer wish to participate (no questions will be asked). You may also skip any question during the survey, but continue to participate in the rest of the study. The person in charge of the research can also remove you from the research at any time without your approval for any reason. If you decide to leave the study, please contact the investigator.

Use of Your Study Information

We may use your **study information** for future research studies **and/or may send** your study information to other research collaborators. If we do this, we will remove identifiers such as your name. We will label all of your study information with a code. Nobody outside of this study will know which study information is yours.

If I take part in this research, how will my privacy be protected? What happens to the information you collect?

Your responses to survey questions will be kept confidential. At no time will your actual identity be revealed. You will be assigned a random numerical code. Anyone who helps me with this research will only know you by this code. The key code linking your name with your number will be kept in a locked file cabinet in a locked office in the United States, and no one else will have access to it. It will be destroyed as soon as I have finished analyzing your responses to my questions. The data you give me will be used for academic articles that are currently being written and may be used as the basis for articles or presentations in the future. We won't use your name or information that would identify you in any publications or presentations. Your name and other identifying information will always be kept anonymous. Data collected, including your identifiable information, may be seen by the Boston University Institutional Review Board (IRB) that oversees the research.

If I have any questions, concerns or complaints about this research study, who can I talk to?

If you have questions or concerns about this research, please contact **Mr. Patrick Baxter**, who is locally in charge of this study.

Mr. Patrick Baxter
Innovations for Poverty Action (IPA) Malawi
E-mail: pbaxter@poverty-action.org
Telephone: +265 1762424
Availability: Monday to Friday, 9 AM to 5 PM

Please contact Mr. Baxter:

- If you have questions, concerns, or complaints,
- If you would like to talk to the research team,
- If you think the research has hurt you, or
- If you wish to withdraw from the study.

The Local Investigator of this study is **Dr. Bagrey Ngwira**. He can be reached at:

Innovations for Poverty Action (IPA) Malawi
E-mail: bagreyngwira@gmail.com
Telephone: +265 999554003, +265 888554003
Availability: Monday to Friday, 9 AM to 5 PM

This research has been reviewed by the Malawi National Health Sciences Research Committee (NHSRC) and by the Boston University International Review Board. If you wish to speak with someone from the IRB, please contact the Charles River IRB Office at 617-358-6115 or 25 Buick St, Room 157, Boston, MA for any of the following:

- If your questions, concerns, or complaints are not being answered by the research team,
- If you cannot reach the research team,
- If you want to talk to someone besides the research team,
- If you have questions about your rights as a research participant, or
- If you want to get information or provide input about this research.

You may also contact the Malawi NHSRC at Ministry of Health, P.O. Box 30377, Lilongwe 3, Malawi, by phone at +265 1 726 422/418, or by e-mail at mohdoccentre@gmail.com.

Statement of Consent

I have read the information in this consent form including risks and possible benefits. All my questions about the research have been answered to my satisfaction. I understand that I am free to withdraw at any time without penalty or loss of benefits to which I am otherwise entitled.

I consent to participate in the study.

SIGNATURE

Your signature below indicates your permission to take part in this research

____
Name of participant

______________________________ ______________
Signature of participant Date

______________________________ ______________
Signature of person obtaining consent Date

____
Printed name of person obtaining consent

DATE ____________________

QUANTITATIVE SURVEY
BASELINE QUESTIONNAIRE
MALAWI BEHAVIORAL BIASES STUDY - WAVE I, 2019
BOSTON UNIVERSITY, IPA MALAWI

IDENTIFICATION (1)

NAME AND NO. OF THE DISTRICT ____________________

AREA . . . .

SECTOR . . . .

CLUSTER NUMBER . . . .

HOUSEHOLD ID NUMBER . . . .

HOUSEHOLD ADDRESS ____________________

DESCRIPTION OF HOUSE/ LANDMARKS ____________________

NAME OF HOUSEHOLD HEAD ____________________

PRIMARY PHONE NO. OF HH HEAD ____________________

NAME AND LINE NUMBER OF RESPONDENT ____________________

PRIMARY PHONE NO. OF RESPONDENT ____________________

ALTERNATE PHONE NO. OF RESPONDENT ____________________

E-MAIL OF RESPONDENT ____________________

GPS COORDINATES					
		N/S	D D	X X	. X X X
	LATITUDE				
		E/W	D D	X X	. X X X
	LONGITUDE				

INTERVIEWER VISITS

	1	2	3	FINAL VISIT
DATE	______ D D M M Y Y Y Y	______	______	DAY [ ][ ] MONTH [ ][ ] YEAR [ ][ ][ ][ ]
INTERVIEWER'S NAME	______	______	______	INT. NUMBER [ ][ ]
RESULT*	______	______	______	RESULT [ ]
NEXT VISIT: DATE	______	______		TOTAL NUMBER OF VISITS [ ]
TIME	______	______		

*RESULT CODES:

1 COMPLETED
2 NO HOUSEHOLD MEMBER AT HOME OR NO COMPETENT RESPONDENT AT HOME AT TIME OF VISIT
3 ENTIRE HOUSEHOLD ABSENT FOR EXTENDED PERIOD OF TIME
4 POSTPONED
5 REFUSED
6 DWELLING VACANT OR ADDRESS NOT A DWELLING
7 DWELLING DESTROYED
8 DWELLING NOT FOUND
9 MOVED TO ANOTHER VILLAGE/TOWN/DISTRICT
A MOVED TO NEIGHBORING COUNTRY
B SHIFTED TO UNKNOWN LOCATION
C TRANSFERRED DUE TO WORK/EDUCATION
D OTHER ______ (SPECIFY)

TOTAL PERSONS IN HOUSEHOLD [ ][ ]

LINE NO. OF RESPONDENT TO HOUSEHOLD QUESTIONNAIRE [ ][ ]

FIELD MANAGER	INTERVIEWER	OFFICE EDITOR	KEYED BY
NAME ______ [ ]	NAME ______ [ ][ ]	[ ][ ]	[ ][ ]

THIS PAGE IS INTENTIONALLY BLANK

INTRODUCTION

In this section, I would like to ask you some questions about your household. The questions in this section usually take about 10 to 15 minutes. All of the answers you give will be confidential and will not be shared with anyone other than members of our survey team. You don't have to be in the survey, but we hope you will agree to answer the questions since your views are important. If I ask you any question you don't want to answer, just let me know and I will go on to the next question or you can stop the interview at any time.

In case you need more information about the survey, you may contact the person listed on the card that has been given to your household.

PRESENT CARD WITH CONTACT INFORMATION TO REMIND RESPONDENT

Do you have any questions?
May I begin the interview now?

HOUSEHOLD ROSTER WITH NUMBER OF MEMBERS

NO.	QUESTIONS AND FILTERS	CODING CATEGORIES	SKIP
	I would like to start by asking you about the number of members in your household.	[ ][ ]	
H1	How many usually live in your household (excluding guests)?<br><br>RECORD NUMBER. IF OTHER, RECORD 96. IF DK, RECORD 88. IF REFUSED, RECORD 99.	[ ][ ]	
H2	How many people were guests of the household who stayed here last night?<br>RECORD NUMBER. IF OTHER, RECORD 96. IF DK, RECORD 88. IF REFUSED, RECORD 99.	[ ][ ]	
H2A	HOUSEHOLD CONFIRMATION:<br>THERE ARE H1 + H2 HOUSEHOLD MEMBERS IN THE HOUSEHOLD. IS THAT CORRECT? PLEASE CONFIRM THE NUMBER OF HOUSEHOLD MEMBERS. AFTER THIS POINT THERE IS NO GOING BACK!	YES . . . . 1<br>NO 2	 →GO BACK
H3	How many men over the age of 18 live in your household?<br><br>RECORD NUMBER. IF OTHER, RECORD 96. IF DK, RECORD 88. IF REFUSED, RECORD 99.	[ ][ ]	
H4	How many women over the age of 18 live in your household?<br><br>RECORD NUMBER. IF OTHER, RECORD 96. IF DK, RECORD 88. IF REFUSED, RECORD 99.	[ ][ ]	
H5	How many male children under the age of 5 live in your household?<br>RECORD NUMBER. IF OTHER, RECORD 96. IF DK, RECORD 88. IF REFUSED, RECORD 99.	[ ][ ]	
H6	How many female children under the age of 5 live in your household?<br>RECORD NUMBER. IF OTHER, RECORD 96. IF DK, RECORD 88. IF REFUSED, RECORD 99.	[ ][ ]	

NO.	QUESTIONS AND FILTERS	CODING CATEGORIES	SKIP
H5	How many male children from the ages of 6-17 live in your household?<br>RECORD NUMBER. IF OTHER, RECORD 96. IF DK, RECORD 88. IF REFUSED, RECORD 99.	[ ][ ]	
H6	How many female children from the ages of 6-17 live in your household?<br>RECORD NUMBER. IF OTHER, RECORD 96. IF DK, RECORD 88. IF REFUSED, RECORD 99.	[ ][ ]	

HOUSEHOLD SCHEDULE

							IF AGE 15 OR OLDER			
LINE NO.	USUAL RESIDENTS AND VISITORS	RELATIONSHIP TO HEAD OF HOUSEHOLD	SEX	RESIDENCE		AGE	MARITAL STATUS	ELIGIBILITY		
**1**	**2**	**3**	**4**	**5**	**6**	**7**	**8**	**9**	**10**	**11**
	Please give me the names of the persons who usually live in your household and guests of the household who stayed here last night, starting with the head of the household.<br><br>AFTER LISTING THE NAMES AND RECORDING THE RELATIONSHIP AND SEX FOR EACH PERSON, ASK QUESTIONS 2A-2C TO BE SURE THAT THE LISTING IS COMPLETE.<br><br>THEN ASK APPROPRIATE QUESTIONS IN COLUMNS 5-20 FOR EACH PERSON.	What is the relationship of (NAME) to the head of the household?<br><br>SEE CODES BELOW.	Is (NAME) male or female?	Does (NAME) usually live here?	Did (NAME) stay here last night?	How old was (NAME) at his/her last birthday?<br><br>IF 95 OR MORE, RECORD '95'.	What is (NAME)'s current marital status?<br><br>1 = MARRIED OR LIVING TOGETHER<br>2 = DIVORCED/ SEPARATED<br>3 = WIDOWED<br>4 = NEVER-MARRIED AND NEVER LIVED TOGETHER<br>↓<br>IF 2,3, OR 4, THE WOMAN IS INELIGIBLE. EXIT THE SURVEY.	CIRCLE LINE NUMBER OF THE ELIGIBLE WOMAN	CIRCLE LINE NUMBER OF THE RESP-ONDENT	CIRCLE LINE NUMBER OF ALL THE ELIGIBLE WOMAN'S CHILDREN BETWEEN AGE 0-5
			M F	Y N	Y N	IN YEARS				
HHH			1 2	1 2	1 2			HHH	HHH	HHH
EW			1 2	1 2	1 2			EW	EW	EW
HUSB			1 2	1 2	1 2		(NOT ASKED)	HUSB	HUSB	HUSB

TICK HERE IF CONTINUATION SHEET USED ☐

2A) Just to make sure that I have a complete listing: are there any other persons such as small children or infants that we have not listed? YES ☐ → ADD TO TABLE NO ☐

2B) Are there any other people who may not be members of your family, such as domestic servants, lodgers, or friends who usually live here? YES ☐ → ADD TO TABLE NO ☐

2C) Are there any guests or temporary visitors staying here, or anyone else who stayed here last night, who have not been listed? YES ☐ → ADD TO TABLE NO ☐

CODES FOR Q. 3: RELATIONSHIP TO HEAD OF HOUSEHOLD

01 = HEAD
02 = WIFE OR HUSBAND
03 = SON OR DAUGHTER
04 = SON-IN-LAW OR DAUGHTER-IN-LAW
05 = GRANDCHILD
06 = PARENT
07 = PARENT-IN-LAW
08 = BROTHER OR SISTER
09 = OTHER RELATIVE
10 = ADOPTED/FOSTER/ STEPCHILD
11 = NOT RELATED
98 = DON'T KNOW

	IF AGE 5 YEARS OR OLDER		IF AGE 5-24 YEARS		IF AGE 0-4 YEARS
LINE NO.	EVER ATTENDED SCHOOL		CURRENT/RECENT SCHOOL ATTENDANCE		BIRTH REGIS-TRATION
	**16**	**17**	**18**	**19**	**20**
	Has (NAME) ever attended school?	What is the highest level of school (NAME) has attended?<br><br>SEE CODES BELOW.<br><br>What is the highest grade (NAME) completed at that level?<br><br>SEE CODES BELOW.	Did (NAME) attend school at any time during the (2016-2017) **(2)** school year?	During this/that school year, what level and grade [is/was] (NAME) attending?<br><br>SEE CODES BELOW.	Does (NAME) have a birth certificate?<br><br>IF NO, PROBE: Has (NAME)'s birth ever been registered with the civil authority?<br><br>1 = HAS CERTIFICATE<br>2 = REGISTERED<br>3 = NEITHER<br>8 = DON'T KNOW
HHH	Y N<br>1 2<br>↓<br>NEXT LINE	LEVEL GRADE<br>☐ ☐☐	Y N<br>1 2<br>↓<br>NEXT LINE	LEVEL GRADE<br>☐ ☐☐	☐
EW	1 2<br>↓<br>NEXT LINE	☐ ☐☐	1 2<br>↓<br>NEXT LINE	☐ ☐☐	☐
HUSB	1 2<br>↓<br>NEXT LINE	☐ ☐☐	1 2<br>↓<br>NEXT LINE	☐ ☐☐	☐

CODES FOR Qs. 17 AND 19: EDUCATION

**LEVEL**	**GRADE**
1 = PRIMARY	00 = LESS THAN 1 YEAR COMPLETED
2 = SECONDARY	(USE '00' FOR Q. 17 ONLY.
3 = HIGHER	THIS CODE IS NOT ALLOWED
6 = PRE-PRIMARY	FOR Q. 19)
8 = DON'T KNOW	98 = DON'T KNOW

HOUSEHOLD CHARACTERISTICS

NO.	QUESTIONS AND FILTERS	CODING CATEGORIES	SKIP
102	What is the main source of drinking water for members of your household?	PIPED WATER<br>PIPED INTO DWELLING . . . . 11<br>PIPED TO YARD/PLOT . . . . 12<br>PUBLIC TAP/STANDPIPE . . . . 13<br>TUBE WELL OR BOREHOLE . . . . 21<br>DUG WELL<br>PROTECTED WELL . . . . 31<br>UNPROTECTED WELL . . . . 32<br>WATER FROM SPRING<br>PROTECTED SPRING . . . . 41<br>UNPROTECTED SPRING . . . . 42<br>RAINWATER . . . . 51<br>TANKER TRUCK . . . . 61<br>CART WITH SMALL TANK . . . . 71<br>SURFACE WATER (RIVER/DAM/<br>LAKE/POND/STREAM/CANAL/<br>IRRIGATION CHANNEL) . . . . 81<br>BOTTLED WATER . . . . 91<br><br>OTHER ______________ 96<br>(SPECIFY)	
107	What kind of toilet facility do members of your household usually use? **(3)**	FLUSH OR POUR FLUSH TOILET<br>FLUSH TO PIPED SEWER<br>SYSTEM . . . . 11<br>FLUSH TO SEPTIC TANK . . . . 12<br>FLUSH TO PIT LATRINE . . . . 13<br>FLUSH TO SOMEWHERE ELSE . . . . 14<br>FLUSH, DON'T KNOW WHERE . . . . 15<br>PIT LATRINE<br>VENTILATED IMPROVED<br>PIT LATRINE . . . . 21<br>PIT LATRINE WITH SLAB . . . . 22<br>PIT LATRINE WITHOUT SLAB/<br>OPEN PIT . . . . 23<br>COMPOSTING TOILET . . . . 31<br>BUCKET TOILET . . . . 41<br>HANGING TOILET/HANGING<br>LATRINE . . . . 51<br>NO FACILITY/BUSH/FIELD . . . . 61<br><br>OTHER ______________ 96<br>(SPECIFY)	 <br><br><br><br><br><br><br><br><br><br><br><br><br><br><br><br>→ 110
108	Do you share this toilet facility with other households?	YES . . . . 1<br>NO 2	 → 110
109	How many households use this toilet facility?	NO. OF HOUSEHOLDS<br>IF LESS THAN 10 . . . . [0][ ]<br><br>10 OR MORE HOUSEHOLDS . . . . 95<br>DON'T KNOW 98	

NO.	QUESTIONS AND FILTERS	CODING CATEGORIES	SKIP
110	Does your household have: **(4)**<br><br>Electricity?<br>Koloboyi?<br>A paraffin lamp other than a koloboyi?<br>A radio?<br>A television?<br>A cellular phone?<br>A telephone (landline)?<br>A bed with mattress?<br>A sofa set?<br>A refrigerator?<br>A dinner table?<br>Chairs?<br>A cabinet/cupboard?<br>A stove?<br>A washing machine?<br>A fan/air conditioner?<br>A generator?<br>A computer?<br>A VCR?<br>A CD/cassette player?<br>A camera?<br><br>[ADD ADDITIONAL ITEMS. SEE FOOTNOTE 4.]	YES NO<br>ELECTRICITY . . . . 1 2<br>KOLOBOYI . . . . 1 2<br>PARAFFIN LAMP . . . . 1 2<br>RADIO . . . . 1 2<br>TELEVISION . . . . 1 2<br>CELL PHONE . . . . 1 2<br>TELEPHONE (LANDLINE) . . . 1 2<br>BED WITH MATTRESS . . . . . 1 2<br>SOFA SET . . . . 1 2<br>REFRIGERATOR . . . . 1 2<br>DINNER TABLE . . . . 1 2<br>CHAIRS . . . . 1 2<br>CABINET/CUPBOARD . . . . 1 2<br>STOVE . . . . 1 2<br>WASHING MACHINE . . . . 1 2<br>FAN/AIR CONDITIONER . . . . . 1 2<br>GENERATOR . . . . 1 2<br>COMPUTER . . . . 1 2<br>VCR . . . . 1 2<br>CD/CASSETTE PLAYER . . . . . 1 2<br>CAMERA 1 2	
111	What type of fuel does your household mainly use for cooking?	ELECTRICITY . . . . 01<br>LPG . . . . 02<br>NATURAL GAS . . . . 03<br>BIOGAS . . . . 04<br>KEROSENE . . . . 05<br>COAL, LIGNITE . . . . 06<br>CHARCOAL . . . . 07<br>WOOD . . . . 08<br>STRAW/SHRUBS/GRASS . . . . 09<br>AGRICULTURAL CROP . . . . 10<br>ANIMAL DUNG . . . . 11<br><br>NO FOOD COOKED<br>IN HOUSEHOLD . . . . 95<br><br>OTHER ______________________ 96<br>(SPECIFY)	 <br><br><br><br><br><br><br><br><br><br><br><br>→ 114
112	Is the cooking usually done in the house, in a separate building, or outdoors?	IN THE HOUSE . . . . 1<br>IN A SEPARATE BUILDING . . . . 2<br>OUTDOORS . . . . 3<br><br>OTHER ______________________ 6<br>(SPECIFY)	2, 3, 6 → 114
113	Do you have a separate room which is used as a kitchen?	YES . . . . 1<br>NO 2	

NO.	QUESTIONS AND FILTERS	CODING CATEGORIES	SKIP
114	MAIN MATERIAL OF THE FLOOR. **(3)**<br><br>RECORD OBSERVATION.	NATURAL FLOOR<br>EARTH/SAND . . . . 11<br>DUNG . . . . 12<br>RUDIMENTARY FLOOR<br>WOOD PLANKS . . . . 21<br>PALM/BAMBOO . . . . 22<br>FINISHED FLOOR<br>PARQUET OR POLISHED<br>WOOD . . . . 31<br>VINYL OR ASPHALT STRIPS . . . . 32<br>CERAMIC TILES . . . . 33<br>CEMENT . . . . 34<br>CARPET . . . . 35<br><br>OTHER ______________ 96<br>(SPECIFY)	
115	MAIN MATERIAL OF THE ROOF. **(3)**<br><br>RECORD OBSERVATION.	NATURAL ROOFING<br>NO ROOF . . . . 11<br>THATCH/PALM LEAF . . . . 12<br>SOD . . . . 13<br>RUDIMENTARY ROOFING<br>RUSTIC MAT . . . . 21<br>PALM/BAMBOO . . . . 22<br>WOOD PLANKS . . . . 23<br>CARDBOARD . . . . 24<br>FINISHED ROOFING<br>METAL . . . . 31<br>LOCAL TILES . . . . 32<br>WOOD . . . . 33<br>CALAMINE/CEMENT FIBER . . . . 34<br>CERAMIC TILES . . . . 35<br>CEMENT . . . . 36<br>ROOFING SHINGLES . . . . 37<br><br>OTHER ______________ 96<br>(SPECIFY)	
116	MAIN MATERIAL OF THE EXTERIOR WALLS. **(3)**<br><br>RECORD OBSERVATION.	NATURAL WALLS<br>NO WALLS . . . . 11<br>CANE/PALM/TRUNKS . . . . 12<br>DIRT . . . . 13<br>RUDIMENTARY WALLS<br>BAMBOO WITH MUD . . . . 21<br>STONE WITH MUD . . . . 22<br>UNCOVERED ADOBE . . . . 23<br>PLYWOOD . . . . 24<br>CARDBOARD . . . . 25<br>REUSED WOOD . . . . 26<br>FINISHED WALLS<br>CEMENT . . . . 31<br>STONE WITH LIME/CEMENT . . . . 32<br>BRICKS . . . . 33<br>CEMENT BLOCKS . . . . 34<br>COVERED ADOBE . . . . 35<br>WOOD PLANKS/SHINGLES . . . . 36<br><br>OTHER ______________ 96<br>(SPECIFY)	

NO.	QUESTIONS AND FILTERS	CODING CATEGORIES	SKIP
117	How many rooms in this household are used for sleeping?	ROOMS . . . . [ ][ ]	
118	Does any member of this household own:<br><br>A watch?<br>A bicycle?<br>A motorcycle or motor scooter?<br>An animal-drawn cart (oxcart)?<br>A car or truck?<br>A boat with a motor?	YES NO<br>WATCH . . . . 1 2<br>BICYCLE . . . . 1 2<br>MOTORCYCLE/SCOOTER . . . 1 2<br>ANIMAL-DRAWN CART . . . . . 1 2<br>CAR/TRUCK . . . . 1 2<br>BOAT WITH MOTOR 1 2	
119	Does any member of this household own any agricultural land?	YES . . . . 1<br>NO 2	 → W101
120	How much agricultural land do members of this household own?<br><br>RECORD IN UNITS RESPONDENT USES.	ACRES . . . . 1 [ ][ ].[ ]<br>HECTARES . . . . 2 [ ][ ].[ ]<br>FOOTBALL PITCHES . . . 3 [ ][ ].[ ]<br>95 OR MORE ACRES/HECTARES/FOOTBALL PITCHES . . . . 9995<br>DON'T KNOW 9998	

CODES FOR 106: HIGHEST EDUCATION LEVEL ATTAINED

Description	Code
Some schooling but not Completed Standard 1\	10
Completed Standard 1	11
Completed Standard 2	12
Completed Standard 3	13
Completed Standard 4	14
Completed Standard 5	15
Completed Standard 6	16
Completed Standard 7	17
Completed Standard 8	18
Completed Form 1	21
Completed Form 2	22
Completed Form 3	23
Completed Form 4	24
Completed Adult Education / Vocational Training	31
Completed University Year 1	41
Completed University Year 2	42
Completed University Year 3 or Higher	43
Don't Know	88
Refused	99

FOOTNOTES

(1) This section should be adapted for country-specific survey design.
(2) In Q. 18, the year should refer to the school year that is in session at the time the survey begins. If the su begins between two school years, then the year should refer to the school year that just ended.
(3) Coding categories to be developed locally and revised based on the pretest; however, the broad categori must be maintained.
(4) Each country should add to the list at least five items of furniture (such as a table, a chair, a sofa, a bed, a armoire, or a cupboard or cabinet). In addition, each country should add at least four additional househol appliances so that the list includes at least three items that even a poor household may have, at least thr items that a middle income household may have, and at least three items that a high income household r have. Some possible additions are clock, water pump, grain grinder, fan, blender, water heater, generator, washing machine, microwave oven, computer, VCR or DVD player, cassette or CD player, car air conditioner or cooler, color TV, sewing machine.
(5) Add other country-specific animals, such as oxen, water buffalo, camels, llamas, alpacas, pigs, ducks, ge or elephants.
(6) The question should be deleted in countries that do not have an organized spraying program to prevent tl transmission of malaria.
(7) The question should be deleted in countries that are not affected by malaria.
(8) There are many different kinds of iodine testing kits available. The proper test kit should be selected in ea country depending on the type of iodine additive used in the country (potassium iodate or potassium iodid If both of these additives are used in a country, then both types of test kits should be used.
(9) Year of fieldwork is assumed to be 2010. For fieldwork beginning in 2011 or 2012, the year should be 2006 or 2007, respectively.
(10) In countries where the weighing scale shows the weight to only one decimal place, retain only one box aft the decimal point and delete the first '9' from the other three codes.
(11) In countries where some enumeration areas are higher than 1,000 meters, altitude information should be collected on a separate form for each enumeration area higher than 1,000 meters so that the anemia est can be adjusted appropriately.
(12) Questions should be omitted in countries in which HIV testing is not a component of the survey.

SECTION 1. RESPONDENT'S BACKGROUND

INTRODUCTION

In this section, I will ask you questions about your health and well-being. The questions in this section usually take about 60 minutes. All of the answers you give will be confidential and will not be shared with anyone other than members of our survey team. You don't have to be in the survey, but we hope you will agree to answer the questions since your views are important. If I ask you any question you don't want to answer, just let me know and I will go on to the next question or you can stop the interview at any time.

In case you need more information about the survey, you may contact the person listed on the card that has already been given to your household.
Do you have any questions? May I begin the interview now?

NO.	QUESTIONS AND FILTERS	CODING CATEGORIES	SKIP
101	RECORD THE TIME.	HOUR . . . . [ ][ ]<br>MINUTES . . . . [ ][ ]	
102_1	Do you know your date of birth?	YES . . . . 1<br>NO 2	 → 102
102_2	What is your date of birth?	DATE OF BIRTH: ____________	
102	In what month and year were you born?	MONTH . . . . [ ][ ]<br>DON'T KNOW MONTH . . . . 98<br>YEAR . . . . [ ][ ][ ][ ]<br>DON'T KNOW YEAR 9998	
103	How old were you at your last birthday?<br><br>COMPARE AND CORRECT 102 AND/OR 103 IF INCONSISTENT.	AGE IN COMPLETED YEARS [ ][ ]	
104	Have you ever attended school?	YES . . . . 1<br>NO 2	 → 108
105	What is the highest level of school you attended: primary, secondary, or higher? (1)	PRE-PRIMARY 0<br>PRIMARY . . . . 1<br>SECONDARY . . . . 2<br>HIGHER . . . . 3<br>DON'T KNOW 3	
106	What is the highest (class/form/year) you completed at that level? (1)<br><br>IF COMPLETED LESS THAN ONE YEAR AT THAT LEVEL, RECORD '00'.	CLASS/FORM/YEAR . . . . [ ][ ]	

NO.	QUESTIONS AND FILTERS	CODING CATEGORIES	SKIP
107	CHECK 105 (SCHOOL CODE):<br>PRIMARY ☐ ↓ SECONDARY OR HIGHER ☐ →		113
108	Now I would like you to read this sentence to me.<br><br>SHOW CARD TO RESPONDENT. **(2)**<br>IF RESPONDENT SPEAKS MULTIPLE LANGUAGES, SELECT ALL THAT APPLIES.<br><br>IF RESPONDENT CANNOT READ WHOLE SENTENCE, PROBE:<br>Can you read any part of the sentence to me?	**ENGLISH**<br>CANNOT READ AT ALL . . . . 1<br>ABLE TO READ ONLY PARTS OF SENTENCE . . . . 2<br>ABLE TO READ WHOLE SENTENCE 3<br><br>**CHICHEWA**<br>CANNOT READ AT ALL . . . . 4<br>ABLE TO READ ONLY PARTS OF SENTENCE . . . . 5<br>ABLE TO READ WHOLE SENTENCE 6<br><br>**TUMBUKA**<br>CANNOT READ AT ALL . . . . 7<br>ABLE TO READ ONLY PARTS OF SENTENCE . . . . 8<br>ABLE TO READ WHOLE SENTENCE 9<br><br>NO CARD WITH REQUIRED LANGUAGE__________________ 21<br>(SPECIFY LANGUAGE)<br>BLIND/VISUALLY IMPAIRED 31	
109	Have you ever participated in a literacy program or any other program that involves learning to read or write (not including primary school)?	YES . . . . 1<br>NO 2	
113	What is your religion?	CATHOLIC . . . . 1<br>CCAP . . . . 2<br>ANGLICAN . . . . 3<br>SEVENTH DAY ADVENTIST / BAPTIST 4<br>OTHER CHRISTIAN . . . . 5<br>MUSLIM . . . . 6<br>NO RELIGION . . . . 7<br>OTHER________________________ 96<br>(SPECIFY)	
114	What is your tribe or ethnic group?	CHEWA . . . . 1<br>TUMBUKA . . . . 2<br>LOMWE . . . . 3<br>TONGA . . . . 4<br>YAO . . . . 5<br>SENA . . . . 6<br>NKHONDE . . . . 7<br>NGONI . . . . 8<br>OTHER________________________ 96<br>(SPECIFY)	

NO.	QUESTIONS AND FILTERS	CODING CATEGORIES	SKIP
115	As you know, some women take up jobs for which they are paid in cash or kind. Others sell things, have a small business or work on the family farm or in the family business. In the last seven days, have you done any of these things or any other work?	YES . . . . 1<br>NO . . . . 2<br>DON'T KNOW . . . . #	1 → 118
116	Although you did not work in the last seven days, do you have any job or business from which you were absent for leave, illness, vacation, maternity leave or any other such reason?	YES . . . . 1<br>NO . . . . 2<br>DON'T KNOW . . . . #	1 → 118
117	Have you done any work in the last 12 months?	YES . . . . 1<br>NO . . . . 2<br>DON'T KNOW 8	
118	Do you do this work for a member of your family, for someone else, or are you self-employed?	FOR FAMILY MEMBER . . . . 1<br>FOR SOMEONE ELSE . . . . 2<br>SELF-EMPLOYED 3	
119	Are you paid in cash or kind for this work, or are you not paid at all?	CASH ONLY . . . . 1<br>CASH AND KIND . . . . 2<br>IN KIND ONLY . . . . 3<br>NOT PAID 4	

(1) Revise according to the local education system.
(2) Each card should have four simple sentences appropriate to the country (e.g., "Parents love their children "Farming is hard work.", "The child is reading a book.", "Children work hard at school."). Cards should be prepared for every language in which respondents are likely to be literate.
(3) The question may be considered for deletion in countries with a very low HIV prevalence.

SECTION 2. REPRODUCTION

NO.	QUESTIONS AND FILTERS	CODING CATEGORIES	SKIP
201	Now I would like to ask about all the births you have had during your life. Have you ever given birth?	YES . . . . 1<br>NO 2	 → 209
202	Do you have any sons or daughters to whom you have given birth who are now living with you?	YES . . . . 1<br>NO 2	 → 204
203	How many sons live with you?<br><br>And how many daughters live with you?<br><br>IF NONE, RECORD '00'.	SONS AT HOME . . . . [ ][ ]<br><br>DAUGHTERS AT HOME . . . . . [ ][ ]	
204	Do you have any sons or daughters to whom you have given birth who are alive but do not live with you?	YES . . . . 1<br>NO 2	 → 206
205	How many sons are alive but do not live with you?<br><br>And how many daughters are alive but do not live with you?<br><br>IF NONE, RECORD '00'.	SONS ELSEWHERE . . . . [ ][ ]<br><br>DAUGHTERS ELSEWHERE [ ][ ]	
205_1	SUM ANSWERS TO 203, 205, AND ENTER TOTAL LIVE SONS.<br>IF NONE, RECORD '00'.	TOTAL LIVE SONS . . . . [ ][ ]	
205_2	SUM ANSWERS TO 203, 205, AND ENTER TOTAL LIVE DAUGHTERS. IF NONE, RECORD '00'.	TOTAL LIVE DAUGHTERS [ ][ ]	
205_3	SUM ANSWERS TO 206, 207, AND ENTER TOTAL LIVE BIRTHS.<br>IF NONE, RECORD '00'.	TOTAL LIVE BIRTHS . . . . [ ][ ]	
206	Have you ever given birth to a boy or girl who was born alive but later died?<br><br>IF NO, PROBE: Any baby who cried or showed signs of life but did not survive?	YES . . . . 1<br>NO 2	 → 211
207_M<br><br>207_F	How many boys have died?<br><br>And how many girls have died?<br><br>IF NONE, RECORD '00'.	BOYS DEAD . . . . [ ][ ]<br><br>GIRLS DEAD . . . . [ ][ ]	
208	SUM ANSWERS TO 203, 205, AND 207, AND ENTER TOTAL.<br>IF NONE, RECORD '00'.	TOTAL BIRTHS . . . . [ ][ ]	→ If 00, END
209	CHECK 208:<br><br>Just to make sure that I have this right: you have had in TOTAL _____ births during your life. Is that correct?<br><br>YES [ ] ↓ NO [ ] → PROBE AND CORRECT 201-208 AS NECESSARY.		
210	CHECK 208:<br><br>ONE OR MORE BIRTHS [ ] ↓ NO BIRTHS [ ] →		226

211	Now I would like to record the name of your last birth, whether still alive or not<br>RECORD THE NAME OF YOUR LAST BIRTH IN 212. RECORD TWINS AND TRIPLETS ON SEPARATE ROWS.

212<br><br>What name was given to your last baby?<br><br>RECORD NAME.<br><br>BIRTH HISTORY NUMBER	213<br><br>Is (NAME) a boy or a girl?	214<br><br>Was this birth a twin?	215<br><br>In what month and year was (NAME) born?<br><br>PROBE:<br>When is his/her birthday?	216<br><br>Is (NAME) still alive?	217<br>IF ALIVE:<br><br>How old was (NAME) at his/her last birthday?<br><br>RECORD AGE IN COM-PLETED YEARS.	218<br>IF ALIVE:<br><br>Is (NAME) living with you?<br><br>Is (NAME) here in this house at thi time?	220<br>IF DEAD:<br><br>How old was (NAME) when he/she died?<br><br>IF '1 YR', PROBE:<br>How many months old was (NAME)?<br>RECORD DAYS IF LESS THAN 1 MONTH; MONTHS IF LESS THAN TWO YEARS; OR YEARS.	
01	BOY 1<br><br>GIRL 2	SING 1<br><br>MULT 2	MONTH<br><br>YEAR	YES . . 1<br><br>NO . . . 2<br>↓<br>220	AGE IN YEARS	YES . . . 1<br><br>NO 2	DAYS . . . 1<br><br>MONTHS 2<br><br>YEARS . . 3	

221	Have you had any live births since the birth of (NAME OF LAST BIRTH)? IF YES, RECORD BIRTH(S) IN TABLE.	YES . . . . 1<br>NO 2

NO.	QUESTIONS AND FILTERS	CODING CATEGORIES	SKIP
225	**C** FOR EACH BIRTH SINCE APRIL 2018 **(1)**, ENTER 'B' IN THE MONTH OF BIRTH IN THE CALENDAR. WRITE THE NAME OF THE CHILD TO THE LEFT OF THE 'B' CODE. FOR EACH BIRTH, ASK THE NUMBER OF MONTHS THE PREGNANCY LASTED AND RECORD 'P' IN EACH OF THE PRECEDING MONTHS ACCORDING TO THE DURATION OF PREGNANCY. (NOTE: THE NUMBER OF 'P's MUST BE ONE LESS THAN THE NUMBER OF MONTHS THAT THE PREGNANCY LASTED.)		
226	Are you pregnant now?	YES . . . . 1<br>NO . . . . 2<br>UNSURE 8	1 → END<br>2, 8 → 230
230	Have you ever had a pregnancy that was miscarried, was aborted, or ended in a stillbirth?	YES . . . . 1<br>NO 2	2 → 302
231	In which year did the last such pregnancy end?	MONTH . . . . [ ][ ]<br>YEAR . . . . [ ][ ][ ][ ]	

(1) Year of fieldwork is assumed to be 2010. For fieldwork beginning in 2011 or 2012, the year should be 200 or 2007, respectively.

SECTION 3. CONTRACEPTION

NO.	QUESTIONS AND FILTERS	CODING CATEGORIES	SKIP
301	Now I would like to talk about family planning - the various ways or methods that a couple can use to delay or avoid a pregnancy.<br><br>Which ways or methods of family planning have you heard about?<br>FOR METHODS NOT MENTIONED SPONTANEOUSLY, ASK:<br>Have you ever heard of (METHOD)?<br><br>CIRCLE CODE 1 IN 301 FOR EACH METHOD MENTIONED SPONTANEOUSLY. THEN PROCEED DOWN COLUMN 301, READING THE NAME AND DESCRIPTION OF EACH METHOD NOT MENTIONED SPONTANEOUSLY. CIRCLE CODE 1 IF METHOD IS RECOGNIZED, AND CODE 2 IF NOT RECOGNIZED. THEN, FOR EACH METHOD WITH CODE 1 CIRCLED IN 301, ASK 302.		
01	FEMALE STERILIZATION Women can have an operation to avoid having any more children.	YES . . . . 1<br>NO 2	↓
02	MALE STERILIZATION Men can have an operation to avoid having any more children.	YES . . . . 1<br>NO 2	↓
03	PILL Women can take a pill every day to avoid becoming pregnant.	YES . . . . 1<br>NO 2	↓
04	IUD Women can have a loop or coil placed inside them by a doctor or a nurse.	YES . . . . 1<br>NO 2	↓
05	INJECTABLES Women can have an injection by a health provider that stops them from becoming pregnant for one or more months.	YES . . . . 1<br>NO 2	↓
06	IMPLANTS Women can have two or more small rods placed in their upper arm by a doctor or nurse which can prevent pregnancy for one or more years.	YES . . . . 1<br>NO 2	↓
07	MALE CONDOM Men can put a rubber sheath on their penis before sexual intercourse.	YES . . . . 1<br>NO 2	↓
08	FEMALE CONDOM Women can place a sheath in their vagina before sexual intercourse.	YES . . . . 1<br>NO 2	↓
09	RHYTHM OR PERIODIC ABSTINENCE Every month that a woman is sexually active she can avoid pregnancy by not having sexual intercourse on the days of the month she is most likely to get pregnant.	YES . . . . 1<br>NO 2	↓
10	WITHDRAWAL Men can be careful and pull out before climax.	YES . . . . 1<br>NO 2	↓
11	EMERGENCY CONTRACEPTION As an emergency measure after unprotected sexual intercourse, women can take special pills at any time within five days to prevent pregnancy.	YES . . . . 1<br>NO 2	↓
12	STANDARD DAYS METHOD<br>For a woman with regular menstrual cycles, they can identify the window when she is likely to be pregnant using beads (necklace)	YES . . . . 1<br>NO 2	↓
12	Have you heard of any other ways or methods that women or men can use to avoid pregnancy?	YES . . . . 1<br>______________<br>(SPECIFY)<br>______________<br>(SPECIFY)<br>NO 2	

NO.	QUESTIONS AND FILTERS	CODING CATEGORIES	SKIP
302	CHECK 226:<br>NOT PREGNANT OR UNSURE ☐ ↓ PREGNANT ☐ →		END
303A	Which ways or methods of family planning have you heard about?<br>Have you ever heard of (METHOD)?<br><br>CHOOSE ALL METHODS THAT ARE MENTIONED.	FEMALE STERILIZATIO .... A<br>MALE STERILIZATION .... B<br>IUD .... C<br>INJECTABLES .... D<br>IMPLANTS .... E<br>PILL .... F<br>CONDOM .... G<br>FEMALE CONDOM .... H<br>DIAPHRAGM .... I<br>FOAM/JELLY .... J<br>STANDARD DAYS METHOD .... K<br>LACTATIONAL AMEN. METHOI ...... L<br>TWO DAY METHOI .... M<br>RHYTHM METHOD .... N<br>WITHDRAWAL .... O<br>OTHER MODERN METHOD .... X<br>OTHER TRADITIONAL METHOD .... Y	A → END<br>B → END<br>C, D, E → 308B<br>G → 306<br>H–X → 308A
303	Are you currently doing something or using any method to delay or avoid getting pregnant?	YES .... 1<br>NO 2	2 → 311
304	Which method are you using? **(4)**<br><br>CIRCLE ALL MENTIONED.<br><br>IF MORE THAN ONE METHOD MENTIONED, FOLLOW SKIP INSTRUCTION FOR HIGHEST METHOD IN LIST.	FEMALE STERILIZATIO .... 1<br>MALE STERILIZATION .... 2<br>IUD .... 3<br>INJECTABLES .... 4<br>IMPLANTS .... 5<br>PILL .... 6<br>CONDOM .... 7<br>FEMALE CONDOM .... 8<br>DIAPHRAGM/FOAM/JELLY 9<br>TWO DAY METHOD 10<br>STANDARD DAYS METHOD .... 11<br>LACTATIONAL AMI .... 12<br>RHYTHM METHOD .... 13<br>WITHDRAWAL .... 14<br>OTHER MODERN METHOD 15<br>OTHER TRADITIONAL METH .... 16<br>....	1 → END<br>2 → END<br>3, 4, 5 → 308B<br>7 → 306<br>8–16 → 308A
305	What is the brand name of the pills you are using?<br><br>IF DON'T KNOW THE BRAND,<br>ASK TO SEE THE PACKAGE.	LOFEMINOL 01<br>MICROGYNON 02<br>OVRETTE .... 03<br><br>OTHER ______________ 96<br>(SPECIFY)<br>DON'T KNOW 98	→ 308A
306	What is the brand name of the condoms you are using?<br><br>IF DON'T KNOW THE BRAND,<br>ASK TO SEE THE PACKAGE.	CHISHANGO .... 01<br>MANYUCHI .... 02<br>CARE (FEMALE CONDOM) .... 03<br><br>OTHER ______________ 96<br>(SPECIFY)<br><br>DON'T KNOW 98	→ 308A
308<br><br>308A<br><br><br><br>308B	In what month and year was the sterilization performed?<br><br>Since what month and year have you been using (CURRENT METHOD) without stopping?<br><br>PROBE: For how long have you been using (CURRENT METHOD) now without stopping?<br><br>FOR WOMEN WHO USE INJECTABLES:<br>When was the last time that you received an injectable/IUD/Implants?<br>RECORD NUMBER OF MONTHS SINCE LAST INJECTABLE.	MONTH .... ☐☐<br>YEAR .... ☐☐☐☐<br><br>MONTHS SINCE LAST INJECTABLE<br>☐☐	

NO.	QUESTIONS AND FILTERS	CODING CATEGORIES	SKIP
309	CHECK 308/308A, 215 AND 231:<br><br>ANY BIRTH OR PREGNANCY TERMINATION AFTER MONTH AND YEAR OF START OF USE OF CONTRACEPTION IN 308/308A<br><br>GO BACK TO 308/308A, PROBE AND RECORD MONTH AND YEAR AT START OF CONTINUOUS USE OF CURRENT METHOD (MUST BE AFTER LAST BIRTH OR PREGNANCY TERMINATION).	YES ☐ NO ☐	
310	CHECK 308/308A:<br><br>April 2018 **(6)** OR LATER ☐<br><br>C ENTER CODE FOR METHOD USED IN MONTH OF INTERVIEW IN THE CALENDAR AND IN EACH MONTH BACK TO THE DATE STARTED USING.	March 2018 **(7)** OR EARLIER ☐<br><br>C ENTER CODE FOR METHOD USED IN MONTH OF INTERVIEW IN THE CALENDAR AND EACH MONTH BACK TO APRIL 2018 **(6)**.<br><br>THEN SKIP TO	322
311	I would like to ask you some questions about the times you or your partner may have used a method to avoid getting pregnant during the last year.<br><br>USE CALENDAR TO PROBE FOR EARLIER PERIODS OF USE AND NONUSE, STARTING WITH MOST RECENT USE, BACK TO APRIL 2018. **(6)**<br>RECENT USE, BACK TO APRIL 2018. **(6)**<br><br>C **IN COLUMN 1**, ENTER METHOD USE CODE OR '0' FOR NONUSE IN EACH BLANK MONTH.<br><br>ILLUSTRATIVE QUESTIONS:<br>* When was the last time you used a method? Which method was that?<br>* When did you start using that method? How long after the birth of (NAME)?<br>* How long did you use the method then?<br><br>**IN COLUMN 2**, ENTER CODES FOR DISCONTINUATION NEXT TO THE LAST MONTH OF USE. NUMBER OF CODES IN COLUMN 2 MUST BE SAME AS NUMBER OF INTERRUPTIONS OF METHOD USE IN COLUMN 1.<br><br>ASK WHY SHE STOPPED USING THE METHOD. IF A PREGNANCY FOLLOWED, ASK WHETHER SHE BECAME PREGNANT UNINTENTIONALLY WHILE USING THE METHOD OR DELIBERATELY STOPPED TO GET PREGNANT.<br><br>ILLUSTRATIVE QUESTIONS:<br>* Why did you stop using the (METHOD)? Did you become pregnant while using (METHOD), or did you stop to get pregnant, or did you stop for some other reason?<br>* IF DELIBERATELY STOPPED TO BECOME PREGNANT, ASK: How many months did it take you to get pregnant after you stopped using (METHOD)? AND ENTER ‘0’ IN EACH SUCH MONTH IN COLUMN 1.		

NO.	QUESTIONS AND FILTERS	CODING CATEGORIES	SKIP
312	CHECK THE CALENDAR FOR USE OF ANY CONTRACEPTIVE METHOD IN ANY MONTH<br><br>NO METHOD USED ☐ ↓ ANY METHOD USED ☐		→ 316
313	Have you ever used anything or tried in any way to delay or avoid getting pregnant?	YES . . . . 1<br>NO 2	 → 324A
313B	Which method(s) have you used? **(4)**<br><br>CIRCLE ALL MENTIONED.<br><br>IF MORE THAN ONE METHOD MENTIONED, FOLLOW SKIP INSTRUCTION FOR HIGHEST METHOD IN LIST.	FEMALE STERILIZATIO . . . . 1<br>MALE STERILIZATION . . . . 2<br>IUD . . . . 3<br>INJECTABLES . . . . . . 4<br>IMPLANTS . . . . . . 5<br>PILL . . . . 6<br>CONDOM . . . . 7<br>FEMALE CONDOM . . . . . 8<br>DIAPHRAGM/FOAM/JELLY 9<br>TWO DAY METHOD 10<br>STANDARD DAYS METHOD . . . 11<br>LACTATIONAL AMI . . . . . 12<br>RHYTHM METHOD . . . . . 13<br>WITHDRAWAL . . . . . . 14<br>OTHER MODERN METHOD 15<br>OTHER TRADITIONAL METH . . . 16	→ END<br>→ END<br>(3–16) → 324A
316	CHECK 304:<br><br>CIRCLE METHOD CODE:<br><br>IF MORE THAN ONE METHOD CODE CIRCLED IN 304, CIRCLE CODE FOR HIGHEST METHOD IN LIST.	NO CODE CIRCLED . . . . . 00<br><br>FEMALE STERILIZATIO . . . . 1<br>MALE STERILIZATION . . . . 2<br>IUD . . . . 3<br>INJECTABLES . . . . . . 4<br>IMPLANTS . . . . . . 5<br>PILL . . . . 6<br>CONDOM . . . . 7<br>FEMALE CONDOM . . . . . 8<br>DIAPHRAGM/FOAM/JELLY 9<br>TWO DAY METHOD 10<br>STANDARD DAYS METHOD . . . 11<br>LACTATIONAL AMI . . . . . 12<br>RHYTHM METHOD . . . . . 13<br>WITHDRAWAL . . . . . . 14<br>OTHER MODERN METHOD 15<br>OTHER TRADITIONAL METH . . . 16	→ 324A<br><br>→ END<br>→ END<br><br><br><br><br>→ 323 (7)<br>(8–10) → 320<br>(11–16) → 324A
317	At that time, were you told about side effects or problems you might have with the method?	YES . . . . 1<br>NO 2	→ 319
318	Were you ever told by a health or family planning worker about side effects or problems you might have with the method?	YES . . . . 1<br>NO 2	 → 320
319	Were you told what to do if you experienced side effects or problems?	YES . . . . 1<br>NO 2	

NO.	QUESTIONS AND FILTERS	CODING CATEGORIES	SKIP
320	CHECK 317:<br><br>CODE '1' CIRCLED ↓ — At that time, were you told about other methods of family planning that you could use alternatively?<br><br>CODE '1' NOT CIRCLED ↓ — When you obtained (CURRENT METHOD FROM 314) from (SOURCE OF METHOD FROM 307 OR 315), were you told about other methods of family planning that you could use?	YES . . . . 1<br>NO 2	→ 322
321	Were you ever told by a health or family planning worker about other methods of family planning that you could use?	YES . . . . 1<br>NO 2	
322	CHECK 304:<br><br>CIRCLE METHOD CODE:<br><br>IF MORE THAN ONE METHOD CODE CIRCLED IN 304, CIRCLE CODE FOR HIGHEST METHOD IN LIST.	FEMALE STERILIZATIO . . . . 1<br>MALE STERILIZATION . . . . 2<br>IUD . . . . 3<br>INJECTABLES . . . . 4<br>IMPLANTS . . . . 5<br>PILL . . . . 6<br>CONDOM . . . . 7<br>FEMALE CONDOM . . . . 8<br>DIAPHRAGM/FOAM/JELLY 9<br>TWO DAY METHOD 10<br>STANDARD DAYS METHOD . . . . 11<br>LACTATIONAL AMI . . . . 12<br>RHYTHM METHOD . . . . 13<br>WITHDRAWAL . . . . 14<br>OTHER MODERN METHOD 15<br>OTHER TRADITIONAL METH 16	→ END (1)<br>→ END (2)<br><br>11–16 → 324A

NO.	QUESTIONS AND FILTERS	CODING CATEGORIES	SKIP
323	Where did you obtain (CURRENT METHOD) the last time? **(5)**<br><br>PROBE TO IDENTIFY THE TYPE OF SOURCE.<br><br>NAME OF SOURCE:<br>____________________<br><br>LOCATION OF SOURCE:<br><br>GPS COORDINATES:<br><br>LATITUDE:<br>N/S D D X X . X X X<br>[ ] [ ][ ] [ ][ ] . [ ][ ][ ]<br><br>LONGITUDE:<br>E/W D D X X . X X X<br>[ ] [ ][ ] [ ][ ] . [ ][ ][ ]	GOOD HEALTH KAUMA CLINIC 01<br>**PUBLIC SECTOR**<br>GOVT. HOSPITAL . . . . 11<br>GOVT. HEALTH CENTER . . . . 12<br>GOV'T HEALTH POST/<br>OUTREACH . . . . 13<br>MOBILE CLINIC . . . . 14<br>HSA . . . . 15<br>CBDA / DOOR-TO-DOOR . . . . 16<br>OTHER PUBLIC<br>SECTOR ____________ 17<br>(SPECIFY)<br><br>**CHAM / MISSION**<br>HOSPITAL . . . . 21<br>HEALTH CENTER . . . . 22<br>MOBILE CLINIC . . . . 23<br>DOOR-TO-DOOR . . . . 24<br><br>**PRIVATE MEDICAL SECTOR**<br>PRIVATE HOSPITAL / CLINIC . . . . . . 31<br>PHARMACY . . . . 32<br>PRIVATE DOCTOR . . . . 33<br>MOBILE CLINIC . . . . 34<br>CBDA / DOOR-TO-DOOR . . . . 35<br>OTHER PRIVATE MEDICAL<br>SECTOR ____________ 36<br>(SPECIFY)<br><br>**BANJA LA MTSOGOLO (BLM)** 41<br>**MACRO** 51<br>**TUNZA (PSI) CLINIC** 61<br>**YOUTH DROP IN CENTRE** 71<br><br>**OTHER SOURCE**<br>SHOP . . . . 81<br>CHURCH . . . . 82<br>FRIEND/RELATIVE . . . . 83<br><br>OTHER ____________ 96<br>(SPECIFY)	01–36 → 323A<br><br>41–96 → 323A
323A	How many kilometers did you have to travel to reach this (SERVICE PROVIDER) to receive (CURRENT METHOD)?	KM . . . . [ ][ ]	
323B	How many minutes did it take for you to travel to this (SERVICE PROVIDER) to receive (CURRENT METHOD)?	MINUTES . . . . [ ][ ][ ][ ]	

NO.	QUESTIONS AND FILTERS	CODING CATEGORIES	SKIP
323B_2	What mode(s) of transportation did you use to travel to this (SERVICE PROVIDER) to receive (CURRENT METHOD)?	NONE (RECEIVED AT HOME) 1<br>WALK 2<br>BICYCLE 3<br>MOTORCYCLI . . . . 4<br>BUS . . . . 5<br>CAR / TAXI 6<br>OTHER . . . . 96<br>DON'T KNOW 88<br>REFUSED 99	
323C	How much, in Malawian Kwacha, did you have to pay in transportation costs to go to this (SERVICE PROVIDER)?	MKW . [ ][ ][ ][ ][ ][ ]	
323D	How many minutes did you have to wait at the (SERVICE PROVIDER) before you received (CURRENT METHOD)?	MINUTES . . . . [ ][ ][ ][ ]	
324A	At any time during your current / last pregnancy, were you counselled about family planning / birth spacing?	YES . . . . 1<br>NO . . . . 2<br>DON'T KNOW . . . . 88<br>REFUSED 99	NO / DON'T KNOW / REFUSED → 325A
324B	How many times did you receive information or counseling on family planning / birth spacing during your current / last pregnancy?	[ ][ ] TIMES COUNSELED<br>DON'T KNOW . . . . 88<br>REFUSED 99	

NO.	QUESTIONS AND FILTERS	CODING CATEGORIES	SKIP
324E	Which method(s) were you counseled on?<br><br>CIRCLE ALL MENTIONED.	FEMALE STERILIZATIO . . . . 1<br>MALE STERILIZATION . . . . 2<br>IUD . . . . 3<br>INJECTABLES . . . . 4<br>IMPLANTS . . . . 5<br>PILL . . . . 6<br>CONDOM . . . . 7<br>FEMALE CONDOM . . . . 8<br>DIAPHRAGM/FOAM/JELLY 9<br>TWO DAY METHOD 10<br>STANDARD DAYS METHOD . . . . 11<br>LACTATIONAL AMI . . . . 12<br>RHYTHM METHOD . . . . 13<br>WITHDRAWAL . . . . 14<br>OTHER MODERN METHOD 15<br>OTHER TRADITIONAL METH 16	
324C	Did you receive that counseling in a group with other women, individually with the provider, or both the last time?	INDIVIDUAL COUNSELING ONLY 1<br>GROUP COUNSELING ONLY 2<br>INDIVIDUAL + GROUP COUNSELING 3<br>DON'T KNOW . . . . 88<br>REFUSED 99	
324D	In which language(s) were you counseled?<br><br>SELECT ALL THAT APPLY.	CHICHEWA . . . . 1<br>TUMBUKA . . . . 2<br>ENGLISH . . . . 3<br>OTHER . . . . 96<br>DON'T REMEMBER/DON'T KNOW 88<br>REFUSED 99	
324F	Where did you receive counseling the last time?<br><br>PROBE TO IDENTIFY THE TYPE OF SOURCE.<br><br>NAME OF SOURCE:<br><br>______________________________<br><br>LOCATION OF SOURCE:<br><br>GPS COORDINATES:<br><br>LATITUDE:<br>N/S D D X X . X X X<br>[ ] [ ][ ] [ ][ ] . [ ][ ][ ]<br><br>LONGITUDE:<br>E/W D D X X . X X X<br>[ ] [ ][ ] [ ][ ] . [ ][ ][ ]	HOME 00<br>GOOD HEALTH KAUMA CLINIC 01<br>**PUBLIC SECTOR**<br>GOVT. HOSPITAL . . . . 11<br>GOVT. HEALTH CENTER . . . . 12<br>GOV'T HEALTH POST/<br>OUTREACH . . . . 13<br>MOBILE CLINIC . . . . 14<br>HSA . . . . 15<br>CBDA / DOOR-TO-DOOR . . . . 16<br>OTHER PUBLIC<br>SECTOR ____________ 17<br>(SPECIFY)<br><br>**CHAM / MISSION**<br>HOSPITAL . . . . 21<br>HEALTH CENTER . . . . 22<br>MOBILE CLINIC . . . . 23<br>DOOR-TO-DOOR . . . . 24<br><br>**PRIVATE MEDICAL SECTOR**<br>PRIVATE HOSPITAL / CLINIC . . . . . . 31<br>PHARMACY . . . . 32<br>PRIVATE DOCTOR . . . . 33<br>MOBILE CLINIC . . . . 34<br>CBDA / DOOR-TO-DOOR . . . . 35<br>OTHER PRIVATE MEDICAL<br>SECTOR ____________ 36<br>(SPECIFY)<br><br>**BANJA LA MTSOGOLO (BLM)** 41<br>**MACRO** 51<br>**TUNZA (PSI) CLINIC** 61<br>**YOUTH DROP IN CENTRE** 71<br><br>**OTHER SOURCE**<br>SHOP . . . . 81<br>CHURCH . . . . 82<br>FRIEND/RELATIVE . . . . 83<br><br>OTHER ____________ 96<br>(SPECIFY)	(00–36) → 325A<br><br>(41–96) → 325A
325A	When you received this method, did you feel that you got enough information about [current method(s)]?	YES . . . . 1<br>NO . . . . 2<br>DON'T KNO . . . . 88<br>REFUS 99	

NO.	QUESTIONS AND FILTERS	CODING CATEGORIES	SKIP
325B	Was the method that you wanted available to you?	YES . . . 1<br>NO . . . 2<br>DON'T KNOW . . . 88<br>REFUSED . . . 99	
325C	Do you feel that you had access to a wide range of family planning methods, or only to a select few?	A WIDE RANGE . . . 1<br>A SELECT FEW . . . 2<br>DON'T KNOW . . . 88<br>REFUSED . . . 99	
325D	Did you ever feel pressured into using [current method(s)]	YES . . . 1<br>NO . . . 2<br>DON'T KNOW . . . 88<br>REFUSED . . . 99	
325E	Did you feel you could say no to using [current method(s)] at any time?	YES . . . 1<br>NO . . . 2<br>DON'T KNOW . . . 88<br>REFUSED . . . 99	
326	In the last 12 months, were you visited by a fieldworker who talked to you about family planning? **(8)**	YES . . . 1<br>NO . . . 2<br>DON'T KNOW . . . 88<br>REFUSED . . . 99	
327	In the last 12 months, have you visited a health facility for care for yourself (or your children)?	YES . . . 1<br>NO . . . 2<br>DON'T KNOW . . . 88<br>REFUSED . . . 99	NO → 329
328	Did any staff member at the health facility speak to you about family planning methods?	YES . . . 1<br>NO . . . 2<br>DON'T KNOW . . . 88<br>REFUSED . . . 99	

(1) If Standard Days Method is commonly used, it may be added to the table before Lactational Amenorrhea. "**Standard Days Method** (use local term, such as CycleBeads™ , as appropriate) PROBE: A woman uses a string of colored beads to know the days she can get pregnant. On the days she can get pregnant, she uses a condom or does not have sexual intercourse." If Standard Days Method is added to Q. 301, it should also be added before LAM to Qs. 304, 314, 316, 322, and Column 1 of the calendar.

(2) The LAM method should be deleted in countries that do not have a LAM program. In these countries, LAM should also be deleted as a coding category in Qs. 304, 314, 316, 322, and Column 1 of the calendar. A description of LAM should not be provided in Q. 301.

(3) Studies have indicated emergency contraception can be effective up to five days. Verify country program recommendations and modify wording if appropriate.

(4) Other commonly used methods may be added to the list, such as contraceptive patch, contraceptive vaginal ring, or sponge. Any codes added in Q. 304 must also be added to Qs. 314, 316, 322, and Column 1 of the calendar. These methods should not be added to Q. 301.

(5) Coding categories to be developed locally and revised based on the pretest; however, the broad categories must be maintained.

(6) Year of fieldwork is assumed to be 2010. For fieldwork beginning in 2011 or 2012, the year should be 2006 or 2007, respectively.

(7) Year of fieldwork is assumed to be 2010. For fieldwork beginning in 2011 or 2012, the year should be 2005 or 2006, respectively.

(8) In countries without national fieldworker programs that include family planning, Q. 326 should be deleted.

NO.	QUESTIONS AND FILTERS	CODING CATEGORIES	SKIP
329	CHECK 303:<br><br>CURRENTLY USING ☐ ↓ NOT CURRENTLY USING ☐ →		336
330	What are some of the reasons as to why you chose to use this particular family planning method?<br><br>SELECT ALL THAT APPLY.	EFFECTIVE IT IS AT PREVENTING PREGNANCY 1<br>CAN BE USED WITHOUT ANYONE ELSE KNOWING 2<br>PROTECTS AGAINST STI/HIV . . . . 3<br>DURATION OF EFFECT / LASTS LONG . . . . 4<br>NO RISK OF HARMING HEALTH . . . . 5<br>NO EFFECT ON REGULAR MONTHLY BLEEDING 6<br>NO UNPLEASANT SIDE EFFECTS . . . . 7<br>LOW COST . . . . 9<br>EASILY AVAILABLE AT THE CLNIC . . . . 10<br>CAN BE USED FOR A LONG TIME WITHOUT NEED TO VISIT CLINIC OR RE-SUPPLY . . . . 11<br>WILL BE ABLE TO GET PREGNANT WHEN I WANT 12<br>NO NEED TO GO TO A CLINIC TO OBTAIN THE METHOD 13<br>NO RISK OF INFERTILI[illegible] . . . . 14<br>NO NEED TO REMEMBER USING THE METHOD 15<br>WANT TO TRY SOMETHING NEW / TIRED OF OLD METH 16<br>MY DOCTOR RECOMMENDED IT TO M . . . . 17<br>MY HUSBAND WANTED ME TO USE THIS METHOD 18<br>OTHER WOMEN IN MY FAMILY HAVE USED THIS METH(19<br>FRIENDS HAVE USED THIS METHOD 20<br>DOES NOT INTERRUPT SEX 21<br>OTHER ______________ 96<br>(SPECIFY)<br>DON'T KNOW . . . . 88<br>REFUSED 99	
331	If you had the choice and ability to switch to another family planning method, would you choose to switch?	YES . . . . 1<br>NO 2	 → 336
332	Which method(s) would you want to switch to?<br><br>CIRCLE ALL MENTIONED.	FEMALE STERILIZATION . . . . 1<br>MALE STERILIZATION . . . . 2<br>IUD . . . . 3<br>INJECTABLES . . . . 4<br>IMPLANTS . . . . 5<br>PILL . . . . 6<br>CONDOM . . . . 7<br>FEMALE CONDOM . . . . 8<br>DIAPHRAGM/FOAM/JELLY 9<br>TWO DAY METHOD 10<br>STANDARD DAYS METHOD . . . . 11<br>LACTATIONAL AMI . . . . 12<br>RHYTHM METHOD . . . . 13<br>WITHDRAWAL . . . . 14<br>OTHER MODERN METHOD 15<br>OTHER TRADITIONAL METH(. 16	

NO.	QUESTIONS AND FILTERS	CODING CATEGORIES	SKIP
333	Why would you want to switch to this method?<br><br>SELECT ALL THAT APPLY.	EFFECTIVE IT IS AT PREVENTING PREGNANCY 1<br>CAN BE USED WITHOUT ANYONE ELSE KNOWING 2<br>PROTECTS AGAINST STI/HIV . . . . 3<br>DURATION OF EFFECT / LASTS LONG . . . . 4<br>NO RISK OF HARMING HEALTH . . . . 5<br>NO EFFECT ON REGULAR MONTHLY BLEEDING 6<br>NO UNPLEASANT SIDE EFFECTS . . . . 7<br>LOW COST . . . . 9<br>EASILY AVAILABLE AT THE CLNIC . . . . 10<br>CAN BE USED FOR A LONG TIME WITHOUT NEED TO VISIT CLINIC OR RE-SUPPLY . . . . 11<br>WILL BE ABLE TO GET PREGNANT WHEN I WANT 12<br>NO NEED TO GO TO A CLINIC TO OBTAIN THE METHOD 13<br>NO RISK OF INFERTILI[illegible] . . . . 14<br>NO NEED TO REMEMBER USING THE METHOD 15<br>WANT TO TRY SOMETHING NEW / TIRED OF OLD METH 16<br>MY DOCTOR RECOMMENDED IT TO M . . . . 17<br>MY HUSBAND WANTED ME TO USE THIS METHOD 18<br>OTHER WOMEN IN MY FAMILY HAVE USED THIS METH[illegible] 19<br>FRIENDS HAVE USED THIS METHOD 20<br>DOES NOT INTERRUPT SEX 21<br>OTHER ______________________ 96<br>(SPECIFY)<br>DON'T KNOW . . . . 88<br>REFUSED 99	
334	Why have you not yet switched to this method?	FERTILITY-RELATED REASONS<br>NOT HAVING SEX . . . . 2<br>INFREQUENT SEX . . . . 3<br>MENOPAUSAL/HYSTERECTOMY 4<br>CAN'T GET PREGNANT . . . . 5<br>NOT MENSTRUATED SINCE LAST BIRTH . . . . 6<br>BREASTFEEDING . . . . 7<br>UP TO GOD/FATALISTIC . . . . 8<br><br>OPPOSITION TO USE<br>RESPONDENT OPPOSED . . . . 9<br>HUSBAND/PARTNER OPPOSE[illegible] . . . . 10<br>OTHERS OPPOSED . . . . 11<br>RELIGIOUS PROHIBITION . . . . 12<br>SOCIAL PRESSURE . . . . 13<br><br>LACK OF KNOWLEDGE<br>KNOWS NO METHOD . . . . 14<br>KNOWS NO SOURCE . . . . 15<br><br>METHOD-RELATED REASONS<br>FEAR OF INFERTILITY . . . . 16<br>FEAR OF SIDE EFFECTS . . . . 17<br>INTERFERES WITH BODY'S NORMAL PROCESSES . . . . 18<br>OTHER HEALTH CONCER[illegible] . . . . 19<br>LACK OF ACCESS/TOO FAR . . . . . . 20<br>LONG WAITING TIME AT . . . . 21<br>TOO BUSY/NO TIME . . . . 22<br>COSTS TOO MUCH . . . . 23<br>PREFERRED METHOD NOT AVAILABLE . . . . 24<br>NO METHOD AVAILABLE . . . . 25<br>INCONVENIENT TO USE . . . . 26<br>NOT EFFECTIVE . . . . 27<br>OTHER ______________________ 96<br>(SPECIFY)<br>DON'T KNOW 88	FOR ALL OPTION S SKIP TO 331

NO.	QUESTIONS AND FILTERS	CODING CATEGORIES	SKIP
336	In choosing a contraceptive method, what feature(s) would be most important to you?<br><br>SELECT ALL THAT APPLY.	EFFECTIVE IT IS AT PREVENTING PREGNANCY 1<br>CAN BE USED WITHOUT ANYONE ELSE KNOWING 2<br>PROTECTS AGAINST STI/HIV . . . . 3<br>DURATION OF EFFECT / LASTS LONG . . . . 4<br>NO RISK OF HARMING HEALTH . . . . 5<br>NO EFFECT ON REGULAR MONTHLY BLEEDING 6<br>NO UNPLEASANT SIDE EFFECTS . . . . 7<br>LOW COST . . . . 9<br>EASILY AVAILABLE AT THE CLNIC . . . . 10<br>CAN BE USED FOR A LONG TIME WITHOUT NEED TO VISIT CLINIC OR RE-SUPPLY . . . . 11<br>WILL BE ABLE TO GET PREGNANT WHEN I WANT 12<br>NO NEED TO GO TO A CLINIC TO OBTAIN THE METHOD 13<br>NO RISK OF INFERTILI⁻ . . . . 14<br>NO NEED TO REMEMBER USING THE METHOD 15<br>WANT TO TRY SOMETHING NEW / TIRED OF OLD METH 16<br>MY DOCTOR RECOMMENDED IT TO M . . . . 17<br>MY HUSBAND WANTED ME TO USE THIS METHOD 18<br>OTHER WOMEN IN MY FAMILY HAVE USED THIS METH(19<br>FRIENDS HAVE USED THIS METHOD 20<br>DOES NOT INTERRUPT SEX 21<br>OTHER ______________ 96<br>(SPECIFY)<br>DON'T KNOW . . . . 88<br>REFUSED 99	
337	RECORD NUMBER OF FEATURES SELECTED FROM 336.<br><br>IF MORE THAN ONE, GO TO 338	NUMBER SELECTED: [ ][ ]	
338	You made ____ choices of features that were most important to you. Based on your choices above, I will now like to ask you to rank your **top three** choices of features from most important feature (1) to least important feature (3).<br><br>1) Which of these features is the most important for you when choosing a contraceptive method?<br>2) Which of these features is the next most important for you when choosing a contraceptive method?<br>3) Which of these features is the third most important for you when choosing a contraceptive method?<br><br>RECORD RANKING OF ATTRIBUTES IN ATTRIBUTES COLUMN.<br><br>SHOW THE CHART WITH THE BOXES 1-3. TAKE OUT 20 COUNTERS.<br><br>I will now give you 20 counters. Each counter represents how important that feature is to you in choosing a contraceptive method. For each of your top three features, place the number of counters next to the feature based on how important that feature is to you. The more counters that you have next to a feature, the more important that feature is to you. You may place as many (or even all) of the counter next to any feature.<br><br>DEMONSTRATE AN EXAMPLE OF ALLOCATING COUNTERS [illegible]	RANK / ATTRIBUTE / NO. OF COUNTERS<br>1 [ ][ ] [ ][ ]<br>2 [ ][ ] [ ][ ]<br>3 [ ][ ] [ ][ ]	

NO.	QUESTIONS AND FILTERS	CODING CATEGORIES	SKIP
338A	CHECK 303 CURRENT_USE. IF CURRENTLY NOT USING:<br>Do you think you will use a contraceptive method to delay or avoid pregnancy at any time in the future?	YES . . . . 1<br>NO . . . . 2<br>DON'T KNOW . . . . 88<br>REFUSED 99	 → 339<br>→ 339<br>→ 339
338B	CHECK 303 CURRENT_USE. IF CURRENTLY NOT USING:<br>Which method would you want to use?	FEMALE STERILIZATION . . . . 1<br>MALE STERILIZATION . . . . 2<br>IUD . . . . 3<br>INJECTABLES . . . . 4<br>IMPLANTS . . . . 5<br>PILL . . . . 6<br>CONDOM . . . . 7<br>FEMALE CONDOM . . . . 8<br>DIAPHRAGM/FOAM/JELLY 9<br>TWO DAY METHOD 10<br>STANDARD DAYS METHOD . . . . 11<br>LACTATIONAL AMI . . . . 12<br>RHYTHM METHOD . . . . 13<br>WITHDRAWAL . . . . 14<br>OTHER MODERN METHOD 15<br>OTHER TRADITIONAL METH(. 16	

Now, I have some questions about family planning methods and pregnancy. By family planning methods, I mean anything a person might take, do or use to prevent becoming pregnant.

NO.	QUESTIONS AND FILTERS	CODING CATEGORIES	SKIP
339	In your opinion, during a woman's monthly cycle, are there certain days when she is more likely to become pregnant if she has sex?	YES . . . . 1<br>NO . . . . 2<br>DON'T KNOW . . . . 88<br>REFUSED 99	 → 341<br>→ 341<br>→ 341
340	For most women, the time when she is more likely to get pregnant is…	ON THE DAY OF HER PERIOD 1<br>EARLY IN HER PERIOD (DAYS 1-7) 2<br>MIDDLE OF HER PERIOD (DAYS 8-19) 3<br>END OF HER PERIOD (20-30) 4<br>DON'T KNOW . . . . 88<br>REFUSED 99	
341	In your opinion, is it possible for a woman who is breastfeeding to not become pregnant?	YES . . . . 1<br>NO . . . . 2<br>DON'T KNOW . . . . 88<br>REFUSED 99	 → 343<br>→ 343<br>→ 343

NO.	QUESTIONS AND FILTERS	CODING CATEGORIES	SKIP
342	In your opinion, for how long can breastfeeding help to prevent pregnancy?<br><br>RECORD ANSWER IN MONTHS.	NUMBER OF MONTHS . . . . . [ ][ ]<br>DON'T KNOW . . . . 88<br>REFUSED 99	
343	In your opinion, for how long should a woman who has just given birth wait before trying to get pregnant in order to minimize any health risks for her next birth?<br><br>RECORD ANSWER IN MONTHS IF LESS THAN ONE YEAR. IF RIGHT AWAY, RECORD '00'.	NUMBER OF MONTHS . . . . . [ ][ ]<br>ONE TO LESS THAN TWO YEARS 13<br>TWO TO LESS THAN THREE YEARS 14<br>THREE YEARS OR MORE . . . . 15<br>DON'T KNOW . . . . 88<br>REFUSED 99	

Now, I have some questions about your thoughts on how the use of family planning methods might affect women.

344	How likely do you think it is for a woman to gain weight while using family planning methods?	VERY UNLIKELY . . . . 1<br>SOMEWHAT UNLIKELY . . . . 2<br>NEUTRAL . . . . 3<br>SOMEWHAT LIKELY . . . . 4<br>VERY LIKELY 5	
345	How likely do you think it is for a woman's menstrual cycle to change or stop while using family planning methods?	VERY UNLIKELY . . . . 1<br>SOMEWHAT UNLIKELY . . . . 2<br>NEUTRAL . . . . 3<br>SOMEWHAT LIKELY . . . . 4<br>VERY LIKELY 5	
346	How likely do you think it is for a woman's ability to become pregnant to be affected while using family planning methods?	VERY UNLIKELY . . . . 1<br>SOMEWHAT UNLIKELY . . . . 2<br>NEUTRAL . . . . 3<br>SOMEWHAT LIKELY . . . . 4<br>VERY LIKELY 5	

As I read each of the following statements, please tell me how much you, personally, agree or disagree with each statement. Do you strongly agree, somewhat agree, somewhat disagree, or strongly disagree with the following:

347	It is too much of a hassle to use a condom every time you have sex.	STRONGLY AGREI . . . . 1<br>SOMEWHAT AGREE . . . . 2<br>NEUTRAL . . . . 3<br>SOMEWHAT DISAGREE . . . . 4<br>STRONGLY DISAGREI 5	
348	Using family planning is morally wrong.	STRONGLY AGREI . . . . 1<br>SOMEWHAT AGREE . . . . 2<br>NEUTRAL . . . . 3<br>SOMEWHAT DISAGREE . . . . 4<br>STRONGLY DISAGREI 5	
349	It doesn't matter whether you use family planning methods or not; when it is your time to get pregnant, it will happen.	STRONGLY AGREI . . . . 1<br>SOMEWHAT AGREE . . . . 2<br>NEUTRAL . . . . 3<br>SOMEWHAT DISAGREE . . . . 4<br>STRONGLY DISAGREI 5	
350	It is mainly a woman’s responsibility to make decisions about family planning methods.	STRONGLY AGREI . . . . 1<br>SOMEWHAT AGREE . . . . 2<br>NEUTRAL . . . . 3<br>SOMEWHAT DISAGREE . . . . 4<br>STRONGLY DISAGREI 5	

NO.	QUESTIONS AND FILTERS	CODING CATEGORIES	SKIP
351	In your opinion, what are some of the benefits or advantages to using family planning?<br><br>RECORD ALL MENTIONED.	PREVENTS UNWANTED PREGNANCY 1<br>ALLOWS ME TO SPACE . . . . 2<br>PROTECTS AGAINST STI/HIV . . . . . . 3<br>BENEFITS MY HEALTH . . . . 4<br>SAVES US MONEY . . . . 5<br>ALLOWS ME TO SPEND<br>MORE ON CHILDREN . . . . 6<br>ALLOWS ME TO ENJOY SEX MORE . . 7<br>OTHER (SPECIFY) . . . . 8<br>DON'T KNOW . . . . 88<br>REFUSE 99	
352	In your opinion, what are some of the costs or disadvantages to using family planning?<br><br>RECORD ALL MENTIONED	NOT EFFECTIVE/M . . . . 1<br>METHODS ARE EXPENS . . . . 2<br>TAKES TOO MUCH . . . . 3<br>EMBARASSING TO USE . . . . 4<br>SIDE EFFECTS WITH USING FP . . . . 5<br>RISK OF INFERTILITY . . . . 6<br>HARMS MY HEALTH . . . . 7<br>HUSBAND OPPOSED . . . . 8<br>OTHERS OPPOSED . . . . 9<br>RELIGIOUS OPPOSITION . . . . 10<br>INTERFERES WITH SEX . . . . 11<br>OTHER (SPECIFY) . . . . 96<br>DON'T KNOW 88	

(1) If Standard Days Method is commonly used, it may be added to the table before Lactational Amenorrhea. "**Standard Days Method** (use local term, such as CycleBeads™ , as appropriate) PROBE: A woman uses a string of colored beads to know the days she can get pregnant. On the days she can get pregnant, she uses a condom or does not have sexual intercourse." If Standard Days Method is added to Q. 301, it should also be added before LAM to Qs. 304, 314, 316, 322, and Column 1 of the calendar.

(2) The LAM method should be deleted in countries that do not have a LAM program. In these countries, LAM should also be deleted as a coding category in Qs. 304, 314, 316, 322, and Column 1 of the calendar. A description of LAM should not be provided in Q. 301.

(3) Studies have indicated emergency contraception can be effective up to five days. Verify country program recommendations and modify wording if appropriate.

(4) Other commonly used methods may be added to the list, such as contraceptive patch, contraceptive vaginal ring, or sponge. Any codes added in Q. 304 must also be added to Qs. 314, 316, 322, and Column 1 of the calendar. These methods should not be added to Q. 301.

(5) Coding categories to be developed locally and revised based on the pretest; however, the broad categories must be maintained.

(6) Year of fieldwork is assumed to be 2010. For fieldwork beginning in 2011 or 2012, the year should be 2006 or 2007, respectively.

(7) Year of fieldwork is assumed to be 2010. For fieldwork beginning in 2011 or 2012, the year should be 2005 or 2006, respectively.

(8) In countries without national fieldworker programs that include family planning, Q. 326 should be deleted.

401	CHECK 224:<br>ONE OR MORE BIRTHS IN APRIL 2018 **(1)** OR LATER ☐<br>NO BIRTHS IN APRIL 2018 **(1)** OR LATER ☐		→ 601
402	CHECK 215: ENTER IN THE TABLE THE BIRTH HISTORY NUMBER, NAME, AND SURVIVAL STATUS OF THE LAST BIRTI IN 2018. ASK THE QUESTIONS ABOUT THIS BIRTH.<br><br>Now I would like to ask some questions about your last birth.		
403	BIRTH HISTORY NUMBER FROM 212 IN BIRTH HISTORY	LAST BIRTH<br>BIRTH HISTORY NUMBER ☐☐	
404	FROM 212 AND 216	NAME ______________<br>LIVING ☐ DEAD ☐	
405	When you got pregnant with (NAME), did you want to get pregnant at that time?	YES . . . . 1<br>(SKIP TO 430)<br>NO 2	
406	Did you want to have a baby later on, or did you not want any (more) children?	LATER . . . . 1<br>NO MORE . . . . 2<br>(SKIP TO 430)	
407	How much longer did you want to wait?	MONTHS . . 1 ☐☐<br>YEARS . . 2 ☐☐<br>DON'T KNOW . . . 998	
430	When (NAME) was born, was he/she very large, larger than average, average, smaller than average, or very small?	VERY LARGE . . . . . 1<br>LARGER THAN AVERAGE . . . . . 2<br>AVERAGE . . . . 3<br>SMALLER THAN AVERAGE . . . . . 4<br>VERY SMALL . . . . . 5<br>DON'T KNOW 8	

NO.	QUESTIONS AND FILTERS	LAST BIRTH<br>NAME _______________
447	Has your menstrual period returned since the birth of (NAME)?	YES . . . . 1<br>NO . . . . 2<br>(SKIP TO 450)
449	For how many months after the birth of (NAME) did you not have a period?	MONTHS . . . [ ][ ]<br>DON'T KNOW 98
450	CHECK 226:<br>IS RESPONDENT PREGNANT?	NOT PREG-NANT [ ]<br>PREGNANT OR UNSURE [ ] (SKIP TO 452)
451	Have you had sexual intercourse since the birth of (NAME)?	YES . . . . 1<br>NO . . . . 2<br>(SKIP TO 453)
452	For how many months after the birth of (NAME) did you not have sexual intercourse?	MONTHS . . . [ ][ ]<br>DON'T KNOW 98
453	Did you ever breastfeed (NAME)?	YES . . . . 1<br>(SKIP TO 455)<br>NO 2
454	CHECK 404:<br>IS CHILD LIVING?	LIVING [ ] (SKIP TO 601)<br>DEAD [ ] (GO TO 601)

NO.	QUESTIONS AND FILTERS	LAST BIRTH<br>NAME _______________
455	How long after birth did you first put (NAME) to the breast?<br><br>IF LESS THAN 1 HOUR, RECORD '00' HOURS.<br>IF LESS THAN 24 HOURS, RECORD HOURS.<br>OTHERWISE, RECORD DAYS.	IMMEDIATELY . . . 000<br><br>HOURS 1 [ ][ ]<br><br>DAYS 2 [ ][ ]
456	In the first three days after delivery, was (NAME) given anything to drink other than breast milk?	YES . . . . 1<br>NO . . . . 2<br>(SKIP TO 458)
457	What was (NAME) given to drink?<br><br>Anything else?<br><br>RECORD ALL LIQUIDS MENTIONED.	MILK (OTHER THAN BREAST MILK) A<br>PLAIN WATER . . . B<br>SUGAR OR GLUCOSE WATER . . . C<br>GRIPE WATER . . . D<br>SUGAR-SALT-WATER SOLUTION . . . . . E<br>FRUIT JUICE . . . . . F<br>INFANT FORMULA G<br>TEA/INFUSIONS . . . H<br>COFFEE . . . . I<br>HONEY . . . . J<br><br>OTHER ___________ X<br>(SPECIFY)
458	CHECK 404:<br><br>IS CHILD LIVING?	LIVING [ ] DEAD [ ]<br>(GO TO 601)
459	Are you still breastfeeding (NAME)?	YES . . . . 1<br>NO 2

(1) Year of fieldwork is assumed to be 2010. For fieldwork beginning in 2011 or 2012, the year should be 2006 or 2007, respectively.
(2) Coding categories to be developed locally and revised based on the pretest; however, the broad categories must be maintained.
(3) Vaccination practices may vary; this question should specify where the injection is given, e.g. arm or should
(4) Syrup should be deleted in countries where syrup is not used.
(5) In countries where it is important to know the number of iron tablets taken per day, an appropriate question may be added.
(6) The question should be deleted in surveys in countries where there is no program for intermittent preventive treatment against malaria during pregnancy.

SECTION 6. MARRIAGE AND SEXUAL ACTIVITY

	Now I would like to ask you some questions about your recent sexual activity. Let me assure you again that your answers are completely confidential and will not be told to anyone. If we should come to any question that you don't want to answer, just let me know and we will go to the next question.<br><br>CHECK FOR THE PRESENCE OF OTHERS. BEFORE CONTINUING, MAKE EVERY EFFORT TO ENSURE PRIVACY.

NO.	QUESTIONS AND FILTERS	CODING CATEGORIES	SKIP
604	Is your (husband/partner) living with you now or is he staying elsewhere?	LIVING WITH HER . . . . 1<br>STAYING ELSEWHERE 2	
605	RECORD THE HUSBAND'S/PARTNER'S NAME AND LINE NUMBER FROM THE HOUSEHOLD QUESTIONNAIRE.<br>IF HE IS NOT LISTED IN THE HOUSEHOLD, RECORD '00'.<br>2	NAME ___________________________<br><br>LINE NO. . . . . [ ][ ]	
606<br>**(1)**	Does your (husband/partner) have other wives or does he live with other women as if married?	YES . . . . 1<br>NO . . . . 2<br>DON'T KNOW 8	2, 8 → 609
607<br>**(1)**	Including yourself, in total, how many wives or live-in partners does he have?	TOTAL NUMBER OF WIVES AND LIVE-IN PARTNERS . . . [ ][ ]<br><br>DON'T KNOW 98	
608<br>**(1)**	Are you the first, second, … wife?	RANK . . . . [ ][ ]	
609	Have you been married or lived with a man only once or more than once?	ONLY ONCE . . . . 1<br>MORE THAN ONCE 2	
610	CHECK 609:<br><br>MARRIED/ LIVED WITH A MAN ONLY ONCE ☐ ↓<br>In what month and year did you start living with your (husband/partner)?<br><br>MARRIED/ LIVED WITH A MAN MORE THAN ONCE ☐ ↓<br>Now I would like to ask about your first (husband/partner). In what month and year did you start living with him?	MONTH . . . . [ ][ ]<br><br>DON'T KNOW MONTH . . . . 98<br><br>YEAR . . . . [ ][ ][ ][ ]<br><br>DON'T KNOW YEAR 9998	→ 612
611	How old were you when you first started living with him?	AGE . . . . [ ][ ]	

NO.	QUESTIONS AND FILTERS	CODING CATEGORIES	SKIP
612	CHECK FOR THE PRESENCE OF OTHERS. BEFORE CONTINUING, MAKE EVERY EFFORT TO ENSURE PRIVACY.		
612B	Now I would like to ask some questions about sexual activity in order to gain a better understanding of some important life issues.	NEVER HAD SEXUAL INTERCOURSE 0	→ 624
613	How old were you when you had sexual intercourse for the very first time?	AGE IN YEARS . . . . [ ][ ]<br>FIRST TIME WHEN STARTED LIVING WITH (FIRST) HUSBAND/PARTNER 95	
615	When was the <u>last</u> time you had sexual intercourse?<br>IF LESS THAN 12 MONTHS, ANSWER MUST BE RECORDED IN DAYS, WEEKS OR MONTHS.<br>IF 12 MONTHS (ONE YEAR) OR MORE, ANSWER MUST BE RECORDED IN YEARS.	DAYS AGO . . . . 1 [ ][ ]<br>WEEKS AGO . . . . 2 [ ][ ]<br>MONTHS AGO . . . . 3 [ ][ ]<br>YEARS AGO . . . . 4 [ ][ ]	
616A	CHECK 302: EVER USED A CONTRACEPTIVE METHOD?<br>YES, USED [ ] ↓<br>NO, NEVER USED / NOT ASKED [ ]		→ 618
616	The last time that you had sexual intercourse, did you or your partner use any method(s) of family planning?	YES . . . . 1<br>NO . . . . 2<br>DON'T KNOW 8	 → 618 (2, 8)
617	Which method did you use? **(4)**<br>CIRCLE ALL MENTIONED.	FEMALE STERILIZATION . . . . A<br>MALE STERILIZATION . . . . B<br>IUD . . . . C<br>INJECTABLES . . . . D<br>IMPLANTS . . . . E<br>PILL . . . . F<br>CONDOM . . . . G<br>FEMALE CONDOM . . . . H<br>DIAPHRAGM . . . . I<br>FOAM/JELLY . . . . J<br>STANDARD DAYS METHOD . . . . K<br>LACTATIONAL AMEN. METHOD . . . . . L<br>RHYTHM METHOD . . . . M<br>WITHDRAWAL . . . . N<br>OTHER MODERN METHOD . . . . X<br>OTHER TRADITIONAL METHOD . . . Y	
618	During the past week, how often have you found yourself thinking about sex with any interest or desire?	SEVERAL TIMES A DAY 1<br>AT LEAST ONCE A DAY 2<br>AT LEAST TWICE A WEEK 3<br>AT LEAST ONCE A WEEK 4<br>NOT AT ALL 5	

NO.	QUESTIONS AND FILTERS	CODING CATEGORIES	SKIP
619A	CHECK 615: SEX IN THE PAST WEEK?<br>YES, SEX LAST WEEK ☐ ↓	NO, NO SEX IN PAST WEEK ☐ →	623
619	In the past week, how many times have you had sexual intercourse?	NUMBER OF TIMES ☐☐	
620	In the past week, how would you describe your ability to enjoy sex?	FULLY ENJOYED 1<br>SOMETIMES ENJOYED 2<br>BARELY ENJOYED 3<br>NEVER ENJOYED 4<br>DON'T KNOW 8	
621	In the past week, how satisfied were you with your sexual functioning?	COMPLETELY 1<br>HIGHLY 2<br>MODERATELY 3<br>SLIGHTLY 4<br>NOT AT ALL 5	
622	In the past week, how satisfied were you with your husband's / partner's sexual functioning?	COMPLETELY 1<br>HIGHLY 2<br>MODERATELY 3<br>SLIGHTLY 4<br>NOT AT ALL 5	
623	From a scale of 1 to 10, with 1 being "extremely dissatisfied" and 10 being "extremely satisfied", how satisfied are you with your sex life overall?	SATISFACTION ☐☐	
624	From a scale of 1 to 10, with 1 being "extremely dissatisfied" and 10 being "extremely satisfied", how satisfied are you with your marriage overall?	SATISFACTION ☐☐	
	On a scale from Strongly Disagree to Strongly Agree, to what extent do you agree with the following statements?		
625A	I often feel embarrassed or uncomfortable during sex.	STRONGLY AGREE 1<br>SOMEWHAT AGREE 2<br>NEUTRAL 3<br>SOMEWHAT DISAGREE 4<br>STRONGLY DISAGREE 5	
625B	Sex is a natural thing that women should be free to enjoy.	STRONGLY AGREE 1<br>SOMEWHAT AGREE 2<br>NEUTRAL 3<br>SOMEWHAT DISAGREE 4<br>STRONGLY DISAGREE 5	
625C	Consider the following scenario. A woman's husband privately tells her that he's been unsatisfied with her sexual performance for a long time. She has been having sex with him regularly without knowing that anything was wrong. How would you expect her to respond?	She would feel badly about herself and would wish that she performed better. 1<br>She would make more of an effort the next time they had sex. 2<br>She would think "this kind of thing is normal in a marriage." 3<br>She would be upset with her husband for not accepting her as she is. 4<br>She would feel angry and betrayed by her husband. 5<br>She would feel hurt or offended. 6	
625D	Consider the following scenario. A woman is walking through her village when she overhears a group of neighbours gossiping about her and her husband. The neighbours are laughing because the woman's husband has been telling everyone in the village that he's been unsatisfied with her sexual performance for a long time. The woman has been having sex with her husband regularly without knowing that anything was wrong. How would you expect her to respond?	She would feel badly about herself and would wish that she performed better. 1<br>She would make more of an effort the next time they had sex. 2<br>She would think "this kind of thing is normal in a marriage." 3<br>She would be upset with her husband for not accepting her as she is. 4<br>She would feel angry and betrayed by her husband. 5	

NO.	QUESTIONS AND FILTERS	CODING CATEGORIES		SKIP
		She would feel hurt or offended.	6	

Now, I have some questions about your thoughts on how the use of family planning methods might affect men's and women's desire for sex.

NO.	QUESTIONS AND FILTERS	CODING CATEGORIES	SKIP
626	How likely do you think it is for a WOMAN's desire for sex to be affected while using family planning methods?	NOT AT ALL AFFECTED . . . . 0<br>VERY UNLIKELY . . . . 1<br>SOMEWHAT UNLIKELY . . . . 2<br>NEUTRAL . . . . 3<br>SOMEWHAT LIKELY . . . . 4<br>VERY LIKELY . . . . 5<br>DEFINITELY AFFECTED 6	
627	How likely do you think it is for a MAN's desire for sex to be affected while using family planning methods?	NOT AT ALL AFFECTED . . . . 0<br>VERY UNLIKELY . . . . 1<br>SOMEWHAT UNLIKELY . . . . 2<br>NEUTRAL . . . . 3<br>SOMEWHAT LIKELY . . . . 4<br>VERY LIKELY . . . . 5<br>DEFINITELY AFFECTED 6	

NO.	QUESTIONS AND FILTERS	CODING CATEGORIES	SKIP
628	CHECK 302: EVER USED A CONTRACEPTIVE METHOD?<br>YES, USED ↓ NO, NEVER USED / NOT ASKED →		S7
630	How do you believe that your desire for sex was affected while using family planning methods?	NOT AT ALL AFFECTED . . . . 0<br>SOMEWHAT UNAFFECTED 1<br>NEUTRAL . . . . 2<br>SOMEWHAT AFFECT . . . . 3<br>DEFINITELY AFFECTED 4	
631	How do you believe that your husband's desire for sex was affected while using family planning methods?	NOT AT ALL AFFECTED . . . . 0<br>SOMEWHAT UNAFFECTED 1<br>NEUTRAL . . . . 2<br>SOMEWHAT AFFECT . . . . 3<br>DEFINITELY AFFECTED 4	

SECTION 7. FERTILITY PREFERENCES

NO.	QUESTIONS AND FILTERS	CODING CATEGORIES	SKIP
701	CHECK 304:<br>NEITHER STERILIZED ☐ ↓ HE STERILIZED ☐ →		END
702	CHECK 226:<br>PREGNANT ☐ ↓ NOT PREGNANT OR UNSURE ☐ →		704
703	Now I have some questions about the future. After the child you are expecting now, would you like to have another child, or would you prefer not to have any more children?	YES .... 1<br>NO .... 2<br>UNDECIDE[illegible] .... 3<br>UP TO GOD .... 4<br>DON'T KNOW 8	→ 705<br><br><br>→<br>711
704	Now I have some questions about the future. Would you like to have (a/another) child, or would you prefer not to have any (more) children?	YES .... 1<br>NO .... 2<br><br>UNDECIDED .... 4<br>UP TO GO[illegible] .... 5<br>DON'T KNOW 8	 → 707<br><br><br>→ 710
705	CHECK 226:<br>NOT PREGNANT OR UNSURE ☐ ↓ PREGNANT ☐ ↓<br>How long would you like to wait from now before the birth of (a/another) child? / After the birth of the child you are expecting now, how long would you like to wait before the birth of another child?	MONTHS .... 1 [ ][ ]<br>YEARS .... 2 [ ][ ]<br><br>SOON/NOW .... 993<br>SAYS SHE CAN'T GET PREGNANT 994<br>AFTER MARRIAGE .... 995<br>UP TO GOD .... 997<br><br>OTHER ________________________ 996<br>(SPECIFY)<br>DON'T KNOW 998	 <br><br>→ 710<br>→ 712<br><br><br><br>→ 710
706	CHECK 226:<br>NOT PREGNANT OR UNSURE ☐ ↓ PREGNANT ☐ →		711
707	CHECK 303: USING A CONTRACEPTIVE METHOD?<br>NOT CURRENTLY USING ☐ ↓ CURRENTLY USING ☐ →		712

NO.	QUESTIONS AND FILTERS	CODING CATEGORIES	SKIP
708	CHECK 705:<br>NOT ASKED ☐ ↓ 24 OR MORE MONTHS OR 02 OR MORE YEARS ☐ ↓ 00-23 MONTHS OR 00-01 YEAR ☐ →		711
709	CHECK 704:<br>WANTS TO HAVE A/ANOTHER CHILD ☐ ↓<br>You have said that you do not want (a/another) child soon.<br>Can you tell me why you are not using a method to prevent pregnancy?<br>Any other reasons?<br><br>WANTS NO MORE/ NONE ☐ ↓<br>You have said that you do not want any (more) children.<br>Can you tell me why you are not using a method to prevent pregnancy?<br>Any other reasons?<br><br>RECORD ALL REASONS MENTIONED.	FERTILITY-RELATED REASONS<br>NOT HAVING SEX .... 2<br>INFREQUENT SEX .... 3<br>MENOPAUSAL/HYSTERECTOMY 4<br>CAN'T GET PREGNANT .... 5<br>NOT MENSTRUATED SINCE<br>LAST BIRTH .... 6<br>BREASTFEEDING .... 7<br>UP TO GOD/FATALISTI(.............. 8<br><br>OPPOSITION TO USE<br>RESPONDENT OPPOSED .... 9<br>HUSBAND/PARTNER OPPOSED .... 10<br>OTHERS OPPOSED .... 11<br>RELIGIOUS PROHIBITION .... 12<br>SOCIAL PRESSUR .... 13<br><br>LACK OF KNOWLEDGE<br>KNOWS NO METHOD .... 14<br>KNOWS NO SOURCE .... 15<br><br>METHOD-RELATED REASONS<br>FEAR OF INFERTILITY .... 16<br>FEAR OF SIDE EFFECTS .... 17<br>INTERFERES WITH BODY'S<br>NORMAL PROCESSES .... 18<br>OTHER HEALTH CONCER .... 19<br>LACK OF ACCESS/TOO FAR .... 20<br>LONG WAITING TIME A' .... 21<br>TOO BUSY/NO TIME .... 22<br>COSTS TOO MUCH .... 23<br>PREFERRED METHOD<br>NOT AVAILABLE .... 24<br>NO METHOD AVAILABLE .... 25<br>INCONVENIENT TO USE .... 26<br>NOT EFFECTIVE .... 27<br>OTHER ________________ 96<br>(SPECIFY)<br>DON'T KNOW 88	
710	CHECK 303: USING A CONTRACEPTIVE METHOD?<br>NOT ASKED ☐ ↓ NO, NOT CURRENTLY USING ☐ ↓ YES, CURRENTLY USING ☐ →		712

NO.	QUESTIONS AND FILTERS	CODING CATEGORIES	SKIP
711A	Do you think you will use a contraceptive method to delay or avoid pregnancy at any time in the future?	YES . . . 1<br>NO . . . 2<br>DON'T KNOW . . . 8	1 → 712
711B	Which of the following are reasons why you don't think that you will use a contraceptive method in the future?<br><br>RECORD ALL REASONS MENTIONED.	FERTILITY-RELATED REASONS<br>WANTS ANOTHER CHILD SOON . . . 1<br>NOT HAVING SEX . . . 2<br>INFREQUENT SEX . . . 3<br>MENOPAUSAL/HYSTERECTOMY 4<br>CAN'T GET PREGNANT . . . 5<br>NOT MENSTRUATED SINCE LAST BIRTH . . . 6<br>BREASTFEEDING . . . 7<br>UP TO GOD/FATALISTIC . . . 8<br><br>OPPOSITION TO USE<br>RESPONDENT OPPOSED . . . 9<br>HUSBAND/PARTNER OPPOSED . . . 10<br>OTHERS OPPOSED . . . 11<br>RELIGIOUS PROHIBITION . . . 12<br>SOCIAL PRESSUR . . . 13<br><br>LACK OF KNOWLEDGE<br>KNOWS NO METHOD . . . 14<br>KNOWS NO SOURCE . . . 15<br><br>METHOD-RELATED REASONS<br>FEAR OF INFERTILITY . . . 16<br>FEAR OF SIDE EFFECTS . . . 17<br>INTERFERES WITH BODY'S NORMAL PROCESSES . . . 18<br>OTHER HEALTH CONCERN . . . 19<br>LACK OF ACCESS/TOO FAR . . . 20<br>LONG WAITING TIME A . . . 21<br>TOO BUSY/NO TIME . . . 22<br>COSTS TOO MUCH . . . 23<br>PREFERRED METHOD NOT AVAILABLE . . . 24<br>NO METHOD AVAILABLE . . . 25<br>INCONVENIENT TO USE . . . 26<br>NOT EFFECTIVE . . . 27<br>OTHER ____________ 96<br>(SPECIFY)<br>DON'T KNOW . . . 88	
712	CHECK 216:<br>HAS LIVING CHILDREN ☐ / NO LIVING CHILDREN ☐<br><br>HAS LIVING CHILDREN: If you could go back to the time you did not have any children and could choose exactly the number of children to have in your whole life, how many would that be?<br><br>NO LIVING CHILDREN: If you could choose exactly the number of children to have in your whole life, how many would that be?<br><br>PROBE FOR A NUMERIC RESPONSE.	NONE . . . 00<br>NUMBER . . . [ ][ ]<br><br>OTHER ____________ 96<br>(SPECIFY)	00 → 713B<br>96 → 713B

NO.	QUESTIONS AND FILTERS	CODING CATEGORIES	SKIP
713	How many of these children would you like to be boys, how many would you like to be girls and for how many would it not matter if it's a boy or a girl?	BOYS / GIRLS / EITHER<br>NUMBER [ ][ ] [ ][ ] [ ][ ]<br>OTHER ________________ 96<br>(SPECIFY)	
713B	CHECK 216:<br>HAS LIVING CHILDREN [ ] ↓ NO LIVING CHILDREN [ ] ↓ GO TO 714A.<br>If NAME OF YOUNGEST CHILD could have X number of children, how many of these children would you like to be boys, how many would you like to be girls and for how many would it not matter if it's a boy or a girl?	BOYS / GIRLS / EITHER<br>NUMBER [ ][ ] [ ][ ] [ ][ ]<br>OTHER ________________ 96<br>(SPECIFY)	
714A	In the last few months have you heard about family planning:<br>On the radio?<br>On the television?<br>In a newspaper or magazine?<br>On a poster?<br>On clothing (i.e., cap, chitenji, t-shirt)?<br>In a drama?<br>Somewhere else?	YES NO<br>RADIO . . . . 1111 2<br>TELEVISION . . . . 1111 2<br>NEWSPAPER OR MAGAZINI . . . . 1111 2<br>POSTER . . . . 1111 2<br>CLOTHING . . . . 1111 2<br>DRAMA . . . . 1111 2<br>OTHER 1111 2	
714B	In the last few months, have you listened to any of the following program series about family planning or health on the<br>Safe motherhood?<br>Phukusi la Moyo?<br>Radio Doctor/Doctor wapawairesi?<br>Umoyo M'Malawi?<br>Tikuferanji?<br>Chitukuku M'Malawi?<br>Uku ndiko kudya?<br>Other?	YES NO<br>SAFE MOTHERHOOD . . . . . . 1 2<br>PHUKUSI LA MOYO . . . . . . 1 2<br>RADIO DOCTOR . . . . . . 1 2<br>UMOYO M'MALAWI . . . . . . 1 2<br>TIKUFERANJI . . . . . . 1 2<br>CHITUKUKU M'MALAWI . . . . . . 1 2<br>UKU NDIKO KUDYA . . . . . . 1 2<br>OTHER 1 2	
716	CHECK 601:<br>YES, CURRENTLY MARRIED [ ] ↓ YES, LIVING WITH A MAN [ ] ↓ NO, NOT IN UNION [ ] →		801
717	CHECK 303: USING A CONTRACEPTIVE METHOD?<br>CURRENTLY USING [ ] ↓ NOT CURRENTLY USING OR NOT ASKED [ ] →		718C
720	Does your (husband/partner) want the same number of children that you want, or does he want more or fewer than you want?	SAME NUMBER . . . . 1<br>MORE CHILDREN . . . . 2<br>FEWER CHILDREN . . . . 3<br>DON'T KNOW . . . . 88<br>REFUSED 98	
718A	Does your husband/partner know that you are using a method of family planning?	YES . . . . 1<br>NO . . . . 2<br>DON'T KNOW 8	

NO.	QUESTIONS AND FILTERS	CODING CATEGORIES	SKIP
718B	Would you say that using contraception is mainly your decision, mainly your (husband's/partner's) decision, or did you both decide together?	MAINLY WOMAN . . . . 1<br>MOST WOMAN, SOME HUSBAND 2<br>JOINT DECISION . . . . 3<br>MOST HUSBAND, SOME WOMAN 4<br>MAINLY HUSBAND 5<br>OTHER ______________ 96<br>(SPECIFY)	→ 718D
718C	Would you say that NOT using contraception is mainly your decision, mainly your (husband's/partner's) decision, or did you both decide together?	MAINLY WOMAN . . . . 1<br>MOST WOMAN, SOME HUSBAND 2<br>JOINT DECISION . . . . 3<br>MOST HUSBAND, SOME WOMAN 4<br>MAINLY HUSBAND 5<br>OTHER ______________ 6<br>(SPECIFY)	
719	CHECK 304:<br>NEITHER STERILIZED ☐ ↓ HE OR SHE STERILIZED ☐ →		801
721	If you were to not use any family planning method, how likely do you think it is that you will become pregnant during the next year?	VERY UNLIKELY . . . . 1<br>SOMEWHAT UNLIKELY . . . . 2<br>NEUTRAL . . . . 3<br>SOMEWHAT LIKELY . . . . 4<br>VERY LIKELY 5	
722	CHECK 303: USING A CONTRACEPTIVE METHOD?<br>CURRENTLY USING ☐ ↓ NOT CURRENTLY USING OR NOT ASKED ☐ →		725A
723	If you were to continue to use your family planning method, how likely do you think it is that you would become pregnant during the next year?	VERY UNLIKELY . . . . 1<br>SOMEWHAT UNLIKELY . . . . 2<br>NEUTRAL . . . . 3<br>SOMEWHAT LIKELY . . . . 4<br>VERY LIKELY 5	
724A	Different methods of family planning vary in how effective or ineffective they are in preventing pregnancy. How effective do you think that your family planning method is in preventing pregnancy?	VERY EFFECTIVE . . . . 1<br>SOMEWHAT EFFECTIV . . . . 2<br>UNSURE/NEUTRAL . . . . 3<br>SOMEWHAT INEFFECT . . . . 4<br>VERY INEFFECTIVE . . . . 5<br>DON'T KNOW . . . . 88<br>REFUSED 99	

NO.	QUESTIONS AND FILTERS	CODING CATEGORIES	SKIP
724B	Different methods of family planning vary in how convenient they are to use. How convenient is it to use your family planning method?	VERY CONVENIENT . . . . 1<br>SOMEWHAT CONVENII . . . . 2<br>UNSURE/NEUTRA . . . . 3<br>SOMEWHAT INCONVENIE . . . . 4<br>VERY INCONVENIENT . . . . 5<br>DON'T KNOW . . . . 88<br>REFUSED 99	
724C	Overall, how satisfied are you with your current family planning method?	VERY SATISFIED . . . . 1<br>SOMEWHAT SATISFIEI . . . . 2<br>UNSURE/NEUTRA . . . . 3<br>SOMEWHAT UNSATISFIE . . . . 4<br>VERY UNSATISFIED 5	
724D	Overall, how satisfied is your husband with your current family planning method?	VERY SATISFIED . . . . 1<br>SOMEWHAT SATISFIEI . . . . 2<br>UNSURE/NEUTRA . . . . 3<br>SOMEWHAT UNSATISFIE . . . . 4<br>VERY UNSATISFIED 5	
725A	Have you ever faced any problems or negative experiences from discussing, accessing, or using a method of family planning?	YES . . . . 1<br>NO . . . . 2<br>DON'T KNOW . . . . 88<br>REFUSED 99	(NO, DON'T KNOW, REFUSED) → 725
725B	What negative experiences have you faced when <u>discussing</u> family planning with your family, friends, or provider?<br><br>SELECT ALL MENTIONED.	Husband not supportive in FP decisi . . . . 1<br>Mother-in-law not supportive in FP de . . . . 2<br>Father-in-law not supportive in FP decision . . . 3<br>Provider not supportive in FP decisio . . . . 4<br>Friends not supportive in FP decisior . . . . 5<br>Felt that using FP methods might threaten<br>marriage/friendships . . . . 6<br>None . . . . 7<br>OTHER (SPECIFY) . . . . 96<br>DON'T KNOW . . . . 88<br>REFUSED 99	
725C	What negative experiences have you faced when <u>accessing</u> family planning?<br><br>SELECT ALL MENTIONED.	Provider not qualified to give FP 1<br>Expensive to get FF . . . . 3<br>Difficult to travel to get FP . . . . 4<br>Clinic did not have FP / the method I wante ..... 6<br>Clinic refused to provide FP method .... 7<br>I did not have anyone to go with to FP clini .... 9<br>Lack of social support . . . . 10<br>I was nervous / not confident talking to the FP<br>provider about my needs / asking for informa 11<br>Provider did not treat me well /dismissed<br>my questions . . . . 12<br>OTHER (SPECIFY) . . . . 96<br>DON'T KNOW . . . . 88<br>REFUSED 99	
725D	What negative experiences have you faced with <u>using</u> family planning methods?<br><br>SELECT ALL MENTIONED.	HEADACHE . . . . 1<br>DIZZINESS . . . . 2<br>BREAST TENDERNESS 3<br>NAUSEA . . . . 4<br>IRREGULAR BLEEDING .... 5<br>HEAVY BLEEDING ..... 6<br>VAGINAL IRRITATION 7<br>SPOTTING . . . . 8<br>MENSTRUAL CRAMPS 9<br>WEIGHT GAIN . . . .<br>CHANGE IN LIBIDO 10<br>MOOD SWINGS . . . .<br>RAISED BLOOD PRESSURE 11<br>BLOOD CLOTS<br>OTHER (SPECIFY) . . . . 96<br>DON'T KNOW . . . . 88<br>REFUSED 99	
725	If you were to get pregnant within the next year, would it be:<br><br>READ OUT OPTIONS AND CHOOSE ONE RESPONSE.	THE WORST THING THAT COULD<br>HAPPEN TO YOU . . . . 1<br>VERY BAD . . . . 2<br>SOMEWHAT BAD . . . . 3<br>NEUTRAL . . . . 4<br>SOMEWHAT GOOD . . . . 5<br>VERY GOOD . . . . 66<br>THE BEST THING THAT COULD<br>HAPPEN TO YOU 7	

(1) These questions have been added by the researchers to identify sources of non-use and intention to use.

NO.	QUESTIONS AND FILTERS	CODING CATEGORIES	SKIP
806	As you know, some men take up jobs for which they are paid in cash or kind. Others sell things, have a small business or work on the family farm or in the family business. Does your husband do any of these things or any other work in the last seven days?	YES . . . . 1<br>NO . . . . 2<br>DON'T KNOW . . . . 88<br>REFUSED 99	1 → 809
807	Although your husband did not work in the last seven days, does he have any job or business from which you were absent for leave, illness, vacation, maternity leave or any other such reason?	YES . . . . 1<br>NO . . . . 2<br>DON'T KNOW . . . . 88<br>REFUSED 99	1 → 809
808	Has your husband done any work in the last 12 months?	YES . . . . 1<br>NO . . . . 2<br>DON'T KNOW . . . . 88<br>REFUSED 99	2, 88, 99 → END
809	Does your husband do this work for a member of your family, for someone else, or are you self-employed?	FOR FAMILY MEMBER . . . . 1<br>FOR SOMEONE ELSE . . . . 2<br>SELF-EMPLOYED 3	
810	Is your husband paid in cash or kind for this work, or is he not paid at all?	CASH ONLY . . . . 1<br>CASH AND KIND . . . . 2<br>IN KIND ONLY . . . . 3<br>NOT PAID 4	

SECTION 9. HUSBAND'S PREFERENCES, HOUSEHOLD BARGAINING, EMPOWERMENT

NO.	QUESTIONS AND FILTERS	CODING CATEGORIES	SKIP
901	Who in your household makes decisions on big purchases (refrigerator, cars, houses, etc.)?	MAINLY WOMAN . . . . 1<br>MOST WOMAN, SOME HUSBAND 2<br>JOINT DECISION . . . . 3<br>MOST HUSBAND, SOME WOMAN 4<br>MAINLY HUSBAND 5<br>OTHER HOUSEHOLD MEMBER . 6<br>OTHER FRIENDS OR RELATIVES 7<br>OTHER ________ 96<br>(SPECIFY)	
902	Who in your household makes decisions on your children's education (which school they go to, paying for school fees, uniforms, taking	MAINLY WOMAN . . . . 1<br>MOST WOMAN, SOME HUSBAND 2<br>JOINT DECISION . . . . 3<br>MOST HUSBAND, SOME WOMAN 4<br>MAINLY HUSBAND 5<br>OTHER HOUSEHOLD MEMBER . 6<br>OTHER FRIENDS OR RELATIVES 7<br>OTHER ________ 96<br>(SPECIFY)	
903	Who usually decides how the money you earn will be used: mainly you, mainly your husband/partner, or you and your husband/partner jointly?	MAINLY WOMAN . . . . 1<br>MOST WOMAN, SOME HUSBA . . . . . 2<br>JOINT DECISION . . . . 3<br>MOSTLY HUSBAND, SOME WOM . . . 4<br>MAINLY HUSBAND . . . . 5<br>OTHER ________ 96<br>(SPECIFY)	
904	Would you say that the money that you earn is more than what your husband/partner earns, less than what he earns, or about the same?	MORE THAN HIM . . . . 1<br>LESS THAN HIM . . . . 2<br>ABOUT THE SAME . . . . 3<br>HUSBAND/PARTNER DOESN'T<br>BRING IN ANY MONEY . . . . 4<br>DON'T KNOW 88	 <br><br><br>4 → 906
905	Who usually decides how your husband's/partner's earnings will be used: you, your husband/partner, or you and your husband/partner jointly?	MAINLY WOMAN . . . . 1<br>MOST WOMAN, SOME HUSBA . . . . . 2<br>JOINT DECISION . . . . 3<br>MOSTLY HUSBAND, SOME WOM . . . 4<br>MAINLY HUSBAND . . . . 5<br>OTHER ________ 96<br>(SPECIFY)	
906	Who usually makes decisions about health care for yourself: you, your husband/partner, you and your husband/partner jointly, or someone else?	MAINLY WOMAN 1<br>MOST WOMAN, SOME HUSBAND 2<br>JOINT DECISION . . . . 3<br>MOSTLY HUSBAND, SOME WOM . . . 4<br>MAINLY HUSBAND . . . . 5<br>SOMEONE ELSE . . . . 6<br>OTHER ________ 96<br>(SPECIFY)	

NO.	QUESTIONS AND FILTERS	CODING CATEGORIES	SKIP
908	Who usually makes decisions about making purchases for daily household needs?	MAINLY WOMAN . . . . 1<br>MOST WOMAN, SOME HUSBA . . . . . 2<br>JOINT DECISION . . . . 3<br>MOSTLY HUSBAND, SOME WOM . . . 4<br>MAINLY HUSBAND . . . . 5<br>SOMEONE ELSE . . . . 6<br>OTHER ______________ 96<br>(SPECIFY)	
909	Who usually makes decisions about visits to your family or relatives?	MAINLY WOMAN . . . . 1<br>MOST WOMAN, SOME HUSBA . . . . . 2<br>JOINT DECISION . . . . 3<br>MOSTLY HUSBAND, SOME WOM . . . 4<br>MAINLY HUSBAND . . . . 5<br>SOMEONE ELSE . . . . 6<br>OTHER ______________ 96<br>(SPECIFY)	
910A	Do you have any money of your own that you alone can decide how to use?	YES . . . . 1<br>NO 2	
910B	Do you own this or any other house either alone or jointly with someone else?	ALONE ONLY . . . . 1<br>JOINTLY ONLY . . . . 2<br>ALONE AND JOINTLY . . . . 3<br>DOES NOT OWN 4	
911	Do you own any land either alone or jointly with someone else?	ALONE ONLY . . . . 1<br>JOINTLY ONLY . . . . 2<br>ALONE AND JOINTLY . . . . 3<br>DOES NOT OWN 4	
911A	Are you usually allowed to go to the following places:<br>A. To the market?<br>B. To the health facility?<br>C. To the center of town?<br>D. To short distance bus?<br>E. To the home of relatives or friends (in the neighborhood)<br>F. Outside Lilongwe?	Alone =1, With someone else = 2, Not at all = 3<br>a. MARKET 1 2 3<br>b. HEALTH 1 2 3<br>c. CENTER OF TOWN 1 2 3<br>d. BUS 1 2 3<br>e. REL/FRIEND HOME 1 2 3<br>f. OUTSIDE LILONGW 1 2 3	
912	PRESENCE OF OTHERS AT THIS POINT (PRESENT AND LISTENING, PRESENT BUT NOT LISTENING, OR NOT PRESENT)	PRES./ LISTEN. · PRES./ NOT LISTEN. · NOT PRES.<br>CHILDREN < 10 . . . . . 1 2 3<br>HUSBAND . . . . 1 2 3<br>OTHER MALES . . . . . 1 2 3<br>OTHER FEMALES . . . 1 2 3	
913	Sometimes a husband is annoyed or angered by things that his wife does. In your opinion, is a husband justified in hitting or beating his wife in the following situations:<br>If she goes out without telling him?<br>If she neglects the children?<br>If she argues with him?<br>If she refuses to have sex with him?<br>If the food is not properly cooked?	YES NO DK<br>GOES OUT . . . . 1 2 8<br>NEGL. CHILDREN . . . 1 2 8<br>ARGUES . . . . 1 2 8<br>REFUSES SEX . . . . . 1 2 8<br>FOOD 1 2 8	
914A<br>914B<br>914C	1.It is legal for a husband to punish his wife by withholding money<br>2. It is legal for a woman to be refused property inheritance by her husb<br>3. It is legal for a husband to kick his wife out of the house if they have	YES . . . . 1<br>NO . . . . 2<br>DON'T KNOW 88	
914D<br><br>914E	On a scale from 1 to 5, where 1 is Strongly Disagree and 5 is Strongly Agree, to what extent do you agree with the following statements?<br>a)The formal legal system is more trustworthy and reliable than community-based justice<br>b)Community-based justice is more trustworthy and reliable than the formal legal system	STRONGLY DISAGREE · STRONGLY AGREE<br>a. 1 2 3 4 5<br>b. 1 2 3 4 5<br>c. 1 2 3 4 5	

NO.	QUESTIONS AND FILTERS	CODING CATEGORIES	SKIP
914	Have you ever discussed political issues, such as the recent election, with your husband?	YES . . . . 1<br>NO 2	 → 916
915	How often do you talk about political issues with your husband?	VERY FREQUENTI . . . . 1<br>SOMEWHAT FREQUENTL . . . . . 2<br>OCCASIONALLY . . . . 3<br>RARELY . . . . 4<br>NEVEF 5	
915B	Did you and your husband vote for the same political party in the recent election?	YES . . . . 1<br>NO . . . . 2<br>WOMAN DID NOT VOTE . . . . . 3<br>HUSBAND DID NOT VOTE . . . . . 4<br>WOMAN, HUSBAND DID NOT VOTE 5<br>DON'T KNOW . . . . 88<br>REFUSED 99	
916	Have you discussed with husband how many more children you want?	YES . . . . 1<br>NO . . . . 2<br>DON'T KNOW . . . . 88<br>REFUSED 99	 → 920 (NO, DON'T KNOW, REFUSED)
917	How many more sons does your husband want to have?	NUMBER OF BOYS . . . . [ ][ ]	
918	How many more daughters does your husband want to have?	NUMBER OF GIRLS . . . . [ ][ ]	
919	SUM ANSWERS TO 917 AND 918, AND ENTER TOTAL.<br>IF NONE, RECORD '00'.	TOTAL DESIRED BIRTHS by H [ ][ ]	

NO.	QUESTIONS AND FILTERS	CODING CATEGORIES	SKIP
925	Have you ever discussed using family planning methods with your husband?	YES . . . . 1<br>NO 2	1 → 927
926	Why have you not discussed using family planning methods with your husband?<br>RECORD ALL REASONS MENTIONED.	FERTILITY-RELATED REASONS<br>WANTS ANOTHER CHILD SOON . 1<br>NOT HAVING SEX . . . . 2<br>INFREQUENT SEX . . . . 3<br>MENOPAUSAL/HYSTERECTOMY 4<br>CAN'T GET PREGNANT . . . . 5<br>NOT MENSTRUATED SINCE<br>LAST BIRTH . . . . 6<br>BREASTFEEDING . . . . 7<br>UP TO GOD/FATALISTI(. . . . . 8<br><br>OPPOSITION TO USE<br>RESPONDENT OPPOSED . . . . 9<br>HUSBAND/PARTNER OPPOSE . . . 10<br>OTHERS OPPOSED . . . . 11<br>RELIGIOUS PROHIBITION . . . . 12<br>EMBARRASSED/ASHAME . . . . 13<br><br>LACK OF KNOWLEDGE<br>KNOWS NO METHOE . . . . 14<br>KNOWS NO SOURCE . . . . 15<br>OTHER ____________________ 96<br>(SPECIFY)<br>DON'T KNOW . . . . 88<br>REFUSED 99	
927	Which topics about family planning have you discussed with your husband?<br><br>RECORD ALL REASONS MENTIONED.	NUMBER OF CHILDREN . . . . 1<br>CONTRACEPTION . . . . 2<br>BIRTH SPACING AND TIMING 3<br>FERTILITY AND INFERTILITY . . . . . 4<br>SEX AND SEXUAL SATISFACTIOI . . . 5<br>OTHER 96	
927A	When was the last time that you discussed such matters with him?	WITHIN THE LAST WEEK . . . . 1<br>WITHIN THE LAST MONTH . . . . 2<br>IN THE LAST 6 MONTHS . . . . 3<br>MORE THAN 6 MONTHS AGO . . . . . 4<br>DON'T KNOW 88	
928	On a scale of 1 to 5, with 1 being strongly supportive and 5 being strongly opposed, how do you believe your husband feels towards using family planning methods?	STRONGLY SUPPORTIVE . . . . 1<br>SOMEWHAT SUPPORTIVE . . . . 2<br>NEUTRAL . . . . 3<br>SOMEWHAT OPPOSED . . . . 4<br>STRONGLY OPPOSED 5	→
930	Why do you think that your husband does not approve of family planning?		

	Y	N
1. He wants more children — MORE CHILDREN	1	2
2. He thinks using FP makes women promiscuous — PROMISCUOUS	1	2
3. Religious reasons — RELIGIOUS REASONS	1	2
4. His mother/family does not approve — MIL/FAMILY	1	2
5. He worries about side effecs — SIDE EFFECTS	1	2
6. He worries about infertility/impotence — INFERTILITY	1	2
7. He thinks using FP interferes with sex — INTERFERES WITH SEX	1	2
8. He doesn't think that FP is effective at preventing pregnancy — NOT EFFECTIVE	1	2
9. He does not know of any FP method — KNOWS NO METHOD	1	2
10. He does not know where to get methods — KNOWS NO SOURCE	1	2
11. He is embarrassed or afraid of using FP — EMBARRASSED	1	2
12. He fears stigma from friends / family / community — FEARS STIGMA	1	2

SECTION 10. OTHER HEALTH ISSUES

NO.	QUESTIONS AND FILTERS	CODING CATEGORIES	SKIP
1008	Many different factors can prevent women from getting family planning advice or treatment for themselves. When you want to get family planning advice or treatment, is each of the following a big problem or not?	BIG PROB-LEM / NOT A BIG PROB-LEM	
	Opposition from husband or partner?	HUSBAND OPPOSITIOI . 1 2	
	Getting permission to go to the doctor?	PERMISSION TO GO . . . 1 2	
	Getting money needed for advice or treatment?	GETTING MONEY 1 2	
	The distance to the health facility?	DISTANCE 1 2	
	Not wanting to go alone?	GO ALONE 1 2	
	Not having time because of work?	NO TIME DUE TO WOR . 1 2	
	Social pressure from relatives, friends, and neighbors?	SOCIAL PRESSURE . 1 2	
1009	Are you covered by any health insurance? **(2)**	YES . . . . 1<br>NO . . . . 2<br>DON'T KNOW . . . . 88<br>REFUSED 99	NO → 1011
1010	What type of health insurance are you covered by? **(2)**<br><br>RECORD ALL MENTIONED.	MUTUAL HEALTH ORGANIZATION/ COMMUNITY-BASED HEALTH INSURANCE . . . . A<br>HEALTH INSURANCE THROUGH EMPLOYEF . . . . B<br>SOCIAL SECURITY . . . . C<br>OTHER PRIVATELY PURCHASED COMMERCIAL HEALTH INSURANC D<br>OTHER ______________ X<br>(SPECIFY)	
1011	On a scale from 1 to 5, where 1 is Strongly Disagree and 5 is Strongly Agree, to what extent do you agree with the following statements?	STRONGLY DISAGREE ... STRONGLY AGREE	
	a. Patients have sometimes been deceived or misled by health care organizations	a. 1 2 3 4 5	
	b. Health care organizations have sometimes done harmful experiments on patients	b. 1 2 3 4 5	
	c. Mistakes are common in health care organizations	c. 1 2 3 4 5	
1012	In the last 12 months, have you:	YES NO	
	1. failed to take your physician's advice?	a 1 2	
	2. failed to seek medical care when you felt you needed it?	b 1 2	
	3. postponed or delayed seeking care you felt you needed?	c 1 2	
1013	Have you ever gone to any health facility for reproductive health, fertility, or family planning services?	YES . . . . 1<br>NO . . . . 2<br>DON'T KNOW . . . . 88<br>REFUSED 99	NO → 1015
1014	In the last year, how often have you seen a family planning provider'	Once a year . . . . 1<br>Once every 6 montl . . . . 2<br>Once every 3 montl . . . . 3<br>Once every month . . . . 4<br>More than once every month 5	

NO.	QUESTIONS AND FILTERS	CODING CATEGORIES	SKIP
1015	On a scale of 1 to 5, with 5 being excellent and 1 being terrible, how would you rate your last experience going to a health facility for family planning or reproductive health services?	Rating . . . . [ ][ ]	
1016	Would you be more likely to go to a health facility for family planning services if…<br>a. someone paid for the cost of transportation?<br>b.your husband is invited to come with you?<br>c. you were given counseling for family planning<br>d. someone covered the cost of services at the clinic?	Y N<br>PAID FOR TRANSPORT 1 2<br>HUSBAND INVITED 1 2<br>COUNSELING 1 2<br>COVERED SERVICES 1 2	
1017	RECORD THE TIME.	HOUR . . . . [ ][ ]<br>MINUTES . . . . [ ][ ]	

(1) Add local terms.
(2) If a health service prepayment plan or other types of plans are available in the country, add those types of plans to the question.

INSTRUCTIONS:
ONLY ONE CODE SHOULD APPEAR IN ANY BOX.
COLUMN 1 REQUIRES A CODE IN EVERY MONTH.

INFORMATION TO BE CODED FOR EACH COLUMN

COLUMN 1: BIRTHS, PREGNANCIES, CONTRACEPTIVE USE**

- B BIRTHS
- P PREGNANCIES

- T TERMINATIONS

- 0 NO METHOD
- 1 FEMALE STERILIZATION
- 2 MALE STERILIZATION
- 3 IUD
- 4 INJECTABLES
- 5 IMPLANTS
- 6 PILL
- 7 CONDOM
- 8 FEMALE CONDOM
- 9 DIAPHRAGM
- J FOAM OR JELLY
- K STANDARD DAYS METHOD/CYCLEBEADS
- L LACTATIONAL AMENORRHEA METHOD
- M RHYTHM METHOD
- N WITHDRAWAL
- X OTHER MODERN METHOD
- Y OTHER TRADITIONAL METHOD

COLUMN 2: DISCONTINUATION OF CONTRACEPTIVE USE

- 0 INFREQUENT SEX/HUSBAND AWAY
- 1 BECAME PREGNANT WHILE USING
- 2 WANTED TO BECOME PREGNANT
- 3 HUSBAND/PARTNER DISAPPROVED
- 4 WANTED MORE EFFECTIVE METHOD
- 5 SIDE EFFECTS/HEALTH CONCERNS
- 6 LACK OF ACCESS/TOO FAR
- 7 COSTS TOO MUCH
- 8 INCONVENIENT TO USE
- F UP TO GOD/FATALISTIC
- A DIFFICULT TO GET PREGNANT/MENOPAUSAL
- D MARITAL DISSOLUTION/SEPARATION
- X OTHER ______________________ (SPECIFY)
- Z DON'T KNOW

* Year of fieldwork is assumed to be 2010. For fieldwork beginning in 2011 or 2012, the years should be adjusted.

** Response categories may be added for other methods, including fertility awareness methods.

				1	2	
	4	APR	01			
	5	MAY	02			
	6	JUN	03			
	7	JUL	04			
2	8	AUG	05			2
0	9	SEP	06			0
1	10	OCT	07			1
8	11	NOV	08			8
*	12	DEC	09			*
2	1	JAN	10			2
0	2	FEB	11			0
1	3	MAR	12			1
9	4	APR	13			9
	5	MAY	14			
	6	JUN	15			

FOLLOW-UP INFORMATION AND END OF SURVEY

FOLLOW-UP INFORMATION

IS THE WOMAN WHO IS ANSWERING THIS SECTION THE SAME PERSON WHO ANSWERED THE HOUSEHOLD SECT Y N

IF YES, SKIP BELOW TO END OF SURVEY.

Thank you for participating in this survey. We may contact your household again in the future to learn more about how life changes for Malawian families. Could you please give us information about two people who **DO NOT LIVE IN THE HOUSEHOLD** and who would know where you or other household members are, or how to reach you, in the future?

CONTACT 1

FULL NAME: ______________________________

RELATIONSHIP TO YOU: ______________________________

FULL ADDRESS: ______________________________

____

____

PHONE NUMBER: ______________________________

E-MAIL ADDRESS: ______________________________

CONTACT 2

FULL NAME: ______________________________

RELATIONSHIP TO YOU: ______________________________

FULL ADDRESS: ______________________________

____

____

PHONE NUMBER: ______________________________

E-MAIL ADDRESS: ______________________________

OTHER HOUSEHOLD CONTACT:

What is the name and phone number of someone else within your household?

NAME: ______________________________

PHONE: ______________________________

RELATIONSHIP TO YOU: ______________________________

PLANS TO MOVE:

Does your family/household have any plans to move in the next three months? YES NO

IF YES: Where do you plan to move to? ADDRESS: ______________________________

____

____

PHOTO OF RESPONDENT:

With your permission, I would now like to take a photo of you. Taking your photo will help us to find you again in the future.

May I take your photo now?

YES NO

IF YES, PLEASE TAKE A PHOTO OF THE RESPONDENT. BE SURE TO CLEARLY CAPTURE THE RESPONDENT'S FACE FROM THE NECK UP.

END OF SURVEY:

You have now reached the end of the survey. Thank you for your time and ideas. This has been extremely helpful. As I said in the beginning, the purpose of this discussion was to help me learn about women's health in Malawi. I also want to remind you that all of your responses will remain confidential. Finally, I ask that you not share the details of what was said here. If you are asked about this study, please use only general descriptions, such as "I was gathering information about women and men and health issues."

How does that sound to you? Do you have any further questions for me at this time? If you would like to speak with me in private, I will stay here after we end.

Thank you again for your help.

SIGNATURE OF INTERVIEWER: ______________________ DATE: ______________

END. GO TO INTERVIEWER OBSERVATIONS.

DATE ____________________

COUNSELOR VISIT FORM
MALAWI BEHAVIORAL BIASES STUDY
BOSTON UNIVERSITY, IPA MALAWI

SECTION 0: STAFF IDENTIFICATION

NAME OF FIELD MANAGER / STAFF ____________________

FIELD STAFF MEMBER ID NUMBE . . . . [ ][ ][ ][ ]

NAME OF COUNSELOR ____________________

COUNSELOR ID NUMBE . . . . [ ][ ][ ][ ]

COUNSELOR PHONE NUMBE . . . . [ ][ ][ ][ ][ ][ ][ ][ ][ ][ ][ ][ ][ ][ ]

SECTION 1: RESPONDENT IDENTIFICATION

NAME OF RESPONDENT ____________________

IDENTIFICATION OF ELIGIBLE WOMAN:
Hello. My name is _________________ (COUNSELOR NAME) and I am a family health counselor from Innovations for Poverty Action (IPA) Malawi, located in Area 47, Lilongwe. I am here today to speak to (NAME OF ELIGIBLE WOMAN) about a health program for women.

1	Could you tell me if she is at home and available to speak to me at this time?	YES . . . . 1<br>NO 2	→ 3A
2A	Thank you for your time. When would be a good time to return to speak to (NAME OF ELIGIBLE WOMAN)?	DATE: ____________________<br><br>TIME: ____________________	
2B	Thank you for your time. I look forward to returning to speak with (NAME OF ELIGIBLE WOMAN) soon.<br>Thank you for your time. I hope that you have a good day.	END VISIT.<br><br>SAVE FORM AS INCOMPLETE. RETURN AT SPECIFIED DATE AND	END
3A	Thank you. At this time, I would now like to meet (NAME OF ELIGIBLE WOMAN).<br><br>IDENTIFY AND MEET ELIGIBLE WOMAN RESPONDENT.<br><br>VISUALLY CONFIRM IDENTITY OF ELIGIBLE WOMAN WITH PHOTO FROM PROGRAM ID CARD.<br><br>CONFIRM THAT THE INTRODUCTION IS CONDUCTED IN A PRIVATE ROOM.	GO TO INTRODUCTION	

WOMAN PROGRAM ID NUMBER | | | | |

CONFIRM THE FOLLOWING WITH THE RESPONDENT IS CORRECT:
1) THE ADDRESS, PHONE, AND E-MAIL INFORMATION ON THE PROGRAM ID CARD IS THE SAME AS THE INFORMATION THAT IS ON RECORD.
2) THE PHOTOGRAPH ON THE PROGRAM ID CARD MATCHES THE PHOTOGRAPH ON RECORD.

HOUSEHOLD ADDRESS ______________________________

____

____

____

PRIMARY PHONE NO. OF RESPONDENT ______________________________

ALTERNATE PHONE NO. OF RESPONDENT ______________________________

RESPONDENT E-MAIL ______________________________

PHOTOGRAPH OF THE RESPONDENT ______________________________

SECTION 2: INTRODUCTION

Introduction:

Hello (NAME OF ELIGIBLE WOMAN). My name is _________________ (COUNSELOR NAME) and I am a family health counselor from Innovations for Poverty Action (IPA) Malawi, located in Area 47, Lilongwe. I am pleased to inform you that you have been invited to participate in a maternal health and wellness program that aims to promote access to health services for women. As part of this program, you are eligible to receive a package of health and family planning services over approximately a one month period. These services include:
• One free counseling session with a trained health and family planning counselor

Eligibility:

You are eligible for this program because:
1. You are a married woman;
2. You are between the ages of 18 to 35;
3. You live in Lilongwe;
4. You are currently non-pregnant and did not give birth in the 6 months prior to the initial screening
5. You were recently interviewed by an interviewer from Innovations for Poverty Action (IPA) Malawi on your health and well-being.
6. You have neither been sterilized nor have had a hysterectomy
7. You have given birth to at least one child (one live birth) in your lifetime
8. You were living with their husbands at the time of the screening

Background Information Check:

Before I explain the program and each of these benefits to you in more detail, I would like to confirm some background information about you that you had reported the last time that you had spoken to a member of our team.

4_1	Can you tell me your full name?	FIRST NAME ___________________<br>LAST NAME ___________________	
5_1	Do you know your date of birth?	YES 1<br>NO 2	
5_2	What is your date of birth?		
5-2A	In which year were you born?	[ ][ ][ ][ ]	
5-2B	In which month were you born?	[ ][ ]	
6	How old were you at your last birthday?	[ ][ ]	
6_2	CONFIRM WHETHER THE PRIMARY PHONE NUMBER FOR THE CLIENT IS STILL CORRECT:	YES 1<br>NO 2	→ 6_3
6_3	ENTER NEW PRIMARY PHONE NUMBER: +265	___________________	
6_4	DIRECTIONS TO HOUSEHOLD:<br>CURRENT DIRECTIONS:	___________________	
6_5	COLLECT GPS COORDINATES OF THE HOUSEHOLD:<br>current GPS:	___________________	

COUNSELOR VISITS

	VISIT 1	VISIT 2	VISIT 3	FINAL VISIT
DATE	______	______	______	DAY<br>MONTH<br>YEAR
INTERVIEWER'S NAME	______	______	______	INT. NUMBER
RESULT*	______	______	______	RESULT
NEXT VISIT: DATE<br>TIME	______<br>______	______<br>______		TOTAL NUMBER OF VISITS

*RESULT CODES:

1 COMPLETED
2 NOT AT HOME
3 POSTPONED
4 REFUSED
5 PARTLY COMPLETED
6 INCAPACITATED
7 OTHER ______ (SPECIFY)

COUNSELOR TRACKING FORM: PRE-COUNSELING QUESTIONNAIRE

RECORD THE FOLLOWING INFORMATION BEFORE COUNSELING.

NO.	QUESTIONS AND FILTERS	CODING CATEGORIES	SKIP
101_N	RECORD THE DATE AND TIME.	DAY . . . .<br>MONTH . . . .<br>YEAR . . . .<br>HOUR . . . .<br>MINUTES	

RECORD THE FOLLOWING INFORMATION BEFORE STARTING THE COUNSELING SESSION.

NO.	QUESTIONS AND FILTERS	CODING CATEGORIES	SKIP
104	Now I would like to ask about all the births you have had during your life. Have you ever given birth?	YES . . . . 1<br>NO 2	 → 109
WID_1	Would you like to update information for any of the following? Select the information you would like to update.	NUMBER OF ALIVE SC .... 1<br>NUMBER OF ALIVE DAUGHTI .... 2<br>NUMBER OF NON-HOME S .... 3<br>NUMBER OF NON-HOME DAUGHTE 4<br>NUMBER OF DEAD SC .... 5<br>NUMBER OF DEAD DAUGHTI 6	
UPD_1	Number of alive sons	NUMBEF	
UPD_2	Number of alive daughters	NUMBEF	
UPD_3	Number of non-home sons	NUMBEF	
UPD_4	Number of non-home daughters	NUMBEF	
UPD_5	Number of dead sons	NUMBEF	
UPD_6	Number of dead daughters	NUMBEF	

NO.	QUESTIONS AND FILTERS	CODING CATEGORIES	SKIP
115	CHECK 208:<br><br>Just to make sure that I have this right: you have had in TOTAL _____ births during your life. Is that correct?<br><br>YES NO PROBE AND CORRECT if NECESSARY.		

NO.	QUESTIONS AND FILTERS	CODING CATEGORIES		SKIP
116A	Since June 2019, have you received any family planning counseling at any clinic or hospital?	YES	1	
		NO	2	→ 117
116B	Where did you receive counseling?<br><br>CIRCLE ALL THAT ARE MENTIONED.	HOME	0	
		GOOD HEALTH KAUMA CLINIC	1	
		GOVERNMENT HOSPITAL	11	
		GOVERNMENTAL HEALTH CENTRE	12	
		GOVERNMENT HEALTH POST/OUTI	13	
		PUBLIC MOBILE CLINIC	14	
		HAS	15	
		PUBLIC CBDA/DOOR-TO-DOOR	16	
		OTHER PUBLIC SECTOR	17	
		CHAM HOSPITAL	21	
		CHAM HEALTH CENTRE	22	
		CHAM MOBILE CLINIC	23	
		CHAM DOOR-TO-DOOR	24	
		PRIVATE HOSPITAL/CLINIC	31	
		PHARMACY	32	
		PRIVATE DOCTOR	33	
		PRIVATE MOBILE CLINIC	34	
		PRIVATE CBDA/DOOR-TO-DOOR	35	
		OTHER PRIVATE MEDICAL SECTOF	36	
		BANJA LA MTSOGOLO (BLM) CLINI(	41	
		MACRO CLINIC	51	
		TUNZA (PSI) CLINIC	61	
		YOUTH DROP-IN CENTRE	71	
		SHOP	81	
		CHURCH	82	
		FRIEND/RELATIVE	83	
		OTHER (SPECIFY)	96	
117	Is there any topic(s) that you would like to talk about in greater detail or receive more information on in today's session?<br><br>CIRCLE ALL THAT ARE MENTIONED.	INFO ON PARTICULAR METHODS	11	
		INFO ON PREGNANCY	12	
		INFO ON BIRTH SPACING	13	
		INFO ON SIDE EFFECTS	14	
		INFO ON SERVICE AVAILABILITY	15	
		INFO ON SEXUALLY TRANSMITTED INFECTIONS	16	
		INFO ON PARTNER ENGAGEMENT	17	
		INFO ON CHILD HEALTH	18	
		INFO ON BREASTFEEDING	19	
		OTHER	20	
		NOTHING SPECIFIC / DON'T KNOW	99	

NO.	QUESTIONS AND FILTERS	CODING CATEGORIES	SKIP
118	CHECK FOR PREGNANCY STATUS:<br>Are you currently pregnant?	YES 1<br>NO / UNSURE 2	 → 119
118B	How many months pregnant are you?<br>RECORD ANSWER IN MONTHS.	NUMBER OF MONTHS . . . . . [ ][ ]	
119	CHECK 118:<br>PREGNANT [ ]↓ NOT PREGNANT OR UNSURE [ ] →		121
118B	How many months pregnant are you?<br>RECORD ANSWER IN MONTHS.	[ ][ ]	
120	After the child you are expecting now, would you like to have another child, or would you prefer not to have any more children?	YES . . . . 1<br>NO . . . . 2<br>UNDECIDED . . . . 4<br>UP TO GOI . . . . 5<br>DON'T KNOW 8	→ 122<br><br>→ END (2, 4)
121	Would you like to have another child/more children?	YES . . . . 1<br>NO . . . . 2<br>SAYS SHE CAN'T GET PREGNANT 3<br>UNDECIDED . . . . 4<br>UP TO GOI . . . . 5<br>DON'T KNOW 8	 → 123<br>→ 123<br><br>→ 123 (4, 5)

NO.	QUESTIONS AND FILTERS		CODING CATEGORIES		SKIP
122	CHECK 118:<br><br>NOT PREGNANT OR UNSURE<br><br>How long would you like to wait from now before the birth of (a/another) child?	PREGNANT<br><br>After the birth of the child you are expecting now, how long would you like to wait before the birth of another child?	MONTHS . . . . 1<br>YEARS . . . . 2<br><br>SOON/NOW . . . .<br>SAYS SHE CAN'T GET PREGNANT<br>AFTER MARRIAGE . . . .<br>UP TO GOD . . . .<br><br>OTHER ___________________<br>(SPECIFY)<br>DON'T KNOW . . . .<br>REFUSED	 <br><br>993<br>994<br>995<br>997<br><br>996<br><br>998<br>999	
123	Are you currently using any method of family planning to prevent pregnancy?		YES<br>NO	1<br>2	 → 128C
124	Which method(s) are you currently using?<br><br>CIRCLE ALL MENTIONED.		FEMALE STERILIZATION . . . .<br>MALE STERILIZATION . . . .<br>IUD . . . .<br>INJECTABLES . . . .<br>IMPLANTS . . . .<br>PILL . . . .<br>CONDOM . . . .<br>FEMALE CONDOM . . . .<br>DIAPHRAGM/FOAM/JELLY . . . .<br>TWO DAY METHOD . . . .<br>STANDARD DAYS METHOD . . . .<br>LACTATIONAL AMEN. METHOD . . . . .<br>RHYTHM METHOD . . . .<br>WITHDRAWAL . . . .<br>OTHER MODERN METHOD . . . .<br>OTHER TRADITIONAL METHOD . . .	1<br>2<br>3<br>4<br>5<br>6<br>7<br>8<br>9<br>10<br>11<br>12<br>13<br>14<br>15<br>16	

NO.	QUESTIONS AND FILTERS	CODING CATEGORIES		SKIP
125	What are some of the reasons as to why you chose to use these current family planning method(s)?<br><br>SELECT ALL THAT APPLY.	EFFECTIVE AT PREVENTING PREGNANCY	1	
		DURATION OF EFFECT / LASTS LONG	2	
		NO RISK OF HARMING HEALTH	3	
		NO EFFECT ON REGULAR MONTHLY BLEEDING	4	
		NO UNPLEASANT SIDE EFFECTS	5	
		LOW COST	6	
		NO RISK OF INFERTILITY	7	
		NON-HORMONAL	8	
		NO NEED TO GO TO A CLINIC TO OBTAIN THE METHOD	9	
		IMMEDIATE RETURN TO FERTILITY	10	
		PROTECTS AGAINST STI/HIV	11	
		WANT TO TRY SOMETHING NEW / TIRED OF OLD METHOD	12	
		MY DOCTOR RECOMMENDED IT TO ME	13	
		MY HUSBAND WANTED ME TO USE THIS METHOD	14	
		OTHER WOMEN IN MY FAMILY HAVE USED THIS METHOD	15	
		FRIENDS HAVE USED THIS METHOD	16	
		EASILY AVAILABLE AT CLINIC	17	
		NO NEED TO REMEMBER USING THE METHOD	18	
		CAN BE USED FOR A LONG TIME WITHOUT NEED TO VISIT CLINIC OR RE-SUPPLY	19	
		CAN BE USED WITHOUT ANYONE ELSE KNOWING	20	
		DOES NOT INTERRUPT SEX	21	
		OTHER . (SPECIFY)	96	
		DON'T KNOW .	88	
		REFUSED . .	99	
126	If you had the choice and ability to switch to another family planning method, would you choose to switch?	YES	1	
		NO	2	→ 130

NO.	QUESTIONS AND FILTERS	CODING CATEGORIES	SKIP
127	Which method(s) would you want to switch to?<br><br>CIRCLE ALL MENTIONED.	FEMALE STERILIZATION . . . . 1<br>MALE STERILIZATION . . . . 2<br>IUD . . . . 3<br>INJECTABLES . . . . 4<br>IMPLANTS . . . . 5<br>PILL . . . . 6<br>CONDOM . . . . 7<br>FEMALE CONDOM . . . . 8<br>DIAPHRAGM/FOAM/JELLY . . . . 9<br>TWO DAY METHOD . . . . 10<br>STANDARD DAYS METHOD . . . . 11<br>LACTATIONAL AMEN. METHOD . . . . . 12<br>RHYTHM METHOD . . . . 13<br>WITHDRAWAL . . . . 14<br>OTHER MODERN METHOD . . . . 15<br>OTHER TRADITIONAL METHOD . . . 16	
128	Why would you want to switch to this method?<br><br>SELECT ALL THAT APPLY.	EFFECTIVE AT PREVENTING PREGNANCY 1<br>DURATION OF EFFECT / LASTS LONG 2<br>NO RISK OF HARMING HEALTH<br>NO EFFECT ON REGULAR MONTHLY BLEEDING 3<br>NO UNPLEASANT SIDE EFFECTS 4<br>LOW COST 5<br>NO RISK OF INFERTILITY 6<br>NON-HORMONAL 7<br>NO NEED TO GO TO A CLINIC<br>TO OBTAIN THE METHOD 8<br>IMMEDIATE RETURN TO FERTILITY 9<br>PROTECTS AGAINST STI/HIV 10<br>WANT TO TRY SOMETHING NEW / TIRED OF OLD M 11<br>MY DOCTOR RECOMMENDED IT TO ME 12<br>MY HUSBAND WANTED ME TO USE THIS METHOD 13<br>OTHER WOMEN IN MY FAMILY HAVE<br>USED THIS METHOD 14<br>FRIENDS HAVE USED THIS METHOD 15<br>EASILY AVAILABLE AT CLINIC 16<br>NO NEED TO REMEMBER USING THE METHOD 17<br>CAN BE USED FOR A LONG TIME WITHOUT<br>NEED TO VISIT CLINIC OR RE-SUPPLY 18<br>CAN BE USED WITHOUT ANYONE<br>ELSE KNOWING 19<br>DOES NOT INTERRUPT SEX 20<br>OTHER ____________________ 96<br>(SPECIFY)<br>DON'T KNOW . . . . 88<br>REFUSED 99	

NO.	QUESTIONS AND FILTERS	CODING CATEGORIES		SKIP
128B	Why have you not yet switched to this method?	NOT MARRIED	1	
		NOT HAVING SEX	2	
		INFREQUENT SEX	3	
		MENOPAUSAL/HYSTERECTOMY	4	
		CAN'T GET PREGNANT	5	
		NOT MENSTRUATED SINCE LAST B	6	
		BREASTFEEDING	7	
		UP TO GOD/FATALISTIC	8	
		HUSBAND/PARTNER OPPOSED	9	
		DOCTOR / PROVIDER OPPOSED	10	
		OTHERS OPPOSED	11	
		RELIGIOUS PROHIBITION	12	
		SOCIAL PRESSURE	13	
		DOES NOT KNOW ENOUGH ABOUT THE METHOD	14	
		KNOWS NO SOURCE	15	
		HARD TO GET TO CLINIC/TOO FAR	16	
		LONG WAITING TIME AT CLINIC	17	
		TOO BUSY/NO TIME	18	FOR ALL OPTIONS SKIP TO 116
		COSTS TOO MUCH	19	
		PREFERRED METHOD NOT AVAILA	20	
		NO METHOD AVAILABLE	21	
		INCONVENIENT TO USE	22	
		DOES NOT TRUST THE CLINIC / PROVIDER	23	
		INTERFERES WITH BODY'S NORMAL PROCESSES	24	
		NOT EFFECTIVE	25	
		FEAR OF INFERTILITY	26	
		FEAR OF SIDE EFFECTS	27	
		OTHER ____________________ (SPECIFY)	96	
		DON'T KNOW	88	
		REFUSED	99	

NO.	QUESTIONS AND FILTERS	CODING CATEGORIES		SKIP
128C	Check pregnancy status: not pregnant<br><br>Can you tell me why you are not using a method to prevent pregnancy?<br><br>RECORD ALL REASONS MENTIONED.	FERTILITY-RELATED REASONS		
		NOT HAVING SEX	2	
		INFREQUENT SEX	3	
		MENOPAUSAL/HYSTERECTOMY	4	
		CAN'T GET PREGNANT	5	
		NOT MENSTRUATED SINCE LAST BIRTH	6	
		BREASTFEEDING	7	
		UP TO GOD/FATALIST	8	
		OPPOSITION TO USE		
		RESPONDENT OPPOSED	9	
		HUSBAND/PARTNER OPPOSED . . .	10	
		OTHERS OPPOSED	11	
		RELIGIOUS PROHIBITION	12	
		SOCIAL PRESSURE	13	
		LACK OF KNOWLEDGE		
		KNOWS NO METHOD	14	
		KNOWS NO SOURCE	15	
		METHOD-RELATED REASONS		
		FEAR OF INFERTILITY	16	
		FEAR OF SIDE EFFECTS	17	
		INTERFERES WITH BODY'S NORMAL PROCESSES	18	
		OTHER HEALTH CONCERNS	19	
		LACK OF ACCESS/TOO FAR	20	
		LONG WAITING TIME	21	
		TOO BUSY/NO TIME	22	
		COSTS TOO MUCH	23	
		PREFERRED METHOD NOT AVAILABLE	24	
		NO METHOD AVAILABLE	25	
		INCONVENIENT TO USE	26	
		NOT EFFECTIVE	27	
		OTHER ____________________ (SPECIFY)	96	
		DON'T KNOW	88	

NO.	QUESTIONS AND FILTERS	CODING CATEGORIES		SKIP
128D	Do you think you will use a contraceptive method to delay or avoid pregnancy at any time in the future?	YES	1	→ 129
		NO	2	
		DON'T KNOW	88	→ 129
128E	Which of the following are reasons why you don't think that you will use a contraceptive method in the future?<br><br>RECORD ALL REASONS MENTIONED.	FERTILITY-RELATED REASONS		
		WANTS ANOTHER CHILD SO . . .	1	
		NOT HAVING SEX	2	
		INFREQUENT SEX	3	
		MENOPAUSAL/HYSTERECTOMY	4	
		CAN'T GET PREGNAN	5	
		NOT MENSTRUATED SINCE LAST BIRTH	6	
		BREASTFEEDING	7	
		UP TO GOD/FATALIST	8	
		OPPOSITION TO USE		
		RESPONDENT OPPOSED	9	
		HUSBAND/PARTNER OPPOS . . .	10	
		OTHERS OPPOSED	11	
		RELIGIOUS PROHIBITIO	12	
		SOCIAL PRESSUR	13	
		LACK OF KNOWLEDGE		
		KNOWS NO METHO	14	
		KNOWS NO SOURC	15	
		METHOD-RELATED REASONS		
		FEAR OF INFERTILITY	16	
		FEAR OF SIDE EFFECTS	17	
		INTERFERES WITH BODY'S NORMAL PROCESSES	18	
		OTHER HEALTH CONCE	19	
		LACK OF ACCESS/TOO FA	20	
		LONG WAITING TIME	21	
		TOO BUSY/NO TIME	22	
		COSTS TOO MUCH	23	
		PREFERRED METHOD NOT AVAILABLE	24	
		NO METHOD AVAILABLE	25	
		INCONVENIENT TO USE	26	
		NOT EFFECTIV	27	
		OTHER ____________________ (SPECIFY)	96	
		DON'T KNOW	88	

NO.	QUESTIONS AND FILTERS	CODING CATEGORIES		SKIP
129	If you could choose any contraceptive method that you want today, which method would you want to use?<br>CIRCLE ALL MENTIONED.	FEMALE STERILIZATION . . . .<br>MALE STERILIZATION . . . .<br>IUD . . . .<br>INJECTABLES . . . .<br>IMPLANTS . . . .<br>PILL . . . .<br>CONDOM . . . .<br>FEMALE CONDOM . . . .<br>DIAPHRAGM/FOAM/JELLY . . . .<br>TWO DAY METHOD . . . .<br>STANDARD DAYS METHOD . . . .<br>LACTATIONAL AMEN. METHOD . . . . .<br>RHYTHM METHOD . . . .<br>WITHDRAWAL . . . .<br>OTHER MODERN METHOD . . . .<br>OTHER TRADITIONAL METHOD . . .	1<br>2<br>3<br>4<br>5<br>6<br>7<br>8<br>9<br>10<br>11<br>12<br>13<br>14<br>15<br>16	
129A	CHECK IF IDEAL_PRE_COUNSELING IDEAL METHOD IS DIFFERENT FROM THE BASAELINE IDEAL METHOD:<br>In the baseline survey we did with you a month ago, you had mentioned that AN IDEAL CONTRACEPTION METHOD IS: (METHOD FROM BASELINE). This time, you mentioned that (METHOD FROM 129) IS YOUR IDEAL METHOD. Have you changed your mind?	YES, CHANGED MIND . . . .<br><br>NO, DID NOT CHANGE MIND	1<br><br>2	
129B	Why did you change your mind?<br>RECORD ALL REASONS MENTIONED.	FORGOT WHAT SHE SAID EARLIER<br>MORE INFORMATION FROM PROVIDER<br>MORE INFORMATION FROM FRIENDS<br>MORE INFORMATION FROM HUSBAND<br>MORE INFORMATION FROM RELATIVES<br>MORE INFORMATION FROM OTHERS<br>LEARED FROM OTHERS' EXPERIENCE<br>MORE INFORMATION FROM COUNSELOR<br>OTHER<br>DON'T KNOW<br>REFUSED<br>PLEASE SPECIFY:	1<br>2<br>3<br>4<br>5<br>6<br>7<br>8<br>96<br>88<br>99	

NO.	QUESTIONS AND FILTERS	CODING CATEGORIES		SKIP
130	In choosing a contraceptive method, what feature would be most important to you?<br><br>SELECT THE MOST IMPORTANT ONE.	EFFECTIVE AT PREVENTING PREGNANCY	1	
		DURATION OF EFFECT / LASTS LONG	2	
		NO RISK OF HARMING HEALTH	3	
		NO EFFECT ON REGULAR MONTHLY BLEEDING	4	
		NO UNPLEASANT SIDE EFFECTS	5	
		LOW COST	6	
		NO RISK OF INFERTILITY	7	
		NON-HORMONAL	8	
		NO NEED TO GO TO A CLINIC TO OBTAIN THE METHOD	9	
		IMMEDIATE RETURN TO FERTILITY	10	
		PROTECTS AGAINST STI/HIV	11	
		WANT TO TRY SOMETHING NEW / TIRED OF OLD METHOD	12	
		MY DOCTOR RECOMMENDED IT TO ME	13	
		MY HUSBAND WANTED ME TO USE THIS METHOD	14	
		OTHER WOMEN IN MY FAMILY HAVE USED THIS METHOD	15	
		FRIENDS HAVE USED THIS METHOD	16	
		EASILY AVAILABLE AT CLINIC	17	
		NO NEED TO REMEMBER USING THE METHOD	18	
		CAN BE USED FOR A LONG TIME WITHOUT NEED TO VISIT CLINIC OR RE-SUPPLY	19	
		CAN BE USED WITHOUT ANYONE ELSE KNOWING	20	
		DOES NOT INTERRUPT SEX	21	
		OTHER ______________________ (SPECIFY)	96	
		DON'T KNOW	88	
		REFUSED	99	
	CHECK 130 WITH 331, 331B, AND 332 FROM BASELINE:<br><br>TOP CHOICE DOES NOT MATCH ☐ ↓ TOP CHOICE MATCHES ☐ →			INTRO

NO.	QUESTIONS AND FILTERS	CODING CATEGORIES	SKIP
131	The last time you had an interview, you had mentioned that (BASELINE FEATURE) was most important to you. This time, you mentioned that (FEATURE IN 130) is most important to you. Have you changed your mind from (BASELINE FEATURE) to (FEATURE IN 130)?	YES, CHANGED MIND . . . . 1<br>NO, DID NOT CHANGE MIN 2	 → INTRO
132	Why did you change your mind on your most valued attribute?<br>RECORD ALL REASONS MENTIONED.	FORGOT WHAT SHE SAID EARLIER 1<br>MORE INFORMATION FROM PROVIDER 2<br>MORE INFORMATION FROM FRIENDS 3<br>MORE INFORMATION FROM HUSBAND 4<br>MORE INFORMATION FROM RELATIVES 5<br>MORE INFORMATION FROM OTHERS 6<br>LEARED FROM OTHERS' EXPERIEN 7<br>OTHER 96<br>DON'T KNOW 88<br>REFUSED 99<br>PLEASE SPECIFY:	
133	FINAL ATTRIBUTE RANKING:<br><br>IF RESPONDENT **DID NOT CHANGE** THEIR FIRST CHOICE FROM BASELINE (EITHER ACTIVELY DID NOT CHANGE, OR CHANGED CHOICE NOW BUT DID NOT CONFIRM CHANGE WHEN ASKED NOW), RANKING IS: **1) FIRST CHOICE FROM BASELINE, 2) SECOND CHOICE FROM BASELINE, 3) THIRD CHOICE FROM BASELINE.**<br><br>IF RESPONDENT **ACTIVELY CHANGED** THEIR FIRST CHOICE FROM BASELINE TO NOW, RANKING IS: **1) FIRST CHOICE NOW, 2) FIRST CHOICE FROM BASELINE, 3) SECOND CHOICE FROM BASELINE**	RANK ATTRIBUTE<br>1 [__][__]<br>2 [__][__]<br>3 [__][__]	
134	Which attibute's rank is not correct?	FIRST ATTRIBUTE 1<br>SECOND ATTRIBUTE 2<br>THIRD ATTRIBUTE 3	

COUNSELOR TRACKING FORM: COUNSELING COMPONENT

**RECORD THE FOLLOWING INFORMATION ONCE THE PRE-COUNSELING INFORMATION HAS BEEN RECORDED.**			
NO.	QUESTIONS AND FILTERS	CODING CATEGORIES	SKIP
201	RECORD THE DATE AND TIME.	DAY . . . . [ ][ ]<br>MONTH . . . . [ ][ ]<br>YEAR . . . . [ ][ ][ ][ ]<br>HOUR . . . . [ ][ ]<br>MINUTES . . . . [ ][ ]	
**RECORD THE FOLLOWING INFORMATION BEFORE STARTING THE COUNSELING SESSION.**			
202	RECORD TREATMENT GROUP STATUS:	1: LONG COUNSELING, NO HUSBAN 1<br>2: LONG COUNSELING, HUSBAND 2<br>3: SHORT COUNSELING, NO HUSBA 3<br>4: SHORT COUNSELING, HUSBAND 4	

NO.	QUESTIONS AND FILTERS	CODING CATEGORIES	SKIP

INTRODUCTION:

Thank you for this information. I would now like to explain the services that are available to you as part of this maternal health and wellness program.

As part of this program, you are eligible to receive one free counseling session today. In this session, you will discuss different topics related to your health and well-being and the role that family planning can play to help you achieve your ideal family goals. Some topics that will be discussed in these sessions include:
• your fertility preferences and goals
• how you and your husband / partner can healthily plan for your next birth together
• the benefits of family planning and reproductive health for women like you

This session will provide you with an opportunity to share your experiences and to ask any questions that you may have about your pregnancy, childbirth, and family planning.

IF IN TREATMENT GROUPS 1 AND 3:

Husband Invitation
In addition, you are welcome to invite your husband to this counseling session if you choose. It is your choice as to whether or not you would like to invite your husband to the counseling session.

IF IN TREATMENT GROUPS 0 AND 1
Key Terms and Conditions
1. This counseling session will last between 45 minutes and one hour.
2. You may choose to end this counseling session at any time, and you may also choose to end your participation in this counseling service at any time.
3. You will receive a phone call from a counselor two days before a scheduled session to confirm the date and time of your appointment.
4. You will be able to reschedule your appointment to a time that is convenient for you.
5. The counseling session will be held in a private location here in your house, and all discussions between you and the counselor will be kept confidential.
6. This counseling service is meant mainly for your personal and private use only. If you choose to invite your husband, the counselor will counsel you together at the same time.
7. At the end of the counseling session, the counselor will ask you some questions about the session and will gather your feedback and experience with the service that was provided. Participation in this short survey is voluntary, and you may refuse to participate or end your participation at any time without penalty. Your responses to the survey questions will be kept confidential, and your name and other identifying information will always be kept anonymous.
8. If you choose to invite your husband, the counselor will ask him some questions at the end of the counseling session and will gather his feedback and experience with the service that was provided.

NO.	QUESTIONS AND FILTERS	CODING CATEGORIES	SKIP
203	Would you like to participate in the counseling session now?	YES . . . . 1<br>NO 2	1 → 206
206	CHECK TREATMENT STATUS<br>IS THE WOMAN IN TREATMENT GROUP T1 OR T3?	YES . . . . 1<br>NO 2	2 → 209
207	As I mentioned, you have the opportunity to invite your husband to the counseling session. Would you like to invite him to receive counseling with you?	YES . . . . 1<br>NO 2	2 → 209N
209N	Could you tell me why you do not want to invite your husband to participate?	HUSBAND NOT AT HOME . . . . 1<br>HE DOESN'T WANT TO PARTICIPATE . . . . 2<br>I DON'T WANT TO HIM TO RECEIVE COUNSELING WITH ME . . . . 3<br>HUSBAND ONLY AVAILABLE . . . . . 4<br>AT NITHGTIME	
208	Thank you. I will counsel you both together. Before I begin the counseling, I would like to ask your husband a few questions about his health, family preferences, and use of family planning. These questions will take about 10 minutes.<br>.		GO TO HUSB FORM
	MAKE SURE THE WOMAN IS NOT PRESENT DURING THIS PRE-COUNSELING SECTION WITH HUSBAND<br>**WAIT FOR HUSBAND. ADMINISTER HUSBAND CONSENT FORM**	OK 1	
	ASK HUSBAND:<br>Do you consent to participate in the study?	YES . . . . 1<br>NO 2	HUSB PRE COUN FORM<br>2 → HUSB END
HUSB_END	Thank you for your time. I hope that you have a good day. Please feel free to contact me at any time if you have any questions or if you change your mind.<br>Do you mind if I re-confirm some information with your wife? Please ask the woman to come back.	YES . . . . 1<br>NO 2	1 → HUSB wom Check<br>2 → HUSB RESCHE
HUSB_RESCH	Thank you. Hope you have a great day.<br>**SAVE FORM AS INCOMPLETE. SCHEDULE ANOTHER TIME WITH THE WOMAN TO ADMINISTER THE COUNSELING ALONE.**	OK 1	→ END
HUSB_wom Check	**WAIT FOR THE WOMAN.**<br>**ASK THE WOMAN SECRETLY FOR HER AVAILABILITY FOR THE COUNSELING SESSION.**<br>Do you want to receive the counseling session by yourself today? Or do you want to schedule another date for the counseling session?	RECEIVE THE COUNSELING TODAY . . . . . 1<br>RE-SCHEDULE THE COUNSELING TO ANOTHER DATE 2	1 → 209<br>2 → WOM COUN_RESCH DT
WOM COUN_RESCH DT	When would you like me to return for the counseling session?<br>RECORD THE DATE AND TIME.<br>SAVE FORM AS INCOMPLETE.<br>RETURN ON THE DATE AND TIME RECORDED TO COMPLETE THE FORM AND COUNSELING SESSION.	DAY . . . . [ ][ ]<br>MONTH . . . . [ ][ ]<br>YEAR . . . . [ ][ ][ ][ ]<br>HOUR . . . . [ ][ ]<br>MINUTES . . . . [ ][ ]	SAVE AS INCOM-PLETE RETURN LATER.

NO.	QUESTIONS AND FILTERS	CODING CATEGORIES	SKIP
209	Thank you. I would now like to administer the counseling session with you.<br><br>Do you have any questions for me before I begin?<br>IF YES, ANSWER ANY QUESTIONS.	OK 1	
HUSB COUN_ cons	Do you consent to receive the counseling?	YES . . . . 1<br>NO 2	HUSB PRE COUN FORM<br>→ HUSB END
	Do you consent to receive the counseling today?	YES . . . . 1<br>NO 2	→ 210<br>→ END
209_CB_Q	I understand. Would you like for me to come back at a later time to participate in the counseling session, or are you not at all interested in this opportunity?	YES . . . . 1<br>NO 2	
209_CB_Q DT	When would you like me to return for the counseling session?<br><br>RECORD THE DATE AND TIME.<br><br>SAVE FORM AS INCOMPLETE.<br>RETURN ON THE DATE AND TIME RECORDED TO COMPLETE THE FORM AND COUNSELING SESSION.	DAY . . . . [ ][ ]<br>MONTH . . . . [ ][ ]<br>YEAR . . . . [ ][ ][ ][ ]<br>HOUR . . . . [ ][ ]<br>MINUTES . . . . [ ][ ]	SAVE AS INCOM-PLETE RETURN LATER.
210	**CHECK 133 AND TREATMENT STATUS:**<br><br>**IF WOMAN IS IN TREATMENT GROUPS 1 OR 2, ADMINISTER FULL (GREEN) COUNSELING FLIPCHART.**<br><br>**IF WOMAN IS IN TREATMENT GROUPS 3 OR 4, THEN BASED ON 119, ADMIINSTER**<br><br>**FLIPCHART COLORS (BASED ON ATTRIBUTES RANKING FROM 133):**<br><br>**FULL FLIPCHART: GREEN (FOR TREATMENT GROUPS 1 AND 2 ONLY)**<br>ATTRIBUTE 1: BLUE ATTRIBUTE 5: PURPLE<br>ATTRIBUTE 2: RED ATTRIBUTE 6: BLACK<br>ATTRIBUTE 3: YELLOW ATTRIBUTE 7: WHITE<br>ATTRIBUTE 4: ORANGE ATTRIBUTE 8: BROWN		
211	**COUNSELING:**<br>COMPLETE COUNSELING FOR WOMEN (AND FOR HUSBANDS, IF APPLICABLE) USING CORRECT COUNSELING FLIPCHART. ANSWER ANY QUESTIONS OR CONCERNS THAT THE WOMAN / COUPLE HAV		GO TO POST-COUNS
COMP CONF	To counselors: Confirm you have completed the counseling session using the right flipchart.	OK 1	
END<br><br>212	Thank you for your time. I hope that you have a good day.<br><br>END FORM.	END FORM.	

COUNSELOR TRACKING FORM: HUSBAND PRE-COUNSELING QUESTIONNAIRE

NO.	QUESTIONS AND FILTERS	CODING CATEGORIES	SKIP
**RECORD THE FOLLOWING INFORMATION BEFORE COUNSELING.**			
101	RECORD THE DATE AND TIME.	DAY . . . . [ ][ ]<br>MONTH . . . . [ ][ ]<br>YEAR . . . . [ ][ ][ ][ ]<br>HOUR . . . . [ ][ ]<br>MINUTES . . . . [ ][ ]	
**RECORD THE FOLLOWING INFORMATION BEFORE STARTING THE COUNSELING SESSION.**			
102A	Do you know your date of birth?	YES . . . . 1<br>NO 2	 → 102C
102B	What is your date of birth?	HUSBAND'S DATE OF BIRTH ________	
102C	In what month and year were you born?	MONTH . . . . [ ][ ]<br>DON'T KNOW MONTH . . . . 98<br>YEAR . . . . [ ][ ][ ][ ]<br>DON'T KNOW YEAR 9998	
103	How old were you at your last birthday?<br><br>COMPARE AND CORRECT 102 AND/OR 103 IF INCONSISTENT.	AGE IN COMPLETED YEARS [ ][ ]	
104A	Have you ever attended school?	YES . . . . 1<br>NO . . . . 2<br>DON'T KNOW . . . . 88<br>REFUSED 99	→ 104B
104B	What was the highest level of school you attended: primary, secondary, or higher? **(1)**	PRE-PRIMARY 0<br>PRIMARY . . . . 1<br>SECONDARY . . . . 2<br>HIGHER . . . . 3<br>DON'T KNOW 88	 <br><br><br>→ 106
105	What was the highest (class/form/year) you completed at that level? **(1)**<br><br>IF COMPLETED LESS THAN ONE YEAR AT THAT LEVEL, RECORD '00'.	CLASS . . . . [ ][ ]<br>DON'T KNOW 88	

NO.	QUESTIONS AND FILTERS	CODING CATEGORIES	SKIP
106	As you know, some men take up jobs for which they are paid in cash or kind. Others sell things, have a small business or work on the family farm or in the family business. Do you do any of these things or any other work in the last seven days?	YES . . . . 1<br>NO . . . . 2<br>DON'T KNOW . . . . 88<br>REFUSED 99	(YES) → 108B
107	Although you did not work in the last seven days, do you have any job or business from which you were absent for leave, illness, vacation, maternity leave or any other such reason?	YES . . . . 1<br>NO . . . . 2<br>DON'T KNOW . . . . 88<br>REFUSED 99	(YES) → 108B
108A	Have you done any work in the last 12 months?	YES . . . . 1<br>NO . . . . 2<br>DON'T KNOW . . . . 88<br>REFUSED 99	(NO, DON'T KNOW, REFUSED) → 110
108B	Do you do this work for a member of your family, for someone else, or are you self-employed?	FOR FAMILY MEMBER . . . . 1<br>FOR SOMEONE ELSE . . . . 2<br>SELF-EMPLOYED 3	
**109**	Are you paid in cash or kind for this work, or are you not paid at all?	CASH ONLY . . . . 1<br>CASH AND KIND . . . . 2<br>IN KIND ONLY . . . . 3<br>NOT PAID 4	

NO.	QUESTIONS AND FILTERS	CODING CATEGORIES	SKIP
110	Since June 2019, have you received any family planning counseling at any clinic or hospital?	YES 1<br>NO 2	 → 112
111	Where did you receive counseling?<br><br>CIRCLE ALL THAT ARE MENTIONED.	GOOD HEALTH KAUMA CLINIC 1<br>OTHER TUNZA CLINIC 2<br>BLM CLINIC 3<br>OTHER CLINIC / HOSPITAL . 4<br>MOBILE CLINIC 5<br>HSA 6<br>CBDA / DOOR-TO-DOOR 7<br>DON'T KNOW 9	
112	Is there any topic(s) that you would like to talk about in greater detail or receive more information on in today's session?<br><br>CIRCLE ALL THAT ARE MENTIONED.	INFO ON PARTICULAR METHODS 11<br>INFO ON PREGNANCY 12<br>INFO ON BIRTH SPACING 13<br>INFO ON SIDE EFFECTS 14<br>INFO ON SERVICE AVAILABILITY 15<br>INFO ON STI 16<br>INFO ON PARTNER ENGAGEMENT 17<br>INFO ON CHILD HEALTH 18<br>INFO ON BREASTFEEDING 19<br>OTHER 20<br>NOTHING SPECIFIC / DON'T KNOW 99	
113	Husband Fertility Preferences:<br><br>Would you like to have another child/more children?	YES . . . . 1<br>NO . . . . 2<br><br>UNDECIDED . . . . 3<br>UP TO GOI . . . . 4<br>DON'T KNOW 88	 → 123<br>→ 123<br><br>→ 123
113A	How many more children do you want to have?	NUMBER OF CHILDREN ☐☐	
113B	How many of them do you want to be boys?	NUMBER OF BOYS ☐☐	
113C	How many of them do you want to be girls?	NUMBER OF GIRLS ☐☐	
113D	SUM ANSWERS TO 922 AND 923, AND ENTER TOTAL.<br>IF NONE, RECORD '00'.	TOTAL DESIRED BIRTHS ☐☐	
113E	Does your wife want more or fewer children than you do?	SAME NUMBER . . . . 1<br>MORE CHILDREN . . . . 2<br>FEWER CHILDRE . . . . 3<br>DON'T KNOW . . . . 88<br>REFUSED 98	

NO.	QUESTIONS AND FILTERS	CODING CATEGORIES		SKIP
114	Are you currently using any method of family planning to prevent pregnancy?	YES<br>NO	1<br>2	 → 121
115	Which method(s) are you currently using?<br><br>CIRCLE ALL MENTIONED.	MALE STERILIZATION . . . .<br>CONDOM . . . .<br>WITHDRAWAL . . . .<br>OTHER MODERN METHOD . . . .<br>OTHER TRADITIONAL METHOD . . .	2<br>7<br>14<br>15<br>16	
116	What are some of the reasons as to why you chose to use these current family planning method(s)?<br><br>SELECT ALL THAT APPLY.	EFFECTIVE AT PREVENTING PREGNANCY<br>DURATION OF EFFECT / LASTS LONG<br>NO RISK OF HARMING HEALTH<br>NO EFFECT ON REGULAR MONTHLY BLEEDING<br>NO UNPLEASANT SIDE EFFECTS<br>LOW COST<br>NO RISK OF INFERTILITY<br>NON-HORMONAL<br>NO NEED TO GO TO A CLINIC TO OBTAIN THE METHOD<br>IMMEDIATE RETURN TO FERTILITY<br>PROTECTS AGAINST STI/HIV<br>WANT TO TRY SOMETHING NEW / TIRED OF OLD METHOD<br>MY DOCTOR RECOMMENDED IT TO ME<br>MY WIFE WANTED ME TO USE THIS METHOD<br>OTHER WOMEN IN MY FAMILY HAVE USED THIS METHOD<br>FRIENDS HAVE USED THIS METHOD<br>EASILY AVAILABLE AT CLINIC<br>NO NEED TO REMEMBER USING THE METHOD<br>CAN BE USED FOR A LONG TIME WITHOUT NEED<br>TO VISIT CLINIC OR RE-SUPPLY<br>CAN BE USED WITHOUT ANYONE ELSE KNOWING<br>DOES NOT INTERRUPT SEX<br>OTHER .<br>(SPECIFY)<br>DON'T KNOW .<br>REFUSED . .	1<br>2<br>3<br>4<br>5<br>6<br>7<br>8<br>9<br>10<br>11<br>12<br>13<br>14<br>15<br>16<br>17<br>18<br>19<br><br>20<br>21<br>96<br><br>88<br>99	

NO.	QUESTIONS AND FILTERS	CODING CATEGORIES		SKIP
117	If you had the choice and ability to switch to another family planning method, would you choose to switch?	YES . . . .<br>NO	1<br>2	 → 130
118	Which method(s) would you want to switch to?<br><br>CIRCLE ALL MENTIONED.	MALE STERILIZATION . . . .<br>CONDOM . . . .<br>WITHDRAWAL . . . .<br>OTHER MODERN METHOD . . . .<br>OTHER TRADITIONAL METHOD . . .	2<br>7<br>14<br>15<br>16	
119	Why would you want to switch to this method?<br><br>SELECT ALL THAT APPLY.	FORGOT WHAT SHE SAID EARLIE<br>EFFECTIVE AT PREVENTING PREGNANCY<br>DURATION OF EFFECT / LASTS LONG<br>NO RISK OF HARMING HEALTH<br>NO EFFECT ON REGULAR MONTHLY BLEEDING<br>NO UNPLEASANT SIDE EFFECTS<br>LOW COST<br>NO RISK OF INFERTILITY<br>NON-HORMONAL<br>NO NEED TO GO TO A CLINIC<br>TO OBTAIN THE METHOD<br>IMMEDIATE RETURN TO FERTILITY<br>PROTECTS AGAINST STI/HIV<br>WANT TO TRY SOMETHING NEW / TIRED OF OLD M<br>MY DOCTOR RECOMMENDED IT TO ME<br>MY WIFE WANTED ME TO USE THIS METHOD<br>OTHER WOMEN IN MY FAMILY HAVE<br>USED THIS METHOD<br>FRIENDS HAVE USED THIS METHOD<br>EASILY AVAILABLE AT CLINIC<br>NO NEED TO REMEMBER USING THE METHOD<br>CAN BE USED FOR A LONG TIME WITHOUT<br>NEED TO VISIT CLINIC OR RE-SUPPLY<br>CAN BE USED WITHOUT ANYONE<br>ELSE KNOWING<br>DOES NOT INTERRUPT SEX<br>OTHER ________________<br>(SPECIFY)<br>DON'T KNOW . . . .<br>REFUSED	0<br>1<br>2<br><br>3<br>4<br>5<br>6<br>7<br><br>8<br>9<br>10<br>11<br>12<br>13<br><br>14<br>15<br>16<br>17<br><br>18<br><br>19<br>20<br>96<br><br>88<br>99	

NO.	QUESTIONS AND FILTERS	CODING CATEGORIES		SKIP
120	Why have you not yet switched to this method?	NOT MARRIED	1	FOR ALL OPTION S SKIP TO 122
		NOT HAVING SEX	2	
		INFREQUENT SEX	3	
		MENOPAUSAL/HYSTERECTOMY	4	
		CAN'T GET PREGNANT	5	
		NOT MENSTRUATED SINCE LAST B	6	
		BREASTFEEDING	7	
		UP TO GOD/FATALISTIC	8	
		WIFE/PARTNER OPPOSED	9	
		DOCTOR / PROVIDER OPPOSED	10	
		OTHERS OPPOSED	11	
		RELIGIOUS PROHIBITION	12	
		SOCIAL PRESSURE	13	
		DOES NOT KNOW ENOUGH ABOUT THE METHOD	14	
		KNOWS NO SOURCE	15	
		HARD TO GET TO CLINIC/TOO FAR	16	
		LONG WAITING TIME AT CLINIC	17	
		TOO BUSY/NO TIME	18	
		COSTS TOO MUCH	19	
		PREFERRED METHOD NOT AVAILA	20	
		NO METHOD AVAILABLE	21	
		INCONVENIENT TO USE	22	
		DOES NOT TRUST THE CLINIC / PROVIDER	23	
		INTERFERES WITH BODY'S NORMAL PROCESSES	24	
		NOT EFFECTIVE	25	
		FEAR OF INFERTILITY	26	
		FEAR OF SIDE EFFECTS	27	
		OTHER ____________________ (SPECIFY)	96	
		DON'T KNOW	88	
		REFUSED	99	

NO.	QUESTIONS AND FILTERS	CODING CATEGORIES		SKIP
120A	Check pregnancy status: not pregnant<br><br>Can you tell me why you are not using a method to prevent pregnancy?<br><br>RECORD ALL REASONS MENTIONED.	FERTILITY-RELATED REASONS		
		NOT HAVING SEX	2	
		INFREQUENT SEX	3	
		MENOPAUSAL/HYSTERECTOMY	4	
		CAN'T GET PREGNAN	5	
		NOT MENSTRUATED SINCE<br>LAST BIRTH	6	
		BREASTFEEDING	7	
		UP TO GOD/FATALIST	8	
		OPPOSITION TO USE		
		RESPONDENT OPPOSE	9	
		HUSBAND/PARTNER OPPOS . . .	10	
		OTHERS OPPOSED	11	
		RELIGIOUS PROHIBITIO	12	
		SOCIAL PRESSUR	13	
		LACK OF KNOWLEDGE		
		KNOWS NO METHO	14	
		KNOWS NO SOURC	15	
		METHOD-RELATED REASONS		
		FEAR OF INFERTILITY	16	
		FEAR OF SIDE EFFECTS	17	
		INTERFERES WITH BODY'S<br>NORMAL PROCESSES	18	
		OTHER HEALTH CONCE	19	
		LACK OF ACCESS/TOO FA	20	
		LONG WAITING TIME	21	
		TOO BUSY/NO TIME	22	
		COSTS TOO MUCH	23	
		PREFERRED METHOD<br>NOT AVAILABL	24	
		NO METHOD AVAILABLE	25	
		INCONVENIENT TO USE	26	
		NOT EFFECTIV	27	
		OTHER ____________________<br>(SPECIFY)	96	
		DON'T KNOW	88	
120B	Do you think you will use a contraceptive method to delay or avoid pregnancy at any time in the future?	YES	1	→ 129
		NO	2	
		DON'T KNOW	88	→ 129

NO.	QUESTIONS AND FILTERS	CODING CATEGORIES	SKIP
120C	Which of the following are reasons why you don't think that you will use a contraceptive method in the future?<br><br>RECORD ALL REASONS MENTIONED.	FERTILITY-RELATED REASONS<br>WANTS ANOTHER CHILD SO . . . 1<br>NOT HAVING SEX . . . . 2<br>INFREQUENT SE) . . . . 3<br>MENOPAUSAL/HYSTERECTOMY 4<br>CAN'T GET PREGNAN . . . . 5<br>NOT MENSTRUATED SINCE<br>LAST BIRTH . . . . 6<br>BREASTFEEDING . . . . 7<br>UP TO GOD/FATALIST . . . . 8<br><br>OPPOSITION TO USE<br>RESPONDENT OPPOSEI . . . . 9<br>HUSBAND/PARTNER OPPOS . . . 10<br>OTHERS OPPOSED . . . . 11<br>RELIGIOUS PROHIBITIO . . . . 12<br>SOCIAL PRESSUI . . . . 13<br><br>LACK OF KNOWLEDGE<br>KNOWS NO METHO . . . . 14<br>KNOWS NO SOURC . . . . 15<br><br>METHOD-RELATED REASONS<br>FEAR OF INFERTILITY . . . . 16<br>FEAR OF SIDE EFFECTS . . . . 17<br>INTERFERES WITH BODY'S<br>NORMAL PROCESSES . . . . 18<br>OTHER HEALTH CONCE . . . . 19<br>LACK OF ACCESS/TOO FA . . . . . 20<br>LONG WAITING TIME . . . . 21<br>TOO BUSY/NO TIME . . . . 22<br>COSTS TOO MUCH . . . . 23<br>PREFERRED METHOD<br>NOT AVAILABLI . . . . 24<br>NO METHOD AVAILABLE . . . . 25<br>INCONVENIENT TO USE . . . . 26<br>NOT EFFECTIV . . . . 27<br>OTHER ____________________ 96<br>(SPECIFY)<br>DON'T KNOW 88	

NO.	QUESTIONS AND FILTERS	CODING CATEGORIES	SKIP
121	If you could choose any contraceptive method that you want, which method would you want or would you want your wife	NONE . . . . 0<br>FEMALE STERILIZATION . . . . 1<br>MALE STERILIZATION . . . . 2<br>IUD . . . . 3<br>INJECTABLES . . . . 4<br>IMPLANTS . . . . 5<br>PILL . . . . 6<br>CONDOM . . . . 7<br>FEMALE CONDOM . . . . 8<br>DIAPHRAGM/FOAM/JELLY . . . . 9<br>TWO DAY METHOD . . . . 10<br>STANDARD DAYS METHOD . . . . 11<br>LACTATIONAL AMEN. METHOD . . . . . 12<br>RHYTHM METHOD . . . . 13<br>WITHDRAWAL . . . . 14<br>OTHER MODERN METHOD . . . . 15<br>OTHER TRADITIONAL METHOD . . . 16	
122	In choosing a contraceptive method, what feature would be most important to you?<br><br>SELECT THE MOST IMPORTANT ONE.	EFFECTIVE AT PREVENTING PREGNANCY 1<br>DURATION OF EFFECT / LASTS LONG 2<br>NO RISK OF HARMING HEALTH 3<br>NO EFFECT ON REGULAR MONTHLY BLEEDING 4<br>NO UNPLEASANT SIDE EFFECTS 5<br>LOW COST 6<br>NO RISK OF INFERTILITY 7<br>NON-HORMONAL 8<br>NO NEED TO GO TO A CLINIC TO OBTAIN THE METHOD 9<br>IMMEDIATE RETURN TO FERTILITY 10<br>PROTECTS AGAINST STI/HIV 11<br>WANT TO TRY SOMETHING NEW / TIRED OF OLD METHOD 12<br>MY DOCTOR RECOMMENDED IT TO ME 13<br>MY WIFE WANTED ME TO USE THIS METHOD 14<br>OTHER WOMEN IN MY FAMILY HAVE USED THIS METHOD 15<br>FRIENDS HAVE USED THIS METHOD 16<br>EASILY AVAILABLE AT CLINIC 17<br>NO NEED TO REMEMBER USING THE METHOD 18<br>CAN BE USED FOR A LONG TIME WITHOUT NEED TO VISIT CLINIC OR RE-SUPPLY 19<br>CAN BE USED WITHOUT ANYONE ELSE KNOWING 20<br>DOES NOT INTERRUPT SEX 21<br>OTHER ______________________ 96<br>(SPECIFY)<br>DON'T KNOW . . . . 88<br>REFUSED 99	
124	The last time that you had sexual intercourse, did you or your partner use any method(s) of family planning?	YES . . . . 1<br>NO . . . . 2<br>DON'T KNOW 8	NO/DON'T KNOW → 618
125	Which method did you use? **(4)**<br><br>CIRCLE ALL MENTIONED.	NONE . . . . 0<br>FEMALE STERILIZATION . . . . 1<br>MALE STERILIZATION . . . . 2<br>IUD . . . . 3<br>INJECTABLES . . . . 4<br>IMPLANTS . . . . 5<br>PILL . . . . 6<br>CONDOM . . . . 7<br>FEMALE CONDOM . . . . 8<br>DIAPHRAGM/FOAM/JELLY . . . . 9<br>TWO DAY METHOD 10	

NO.	QUESTIONS AND FILTERS	CODING CATEGORIES	SKIP
		STANDARD DAYS METHOI . . . . 11<br>LACTATIONAL AMEN. METH(. . . . . 12<br>RHYTHM METHOI . . . . 13<br>WITHDRAWAL . . . . 14<br>OTHER MODERN METHOD . . . . 15<br>OTHER TRADITIONAL METHOD . . . 16	

NO.	QUESTIONS AND FILTERS	CODING CATEGORIES	SKIP
126	During the past week, how often have you found yourself thinking about sex with any interest or desire?	SEVERAL TIMES A DAY 1<br>AT LEAST ONCE A DAY 2<br>AT LEAST TWICE A WEEK 3<br>AT LEAST ONCE A WEEK 4<br>NOT AT ALL 5	
131A	From a scale of 1 to 10, with 1 being "extremely dissatisfied" and 10 being "extremely satisfied", how satisfied are you with your sex life overall?	SATISFACTION	
131B	From a scale of 1 to 10, with 1 being "extremely dissatisfied" and 10 being "extremely satisfied", how satisfied are you with your marriage overall?	SATISFACTION	
On a scale from Strongly Disagree to Strongly Agree, to what extent do you agree with the following statements?			
625A	I often feel embarrassed or uncomfortable during sex.	STRONGLY AGREE 1<br>SOMEWHAT AGREE 2<br>NEUTRAL 3<br>SOMEWHAT DISAGREE 4<br>STRONGLY DISAGREE 5	

NO.	QUESTIONS AND FILTERS	CODING CATEGORIES	SKIP
625B	Sex is a natural thing that both men should be free to enjoy.	STRONGLY AGREE 1<br>SOMEWHAT AGREE 2<br>NEUTRAL 3<br>SOMEWHAT DISAGREE 4<br>STRONGLY DISAGREE 5	
625C	Consider the following scenario. A man's wife privately tells him that she's been unsatisfied with his sexual performance for a long time. He has been having sex with her regularly without knowing that anything was wrong. How would you expect him to respond?	He would feel badly about himself and would wish that he performed 1<br>He would make more of an effort the next time they had sex. 2<br>He would think "this kind of thing is normal in a marriage." 3<br>He would be upset with his wife for not accepting him as he is. 4<br>He would feel angry and betrayed by [illegible] 5	
625D	Consider the following scenario. A man is walking through his village when he overhears a group of neighbours gossiping about him and his wife. The neighbours are laughing because the man's wife has been telling everyone in the village that she's been unsatisfied with his sexual performance for a long time. The man has been having sex with his wife regularly without knowing that anything was wrong. How would you expect him to respond?	[illegible] and would wish that he performed better. 1<br>He would make more of an effort the next time they had sex. 2<br>He would think "this kind of thing is normal in a marriage." 3<br>He would be upset with his wife for not accepting him as he is. 4<br>He would feel angry and betrayed by 5	
Now, I have some questions about your thoughts on how the use of family planning methods might affect men's and women's desire for sex.			
132	How likely do you think it is for a WOMAN's desire for sex to be affected while using family planning methods?	NOT AT ALL AFFECTED . . . . 0<br>VERY UNLIKELY . . . . 1<br>SOMEWHAT UNLIKEL` . . . . 2<br>NEUTRAL . . . . 3<br>SOMEWHAT LIKELY . . . . 4<br>VERY LIKELY . . . . 5<br>DEFINITELY AFFECTED 6	
133	How likely do you think it is for a MAN's desire for sex to be affected while using family planning methods?	NOT AT ALL AFFECTED . . . . 0<br>VERY UNLIKELY . . . . 1<br>SOMEWHAT UNLIKEL` . . . . 2<br>NEUTRAL . . . . 3<br>SOMEWHAT LIKELY . . . . 4<br>VERY LIKELY . . . . 5<br>DEFINITELY AFFECTED 6	

NO.	QUESTIONS AND FILTERS	CODING CATEGORIES	SKIP
134	CHECK 302: EVER USED A CONTRACEPTIVE METHOD?<br>YES, USED ☐ ↓ NO, NEVER USED / NOT ASKED ☐ →		135B
135A	Overall, how satisfied are you with your current family planning method?	VERY SATISFIED . . . . 1<br>SOMEWHAT SATISFIE . . . . 2<br>UNSURE/NEUTRA . . . . 3<br>SOMEWHAT UNSATISFI . . . . 4<br>VERY UNSATISFIED 5	
135B	Overall, how satisfied is your wife with your current family planning method?	VERY SATISFIED . . . . 1<br>SOMEWHAT SATISFIE . . . . 2<br>UNSURE/NEUTRA . . . . 3<br>SOMEWHAT UNSATISFI . . . . 4<br>VERY UNSATISFIED 5	
135C	Would you say that using contraception is mainly your decision, mainly your wife's decision, or did you both decide together?	MAINLY WOMAN . . . . 1<br>MOST WOMAN, SOME HUSBAND 2<br>JOINT DECISION . . . . 3<br>MOST HUSBAND, SOME WOMAN 4<br>MAINLY HUSBAND 5<br>OTHER ______________ 96<br>(SPECIFY)	718D
136	Would you say that NOT using contraception is mainly your decision, mainly your wife's decision, or did you both decide together?	MAINLY WOMAN . . . . 1<br>MOST WOMAN, SOME HUSBAND 2<br>JOINT DECISION . . . . 3<br>MOST HUSBAND, SOME WOMAN 4<br>MAINLY HUSBAND 5<br>OTHER ______________ 6<br>(SPECIFY)	
138	If you were to not use any family planning method, how likely do you think it is that your wife will become pregnant during the next year?	VERY UNLIKELY . . . . 1<br>SOMEWHAT UNLIKEL . . . . 2<br>NEUTRAL . . . . 3<br>SOMEWHAT LIKELY . . . . 4<br>VERY LIKELY 5	

NO.	QUESTIONS AND FILTERS	CODING CATEGORIES	SKIP
139	Who in your household makes decisions on big purchases (refrigerator, cars, houses, etc.)?	MAINLY WOMAN . . . . 1<br>MOST WOMAN, SOME HUSBAND 2<br>JOINT DECISION . . . . 3<br>MOST HUSBAND, SOME WOMAN 4<br>MAINLY HUSBAND 5<br>OTHER HOUSEHOLD MEMBER . 6<br>OTHER FRIENDS OR RELATIVES 7<br>OTHER ______ 96<br>(SPECIFY)	
140	Who usually decides how the money you earn will be used: mainly you, mainly your wife, or you and your wife jointly?	MAINLY WOMA . . . . 1<br>MOST WOMAN, SOME HUSB. . . . . . 2<br>JOINT DECISION . . . . 3<br>MOSTLY HUSBAND, SOME WO . . . 4<br>MAINLY HUSBANI . . . . 5<br>OTHER ______ 96<br>(SPECIFY)	
141	Would you say that the money that you earn is more than what your wife earns, less than what she earns, or about the same?	MORE THAN HI . . . . 1<br>LESS THAN HIN . . . . 2<br>ABOUT THE SAMI . . . . 3<br>WIFE/PARTNER DOESN'T<br>BRING IN ANY MONEY . . . . 4<br>DON'T KNOW . . . . 88<br>REFUSED 99	 <br><br><br>→ 906
142	Sometimes a husband is annoyed or angered by things that his wife does. In your opinion, is a husband justified in hitting or beating his wife in the following situations:		

		YES	NO	DK
If she goes out without telling him?	GOES OUT	1	2	8
If she neglects the children?	NEGL. CHILDREN . . .	1	2	8
If she argues with him?	ARGUES	1	2	8
If she refuses to have sex with him?	REFUSES SEX	1	2	8
If the food is not properly cooked?	FOOD	1	2	8

NO.	QUESTIONS AND FILTERS	CODING CATEGORIES	SKIP
148	Have you ever discussed using family planning methods with your wife?	YES . . . . 1<br>NO 2	1 → 150
149	Why have you not discussed using family planning methods with your wife?<br>RECORD ALL REASONS MENTIONED.	FERTILITY-RELATED REASONS<br>WANTS ANOTHER CHILD SOOI . 1<br>NOT HAVING SEX . . . . 2<br>INFREQUENT SE) . . . . 3<br>MENOPAUSAL/HYSTERECTOMY 4<br>CAN'T GET PREGNAN . . . . 5<br>NOT MENSTRUATED SINCE<br>LAST BIRTH . . . . 6<br>BREASTFEEDING . . . . 7<br>UP TO GOD/FATALIST . . . . 8<br><br>OPPOSITION TO USE<br>RESPONDENT OPPOSEI . . . . 9<br>WIFE/PARTNER OPPOSED . . . 10<br>OTHERS OPPOSED . . . . 11<br>RELIGIOUS PROHIBITIO . . . . 12<br>EMBARRASSED/ASHAM . . . . 13<br><br>LACK OF KNOWLEDGE<br>KNOWS NO METHO . . . . 14<br>KNOWS NO SOURC . . . . 15<br>OTHER ____________________ 96<br>(SPECIFY)<br>DON'T KNOW . . . . 88<br>REFUSED 99	
150	Which topics about family planning have you discussed with your wife?<br><br>RECORD ALL REASONS MENTIONED.	NUMBER OF CHILDREN . . . . 1<br>CONTRACEPTION . . . . 2<br>BIRTH SPACING AND TIMING 3<br>FERTILITY AND INFERTILITY . . . . . 4<br>SEX AND SEXUAL SATISFACTI(. . . 5<br>OTHER 96	
151	On a scale from 1 to 5, how would you rate your attitude towards family planning method usage in general (usage by your wife or yourself)?	STRONGLY OPPOSED . . . . 1<br>SOMEWHAT OPPOSE . . . . 2<br>NEUTRAL . . . . 3<br>SOMEWHAT SUPPORTI\ . . . . 4<br>STRONGLY SUPPORTIV 5	1 → 201<br>2 → 201
152	Why don't you approve of family planning?<br><br>1. He wants more children<br>2. He thinks using FP makes women promiscuous<br>3. Religious reasons<br>4. His mother/family does not approve<br>5. He worries about side effecs<br>6. He worries about infertility/impotence<br>7. He thinks using FP interferes with sex<br>8. He doesn't think that FP is effective at preventing pregnancy<br>9. He does not know of any FP method<br>10. He does not know where to get methods<br>11. He is embarrassed or afraid of using FP<br>12. He fears stigma from friends / family / community	Y N<br>MORE CHILDREN 1 2<br>PROMISCUOUS 1 2<br>RELIGIOUS REASONS 1 2<br>MIL/FAMILY 1 2<br>SIDE EFFECTS 1 2<br>INFERTILITY 1 2<br>INTERFERES WITH SEX 1 2<br>NOT EFFECTIVE 1 2<br>KNOWS NO METHOD 1 2<br>KNOWS NO SOURCE 1 2<br>EMBARRASSED 1 2<br>FEARS STIGMA 1 2	

NO.	QUESTIONS AND FILTERS	CODING CATEGORIES	SKIP
153	PLEASE ASK THE WOMAN TO COME BACK AND RECEIVE THE COUNSELING WITH HUSBAND TOGETHER.		

COUNSELOR TRACKING FORM: POST-COUNSELING QUESTIONNAIRE

RECORD THE FOLLOWING INFORMATION ONCE THE COUNSELING SESSION HAS ENDED.

NO.	QUESTIONS AND FILTERS	CODING CATEGORIES		SKIP
301	RECORD THE DATE AND TIME.	DAY . . . .<br>MONTH . . . .<br>YEAR . . . .<br>HOUR . . . .<br>MINUTES		
302A	Have all of your questions been answered today?<br>IF NO, ANSWER ANY UNANSWERED QUESTIONS.	YES . . . .<br>NO	1<br>2	
302B	Overall, how satisfied were you with your counseling session today?	NOT SATISFIED<br>SOMEWHAT SATISFIED<br>SATISFIED<br>VERY SATISFIED<br>NOT SURE / DON'T KNOW	1<br>2<br>3<br>4<br>88	
302C	Overall, how would you rate the length of the counseling?	TOO LONG<br>THE LENGTH IS JUST RIGHT<br>TOO SHORT<br>DON'T KNOW<br>REFUSED	1<br>3<br>9<br>88<br>99	
302D	Overall, how would you describe the amount of information provided to you during the counseling?	TOO MUCH INFORMATION<br>JUST RIGHT FOR WHAT I NEED TO<br>TOO LITTLE INFORMATION<br>DON'T KNOW<br>REFUSED	1<br>2<br>3<br>88<br>99	
302E	As a result of this counseling session, do you feel that you have more or fewer options in your choice of contraceptives?	MORE OPTIONS<br>SAME NUMBER OF OPTIONS<br>FEWER OPTIONS<br>DON'T KNOW<br>REFUSED	1<br>2<br>3<br>88<br>99	
302F	Overall, how would you describe the counseling session's helpfulness in your choosing family planning methods?	NOT AT ALL HELPFUL<br>A LITTLE HELPFUL<br>NEITHER HELPFUL NOR UNHELPFU<br>SOMEWHAT HELPFUL<br>VERY HELPFUL	1<br>2<br>3<br>4<br>5	

NO.	QUESTIONS AND FILTERS	CODING CATEGORIES	SKIP
Now that you have had the counseling session, I would now like to ask you a few questions about your family planning and contraception preferences and opinions.			
303	If you could choose any contraceptive method that you want today, which method would you want to use?<br>CIRCLE ALL MENTIONED.	FEMALE STERILIZATION . . . . 1<br>MALE STERILIZATION . . . . 2<br>IUD . . . . 3<br>INJECTABLES . . . . 4<br>IMPLANTS . . . . 5<br>PILL . . . . 6<br>CONDOM . . . . 7<br>FEMALE CONDOM . . . . 8<br>DIAPHRAGM/FOAM/JELLY . . . . 9<br>TWO DAY METHOD . . . . 10<br>STANDARD DAYS METHOD . . . . 11<br>LACTATIONAL AMEN. METHO . . . . . 12<br>RHYTHM METHOD . . . . 13<br>WITHDRAWAL . . . . 14<br>OTHER MODERN METHOD . . . . 15<br>OTHER TRADITIONAL METHOD . . . 16	
303A	Before you had the counseling session, you had mentioned that AN IDEAL CONTRACEPTION METHOD IS: (METHOD FROM 129). This time, you mentioned that (METHOD FROM 303) IS YOUR IDEAL METHOD. Have you changed your mind?	YES, CHANGED MIND . . . . 1<br>NO, DID NOT CHANGE MIND 2	 → 304
303C	Why did you change your mind?<br>RECORD ALL REASONS MENTIONED.	FORGOT WHAT SHE SAID EARLIER 1<br>MORE INFORMATION FROM PROVIDER 2<br>MORE INFORMATION FROM FRIENDS 3<br>MORE INFORMATION FROM HUSBAND 4<br>MORE INFORMATION FROM RELATIVES 5<br>MORE INFORMATION FROM OTHERS 6<br>LEARED FROM OTHERS' EXPERIEN 7<br>MORE INFORMATION FROM COUNSELOR 8<br>OTHER 96<br>DON'T KNOW 88<br>REFUSED 99<br>PLEASE SPECIFY:	
303B	How would you describe the degree to which your choice affected by the counseling session today?<br><br>5- STRONGLY AFFECTED, 1- NOT AT ALL AFFECTED	NOT AT ALL AFFECTED . . . . 1<br>SOMEWHAT UNAFFECTED . . . . 2<br>NEUTRAL . . . . 3<br>SOMEWHAT AFFEC . . . . 4<br>STRONGLY AFFECTED 5	

NO.	QUESTIONS AND FILTERS	CODING CATEGORIES		SKIP
304	In choosing a contraceptive method, what feature would be most important to you?<br><br>SELECT THE MOST IMPORTANT ONE.	EFFECTIVE AT PREVENTING PREGNANCY	1	
		DURATION OF EFFECT / LASTS LONG	2	
		NO RISK OF HARMING HEALTH	3	
		NO EFFECT ON REGULAR MONTHLY BLEEDING	4	
		NO UNPLEASANT SIDE EFFECTS	5	
		LOW COST	6	
		NO RISK OF INFERTILITY	7	
		NON-HORMONAL	8	
		NO NEED TO GO TO A CLINIC TO OBTAIN THE METHOD	9	
		IMMEDIATE RETURN TO FERTILITY	10	
		PROTECTS AGAINST STI/HIV	11	
		WANT TO TRY SOMETHING NEW / TIRED OF OLD METHOD	12	
		MY DOCTOR RECOMMENDED IT TO ME	13	
		MY HUSBAND WANTED ME TO USE THIS METHOD	14	
		OTHER WOMEN IN MY FAMILY HAVE USED THIS METHOD	15	
		FRIENDS HAVE USED THIS METHOD	16	
		EASILY AVAILABLE AT CLINIC	17	
		NO NEED TO REMEMBER USING THE METHOD	18	
		CAN BE USED FOR A LONG TIME WITHOUT NEED TO VISIT CLINIC OR RE-SUPPLY	19	
		CAN BE USED WITHOUT ANYONE ELSE KNOWING	20	
		DOES NOT INTERRUPT SEX	21	
		OTHER . (SPECIFY)	96	
		DON'T KNOW .	88	
		REFUSED . .	99	
	CHECK 303 WITH 129 FROM PRE-COUNSELING FORM:<br><br>TOP CHOICE DOES NOT MATCH ☐ ↓ TOP CHOICE MATCHES ☐ →			INTRO
305	Before you had the counseling session, you had mentioned that (ATTRIBUTE FROM 130) was most important to you. This time, you mentioned that (ATTRIBUTE FROM 304) is most important to you. Have you changed your mind?	YES, CHANGED MIND	1	
		NO, DID NOT CHANGE MIN	2	→ INTRO

NO.	QUESTIONS AND FILTERS	CODING CATEGORIES		SKIP
306	Why did you change your mind?<br>RECORD ALL REASONS MENTIONED.	FORGOT WHAT SHE SAID EARLIER	1	
		MORE INFORMATION FROM PROVIDER	2	
		MORE INFORMATION FROM FRIENDS	3	
		MORE INFORMATION FROM HUSBAND	4	
		MORE INFORMATION FROM RELATIVES	5	
		MORE INFORMATION FROM OTHERS	6	
		LEARED FROM OTHERS' EXPERIEN	7	
		MORE INFORMATION FROM COUNSELOR	8	
		OTHER	96	
		DON'T KNOW	88	
		REFUSED	99	
		PLEASE SPECIFY:		
306A	How would you describe the degree to which your choice affected by the counseling session today?<br><br>5- STRONGLY AFFECTED, 1- NOT AT ALL AFFECTED	NOT AT ALL AFFECTED	1	
		SOMEWHAT UNAFFECTED	2	
		NEUTRAL	3	
		SOMEWHAT AFFEC	4	
		STRONGLY AFFECTED	5	

NO.	QUESTIONS AND FILTERS	CODING CATEGORIES	SKIP

INTRO

I would now like to present you with two additional services. As part of this program, you are eligible to receive a package of health and family planning services over a **one month period**.

This document summarizes the services that are available to you as part of the program. I will present you with a folder that contains:
1. Information about the program and the different services that are available to you as part of this program
2. Contact information of key personnel and support staff
3. Your program identification card

Summary of Program Services:

As part of this program, you are entitled to receive the following two services:
1. A free transportation service to health and family planning clinics in Lilongwe
2. Family planning services and reimbursement for family planning-related costs up to 17,500 MWK, including for the treatment and management of any contraceptive-related side effects, costs for the procurement of contraception, and other related expenses.

Additional details about the program and about each of these services are provided below.

B. TRANSPORTATION SERVICE:

As part of this program, you will also be eligible to use a free taxi service to one family planning clinic in Lilongwe, the Good Health Kauma Clinic. If you wish to receive any family planning care or related reproductive health services at one of these clinics, you will be able to make an appointment with the taxi service to pick you up from your home. A private taxi will arrive at your home on the scheduled appointment date and time and will transport you to the Good Health Kauma Clinic. The Good Health Kauma Clinic in Lilongwe is well-reputed for providing comprehensive, high quality family planning and maternal health services, and women who go for services at this clinic will not have to wait more than 1 hour before being seen by a medical professional. Once you have completed your clinic visit, the taxi will transport you back from the clinic to your home. The taxi service will be provided by a driver who has been hired exclusively for

NO.	QUESTIONS AND FILTERS	CODING CATEGORIES	SKIP

from the clinic to your home. The taxi service will be provided by a driver who has been hired exclusively for your use to ensure that you are safely and effectively transported to and from your home to the clinic.

Key Terms and Conditions

1. This taxi service is meant for your personal and private use only – you may not be accompanied by anyone else in the taxi except for your children, spouse, and any other dependents.**You may invite your husband to accompany you in the taxi if you choose.**
2. You may use this taxi service only for the purpose of receiving family planning or reproductive health care at the Good Health Kauma clinic. Other reasons for calling on this service will not be accepted.
3. You may use this free taxi service as many times as you wish over the course of the program period.
4. You will receive the name and phone number of the taxi driver at the end of this session today. You will need to make an appointment with the driver by telephone to arrange for transport to the Good Health Kauma clinic.
5. You may use this transport service to the Good Health Kauma clinic only during the clinic's daily working hours, which are between 8 AM and 5 PM from Monday to Saturday.
6. To confirm the taxi driver's availability, you will need to notify him at least one day in advance of your clinic visit day to make sure that he will be able to transport you to the clinic at your preferred time.
7. You will also be accompanied in the taxi by one of the program managers. The program manager will make sure that you are receiving the services that you seek in an effective and efficient manner and will also be available to answer any of your questions or concerns.
8. At the end of each trip, the program manager will ask you some questions about your trip and will gather your feedback and experience with the service that was provided. She will also collect any feedback that you may have about your experiences with the taxi service so that this service can be improved in the future. Participation in this short survey is voluntary, and you may refuse to participate or end your participation at any time without penalty. Your responses to the survey questions will be kept confidential, and your name and other identifying information will always be kept anonymous.

C. FAMILY PLANNING REIMBURSEMENT SERVICE:

Hormonal contraceptives, such as the oral contraceptive pill, the injectable contraceptive, and contraceptive implants, are medicines or devices that can reduce a woman's risk of getting pregnant. These methods are effective in preventing pregnancy, are long-lasting, and are safe to use for most women. While most women do not experience side effects from using hormonal contraceptives, a few women may adversely react to using one of these methods. Some side effects from using hormonal contraception include:

• Weight gain
• Headaches
• Sore breasts
• Irregular periods
• Mood changes
• Decreased sexual desire
• Acne
• Nausea

Although these contraceptive-related side effects usually go away on their own after a few months of using the method, it is still important for women who may be experiencing any of these side effects to consult with a doctor and make sure that the method that they are using is best for them.

The final service that is provided to you as part of this program aims to improve follow-up care, comprehensive treatment, and management services in the rare case that you experience a side effect when using a method of family planning.

As part of this service, you will be financially reimbursed for any out of pocket expenditures that you incur for receiving family planning care at the Good Health Kauma Clinic. Family planning costs that will be reimbursed at the Good Health Kauma Clinic include:

• costs related to your purchase of family planning medications or contraceptive methods
• family planning consultation fees
• pregnancy test fees and other family planning-related lab test fees
• exam fees that are related to your family planning use

Your reimbursement allowance is in the amount of 17,500 MKW, and you will be reimbursed for family planning services over multiple visits at the Good Health Kauma Clinic over this one year period. For any family planning service that you receive at the Good Health Kauma Clinic, the cost of the service will be deducted from your 17,500 MKW reimbursement allowance, and you will not have to pay out of pocket.

When you arrive at the facility:

1. You will be received by the program manager on duty, who will assist you in any way that you need to receive the necessary care at the facility.
2. Once you have been seen by a doctor and have received the care that you need, the program manager will consult with the doctor and staff at the facility to determine:

a. the type of treatment that you received;
b. whether the treatment you received was for the management of contraceptive-related side effects or any other care related to family planning; and
c. the costs of receiving family planning care.

3. Following the consultation, any costs that you have incurred for receiving family planning related care will be reimbursed under the program. The types of costs that will be reimbursed include:

a. family planning-related consultation fees and medical exam costs

NO.	QUESTIONS AND FILTERS	CODING CATEGORIES	SKIP

[illegible]
b. costs associated with the procurement of contraceptives and family planning commodities
c. costs of medications, medical procedures, laboratory tests, consultations, and follow-up visits for the treatment and management of contraceptive-related side effects

Additional details and key conditions for using this reimbursement service are provided as follows:

Key Terms and Conditions
1. This family planning reimbursement service is meant for your personal and private use only – no other household member may use this service.
2. You may use this family planning reimbursement service as many times as you wish over the course of the program period.
3. Only costs that are related to the receipt of family planning care, contraceptive care, and the treatment and management of family planning related side effects will be covered under this reimbursement service. These costs include: costs of medications, costs of family planning methods, exams, and lab tests related to family planning care and treatment; costs of additional consultations at the health facility for family planning; costs of treatment for contraceptive related side effects; and costs of switching or discontinuing methods of family planning. Other types of medical costs, which include (but are not limited to): costs for receiving antenatal or postnatal care; costs related to pregnancy care; costs for the treatment of malaria or other illnesses; costs to cover the treatment of HIV/AIDS or other sexually transmitted diseases; and costs to cover any other services and procedures that have not been specified will NOT be covered under this program.
4. The maximum total reimbursement amount that you may receive for routine family planning care at the Good Health Kauma Clinic over the program period is 17,500 MKW. This reimbursement policy for routine family planning care does not apply for any care received at any other clinic.
5. The reimbursement service for covering the cost for the treatment for side effects will apply for all family planning methods and regardless of where the method(s) was procured in Lilongwe.
6. You are advised to keep receipts of any family planning related costs that you incur and would like to claim for reimbursement. These costs include costs of medications, consultations, and other related services. You will then need to contact a program manager and present these receipts to her. The program manager will then evaluate the receipt and determine whether the cost is eligible for reimbursement or not. All reimbursements for a valid incurred cost will be distributed as closely as possible to the time that the reimbursable cost was incurred.
7. At the end of each visit, the program manager will ask you some questions about the visit and will gather your feedback and experience with the services that were provided. Participation in this short survey is voluntary, and you may refuse to participate or end your participation at any time without penalty. Your responses to the survey questions will be kept confidential, and your name and other identifying information will always be kept anonymous.

PROGRAM PERIOD:

The length of this program is one month. You are therefore eligible to receive any of the services that I have mentioned above from today onwards for a one-month period.

PARTICIPATION IN THE PROGRAM:

Your participation in this program is completely voluntary – it is your choice whether or not to participate in any part of this program. If you choose to participate, you may change your mind at any time and not participate. If you have withdrawn from the program, you may also rejoin the program at any later time without any penalty within the one year program period. Refusal to participate or stopping your participation will involve no penalty or loss of benefits to which you are otherwise entitled.

COST OF ENROLLMENT:

There is no cost or fee to enroll or participate in this program. Unless mentioned otherwise, all the services that are part of this program are provided to you free of charge.

NO.	QUESTIONS AND FILTERS	CODING CATEGORIES	SKIP

RISKS:

No physical risks to participating in this program are anticipated.

ALTERNATIVES TO PARTICIPATION:

The alternative to participating in this program is not to participate. Your participation in this program is completely voluntary, and you may refuse to participate or withdraw from the program without penalty. If you have withdrawn from the program, you may also rejoin the program at any later time without any penalty within the six-month program period.

CAN MY TAKING PART IN THE PROGRAM END EARLY?

You may decide not to continue with the program at any time without it being held against you. You may withdraw by informing me or one of the program staff that you no longer wish to participate (no questions will be asked). You may also skip any part of the program, but can continue to participate in the rest of the program. If you decide to leave the program, please contact a program staff member.

Other Program Information:

Program Identification Card

You will receive a personalized Program Identification Card which will help program staff to identify you as a recipient of this program. You will need to present this card each time you wish to use any of our services, including:
• Before a counseling session
• When you wish to use a taxi service
• When you wish to receive a phone consultation
Please keep this card carefully with you, please make sure to bring it with you. As you can see on your card, you have been assigned a unique Program Identification Number.

Please remember your Program Identification Number – it will help program staff to verify your identity in the event that you lose your card. You will also be asked to repeat your Identification Number if you ever call any of our program staff by telephone.

Lost or Damaged Card Replacement:

If you lose or damage your card, please notify a program manager by telephone immediately so that you can get it replaced. When you call, the program manager will ask you for your Program Identification Number and will also ask you other questions so that she can verify your identity. You will then receive a new card within 3-5 days.

Program Personnel Contact Information:

The contact information of the key personnel of this program are provided below and will also be given to you in your introduction folder.

Taxi Driver Contact Information

Taxi Driver Contact Information

You will be provided with the name, phone number, and contact information of the taxi driver who will provide the transport service. The driver will be available to transport you to the Good Health Kauma Clinic during the clinic visiting hours between 8 AM and 5 PM from Monday to Friday. You will need to make an appointment with the driver one day in advance of your clinic visit, and he will inform you about his schedule and availability. If you are experiencing a family planning related emergency and cannot find any means of transport to the nearest clinic, you may call the driver as well.

Program Manager Contact Information

Should you have any questions or concerns about the program or about the services to which you are entitled, please do not hesitate to contact a program manager. At least one program manager will be available at any time. Her phone number and contact information is provided to you.

PROTECTION OF PRIVACY:

Your identity and responses to survey questions will be kept confidential. At no time will your actual identity be revealed to any non-program staff. All of your survey responses that you provide will be linked to your name, and your name has been assigned a random numerical code. Anyone who is part of the program staff will only know of your information by this code. We won’t use your name or information that would identify you in any publications or presentations. Your name and other identifying information will always be kept anonymous.

QUESTIONS OR CONCERNS?

If you have questions or concerns about this program, please contact Dr. Bagrey Ngwira, who is in charge of this program.

Dr. Bagrey Ngwira
Innovations for Poverty Action (IPA) Malawi
E-mail: bagreyngwira@gmail.com
Telephone: +265 999554003
Availability: Monday to Friday, 9 AM to 5 PM

This program has been reviewed by the Malawi National Health Sciences Research Committee (NHSRC). If you wish to speak with someone from the NHSRC, please contact them at: Ministry of Health, P.O. Box 30377, Lilongwe 3, Malawi, or by phone at +265 1 726 422/418, or by e-mail at mohdoccentre@gmail.com for any of the following:

NO.	QUESTIONS AND FILTERS	CODING CATEGORIES	SKIP
CONS	**Statement of Consent**<br>I have read the information in this form including risks and possible benefits. All my questions about the program have been answered to my satisfaction. I understand that I am free to withdraw at any time without penalty or loss of benefits to which I am otherwise entitled.<br><br>I consent to participate in the Innovations for Poverty Action (IPA) Malawi Maternal Health and Wellness Program.		
SIG	**SIGNATURE**<br><br>Your signature below indicates your permission to take part in this program.<br><br>NAME OF PARTICIPANT:<br>______________________________<br><br>SIGNATURE OF PARTICIPANT:<br>______________________________<br><br>DATE:<br>______________________________<br><br>NAME OF PERSON OBTAINING CONSENT:<br>______________________________		
SIG	In joint-counseling group (WHEN BOTH WOMAN AND THEIR HUSBAND RECEIVED THE COUNSELING)<br>**WOMAN'S SIGNATURE**<br><br>Your signature below indicates your permission to take part in this program.<br><br>NAME OF PARTICIPANT:<br>______________________________<br><br>SIGNATURE OF PARTICIPANT:<br>______________________________<br><br>DATE:<br>______________________________<br><br>NAME OF PERSON OBTAINING CONSENT:<br>______________________________<br><br>SIGNATURE OF PERSON OBTAINING CONSENT:<br>______________________________<br><br>DATE:<br>______________________________<br><br>**HUSBAND'S SIGNATURE:**<br><br>Your signature below indicates your permission to take part in this program.<br><br>NAME OF WOMAN'S HUSBAND: ______________________________<br><br>SIGNATURE OF WOMAN'S HUSBAND: ______________________________<br><br>DATE:<br>______________________________		
307	Do you have any questions about this program and about all of the services that are available to you?<br><br>ANSWER ALL QUESTIONS.	OK 1	

NO.	QUESTIONS AND FILTERS	CODING CATEGORIES		SKIP
308	If you could go to the clinic today, which method would you like to pick up?	FEMALE STERILIZATION	1	
		MALE STERILIZATION	2	
		IUD	3	
		INJECTABLES	4	
		IMPLANTS	5	
		PILL	6	
		CONDOM	7	
		FEMALE CONDOM	8	
		DIAPHRAGM/FOAM/JELLY	9	
		TWO DAY METHOD	10	
		STANDARD DAYS METHOD	11	
		LACTATIONAL AMEN. METHO	12	
		RHYTHM METHOD	13	
		WITHDRAWAL	14	
		OTHER MODERN METHOD	15	
		OTHER TRADITIONAL METHOD . . .	16	

NO.	QUESTIONS AND FILTERS	CODING CATEGORIES	SKIP
309	WAS ANYONE ELSE PRESENT IN THE HOUSEHOLD DURING THE COUNSELLING SESSION?	YES . . . . 1<br>NO 2	 → END-1
310	WHO ELSE WAS PRESENT IN THE HOUSEHOLD DURING THE COUNSELLING SESSION?<br><br>SELECT ALL THAT APPLY.	HUSBAND / PARTNER 1<br>CHILD 2<br>RELATIVE 3<br>FRIEND 4<br>NEIGHBOR 5<br>NOT SURE / DON'T KNOW 9<br>OTHER 96<br>IF OTHER, PLEASE SPECIFY________	→ END-1
311	DID THE WOMAN'S HUSBAND / PARTNER PARTICIPATE IN THE COUNSELLING SESSION?	YES . . . . 1<br>NO 2	
END-1	Thank you for your time today. I hope that you have a great day.		END FORM

DATE ____________________

CLINIC VISIT FORM
MALAWI BEHAVIORAL BIASES STUDY
BOSTON UNIVERSITY, IPA MALAWI

SECTION 0: STAFF IDENTIFICATION

NAME OF FIELD MANAGER / STAFF ____________________

FIELD STAFF MEMBER ID NUMBER . . . . [ ][ ][ ][ ]

NAME OF DRIVER ____________________

DRIVER ID NUMBER . . . . [ ][ ][ ][ ]

DRIVER PHONE NUMBER . . . . [ ][ ][ ][ ][ ][ ][ ][ ][ ][ ][ ][ ][ ][ ]

SECTION 1: RESPONDENT IDENTIFICATION

NAME OF RESPONDENT ____________________

ASK RESPONDENT TO PRESENT PROGRAM ID CARD:<br>Do you have your Program Identification Card with you?	YES . . . . 1<br>NO 2	→ GO TO SECTION 2

WOMAN PROGRAM ID NUMBER [ ][ ][ ][ ][ ][ ][ ][ ][ ][ ][ ][ ][ ][ ][ ][ ][ ][ ][ ]

CONFIRM THE FOLLOWING WITH THE RESPONDENT IS CORRECT:
1) THE ADDRESS, PHONE, AND E-MAIL INFORMATION ON THE PROGRAM ID CARD IS THE SAME AS THE INFORMATION THAT IS ON RECORD.
2) THE PHOTOGRAPH ON THE PROGRAM ID CARD MATCHES THE PHOTOGRAPH ON RECORD.

HOUSEHOLD ADDRESS ____________________
____
____
____

PRIMARY PHONE NO. OF RESPONDENT ____________________

ALTERNATE PHONE NO. OF RESPONDENT ____________________

RESPONDENT E-MAIL ____________________

PHOTOGRAPH OF THE RESPONDENT ____________________

SECTION 2: CONFIRMATION OF RESPONDENT IDENTITY

ASK THE RESPONDENT TO CONFIRM TWO OF THE FOLLOWING THREE ITEMS:
1) HER ADDRESS AND PHONE NUMBER
2) THE NAME OF HER HUSBAND/PARTNER
3) THE NAME OF HER OLDEST CHILD

CONFIRM THE FOLLOWING:
1) THE ADDRESS AND PHONE INFORMATION IS THE SAME AS THE INFORMATION THAT IS ON RECORD.
2) THE PHOTOGRAPH THAT IS LINKED TO THE RESPONDENT'S NAME MATCHES THE PHOTOGRAPH ON RECORD.
3) THE NAME OF THE RESPONDENT'S HUSBAND/PARTNER MATCHES THE NAME THAT IS ON RECORD.
4) THE NAME OF THE RESPONDENT'S OLDEST CHILD MATCHES THE NAME THAT IS ON RECORD.

HOUSEHOLD ADDRESS ______________________________

____

____

PRIMARY PHONE NO. OF RESPONDENT ______________________________

ALTERNATE PHONE NO. OF RESPONDENT ______________________________

RESPONDENT E-MAIL ______________________________

PHOTOGRAPH OF THE RESPONDENT ______________________________

NAME OF RESPONDENT'S OLDEST CHILD ______________________________

NAME OF RESPONDENT'S HUSBAND/PARTNER ______________________________

PRE CLINIC VISIT FORM

RECORD THE FOLLOWING INFORMATION BEFORE DEPARTING FOR THE CLINIC / HEALTH CENTER.

NO.	QUESTIONS AND FILTERS	CODING CATEGORIES	SKIP
101	RECORD THE DATE AND TIME.	DAY . . . .<br>MONTH . . . .<br>YEAR . . . .<br>HOUR . . . .<br>MINUTES	
102A	RECORD GPS COORDINATES OF PICK-UP LOCATION	LATITUDE: N/S, D D, X X . X X X<br>LONGITUDE: E/W, D D, X X . X X X	

RECORD THE FOLLOWING INFORMATION WHILE ON THE WAY TO THE CLINIC / HEALTH CENTER.
I would now like to ask you a few questions about your reasons for using our taxi service today. The information you provide will be used to improve the quality of the service.

NO.	QUESTIONS AND FILTERS	CODING CATEGORIES	SKIP
103	On what day and time did you call the taxi driver to arrange this transport?	DAY . . . .<br>MONTH . . . .<br>YEAR . . . .<br>HOUR . . . .<br>MINUTES	
104A	RECORD THE NAME OF THE DESTINATION CLINIC/HEALTH CENTER. CONFIRM THIS LOCATION WITH THE RESPONDENT.	GOOD HEALTH KAUMA CLINIC 1<br>OTHER TUNZA CLINIC 2<br>BLM CLINIC 3<br>OTHER CLINIC / HOSPITAL . . 4<br>DON'T KNOW 9	
104B	CHECK FOR PREGNANCY STATUS:<br>Are you currently pregnant?	YES 1<br>NO / UNSURE 2	1 → 105M
104C	Are you currently doing something or using any method to delay or avoid getting pregnant?	YES 1<br>NO 2	2 → 105M

NO.	QUESTIONS AND FILTERS	CODING CATEGORIES	SKIP
105	Which method(s) are you currently using?<br><br>CIRCLE ALL MENTIONED.	FEMALE STERILIZATION . . . . 1<br>MALE STERILIZATION . . . . 2<br>IUD . . . . 3<br>INJECTABLES . . . . 4<br>IMPLANTS . . . . 5<br>PILL . . . . 6<br>CONDOM . . . . 7<br>FEMALE CONDOM . . . . 8<br>DIAPHRAGM/FOAM/JELLY . . . . 9<br>TWO DAY METHOD . . . . 10<br>STANDARD DAYS METHOD . . . . 11<br>LACTATIONAL AMEN. METHOI . . . . . . 12<br>RHYTHM METHOD . . . . 13<br>WITHDRAWAL . . . . 14<br>OTHER MODERN METHOD . . . . 15<br>OTHER TRADITIONAL METHOD 16	1 → 105D<br>2 → 105D<br>3 → 105E<br>4 → 105F<br>5 → 105E<br>7 → 105C<br>8–15 → 105E
105B	What is the brand name of the pills you are using?<br><br>IF DON'T KNOW THE BRAND,<br>ASK TO SEE THE PACKAGE.	LOFEMINOL 01<br>MICROGYNON 02<br>OVRETTE . . . . 03<br><br>OTHER ______________ 96<br>(SPECIFY)<br>DON'T KNOW 98	01–96 → 105E
105C	What is the brand name of the condoms you are using?<br><br>IF DON'T KNOW THE BRAND,<br>ASK TO SEE THE PACKAGE.	CHISHANGO . . . . 01<br>MANYUCHI . . . . 02<br>CARE (FEMALE CONDOM) . . . . 03<br><br>OTHER ______________ 96<br>(SPECIFY)<br><br>DON'T KNOW 98	01–96 → 105E
105D	Do you remember the date the sterilization was performed?	YES . . . . 1<br>NO / UNSURE 2	
105D_M	In what month and year was the sterilization performed?		
105E	Do you remember the date since which you have been using this method without stopping?	YES . . . . 1<br>NO / UNSURE 2	
105E1	Since what month and year have you been using (CURRENT METHOD) without stopping?<br><br>PROBE: For how long have you been using (CURRENT METHOD) now without stopping?	MONTH . . . . [ ][ ]<br>YEAR . . . . [ ][ ][ ][ ]	
105F	FOR WOMEN WHO USE INJECTABLES:<br>When was the last time that you received an injectable?<br><br>RECORD NUMBER OF MONTHS SINCE LAST INJECTABLE.	MONTHS SINCE LAST INJECTABLE<br>[ ][ ]	

NO.	QUESTIONS AND FILTERS	CODING CATEGORIES	SKIP
105G	Where did you obtain (CURRENT METHOD) the last time? **(5)**<br><br>PROBE TO IDENTIFY THE TYPE OF SOURCE.<br><br>NAME OF SOURCE:<br>________________________<br><br>LOCATION OF SOURCE:<br><br>GPS COORDINATES:<br><br>LATITUDE:<br>N/S D D X X . X X X<br>[ ] [ ][ ] [ ][ ] . [ ][ ][ ]<br><br>LONGITUDE:<br>E/W D D X X . X X X<br>[ ] [ ][ ] [ ][ ] . [ ][ ][ ]	GOOD HEALTH KAUMA CLINIC 01<br>**PUBLIC SECTOR**<br>GOVT. HOSPITAL . . . . 11<br>GOVT. HEALTH CENTER . . . . 12<br>GOV'T HEALTH POST/<br>OUTREACH . . . . 13<br>MOBILE CLINIC . . . . 14<br>HSA . . . . 15<br>CBDA / DOOR-TO-DOOR . . . . 16<br>OTHER PUBLIC<br>SECTOR ____________ 17<br>(SPECIFY)<br><br>**CHAM / MISSION**<br>HOSPITAL . . . . 21<br>HEALTH CENTER . . . . 22<br>MOBILE CLINIC . . . . 23<br>DOOR-TO-DOOR . . . . 24<br><br>**PRIVATE MEDICAL SECTOR**<br>PRIVATE HOSPITAL / CLINIC . . . . . . 31<br>PHARMACY . . . . 32<br>PRIVATE DOCTOR . . . . 33<br>MOBILE CLINIC . . . . 34<br>CBDA / DOOR-TO-DOOR . . . . 35<br>OTHER PRIVATE MEDICAL<br>SECTOR ____________ 36<br>(SPECIFY)<br><br>**BANJA LA MTSOGOLO (BLM)** 41<br>**MACRO** 51<br>**TUNZA (PSI) CLINIC** 61<br>**YOUTH DROP IN CENTRE** 71<br><br>**OTHER SOURCE**<br>SHOP . . . . 81<br>CHURCH . . . . 82<br>FRIEND/RELATIVE . . . . 83<br><br>OTHER ____________ 96<br>(SPECIFY)	(01–36) → 105H<br><br>(41–96) → 105H

NO.	QUESTIONS AND FILTERS	CODING CATEGORIES	SKIP
105H	How many kilometers did you have to travel to reach this (SERVICE PROVIDER) to receive (CURRENT METHOD)?	KM . . . . [ ][ ]	
105I	How many minutes did it take for you to travel to this (SERVICE PROVIDER) to receive (CURRENT METHOD)?	MINUTES . . . . [ ][ ][ ][ ]	
105J	What mode(s) of transportation did you use to travel to this (SERVICE PROVIDER) to receive (CURRENT METHOD)?	NONE (RECEIVED AT HOME) 1<br>WALK 2<br>BICYCLE 3<br>MOTORCYCLE . . . . 4<br>BUS . . . . 5<br>CAR / TAXI 6<br>OTHER . . . . 96<br>DON'T KNOW 88<br>REFUSED 99	
105K	How much, in Malawian Kwacha, did you have to pay in transportation costs to go to this (SERVICE PROVIDER)?	MKW . [ ][ ][ ][ ][ ][ ]	

NO.	QUESTIONS AND FILTERS	CODING CATEGORIES	SKIP
105L	How many minutes did you have to wait at the (SERVICE PROVIDER) before you received (CURRENT METHOD)?	MINUTES . . . . [ ][ ][ ][ ]	
105M	What is the reason for your clinic visit today?	ROUTINE FP VISIT<br>FP CONSULTATION 11<br>START A NEW FP METHOD 12<br>RENEW / REFILL FP METHOD 13<br>SWITCH FP METHOD 14<br>OTHER ROUTINE 15<br>NON-ROUTINE FP VISIT<br>EMERGENCY CONSULTATION 21<br>SIDE EFFECTS TREATMENT 22<br>OTHER NON-ROUTINE 23<br>NOT SURE / DON'T KNOW 99	 <br>→ 106<br>→ 107<br>→ 108<br><br><br><br>→ 109
106	Which method(s) would you like to start?<br><br>CIRCLE ALL MENTIONED.	FEMALE STERILIZATION . . . . A<br>MALE STERILIZATION . . . . B<br>IUD . . . . C<br>INJECTABLES . . . . D<br>IMPLANTS . . . . E<br>PILL . . . . F<br>CONDOM . . . . G<br>FEMALE CONDOM . . . . H<br>DIAPHRAGM . . . . I<br>FOAM/JELLY . . . . J<br>STANDARD DAYS METHOD . . . . K<br>LACTATIONAL AMEN. METHOI . . . . . . L<br>RHYTHM METHOD . . . . M<br>WITHDRAWAL . . . . N<br>OTHER MODERN METHOD . . . . X<br>OTHER TRADITIONAL METHOD . . . . Y<br>DON'T KNOW / UNSURE . . . . Z	120
106A	Check with post-counseling preference whether the answer is the same or not.	YES . . . . 1<br>NO / UNSURE 2	111<br>106B
106B	CHECK POST-COUSELING SURVEY, IF IDEAL_METHOD IS NOT SAME AS THE METHOD SHE WANTS TO START<br><br>From a month ago, you mentioned you wanted to receive (METHOD) at the clinic. Are you still interested in receiving this method?	YES . . . . 1<br>NO 2	→ 110

NO.	QUESTIONS AND FILTERS	CODING CATEGORIES		SKIP
106C	Why do you prefer the START_METHOD over the IDEAL_METHOD at the post-counseling session?<br><br>RECORD ALL REASONS MENTIONED.	FORGOT WHAT SHE SAID EARLIER	0	
		EFFECTIVE AT PREVENTING PREGNANCY	1	
		DURATION OF EFFECT / LASTS LONG	2	
		NO RISK OF HARMING HEALTH		
		NO EFFECT ON REGULAR MONTHLY BLEED	3	
		NO UNPLEASANT SIDE EFFECTS	4	
		LOW COST	5	
		NO RISK OF INFERTILITY	6	
		NON-HORMONAL	7	
		NO NEED TO GO TO A CLINIC TO OBTAIN THE METHOD	8	
		IMMEDIATE RETURN TO FERTILITY	9	
		PROTECTS AGAINST STI/HIV	10	
		WANT TO TRY SOMETHING NEW / TIRED OF	11	
		MY DOCTOR RECOMMENDED IT TO ME	12	
		MY HUSBAND WANTED ME TO USE THIS ME	13	
		OTHER WOMEN IN MY FAMILY HAVE USED THIS METHOD	14	
		FRIENDS HAVE USED THIS METHOD	15	
		EASILY AVAILABLE AT CLINIC	16	
		NO NEED TO REMEMBER USING THE METH	17	
		CAN BE USED FOR A LONG TIME WITHOUT NEED TO VISIT CLINIC OR RE-SUPPLY	18	
		CAN BE USED WITHOUT ANYONE ELSE KNOWING	19	
		DOES NOT INTERRUPT SEX	20	
		OTHER ________________________ (SPECIFY)	96	
		DON'T KNOW	88	
		REFUSED	99	
107	For which method(s) would you like to get refills or renew?<br><br>CIRCLE ALL MENTIONED.	IUD	C	→
		INJECTABLES	D	→
		IMPLANTS	E	→
		PILL	F	→
		CONDOM	G	→
		FEMALE CONDOM	H	→
		DIAPHRAGM	I	→
		FOAM/JELLY	J	→
		OTHER MODERN METHOD	X	
		OTHER TRADITIONAL METHOD	Y	
		DON'T KNOW / UNSURE	Z	
107B	CHECK POST-COUSELING SURVEY: IF IDEAL_METHOD IS NOT THE SAME AS THE REFILLED METHOD<br>From a month ago, you mentioned you wanted to receive (METHOD) at the clinic. Are you still interested in receiving this method?			→
		YES	1	
		NO	2	

NO.	QUESTIONS AND FILTERS	CODING CATEGORIES	SKIP
107C	Why did you prefer (REFILLED METHOD) over (METHOD)?<br><br>RECORD ALL REASONS MENTIONED.	FORGOT WHAT SHE SAID EARLIER 0<br>EFFECTIVE AT PREVENTING PREGNANCY 1<br>DURATION OF EFFECT / LASTS LONG 2<br>NO RISK OF HARMING HEALTH<br>NO EFFECT ON REGULAR MONTHLY BLEED 3<br>NO UNPLEASANT SIDE EFFECTS 4<br>LOW COST 5<br>NO RISK OF INFERTILITY 6<br>NON-HORMONAL 7<br>NO NEED TO GO TO A CLINIC<br>TO OBTAIN THE METHOD 8<br>IMMEDIATE RETURN TO FERTILITY 9<br>PROTECTS AGAINST STI/HIV 10<br>WANT TO TRY SOMETHING NEW / TIRED OF 11<br>MY DOCTOR RECOMMENDED IT TO ME 12<br>MY HUSBAND WANTED ME TO USE THIS ME 13<br>OTHER WOMEN IN MY FAMILY HAVE<br>USED THIS METHOD 14<br>FRIENDS HAVE USED THIS METHOD 15<br>EASILY AVAILABLE AT CLINIC 16<br>NO NEED TO REMEMBER USING THE METH 17<br>CAN BE USED FOR A LONG TIME WITHOUT<br>NEED TO VISIT CLINIC OR RE-SUPPLY 18<br>CAN BE USED WITHOUT ANYONE<br>ELSE KNOWING 19<br>DOES NOT INTERRUPT SEX 20<br>OTHER ________________________ 96<br>(SPECIFY)<br>DON'T KNOW . . . . 88<br>REFUSED 99	
108	Which method(s) would you like to switch to?<br><br>CIRCLE ALL MENTIONED.	FEMALE STERILIZATION . . . . 1<br>MALE STERILIZATION . . . . 2<br>IUD . . . . 3<br>INJECTABLES . . . . 4<br>IMPLANTS . . . . 5<br>PILL . . . . 6<br>CONDOM . . . . 7<br>FEMALE CONDOM . . . . 8<br>DIAPHRAGM/FOAM/JELLY . . . . 9<br>TWO DAY METHOD . . . . 10<br>STANDARD DAYS METHOD . . . . 11<br>LACTATIONAL AMEN. METHOI . . . . . . 12<br>RHYTHM METHOD . . . . 13<br>WITHDRAWAL . . . . 14<br>OTHER MODERN METHOD . . . . 15<br>OTHER TRADITIONAL METHOD 16	(1–8) →
108A	CHECK POST-COUNSELING METHOD PREFERENCE.<br><br>IS THE METHOD MENTIONED IN 106 THE SAME?	YES . . . . 1<br>NO / UNSURE 2	1 → 111
108B	CHECK POST-COUNSELING SURVEY:<br>From a month ago, you mentioned you wanted to receive (METHOD) at the clinic. Are you still interested in receiving this method?	YES . . . . 1<br>NO 2	

NO.	QUESTIONS AND FILTERS	CODING CATEGORIES	SKIP
108C	Why do you prefer (CURRENT METHOD) over (PREVIOUS METHOD) (in post-counseling session)?<br><br>RECORD ALL REASONS MENTIONED.	FORGOT WHAT SHE SAID EARLIER 0<br>EFFECTIVE AT PREVENTING PREGNANCY 1<br>DURATION OF EFFECT / LASTS LONG 2<br>NO RISK OF HARMING HEALTH<br>NO EFFECT ON REGULAR MONTHLY BLEED 3<br>NO UNPLEASANT SIDE EFFECTS 4<br>LOW COST 5<br>NO RISK OF INFERTILITY 6<br>NON-HORMONAL 7<br>NO NEED TO GO TO A CLINIC<br>TO OBTAIN THE METHOD 8<br>IMMEDIATE RETURN TO FERTILITY 9<br>PROTECTS AGAINST STI/HIV 10<br>WANT TO TRY SOMETHING NEW / TIRED OF 11<br>MY DOCTOR RECOMMENDED IT TO ME 12<br>MY HUSBAND WANTED ME TO USE THIS ME 13<br>OTHER WOMEN IN MY FAMILY HAVE<br>USED THIS METHOD 14<br>FRIENDS HAVE USED THIS METHOD 15<br>EASILY AVAILABLE AT CLINIC 16<br>NO NEED TO REMEMBER USING THE METH 17<br>CAN BE USED FOR A LONG TIME WITHOUT<br>NEED TO VISIT CLINIC OR RE-SUPPLY 18<br>CAN BE USED WITHOUT ANYONE<br>ELSE KNOWING 19<br>DOES NOT INTERRUPT SEX 20<br>OTHER ________________________ 96<br>(SPECIFY)<br>DON'T KNOW . . . . 88<br>REFUSED 99	
109	Which family planning related side effects are you experiencing?<br><br>CIRCLE ALL MENTIONED.	HEADACHE . . . . A<br>DIZZINESS . . . . B<br>BREAST TENDERNESS . . . . C<br>NAUSEA . . . . D<br>IRREGULAR BLEEDING . . . . E<br>HEAVY BLEEDING . . . . F<br>VAGINAL IRRITATION . . . . G<br>SPOTTING . . . . H<br>MENSTRUAL CRAMPS . . . . I<br>WEIGHT GAIN . . . . J<br>CHANGE IN LIBIDO . . . . K<br>MOOD SWINGS . . . . L<br>RAISED BLOOD PRESSURE . . . . . . M<br>BLOOD CLOTS . . . . N<br>DON'T KNOW / UNSURE . . . . Z	

NO.	QUESTIONS AND FILTERS	CODING CATEGORIES	SKIP
109B	For which method do you want to get treatment for the management of side effects?<br><br>SELECT TOP METHOD CHOICE MENTIONED.	FEMALE STERILIZATION . . . . 1<br>MALE STERILIZATION . . . . 2<br>IUD . . . . 3<br>INJECTABLES . . . . 4<br>IMPLANTS . . . . 5<br>PILL . . . . 6<br>CONDOM . . . . 7<br>FEMALE CONDOM . . . . 8<br>DIAPHRAGM/FOAM/JELLY . . . . 9<br>TWO DAY METHOD . . . . 10<br>STANDARD DAYS METHOD . . . . 11<br>LACTATIONAL AMEN. METHOI . . . . . . 12<br>RHYTHM METHOD . . . . 13<br>WITHDRAWAL . . . . 14<br>OTHER MODERN METHOD . . . . 15<br>OTHER TRADITIONAL METHOD 16	(1–8) →
110	In the month since your counseling session, did you go to a pharmacy, clinic, or hospital to receive any additional family planning services (get a family planning method, receive a counseling session, renew a method, etc.)?	YES . . . . 1<br>NO 2	 → 112

NO.	QUESTIONS AND FILTERS	CODING CATEGORIES		SKIP
111A	Which pharmacy, clinic, or hospital did you go to?	GOOD HEALTH KAUMA CLINIC	01	
		**PUBLIC SECTOR**		
		GOVT. HOSPITAL	11	
		GOVT. HEALTH CENTER	12	
		GOV'T HEALTH POST/ OUTREACH	13	
		MOBILE CLINIC	14	
		HSA	15	
		CBDA / DOOR-TO-DOOR	16	
		OTHER PUBLIC SECTOR ________________ (SPECIFY)	17	
		**CHAM / MISSION**		
		HOSPITAL	21	
		HEALTH CENTER	22	
		MOBILE CLINIC	23	
		DOOR-TO-DOOR	24	
		**PRIVATE MEDICAL SECTOR**		
		PRIVATE HOSPITAL / CLINIC	31	
		PHARMACY	32	
		PRIVATE DOCTOR	33	
		MOBILE CLINIC	34	
		CBDA / DOOR-TO-DOOR	35	
		OTHER PRIVATE MEDICAL SECTOR ________________ (SPECIFY)	36	
		**BANJA LA MTSOGOLO (BLM)**	41	
		**MACRO**	51	
		**TUNZA (PSI) CLINIC**	61	
		**YOUTH DROP IN CENTRE**	71	
		**OTHER SOURCE**		
		SHOP	81	
		CHURCH	82	
		FRIEND/RELATIVE	83	
		OTHER ____________________ (SPECIFY)	96	

NO.	QUESTIONS AND FILTERS	CODING CATEGORIES	SKIP
111B	Did you receive a contraceptive method from the pharmacy, clinic, or hospital you visited?	YES . . . . 1<br>NO 2	2 → 112
111C	Which method(s) did you receive?<br><br>CIRCLE ALL MENTIONED.	FEMALE STERILIZATION . . . . 1<br>MALE STERILIZATION . . . . 2<br>IUD . . . . 3<br>INJECTABLES . . . . 4<br>IMPLANTS . . . . 5<br>PILL . . . . 6<br>CONDOM . . . . 7<br>FEMALE CONDOM . . . . 8<br>DIAPHRAGM/FOAM/JELLY . . . . 9<br>TWO DAY METHOD . . . . 10<br>STANDARD DAYS METHOD . . . . 11<br>LACTATIONAL AMEN. METHOI . . . . . . 12<br>RHYTHM METHOD . . . . 13<br>WITHDRAWAL . . . . 14<br>OTHER MODERN METHOD . . . . 15<br>OTHER TRADITIONAL METHOD 16	1–14 → 112
111D	On a scale from 1 to 5, how would you rate the extent to which you feel supported in using family planning / contraception methods?<br><br>5-I feel strongly supported; 1- I don't feel supported at all	[ ][ ]	
112	Did you discuss the information from the counseling with your husband after the counseling session ended?	YES . . . . 1<br>NO 2	1, 2 → 115
112A	On a scale from 1 to 5, how do you rate your husband's attitude towards using family planning methods?	STRONGLY AGAINST . . . . 1<br>SOMEWHAT AGAINST . . . . 2<br>NEUTRAL . . . . 3<br>SOMEWHAT SUPPORTIVE . . . . 4<br>STORNGLY SUPPORTIVE 5	
113	CHECK IF WOMEN RECEIVED COUNSELING BY HERSELF:<br>Did you discuss the information from your counseling session a month ago with anyone else?	YES . . . . 1<br>NO 2	
114	Who did you speak to about the counseling session?<br><br>RECORD ALL MENTIONED.	MY HUSBAND . . . . 1<br>MY PARENTS . . . . 2<br>MY FRIENDS . . . . 3<br>OTHER RELATIVES 4<br>RELIGIOUS LEADER 5<br>NEIGHBOR 6<br>OTHER (PLEASE SPECIFY) ________ 96	

NO.	QUESTIONS AND FILTERS	CODING CATEGORIES	SKIP
115	As a result of the counseling session, do you feel that you have more or fewer options in your choice of contraceptives?	MORE OPTIONS 1<br>SAME NUMBER OF OPTIONS 2<br>FEWER OPTIONS 3<br>DON'T KNOW 88<br>REFUSED 99	
115_0	On a scale from 1 to 5, with 1 being very unsatisfied and 5 being very satisfied, how would you rate your satisfaction with the counseling session you received a month ago?	[ ][ ]	
115A	Overall, how would you describe the amount of information provided to you during the counseling?	TOO MUCH INFORMATION 1<br>JUST RIGHT FOR WHAT I NEED TO KN 2<br>TOO LITTLE INFORMATION 3<br>DON'T KNOW 88<br>REFUSED 99	
116	Overall, how important do you think the counseling session was in helping you to decide whether or not to use family planning methods?	VERY IMPORTANT 1<br>SOMEWHAT IMPORTANT 2<br>NEITHER IMPORTANT NOR UNIMPOR 3<br>SOMEWHAT UNIMPORTANT 4<br>NOT AT ALL IMPORTANT 5	
117	Overall, to what extent do you think the counseling session affected your attitude towards family planning methods?	VERY POSITIVELY AFFECTS ATTITUD 1<br>SOMEWHAT POSITIVELY AFFECTS AT 2<br>NEITHER POSITIVE NOR NEGATIVE 3<br>SOMEWHAT NEGATIVELY AFFECTS A 4<br>VERY NEGATIVELY AFFECTS ATTITU 5	

Now, I have some general questions about family planning methods and pregnancy for you.

NO.	QUESTIONS AND FILTERS	CODING CATEGORIES	SKIP
122A	In your opinion, during a woman's monthly cycle, are there certain days when she is more likely to become pregnant if she has sex?	YES . . . . 1<br>NO . . . . 2<br>DON'T KNOW . . . . 88<br>REFUSED 99	 → 122C<br>→ 122C<br>→ 122C
122B	For most women, what is the time when she is more likely to get pregnant?	ON THE DAY OF HER PERIOD 1<br>EARLY IN HER PERIOD (DAYS 1-7) 2<br>MIDDLE OF HER PERIOD (DAYS 8-19) 3<br>END OF HER PERIOD (20-30) 4<br>DON'T KNOW . . . . 88<br>REFUSED 99	
122C	In your opinion, is it possible for a woman who is breastfeeding to not become pregnant?	YES . . . . 1<br>NO . . . . 2<br>DON'T KNOW . . . . 88<br>REFUSED 99	 → 122E<br>→ 122E<br>→ 122E

NO.	QUESTIONS AND FILTERS	CODING CATEGORIES		SKIP
122D	In your opinion, for how long can breastfeeding help to prevent pregnancy?<br><br>RECORD ANSWER IN MONTHS.	NUMBER OF MONTHS . . . . .<br>DON'T KNOW . . . .<br>REFUSED	[ ][ ]<br>88<br>99	
122E	In your opinion, for how long should a woman who has just given birth wait before trying to get pregnant in order to minimize any health risks for her next birth?<br><br>RECORD ANSWER IN MONTHS IF LESS THAN ONE YEAR. IF RIGHT AWAY, RECORD '00'.	NUMBER OF MONTHS . . . . .<br>ONE TO LESS THAN TWO YEARS<br>TWO TO LESS THAN THREE YEARS<br>THREE YEARS OR MORE . . . .<br>DON'T KNOW . . . .<br>REFUSED	[ ][ ]<br>13<br>14<br>15<br>88<br>99	

Now, I have some questions about your thoughts on how the use of family planning methods might affect women.

123A	How likely do you think it is for a woman to gain weight while using family planning methods?	VERY UNLIKELY . . . .<br>SOMEWHAT UNLIKELY . . . .<br>NEUTRAL . . . .<br>SOMEWHAT LIKELY . . . .<br>VERY LIKELY	1<br>2<br>3<br>4<br>5	
123B	How likely do you think it is for a woman's menstrual cycle to change or stop while using family planning methods?	VERY UNLIKELY . . . .<br>SOMEWHAT UNLIKELY . . . .<br>NEUTRAL . . . .<br>SOMEWHAT LIKELY . . . .<br>VERY LIKELY	1<br>2<br>3<br>4<br>5	
123C	How likely do you think it is for a woman's ability to become pregnant to be affected while using family planning methods?	VERY UNLIKELY . . . .<br>SOMEWHAT UNLIKELY . . . .<br>NEUTRAL . . . .<br>SOMEWHAT LIKELY . . . .<br>VERY LIKELY	1<br>2<br>3<br>4<br>5	

As I read each of the following statements, please tell me how much you, personally, agree or disagree with each statement. Do you strongly agree, somewhat agree, somewhat disagree, or strongly disagree with the following:

123D	It is too much of a hassle to use a condom every time you have sex.	STRONGLY AGREE . . . .<br>SOMEWHAT AGREE . . . .<br>NEUTRAL . . . .<br>SOMEWHAT DISAGREE . . . .<br>STRONGLY DISAGREE	1<br>2<br>3<br>4<br>5	
123E	Using family planning is morally wrong.	STRONGLY AGREE . . . .<br>SOMEWHAT AGREE . . . .<br>NEUTRAL . . . .<br>SOMEWHAT DISAGREE . . . .<br>STRONGLY DISAGREE	1<br>2<br>3<br>4<br>5	

NO.	QUESTIONS AND FILTERS	CODING CATEGORIES	SKIP
123F	It doesn't matter whether you use family planning methods or not; when it is your time to get pregnant, it will happen.	STRONGLY AGREI . . . . 1<br>SOMEWHAT AGREE . . . . 2<br>NEUTRAL . . . . 3<br>SOMEWHAT DISAGREI . . . . 4<br>STRONGLY DISAGRI 5	
123G	It is mainly a woman's responsibility to make decisions about family planning methods.	STRONGLY AGREI . . . . 1<br>SOMEWHAT AGREE . . . . 2<br>NEUTRAL . . . . 3<br>SOMEWHAT DISAGREI . . . . 4<br>STRONGLY DISAGRI 5	
123H	In your opinion, what are some of the benefits or advantages to using family planning?<br><br>RECORD ALL MENTIONED.	PREVENTS UNWANTED PREGNANCY 1<br>ALLOWS ME TO SPACI . . . . 2<br>PROTECTS AGAINST STI/HIV . . . . . . 3<br>BENEFITS MY HEALTH . . . . 4<br>SAVES US MONEY . . . . 5<br>ALLOWS ME TO SPEND<br>MORE ON CHILDREN . . . . 6<br>ALLOWS ME TO ENJOY SEX MORE . . 7<br>OTHER (SPECIFY) . . . . 8<br>DON'T KNOW . . . . 88<br>REFUSE 99	
123J	In your opinion, what are some of the costs or disadvantages to using family planning?<br><br>RECORD ALL MENTIONED	NOT EFFECTIVE/N . . . . 1<br>METHODS ARE EXPEN . . . . 2<br>TAKES TOO MUCH . . . . 3<br>EMBARASSING TO USI . . . . 4<br>SIDE EFFECTS WITH USING FP . . . . 5<br>RISK OF INFERTILITY . . . . 6<br>HARMS MY HEALTH . . . . 7<br>HUSBAND OPPOSED . . . . 8<br>OTHERS OPPOSED . . . . 9<br>RELIGIOUS OPPOSITION . . . . 10<br>INTERFERES WITH SEX . . . . 11<br>OTHER (SPECIFY) . . . . 96<br>DON'T KNOW 88	
118	OBSERVE:<br>WAS THE WOMAN ACCOMPANIED TO THE CLINIC BY HER HUSBAND?	YES . . . . 1<br>NO 2	→ 120
119A	Did you discuss your plans to visit to the clinic today with your husband?	YES . . . . 1<br>NO 2	→ 120
119B	Since June 2019, have you discussed seeking family planning services with your husband?	YES . . . . 1<br>NO 2	→ 120
119C	Why did you not discuss your visit to the clinic with your husband?	YES . . . . 1<br>NO 2	→ 120

NO.	QUESTIONS AND FILTERS	CODING CATEGORIES	SKIP
Now, I would like to ask you few questions about the people and their opinions, other than your HUSBAND/PARTNER, with whom you have had informal/formal discussions regarding matters of family life, such as childbearing, contraception, family planning, or other related matters. These people can live nearby or far away, and you might talk to them frequently or infrequently.			
119D	Besides your husband/partner, is there anyone else with whom you discuss issues about family planning, reproductive health, or childbearing?	YES . . . . 1<br>NO 2	1 → 119D1
119D1	Other than your husband / partner, with how many other people have you had informal or formal discussions regarding matters of family life, such as childbearing, contraception, family planning, or other related matters?	|__|__|	
119E	Besides your husband/partner, have you told anyone else about your participation in the Malawi Behavioral Biases Study [illegible]	YES . . . . 1<br>NO 2	1 → 119F
119F	Other than your husband / partner, how many other people have you told about your participation in the Malawi Behavioral Biases Study program?<br><br>PROBE: Can you think of anyone else?<br><br>RECORD UP TO 5. IF 'DON'T KNOW', RECORD '88'. IF 'REFUSED', RECORD '99'.	|__|__|	
119F1	Would you mind telling me their names? Please remember that all of this information will be kept confidential.	YES . . . . 1<br>NO 2	1 → 119F2
I will now ask you about your first friend whom you told about your participation in the Malawi Behavioral Biases Study program.			
119F2	Friend's First Name:<br>Friend's Last Name:	__________________<br>__________________	
I will now ask you about your next friend whom you told about your participation in the Malawi Behavioral Biases Study program.			
Check if answer to 119F is larger than or equal to 2:<br>119F3	Friend's First Name:<br>Friend's Last Name:	__________________<br>__________________	
119G	Besides your husband/partner, has anyone else told you about their participation in the Malawi Behavioral Biases Study program?	YES . . . . 1<br>NO 2	1 → 119H

NO.	QUESTIONS AND FILTERS	CODING CATEGORIES	SKIP
119H	Other than your husband / partner, how many other people have told you about their participation in the Malawi Behavioral Biases Study program?<br><br>PROBE: Can you think of anyone else?<br><br>RECORD UP TO 5. IF 'DON'T KNOW', RECORD '88'. IF 'REFUSED', RECORD '99'.	[ ][ ]	
119H1	Would you mind telling me their names? Please remember that all of this information will be kept confidential.	YES . . . . 1<br>NO 2	1 → 120
I will now ask you about your first friend who told you about their participation in the Malawi Behavioral Biases Study program.			
119H2	Friend's First Name:<br>Friend's Last Name:	____________________<br>____________________	
I will now ask you about your next friend who told you about their participation in the Malawi Behavioral Biases Study program.			
Check if answer to 119F is larger than or equal to 2:<br>119H3	Friend's First Name:<br>Friend's Last Name:	____________________<br>____________________	
119I	Have you ever participated in other family planning studies or programs where you discussed matters of family life, such as childbearing, contraception, family planning, or other related matters?	YES . . . . 1<br>NO 2	
119J	Have you participated in a study named Malawi Family Planning Study?	YES . . . . 1<br>NO 2	2 → 119K
119K	Besides your husband/partner, has anyone else told you about their participation in the Malawi Family Planning Study program?	YES . . . . 1<br>NO 2	1 → 119L
119L	Other than your husband / partner, how many other people have told you about their participation in the Malawi Family Planning Study program?<br><br>PROBE: Can you think of anyone else?<br><br>RECORD UP TO 5. IF 'DON'T KNOW', RECORD '88'. IF 'REFUSED', RECORD '99'.	[ ][ ]	
119L1	Would you mind telling me their names? Please remember that all of this information will be kept confidential.	YES . . . . 1<br>NO 2	1 → 120

NO.	QUESTIONS AND FILTERS	CODING CATEGORIES	SKIP
I will now ask you about your first friend/neighbour who told you about their participation in the Malawi Family Study program.			
119L2	Friend's First Name:<br>Friend's Last Name:	______________________<br>______________________	
I will now ask you about your next friend who told you about their participation in the Malawi Family Planning Study program.			
Check if answer to 119F is larger than or equal to 2:<br>119L3	Friend's First Name:<br>Friend's Last Name:	______________________<br>______________________	
**RECORD THE FOLLOWING INFORMATION ONCE THE TAXI HAS REACHED THE CLINIC / HEALTH CENTER SITE.**			
120	RECORD GPS COORDINATES OF CLINIC DROP-OFF LOCATION	LATITUDE: N/S [ ] D D [ ][ ] X X [ ][ ] . X X X [ ][ ][ ]<br>LONGITUDE: E/W [ ] D D [ ][ ] X X [ ][ ] . X X X [ ][ ][ ]	
121	RECORD THE TIME.	HOUR . . . . [ ][ ]<br>MINUTES . . . . [ ][ ]	

POST CLINIC VISIT FORM

RECORD THE FOLLOWING INFORMATION ONCE THE WOMAN HAS FINISHED HER VISIT.			
201	RECORD THE TIME.	HOUR . . . . [ ][ ]<br>MINUTES . . . . [ ][ ]	
202	Did you receive the services and care that you wanted?	YES . . . . 1<br>NO 2	 → 202
203	Which services did you receive at the clinic today?	ROUTINE FP VISIT<br>FP CONSULTATION 11<br>START A NEW FP METHOD 12<br>RENEW / REFILL FP METHOD 13<br>SWITCH FP METHOD 14<br>OTHER ROUTINE 15<br>NON-ROUTINE FP VISIT<br>EMERGENCY CONSULTATION 21<br>SIDE EFFECTS TREATMENT 22<br>OTHER NON-ROUTINE 23<br>NOT SURE / DON'T KNOW 99	 <br>→ 204<br>→ 205<br>→ 206<br><br><br><br>→ 207
204	Which method(s) did you start?<br><br>CIRCLE ALL MENTIONED.	FEMALE STERILIZATION . . . . 1<br>MALE STERILIZATION . . . . 2<br>IUD . . . . 3<br>INJECTABLES . . . . 4<br>IMPLANTS . . . . 5<br>PILL . . . . 6<br>CONDOM . . . . 7<br>FEMALE CONDOM . . . . 8<br>DIAPHRAGM/FOAM/JELLY . . . . 9<br>TWO DAY METHOD . . . . 10<br>STANDARD DAYS METHOD . . . . 11<br>LACTATIONAL AMEN. METHOD . . . . . 12<br>RHYTHM METHOD . . . . 13<br>WITHDRAWAL . . . . 14<br>OTHER MODERN METHOD . . . . 15<br>OTHER TRADITIONAL METHOD . . . 16	→ 207A
205	For which method(s) did you get refills or renew?<br><br>CIRCLE ALL MENTIONED.	IUD . . . . C<br>INJECTABLES . . . . D<br>IMPLANTS . . . . E<br>PILL . . . . F<br>CONDOM . . . . G<br>FEMALE CONDOM . . . . H<br>DIAPHRAGM . . . . I<br>FOAM/JELLY . . . . J<br>OTHER MODERN METHOD . . . . X<br>OTHER TRADITIONAL METHOD . . . Y<br>DON'T KNOW / UNSURE . . . . Z	→ 207A

206	Which method(s) did you switch to?<br><br>CIRCLE ALL MENTIONED.	FEMALE STERILIZATION . . . . 1<br>MALE STERILIZATION . . . . 2<br>IUD . . . . 3<br>INJECTABLES . . . . 4<br>IMPLANTS . . . . 5<br>PILL . . . . 6<br>CONDOM . . . . 7<br>FEMALE CONDOM . . . . 8<br>DIAPHRAGM/FOAM/JELLY . . . . 9<br>TWO DAY METHOD . . . . 10<br>STANDARD DAYS METHOD . . . . 11<br>LACTATIONAL AMEN. METHOI . . . . . 12<br>RHYTHM METHOD . . . . 13<br>WITHDRAWAL . . . . 14<br>OTHER MODERN METHOD . . . . 15<br>OTHER TRADITIONAL METHOD . . . 16	→ 207A
207	For which method(s) did you get treatment for the management of side effects?<br><br>CIRCLE ALL MENTIONED.	IUD . . . . C<br>INJECTABLES . . . . D<br>IMPLANTS . . . . E<br>PILL . . . . F<br>FOAM/JELLY . . . . J<br>OTHER MODERN METHOD . . . . X<br>OTHER TRADITIONAL METHOD . . . Y<br>DON'T KNOW / UNSURE . . . . Z	
207A	CHECK PRE-CLINIC METHOD PREFERENCE FROM 106 AND 112.<br><br>IS THE METHOD MENTIONED IN 106/107/108 THE SAME?	YES . . . . 1<br><br>NO / UNSURE 2	→ 209
207B	Why did you change your choice of method?<br><br>RECORD ALL REASONS MENTIONED.	FORGOT WHAT SHE SAID EARLIER 0<br>EFFECTIVE AT PREVENTING PREGNANCY 1<br>DURATION OF EFFECT / LASTS LONG 2<br>NO RISK OF HARMING HEALTH<br>NO EFFECT ON REGULAR MONTHLY BLEE 3<br>NO UNPLEASANT SIDE EFFECTS 4<br>LOW COST 5<br>NO RISK OF INFERTILITY 6<br>NON-HORMONAL 7<br>NO NEED TO GO TO A CLINIC<br>TO OBTAIN THE METHOD 8<br>IMMEDIATE RETURN TO FERTILITY 9<br>PROTECTS AGAINST STI/HIV 10<br>WANT TO TRY SOMETHING NEW / TIRED (11<br>MY DOCTOR RECOMMENDED IT TO ME 12<br>MY HUSBAND WANTED ME TO USE THIS M 13<br>OTHER WOMEN IN MY FAMILY HAVE<br>USED THIS METHOD 14<br>FRIENDS HAVE USED THIS METHOD 15<br>EASILY AVAILABLE AT CLINIC 16<br>NO NEED TO REMEMBER USING THE MET 17<br>CAN BE USED FOR A LONG TIME WITHOUT<br>NEED TO VISIT CLINIC OR RE-SUPPLY 18<br>CAN BE USED WITHOUT ANYONE<br>ELSE KNOWING 19<br>DOES NOT INTERRUPT SEX 20<br>OTHER ______________________ 96<br>(SPECIFY)<br>DON'T KNOW . . . . 88<br>REFUSED 99	

208	Why didn't you receive the services you wanted?	METHOD NOT AVAILABLE AT CLIN . 1<br>CLINICIAN SUGGESTED THIS METHC 222<br>TAKES A LONG TIME TO APPLY<br>THE METHOD . . . . 333<br>OPPOSITION (HUSBAND/OTHERS)<br>OTHERS__________ (SPECIFY)	→
209	How long, in minutes, did you have to wait before receiving services?<br><br>IF THE WOMAN WAS SEEN IMMEDIATELY, RECORD '00'.	MINUTES . . . . [ ][ ]	
210	Overall, how satisfied were you with your visit today?	NOT SATISFIED 1<br>SOMEWHAT SATISFIED 2<br>SATISFIED 3<br>VERY SATISFIED 4<br>NOT SURE / DON'T KNOW 9	
211	For your visit today, have you incurred any medical costs for receiving FAMILY PLANNING related treatment? These costs may include: costs for medical exams or tests, consultation fees, medication costs, contraceptive commodity costs, etc.	YES . . . . 1<br><br>NO 2	 <br>→ 214
212	For which family planning related service(s) received did you have to pay today?<br><br>CIRCLE ALL MENTIONED.	MEDICAL EXAM(S) . . . . 1<br>MEDICAL TEST(S) . . . . 2<br>OTHER CONSULTATION FEES . . . . . 3<br>MEDICATION . . . . 4<br>CONTRACEPTIVES . . . . 5<br>OTHER (SPECIFY) ________________ 8	
213	In total, how much did you have to pay for receiving these family planning related services today?<br>RECORD AMOUNT TO THE NEAREST MKW.	AMOUNT [ ][ ][ ][ ][ ][ ]	
214	For your visit today, have you incurred any medical costs for receiving NON-FAMILY PLANNING related treatment?	YES . . . . 1<br><br>NO 2	 <br>→ 217
215	For which non-family planning related service(s) received did you have to pay today?<br><br>CIRCLE ALL MENTIONED.	MEDICAL EXAM(S) . . . . 1<br>MEDICAL TEST(S) . . . . 2<br>OTHER CONSULTATION FEES . . . . . 3<br>MEDICATION . . . . 4<br>OTHER (SPECIFY) ________________ 8	
216	In total, how much do you have to pay for receiving these non-family planning related services today?<br>RECORD AMOUNT TO THE NEAREST MKW.	AMOUNT [ ][ ][ ][ ][ ][ ]	

CONFIRM THE COST INFORMATION WITH THE DOCTOR / STAFF MEMBER AT THE CLINIC. ONCE YOU HAVE RECEIVED CONFIRMATION, APPROACH THE RESPONDENT.

217	The total amount that you owe for the services that you received here today is: (**Q213 + Q216) MKW.**<br><br>Of this amount, the program will be able to pay for **Q213 MKW**, which are costs that you have incurred to receive family planning related care.<br><br>You will therefore have to pay the remaining **Q216 MKW** for receiving care that was not related to family planning.<br><br>Do you have any questions about this arrangement?<br><br>ANSWER ANY QUESTIONS THAT THE RESPONDENT MAY HAVE. PAY THE FACILITY IN THE AMOUNT OF **Q213 MKW** TO COVER FAMILY PLANNING RELATED COSTS. OBTAIN A RECEIPT FROM THE FACILITY FOR THE PROJECT RECORDS.	

RECORD THE FOLLOWING INFORMATION AT THE END OF THE TRIP WHEN THE RESPONDENT ARRIVES AT HER FINAL DESTINATION.

218A	RECORD GPS COORDINATES OF DROP-OFF LOCATION	LATITUDE: N/S [ ] D D [ ][ ] X X [ ][ ] . X X X [ ][ ][ ]<br>LONGITUDE: E/W [ ] D D [ ][ ] X X [ ][ ] . X X X [ ][ ][ ]	
218	WHERE WAS THE RESPONDENT DROPPED OFF?	HOME . . . . 1<br>OTHER LOCATION 2	
219	DID ANYONE ELSE TRAVEL WITH THE RESPONDENT TO THE HOSPITAL / CLINIC / HEALTH CENTER?	YES . . . . 1<br>NO 2	→ END-1
220	WHO WAS WITH THE RESPONDENT WHEN SHE TRAVELED TO THE HOSPITAL / CLINIC / HEALTH CENTER?<br><br>SELECT ALL THAT APPLY.	HUSBAND / PARTNER 1<br>CHILD 2<br>RELATIVE 3<br>FRIEND 4<br>NEIGHBOR 5<br>NOT SURE / DON'T KNOW 9	
221	RECORD THE TIME.	HOUR . . . . [ ][ ]<br>MINUTES . . . . [ ][ ]	

INNOVATIONS FOR POVERTY ACTION (IPA) MALAWI MATERNAL HEALTH AND WELLNESS PROGRAM

TERMS OF SERVICE

Introduction:

I am pleased to inform you that you have been invited to participate in a maternal health and wellness program that aims to promote access to health services for women. As part of this program, you are eligible to receive a package of health and family planning services over approximately a **one month** period.

Eligibility:

You are eligible to participate in this program because:

1. You are a married woman;
2. You are between the ages of 18 to 35;
3. You live in Lilongwe;
4. You are currently not pregnant and did not give birth within the 6 months prior to the initial screening
5. You have neither been sterilized nor have had a hysterectomy
6. You have given birth to at least one child in your lifetime
7. You are living with your husband
8. You were recently interviewed by an interviewer from Innovations for Poverty Action (IPA) Malawi on your health and well-being.

This document summarizes the services that are available to you as part of the program. A counselor will present you with a folder that contains:

1. Information about the program and the different services that are available to you as part of this program
2. Contact information of key personnel and support staff
3. Your program identification card

Summary of Program Services:

As part of this program, you are entitled to receive the following three services:

1. One free private counseling session with a trained health and family planning counselor
2. A free transportation service to health and family planning clinics in Lilongwe
3. Free family planning services and reimbursement for family planning-related costs, including for the treatment and management of any contraceptive-related side effects, costs for the procurement of contraception, and other related expenses.

Additional details about the program and about each of these services are provided below.

A. COUNSELING SERVICE:

As part of this program, you are eligible to receive one free counseling session today. This counseling session will be provided to you by a highly trained counselor who will visit you here at your home. In this session, you will discuss different topics related to your health and well-being and the role that family planning can play to help you achieve your ideal family goals. Some topics that will be discussed in these sessions include:

- your fertility preferences and goals
- how you and your husband / partner can healthily plan for your next birth together
- the benefits of family planning and reproductive health for women like you

This session will provide you with an opportunity to share your experiences and to ask any questions that you may have about your pregnancy, childbirth, and family planning.

Husband Invitation

In addition, you are welcome to invite your husband to this counseling session if you choose. It is your choice as to whether or not you would like to invite your husband to the counseling session.

Key Terms and Conditions

1. **This counseling session will last between 15 to 20 minutes.**
2. You may choose to end this counseling session at any time, and you may also choose to end your participation in this counseling service at any time.
3. You will receive a phone call from a counselor two days before a scheduled session to confirm the date and time of your appointment.
4. You will be able to reschedule your appointment to a time that is convenient for you.
5. The counseling session will be held in a private location here in your house, and all discussions between you and the counselor will be kept confidential.
6. This counseling service is meant mainly **for your personal use,** and **you are encouraged to invite your husband to receive the counseling with you. In that case, the counselor will counsel you and your husband together at the same time.**
7. At the end of the counseling session, the counselor will ask you some questions about the session and will gather your feedback and experience with the service that was provided. Participation in this short survey is voluntary, and you may refuse to participate or end your participation at any time without penalty. Your responses to the survey questions will be kept confidential, and your name and other identifying information will always be kept anonymous.
8. If you choose to invite your husband, the counselor will ask him some questions before the counseling session to learn about his fertility preferences and family planning use.

B. TRANSPORTATION SERVICE:

As part of this program, you will also be eligible to use a free taxi service to one family planning clinic in Lilongwe, the Good Health Kauma Clinic. If you wish to receive any family planning care or related reproductive health services at this clinic, you will be able to make an appointment with the taxi service to pick you up from your home. A private taxi will arrive at your home on the scheduled appointment date and time and will transport you to the Good Health Kauma Clinic.

The Good Health Kauma Clinic in Lilongwe is well-reputed for providing comprehensive, high quality family planning and maternal health services, and women who go for services at this clinic will not have to wait more than 1 hour before being seen by a medical professional. Once you have completed your clinic visit, the taxi will transport you back from the clinic to your home. The taxi service will be provided by a driver who has been hired exclusively for your use to ensure that you are safely and effectively transported to and from your home to the clinic.

Key Terms and Conditions

1. This taxi service is meant for **your personal use** – you may not be accompanied by anyone else in the taxi except for your children, husband, and any other dependents**. You may invite your husband to accompany you in the taxi if you choose.**
2. You may use this taxi service **only for the purpose of receiving family planning or reproductive health care at the Good Health Kauma clinic.** Other reasons for calling on this service will not be accepted.
3. You may use this free taxi service as many times as you wish over the course of the program period.
4. You will receive the name and phone number of the taxi driver during your first visit with a counselor. You will need to make an appointment with the program manager by telephone to arrange for transport to the Good Health Kauma clinic.
5. You may use this transport service to the Good Health Kauma clinic only during the clinic's daily working hours, which are between **8 AM and 5 PM from Monday to Friday**.
6. To confirm the taxi driver's availability, you will need to notify the program manager Violet at least **one day** in advance of your clinic visit day to make sure that the driver will be able to transport you to the clinic at your preferred time.
7. You will also be accompanied in the taxi by one of the program managers. The program manager will make sure that you are receiving the services that you seek in an effective and efficient manner and will also be available to answer any of your questions or concerns.
8. At the end of each trip, the program manager will ask you some questions about your trip and will gather your feedback and experience with the service that was provided. She will also collect any feedback that you may have about your experiences with the taxi service so that this service can be improved in the future. Participation in this short survey is voluntary, and you may refuse to participate or end your participation at any time without penalty. Your responses to the survey questions will be kept confidential, and your name and other identifying information will always be kept anonymous.

C. FAMILY PLANNING REIMBURSEMENT SERVICE:

Hormonal contraceptives, such as the oral contraceptive pill, the injectable contraceptive, and contraceptive implants, are medicines or devices that can reduce a woman's risk of getting pregnant. These methods are effective in preventing pregnancy, are long-lasting, and are safe to use for most women. While most women do not experience side effects from using hormonal contraceptives, a few women may adversely react to using one of these methods. Some side effects from using hormonal contraception include:

- Weight gain
- Headaches
- Sore breasts

- Irregular periods
- Mood changes
- Decreased sexual desire
- Acne
- Nausea

Although these contraceptive-related side effects usually go away on their own after a few months of using the method, it is still important for women who may be experiencing any of these side effects to consult with a doctor and make sure that the method that they are using is best for them.

The final service that is provided to you as part of this program aims to improve follow-up care, comprehensive treatment, and management services in the rare case that you experience a side effect when using a method of family planning.

As part of this service, you will be financially reimbursed for any out of pocket expenditures that you incur for receiving family planning care at the Good Health Kauma Clinic. Family planning costs that will be reimbursed at the Good Health Kauma Clinic include:

- costs related to your purchase of family planning medications or contraceptive methods
- family planning consultation fees
- pregnancy test fees and other family planning-related lab test fees
- exam fees that are related to your family planning use

Your reimbursement allowance is in the amount of **17,500 MKW,** and you will be reimbursed for family planning services over multiple visits at the Good Health Kauma Clinic over this one month period. For any family planning service that you receive at the Good Health Kauma Clinic, the cost of the service will be deducted from your 17,500 MKW reimbursement allowance, and you will not have to pay out of pocket.

When you arrive at the facility:

1. You will be received by the program manager on duty, who will assist you in any way that you need to receive the necessary care at the facility.
2. Once you have been seen by a doctor and have received the care that you need, the program manager will consult with the doctor and staff at the facility to determine:
    a. the type of treatment that you received;
    b. whether the treatment you received was for the management of contraceptive-related side effects or any other care related to family planning; and
    c. the costs of receiving family planning care.
3. Following the consultation, any costs that you have incurred for receiving family planning related care will be reimbursed under the program. The types of costs that will be reimbursed include:
    a. family planning-related consultation fees and medical exam costs
    b. costs associated with the procurement of contraceptives and family planning commodities
    c. costs of medications, medical procedures, laboratory tests, consultations, and follow-up visits for the treatment and management of contraceptive-related side effects

Additional details and key conditions for using this reimbursement service are provided as follows:

Key Terms and Conditions

1. This family planning reimbursement service is meant for **your personal and private use only** – no other household member may use this service.
2. You may use this family planning reimbursement service as many times as you wish over the course of the program period.
3. **Only costs that are related to the receipt of family planning care, contraceptive care, and the treatment and management of family planning related side effects will be covered under this reimbursement service.** These costs include: costs of medications, costs of family planning methods, exams, and lab tests related to family planning care and treatment; costs of additional consultations at the health facility for family planning; costs of treatment for contraceptive related side effects; and costs of switching or discontinuing methods of family planning. **Other types of medical costs, which include (but are not limited to): costs for receiving antenatal or postnatal care; costs related to pregnancy care; costs for the treatment of malaria or other illnesses; costs to cover the treatment of HIV/AIDS or other sexually transmitted diseases; and costs to cover any other services and procedures that have not been specified will NOT be covered under this program.**
4. The **maximum total reimbursement amount** that you may receive for routine family planning care at the Good Health Kauma Clinic over the program period is **17,500 MKW**. This reimbursement policy for routine family planning care does not apply for any care received at any other clinic.
5. The reimbursement service for covering the cost for the treatment for side effects will apply for **all family planning methods** and regardless of where the method(s) was procured in Lilongwe.
6. You are advised to keep receipts of any family planning related costs that you incur and would like to claim for reimbursement. These costs include costs of medications, consultations, and other related services. You will then need to contact the program manager and present these receipts to her. The program manager will then evaluate the receipt and determine whether the cost is eligible for reimbursement or not. All reimbursements for a valid incurred cost will be distributed as closely as possible to the time that the reimbursable cost was incurred.
7. At the end of each visit, the program manager will ask you some questions about the visit and will gather your feedback and experience with the services that were provided. Participation in this short survey is voluntary, and you may refuse to participate or end your participation at any time without penalty. Your responses to the survey questions will be kept confidential, and your name and other identifying information will always be kept anonymous.

PROGRAM PERIOD:

The length of this program is one month. You are therefore eligible to receive any of the services that I have mentioned above from today onwards for a one-month period.

PARTICIPATION IN THE PROGRAM:

Your participation in this program is completely voluntary – it is your choice whether or not to participate in any part of this program. If you choose to participate, you may change your mind at any time and not participate. If you have withdrawn from the program, you may also rejoin the program at any later time without any penalty within the one month program period. Refusal to participate or stopping your participation will involve no penalty or loss of benefits to which you are otherwise entitled.

COST OF ENROLLMENT:

There is no cost or fee to enroll or participate in this program. Unless mentioned otherwise, all the services that are part of this program are provided to you free of charge.

RISKS:

No physical risks to participating in this program are anticipated.

ALTERNATIVES TO PARTICIPATION:

The alternative to participating in this program is not to participate. Your participation in this program is completely voluntary, and you may refuse to participate or withdraw from the program without penalty. If you have withdrawn from the program, you may also rejoin the program at any later time without any penalty within the one-month program period.

CAN MY TAKING PART IN THE PROGRAM END EARLY?

You may decide not to continue with the program at any time without it being held against you. You may withdraw by informing me or one of the program staff that you no longer wish to participate (no questions will be asked). You may also skip any part of the program, but can continue to participate in the rest of the program. If you decide to leave the program, please contact a program staff member.

Other Program Information:

Program Identification Card

You will receive a personalized Program Identification Card which will **help program staff to identify you as a recipient of this program**. You will need to present this card each time you wish to use any of our services, including:

- Before a counseling session
- When you wish to use a taxi service
- When you wish to receive a phone consultation

Please keep this card carefully with you, please make sure to bring it with you. As you can see on your card, you have been assigned a unique Program Identification Number.

Please remember your Program Identification Number – it will help program staff to verify your identity in the event that you lose your card. You will also be asked to repeat your Identification Number if you ever call any of our program staff by telephone.

Lost or Damaged Card Replacement:

If you lose or damage your card, please notify the program manager by telephone immediately so that you can get it replaced. When you call, the program manager will ask you for your Program Identification Number and will also ask you other questions so that she can verify your identity. You will then receive a new card within 3-5 days.

Mobile Phone Credit

You will receive phone airtime credit in the amount of 100 MWK. This phone credit is intended to cover any communications expenses (phone credit for consultations, calling the taxi service, etc.) that you may incur. **The phone credit is yours to keep even if you never participate in any part of our program, use any of our services, or experience a contraceptive related side effect.**

Program Personnel Contact Information:

The contact information of the key personnel of this program are provided below and will also be given to you in your introduction folder.

Taxi Driver Contact Information

You will be provided with the name, phone number, and contact information of the taxi driver who will provide the transport service. The driver will be available to transport you to the Good Health Kauma Clinic during the clinic visiting hours between **8 AM and 5 PM from Monday to Friday**. You will need to make an appointment with the program manager **one day in advance of your clinic visit**, and she will inform you about her schedule and availability. If you are experiencing a family planning related emergency and cannot find any means of transport to the nearest clinic, you may call the driver as well.

Program Manager Contact Information

Should you have any questions or concerns about the program or about the services to which you are entitled, please do not hesitate to contact the program manager. Her phone number and contact information is provided to you.

PROTECTION OF PRIVACY:

Your identity and responses to survey questions will be kept confidential. At no time will your actual identity be revealed to any non-program staff. All of your survey responses that you provide will be linked to your name, and your name has been assigned a random numerical code. Anyone who is part of the program staff will only know of your information by this code. We won’t use

your name or information that would identify you in any publications or presentations. Your name and other identifying information will always be kept anonymous.

QUESTIONS OR CONCERNS?

If you have questions or concerns about this program, please contact **Dr. Bagrey Ngwira**, who is in charge of this program.

Dr. Bagrey Ngwira
Innovations for Poverty Action (IPA) Malawi
E-mail: bagreyngwira@gmail.com
Telephone: +265 999554003, +265 888554003
Availability: Monday to Friday, 9 AM to 5 PM

This program has been reviewed by the Malawi National Health Sciences Research Committee (NHSRC). If you wish to speak with someone from the NHSRC, please contact them at: Ministry of Health, P.O. Box 30377, Lilongwe 3, Malawi, or by phone at +265 1 726 422/418, or by e-mail at mohdoccentre@gmail.com for any of the following:

- If your questions, concerns, or complaints are not being answered by the program staff,
- If you cannot reach the program staff,
- If you want to talk to someone besides the program staff,
- If you have questions about your rights as a participant, or
- If you want to get information or provide input about this program.

Statement of Consent

I have read the information in this form including risks and possible benefits. All my questions about the program have been answered to my satisfaction. I understand that I am free to withdraw at any time without penalty or loss of benefits to which I am otherwise entitled.

I consent to participate in the Innovations for Poverty Action (IPA) Malawi Maternal Health and Wellness Program.

SIGNATURE

TO WOMAN:
Your signature below indicates your permission to take part in this program

____
Name of participant

______________________________ ______________
Signature / Thumbprint of participant Date

______________________________ ______________
Signature of person obtaining consent Date

Printed name of person obtaining consent

TO HUSBAND:
Your signature below indicates your permission to take part in this program

Name of woman’s husband

Signature / Thumbprint of woman’s husband

Date

Signature of person obtaining consent

Date

Printed name of person obtaining consent

INNOVATIONS FOR POVERTY ACTION (IPA) MALAWI MATERNAL HEALTH AND WELLNESS PROGRAM

TERMS OF SERVICE

Malonje:

Ndili okondwa kukudziwitsani kuti mwaitanidwa kuti mutenge nawo gawo mupulogalamu ya uchembere ndi umoyo wabwino yomwe cholinga chake ndikutukula kupezeka kwa umoyo wabwino wa azimayi. Ngati mbali imodzi ya pulogalamuyi, muli oyenera kulandira phukusi la zaumoyo ndi utumiki wazakulera pafupifupi kwa mwezi umodzi.

Zoyenereza:

Mukuyenera kutenga nawo mbali mu pulogalamuyi chifukwa:
1. Ndinu mayi wapabanja;
2. Muli ndi zaka zapakati pa 18 mpaka 35;
3. Mumakhala ku Lilongwe;
4. Mulibe pakati padakali pano komanso simunabereke mu miyezi isanu ndi umodzi yapitayi
5. Simunaseketse kapena kuchotsedwa chibelekero
6. Munabelekapo pafupifupi kamodzi (mwana wamoyo) mmoyo wanu
7. Mumakhala ndi amuna anu
8. Munayenderedwa posachedwapa ndi wakafukufuku ochekera ku Innovations for Poverty Action (IPA) Malawi pa zaumoyo ndi thanzi lanu

Mwachidule kalatayi ikufotokoza za mautumiki omwe azapelekedwe kwa inu ngati mbali imodzi ya pulogalamuyi. Phungu azakupatsani foda yomwe muli:
1. Uthenga okhudza pulogalamuyi ndi mautumiki osiyanasiyana omwe azapelekedwa kwa inu ngatimbali imodzi ya pulogalamuyi
2. Za munthu yemwe mungalumikizane naye ndi sitafu yonse yothandizira
3. Khadi yanu yokuzindikiritsani mu pulogalamuyi (ID)

Summary of Program Services:

Ngati mbali imodzi ya pulogalamuyi, muli oyenera kulandira mautumiki atatu awa:
1. Uphungu wapadera kamodzi ndi phungu ophunzitsidwa bwino pa zaumoyo ndi kulera
2. Thiransipoti yaulere ya kuchipatala cha zaumoyo ndi kulera mu Lilongwe
3. Njira zakulera zaulere ndi kubwezeredwa ndalama ngati mwalandira thandizo lazakulera, kuphatikizanso chithandizo ndi chisamaliro cha zotsatira zobwera chifukwa chogwiritsa ntchito njira zakulera, zolipira pogula njira zakulera, ndi zina zokhudza zimenezi.

Tsatanetsatane oonjezera okhudza pulogalamuyi ndi utumiki ulionse zaperekedwa munsimu.

A. COUNSELING SERVICE:

Ngati mbali imodzi ya pulogalamuyi, muli oyenera kulandira uphungu wapadera kamodzi lero. Uphungu umenewu uperekedwa kwa inu ndi phungu ophunzitsidwa bwino yemwe akuyendereni pakhomo panu. Mugawo limeneli, muzakambirana mitu yosiyanasiyana yokhudza umoyo ndi thanzi lanu komanso gawo lomwe kulera kungatenge mbali kukuthandizani kufikira zolinga zabwino zabanja lanu. Mwa mitu ina yomwe ikambidwe mugawo limeneli ndi:

- Ana omwe mungakonde kubereka ndi zolinga
- Mmene inu ndi amuna anu mungapangire limodzi mapulani mwathanzi oberekanso
- Ubwino wakulera ndi uchembere wabwino kwa azimayi ngati inu

Gawo ili likupatsani mwayi ogawa zomwe mumakumana nazo ndiponso kufunsa mafunso alionse omwe mungakhale nawo okhudzana ndi pakati, kubereka komanso kulera.

Kuitana Amuna

Mongoonjezera, muli oloredwa kuitana amuna anu ku gawo la uphunguli ngati mungakonde. Ndichisankho chanu kufuna kapena kusafuna kuitana amuna anu mu gawo lauphunguli.

Key Terms and Conditions

1. **Gawo lauphunguwu litenga pakati pa mphindi 15 kapena 20 mpaka ola limodzi**
2. Mutha kusankha kuthetsa gawo la uphunguli nthawi ina iliyonse, muthanso kusankha kuthetsa kutenga nawo gawo mu uphunguwu nthawi iliyonse.
3. Mudzaimbiridwa phone kuchokera kwa phungu masiku awiri, lisanakwane tsiku lomwe mungagwirizane kudzatenga gawo kuti mugwirizane tsiku ndi nthawi yokumana.
4. Mukhonza kusintha nthawi yokumana nkuika nthawi yomwe ili yoyenera kwa inu.
5. Gawo lauphunguli lidzachitikira pamalo obisika konkuno kunyumba kwanu, ndipo zokambirana zonse pakati pa inu ndi phungu zizasungidwa mwachinsinsi.
6. Uphungu umenewu kwenikweni ndi oti **mugwiritse ntchito painu nokha. Mukupemphedwa kuitana amuna anu, mukatero phungu adzakupatsani uphungu nonse limodzi inu ndi amuna anu nthawi imodzi.**
7. Pamapeto pa gawo lauphunguwu, phungu azakufunsani mafunso okhudza gawoli ndipo azasonkhanitsa ndemanga ndi zokumana nazo pamautumiki omwe anaperekedwa. Kutenga gawo mukafukufukuyi ndimwakufuna kwanu, mutha kukana kutenga nawo gawo kapena kuthetsa kutenga kwanu gawo nthawi ina iliyonse popanda chilango chilichonse. Mayankho anu pamafunso akafukufukuyi azasungidwa mwachinsinsi ndipo zonse zokuzindikiritsani zizasungidwa mosakuzindikiritsani nthawi zonse.
8. Mukasankha kuitana amuna anu, phungu adzawafunsa mafunso angapo pamayambiliro pa gawo la uphungu kuti tidziwe maganizo awo pa nkhani ya chisankho chawo pakubeleka ndiponso njira zakulera.

B. TRANSPORTATION SERVICE:

Ngati gawo limodzi la pulogalamu, muzakhala oyenera kugwiritsa ntchito taxi yaulere kupita ku chipatala chazakulera mu Lilongwe, ku Good Health Kauma Clinic. Ngati mungakonde kukalandira chithandizo chazakulera kapena zokhudzana ndi uchembere wabwino kumodzi mwa zipatala zimenezi, muzatha kupanga ndondomeko yoti taxi izakutengeni pakhomo panu. Taxi yapadera idzafika pakhomo panu pa tsiku ndi nthawi yomwe mwagwirizana ndipo idzakutengani kupita kuchipatala cha Good Health Kauma. Chipatala cha Good Health ndichodziwika bwino popeleka chithandizo chokwanira, njira zolera zapamwamba ndi uchembere wabwino, azimayi omwe amapita kukalandira chithandizo kumeneku samadikira kopitilira ola limodzi asanaonane ndi a dokotala. Pamene mwamaliza zonse kuchipatala, taxi idzakutengani kuchoka kuchipatala kubwelera kunyumba kwanu. Utumiki wa taxi udzapelekedwa ndi dalaivala yemwe wapangidwa hayala ndicholinga chokhacho choti inu mugwiritse ntchito potsimikiza kuti mwatetezeka ndipo mwayendetsedwa bwino kupita kuchipatala ndikubwelera kunyumba kwanu.

Key Terms and Conditions

1. Utumiki wa taxi ndi oti **mugwiritse ntchito inu nokha** – simudzapita ndi wina aliyense mu taxi kupatula ana anu, amuna anu ndi ena okhala nawo. **Mukhonza kuitana amuna anu kuti mupite limodzi mu taxi ngati mungapange chisankho**.
2. Mudzagwirirtsa ntchito utumiki wa taxi **pacholinga chokalandira kulera kapena chithandizo cha uchembere wabwino basi kuchipatala cha Good Health Kauma.** Zifukwa zina zoimbira kufuna utumikiwu sizidzaloredwa.
3. Mutha kugwiritsa ntchito utumiki wa taxi yaulereyi maulendo mmene mungafunire panthawi ya pulogalamuyi.
4. Mudzalandira dzina ndi phone nambala ya dalaivala wa taxi ulendo oyamba omwe phungu adzakuyendereni. Mukuyenera kukambirana ndi dalaivala pa phone kuti mugwirizane zamayendedwe kupita kuchipatala cha Good Health Kauma.
5. Mungagwiritse ntchito mayendedwewa kupita kuchipatala cha Good Health Kauma panthawi yokhayo yomwe chipatala chimakhala chikugwira ntchito tsiku ndi tsiku, nthawiyi ndi pakati pa **8 koloko mamawa ndi 5 koloko madzulo kuyambira lolemba mpaka lachisanu**
6. Pofuna kutsimikiza kupezeka kwa a dalaivala, mukuyenera kuwadziwitsa osachepera tsiku limodzi lisanafike tsiku lanu lopita kuchipatala pofuna kutsimikiza kuti adzakwanitsa kukutengani kupita kuchipatala panthawi yomwe mufuna.
7. Mudzapitanso limodzi mu taxi ndi mtsogoleri mmodzi wa pulogalamuyi. Mtsogoleri wa pulogalamuyi adzaonetsetsa kuti mukulandira chithandizo bwino ndi moyenelera komanso azapezeka kuti azayankhe mafunso anu ndi nkhawa zanu.
8. Pamapeto paulendowu, mtsogoleriyu adzakufunsani mafunso okhudza ulendowu ndi azatolera ndemanga ndi zochitika zautumiki omwe munapatsidwa. Azatoleranso ndemanga zilizonse zokhudza utumiki wa taxi ndicholinga chofuna kupititsa patsogolo utumikiwu mtsogolo muno. Kutenga gawo mukafukufukuyi ndikosakakamika, mutha kukana kutenga nawo gawo kapena kuthetsa kutenga kwanu gawo nthawi ina iliyonse popanda chilango chilichonse. Mayankho anu pamafunso akafukufukuyi azasungidwa mwachinsinsi ndipo zonse zokuzindikiritsani zizasungidwa mosakuzindikiritsani nthawi zonse.

C. FAMILY PLANNING REIMBURSEMENT SERVICE:

Kulera kwa mahomoni (mchere wa nthupi), monga mapilisi akulera, kubaya jakiseni, kuika chapankono, ndi mankhawala omwe amachepetse chiopsezo chokhala ndi pakati. Njira zimenezi ndizothandiza kwambiri popewa kukhala ndi pakati, ndi anthawi yayitali, alibe vuto kugwiritsa ntchito kwa amayi ambiri. Pamene amayi ambiri sakumana ndi mavuto obwera chifukwa cha njirazi, amayi ochepa amapeza zovuta pogwiritsa imodzi mwa njirazi. Mavuto obwera chifukwa chogwiritsa ntchito kulera wa mahomoni ndi:

- Kunenepa
- Kuwawa kwa mutu
- Kuwawa kwa mawere
- Kusamba mosinthasintha
- Kusintha zochita (maganizo)
- Kuchepa kwa chilakolako chogonana
- Ziphuphu
- Nselu

Ngakhale kuti mavuto obwera chifukwa chogwiritsa ntchito njira zakulera amasiya okha pakapita miyezi mukugwiritsa ntchito njira yakulera, ndizofunikabe kwambiri kwa azimayi omwe akukumana ndi mavutowa kuonana ndi a dokotala ndikutsimikiza kuti njira yomwe akugwiritsa ntchitoyo ndiyabwino kwa iwo.

Utumiki omaliza omwe uzapelekedwe kwa inu ngati mbali imodzi ya pulogalamuyi cholinga chake ndikupititsa patsogolo ndi kulondoloza chisamaliro, chithandizo chamankhwala chokwanira, chisamaliro panthawi yomwe mwakumana ndi mavuto pogwiritsa ntchito njira yakulera.

Ngati mbali ya utumikiwu, mudzabwezeredwa ndalama zanu zochotsa m'thumba zomwe mwagwiritsa ntchito polandira chisamaliro chazakulera kuchipatala cha Good Health Kauma Clinic. Ndalama zolipira njira zakulera kuchipatala cha Good Health Kauma ndi:

- Zolipira zokhudzana ndikugula mankwala azakulera kapena njira yakulera
- Ndalama yopeleka kuti muonane ndi a dokotala pankhani zakulera
- Kulipira kuyezedwa ngati muli oyembekezera ndikulipira zina zokhudzana ndi kulera ku labotale (lab)
- Kulipira kuyezedwa zokhudzana ndikugwiritsa ntchito njira yakulera

Ndalama zanu zobwezeredwa zonse pamodzi ndi **17,500 MKW,** mudzabwezeredwa pamaulendo angapo opita kuchipatala cha Good Health Kauma pokalandira utumiki okhuza kulera pafupifupi kwa mwezi umodzi. Pautumiki ulionse wazakulera omwe muzalandire kuchipatala cha Good Health Kauma, zomwe mwalipira zidzachotseredwa ku ndalama zanu zobwezeredwa za 17,500.

Mukafika ku chipatala:

1. Mudzalandiridwa ndi mtsogoleri (manager) wa pulogalamu yemwe akugwira ntchito tsiku limeneli, yemwe azakuthandizeni munjira ina iliyonse yomwe mukufuna kuti mulandire chithandizo choyenera kuchipatalako.
2. Mukangoti mwaonedwa ndi a dokotala ndipo mwalandira chithandizo chomwe mumafuna,mtsogoleri wa pulogalamuyi adzafunsa a dokotala ndi ogwira ntchito pachipatalapo kuti adziwe za:

a. chithandizo chomwe mwalandira;

b. ngati chithandizo chomwe mwalandira chinali chokhudzana ndi mavuto obwera chifukwa chogwiritsa ntchito njira zakulera kapena chithandizo cha zina zilizonse zokhudzana ndi kulera ndiponso;

c. kuti zakwana ndalama zingati polandira chithandizo chazakulera.

3. Kutsatira kuonana ndi a dokotala, cholipira chilichonse chomwe chilipo polandira chithandizo chokhudzana ndikulera zidzalipiridwa ndi pulogalamu. Zomwe pulogalamu idzalipire ndi monga:
   a. Kuonana ndi a dokotala zokhudzana ndikulera komanso kulipira kuyezedwa
   b. Kulipira zokhudzana ndikugula mankhwala akulera ndi zinthu zina zokhudzana ndi kulera
   c. Kulipira mankhwala, zochitika zachipatala, kuyezedwa ku labotare (lab), kuonana ndi a dokotala ndi maulendo otsatira achithandizo komanso chisamaliro cha mavuto okhudza kugwiritsa ntchito njira zakulera

Ndondomeko zoonjezera ndi zenizeni zomwe mungagwiritsire utumiki obwezeredwa ndalama zili motere:

Key Terms and Conditions

1. Utumiki okulipirani pazakulera ndi oti **mugwiritse ntchito inu nokha** – palibe wina aliyense wapakhomo panu angagwiritse ntchito utumikiwu.
2. Mutha kugwiritsa ntchito utumiki wa taxi olipiliridwa pazakulerawu maulendo mmene mungafunire panthawi ya pulogalamuyi.
3. **Zolipira zokhazo zokhudzana ndikulandira chithandizo chazakulera, njira yakulera, ndi chisamaliro cha mavuto okhudza kugwiritsa ntchito njira zakulera zidzalipiridwa ndi utumiki umenewu.** Zolipiliridwazo ndi monga**:** kulipira mankhwala, kulipira njira yakulera, kuyezedwa, kuyezedwa ku labotare zokhudzana ndi chisamaliro chazakulera ndi chithandizo; kulipira zoonjezera poonana ndi a dokotala pachipatalapo pazakulera; kulipira chithandizo cha mavuto okhudza kugwiritsa njira zakulera; ndipo kulipira kusintha kapena kusiya njira yakulera. **Zolipira zina kuchipatala, monga izi( ): kulipira kulandira chithandiza cha sikelo yapakati ndi yamwana, kulipira zokhudza pakati, kulipira chithandizo cha malungo kapena matenda ena; kulipira chithandizo cha HIV/AIDS kapena matenda ena opatsirana pogonana; ndi zolipira zina zonse pa utumiki wina kapena ndondomeko zina zomwe sizinafotokozedwe sizidzalipiridwa mu pulogalamu ino.**
4. **Ndalama zonse pamodzi yoti mukhonza kudzalipiridwa** pamaulendo onse olandira chisamaliro chazakulera kuchipatala cha Good Health Kauma panthawi ya pulogalamuyi ndi **17500 MWK.** Ndondomeko ya maulendo a chisamaliro chazakulera sazagwira ntchito pa chisamaliro chazakulera cha nthawi ndi nthawi chilichonse cholandiridwa kuchipatala china.
5. Utumiki olipiliridwa pachithandizo cholipira mavuto obwera chifukwa cha njira zakulera adzagwira ntchito pa **njira zonse zakulera** posatengera kuti njirayo inatengedwa kuti mu Lilongwe.

6. Mukupemphedwa kusunga ma lisiti alionse okhudzana kulipira njira zakulera zomwe munatenga ndipo mukufuna kuti mubwezeredwe ndalama zanu. Zolipira zimenezi ndi monga mankhwala, kuonana ndi a dokotala, ndi zina zokhudzana ndi utumukiwu. Mukuyenera kulumikizana ndi mtsogoleri (manager) wa pulogalamuyi ndikupeleka malisitiwo. Mtsogoleriyu tsopano adzasanthula bwinobwino malisitiwo ndikuona ngati zolipiridwazo zili zoyenera kubwenzeredwa ndalama kapena ayi. Kubwezeredwa kwa ndalama zonse zazimene zinalipidwa zoyenera zidzapelekedwa ngati nkotheka mwansanga.
7. Pamapeto paulendowu, mtsogoleriyu adzakufunsani mafunso okhudza ulendowu ndi azatolera ndemanga ndi zochitika za mautumiki omwe munapatsidwa. Azatoleranso ndemanga zilizonse zokhudza utumiki wa taxi ndicholinga chofuna kupititsa patsogolo utukimiwu mtsogolo muno. Kutenga gawo mukafukufukuyi ndimwakufuna kwanu, mutha kukana kutenga nawo gawo kapena kuthetsa kutenga kwanu gawo nthawi ina iliyonse popanda chilango chilichonse. Mayankho anu pamafunso akafukufukuyi azasungidwa mwachinsinsi ndipo zonse zokuzindikiritsani zizasungidwa mosakuzindikiritsani nthawi zonse.

NTHAWI YA PULOGALAMU:

Pulogalamuyi itenga mwezi umodzi. Choncho ndinu oyenera kulandira utumiki ulionse omwe ndatchula mmwambamu kuyambira lero ndi mtsogolo muno kwa nthawi ya mwezi umodzi.

KUTENGA GAWO MU PULOGALAMU:

Kutenga nawo gawo kwanu mu pulogalamu imeneyi ndi kosakakamiza konse – ndi chisankho chanu kutenga kapena kusatenga nawo gawo mu pulogalamuyi. Ngati musankhe kutenga nawo gawo, mutha kusintha maganizo anu nthawi ina iliyonse kuti simutenga nawo gawo. Mukatuluka mu pulogalamuyi, mutha kudzalowanso mu pulogalamuyi nthawi ina mtsogolo popanda kulipiritsidwa chilichonse mu mwezi umodzi wa pulogalamuyi. Kukana kapena kusiya kutenga nawo gawo kulibe chilango chilichonse kapena kuluza zabwino zomwe muli oyenelera.

COST OF ENROLLMENT:

Palibe cholipira chilichonse kuti mulowe kapena kutenga nawo mu pulogalamuyi. Mwina munjira ina, koma mautumiki onse omwe atchulidwa ngati mbali imodzi ya pulogalamuyi akupelekedwa mwaulere.

RISKS:

Sitikuyembekezera choopsa chilichonse pakutenga nawo gawo mu pulogalamuyi.

ALTERNATIVES TO PARTICIPATION:

Njira ina yosatenga nawo mbali mu pulogalamuyi ndikusatenga nawo mbali. Kutenga nawo gawo mu pulogalamuyi ndikosakakamiza konse, ndipo mutha kukana kutenga nawo mbali kapena kutuluka mu pulogalamuyi popanda chilango. Mukatuluka mu pulogalamuyi, mutha

kudzalowanso mu pulogalamuyi nthawi ina mtsogolo popanda chilango mu mwezi umodzi wa nthawi ya pulogalamuyi.

KODI KUTENGA MBALI KWANGA KUKHONZA KUTHA MSANGA?

Mutha kupanga chiganizo chosapitiliza kutenga nawo gawo mukafukufukuyi nthawi ina iliyonse popanda chilango chilichonse. Mutha kutuluka pondidziwitsa ine kapena mmodzi ogwira ntchito mu pulogalamuyi kuti simukufuna kupitiliza kutenga nawo gawo (simuzafunsidwa funso lililonse). Muthanso kudumpha funso lililonse nthawi ya kafukufuku, koma kupitiliza kutenga nawo gawo mukafukufuku yense. Ngati mwaganiza zosiya pulogalamuyi, chonde lumikizanani ndi membala ogwira ntchito mu pulogalamuyi.

Zina Zokhudza Pulogalamu:

Khadi Lokudzindikiritsani mu Pulogalamu

Mulandira khadi yanuyanu yokuzindikilitsani mu pulogalamuyi yomwe **idzathandize ogwira ntchito mu pulogalamuyi kukuzindikirani kuti ndinu oyenera mu pulogalamuyi**. Mukuyenera kuonetsa khadi yanu ya mupulogalamu nthawi iliyonse yomwe mukufuna kugwiritsa ntchito utumiki wathu, monga:

- Musanapatsidwe uphungu
- Mukafuna kugwiritsa ntchito utumiki wa taxi
- Ngati mukufuna kufunsa a dokotala pa phone

Chonde sungani khadiyi pabwino, ndipo onetsetsani kuti mwatenga khadi lanu. Mmene mukuonera pakhadi lanulo, mwapatsidwa nambala yanuyanu yokuzindikilitsani mupulogalamu.

Chonde kumbukirani nambala yanu yokuzindikilitsani mupulogalamu – izathandiza ogwira ntchito mupulogalamuyi kutsimikiza pokudzindikirani mutati mwataya khadi. Mudzapemphedwa kutchulanso nambala yokuzindikilitsaniyo mutati mwaimbira phone aliyense ogwira ntchito mupulogalamuyi.

Kubwezeretsa Khadi Lotayika kapena Loonongeka:

Ngati mwataya kaya khadi lanu laonongeka, chonde dziwitsani mtsogoleri (manager) wapulogalamuyi pa phone msangamsanga kuti akupangireni lina. Mukaimba, mtsogoleriyu (manager) azakufunsani za nambala yanu ya mupulogalamu (ID) komanso azakufunsani mafunso ndicholinga choti akuzindikireni. Mukatero mudzalandira khadi latsopano pakati pamasiku atatu ndi asanu.

Maunitsi oimbira phone:

Pamapeto pa uphungu, mulandira zinthu zomwe mungagwiritse ntchito pa nthawi ya zadzidzidzi zomwe ndi maunitsi a phone okwana 100 kwacha a TNM kapena AIRTEL malinga ndikukonda kwanu. Zinthu zimenezi mukuyenera kugwiritsa ntchito pa kuimbira ma phone (ngati mukufuna kulankhulana ndi a dokotala, kuimbira wa galimoto) ndi zinthu zina zonse. **Mayunitsiwa ndi**

anu ngakhale mutasankha kusatenga nawo gawo mu pulogalamuyi, mutafuna thandizo lililonse mu pulogalamuyi kuchokera kwa ife kapena mutakumana ndi zovuta zobwera chifukwa cha njira yakulera.

Zolumikizana ndi Anthu a mu Pulogalamu:

Zonse zokhudza mmene mungalumikizilane ndi anthu ofunikira mupulogalamuyi zaikidwa mmunsimu ndipo zidzapelekedwanso kwa inu mu foda yamalonje.

Kulumikizana ndi dalaivala wa Taxi

Mudzapatsidwa dzina, phone nambala, ndi mmene mungaapezere a dalaivala omwe azidzapeleka utumiki wa mayendedwe. Dalaivala azipezeka kuti akutengeni kupita kuchipatala cha Good Health Kauma nthawi yomwe chipatala chimakhala chotsekula pakati pa **8 koloko mamawa ndi 5 koloko madzulo kuyambira lolemba mpaka lachisanu**. Mukuyenera kukambirani ndi a dalaivala patatsala tsiku limodzi lisanafike tsiku lanu lopita kuchipatala ndipo adzakudziwitsani ndondomeko yawo ndi kupezeka kwawo. Ngati mwakumana ndi vuto ladzidzidzi lokhudzana ndikulera ndipo simukupeza njira yamayendedwe okafikira kuchipatala chapafupi, mutha kuwaimbiranso adalaivala.

Kulumikizana ndi Mtsogoleri wa pulogalamu

Mutakhala ndi mafunso, madandaulo okhudza pulogalamuyi kapena mautumiki omwe muli oyenerezeka, chonde musachedwe kulumikizana ndi mtsogoleri (manager) wa pulogalamuyi. Osalephera m'modzi azapezeka panthawi iliyonse. Manambala a phone ndi mmene mungaapezere zapelekedwa kwa inu.

KUTETEZEDWA KWA CHINSINSI:

Zokuzindikiritsani ndi zonse zomwe muyankhe zizasungidwa mwachinsinsi. Palibe nthawi ina iliyonse yomwe zenizeni za inu zidzauluridwe kwa munthu yemwe simmodzi mwa anthu amupulogalamuyi. Mayankho anu onse adzakhala ndi dzina lanu, ndipo dzina lanu lapatsidwa nambala yotola mongosakaniza. Aliyense yemwe akupanga (staff) nawo pulogalamuyi azadziwa za inu ndi nambalayi basi. Sitizagwiritsa ntchito dzina lanu kapena zokhudza inu zomwe zizakuzindikilitseni pazofalitsa. Dzina lanu ndi zonse zokuzindikilitsani zidzasungidwa mwachinsinsi.

MAFUNSO KAPENA NKHAWA?

Ngati muli ndi mafunso kapena nkhawa zokhudza pulogalamuyi, chonde lumikizanai ndi **Dr. Bagrey Ngwira**, amene ali oyang'anira pulogalamuyi.

Dr. Bagrey Ngwira
Innovations for Poverty Action (IPA) Malawi
E-mail: bagreyngwira@gmail.com
Telephone: +265 999554003, +265 888554003

Availability: Monday to Friday, 9 AM to 5 PM

Kafukufukuyu waunikidwa ndi a Malawi National Health Sciences Research Committee (NHSRC). Ngati mungakonde kulankhulana ndi wina waku NHSRC, chonde lumikizanani nawo pa: Ministry of Health, P.O. Box 30377, Lilongwe 3, Malawi, kapena pa phone nambala iyi +265 1 726 422/418, kapena pa e-mail iyi mohdoccentre@gmail.com pa china chilichonse mwazotsatirazi:

- Ngati mafunso anu, nkhawa, kapena zidandaulo zanu sizinayankhidwe ndi timu yakafukufuku,
- Ngati mukulephera kulumikizani ndi timu yakafukufuku,
- Ngati mukufuna kulankhula ndi wina osati timu yakafukufuku,
- Ngati muli ndi mafunso okhudza ufulu wanu ngati otenga nawo gawo pakafukufuku, kapena
- Ngati mukufuna kudziwa zambiri kapena kupeleka maganizo anu okhudza kafukufukuyi.

MAU A KUVOMERA

Ndawerenga zonse mukalata yachilolezoyi kuphatikizapo chiopsezo komanso phindu. Mafunso anga onseokhudza kafukufukuyi ayankhidwa ndipo ndakhutira nawo. Ndamvetsetsa kuti ndili ndi ufulu wotuluka nthawi ina iliyonse popanda kulipitsidwa kapena kuluza mwayi omwe ndili oyenerezeka.

Ndikuvomera kutenga nawo gawo mupulogalamu ya uchembere ndi umoyo wabwino ya Innovations for Poverty Action (IPA) Malawi.

KUSAYINA

TO WOMAN:
Kusayinila kwanu pansipa kukusonyeza kuvomereza kwanu kutenga nawo gawo mukafukufukuyi.

____
Name of participant

________________________________ ________________
Signature / Thumbprint of participant Date

________________________________ ________________
Signature of person obtaining consent Date

____
Printed name of person obtaining consent

TO HUSBAND:
Your signature below indicates your permission to take part in this program

Name of woman’s husband

Signature / Thumbprint of woman’s husband

Date

Signature of person obtaining consent

Date

Printed name of person obtaining consent
