# Supplementary material for: User-Centered Counseling and Male Involvement in Contraceptive Decision Making: Protocol for a Randomized Controlled Trial
Source: JMIR Res Protoc. 2021 Apr 5;10(4):e24884. doi: 10.2196/24884 (PMC8056297; doi:10.2196/24884)
Supplement: Multimedia Appendix 2 [file resprot_v10i4e24884_app2.pdf]

KULERA

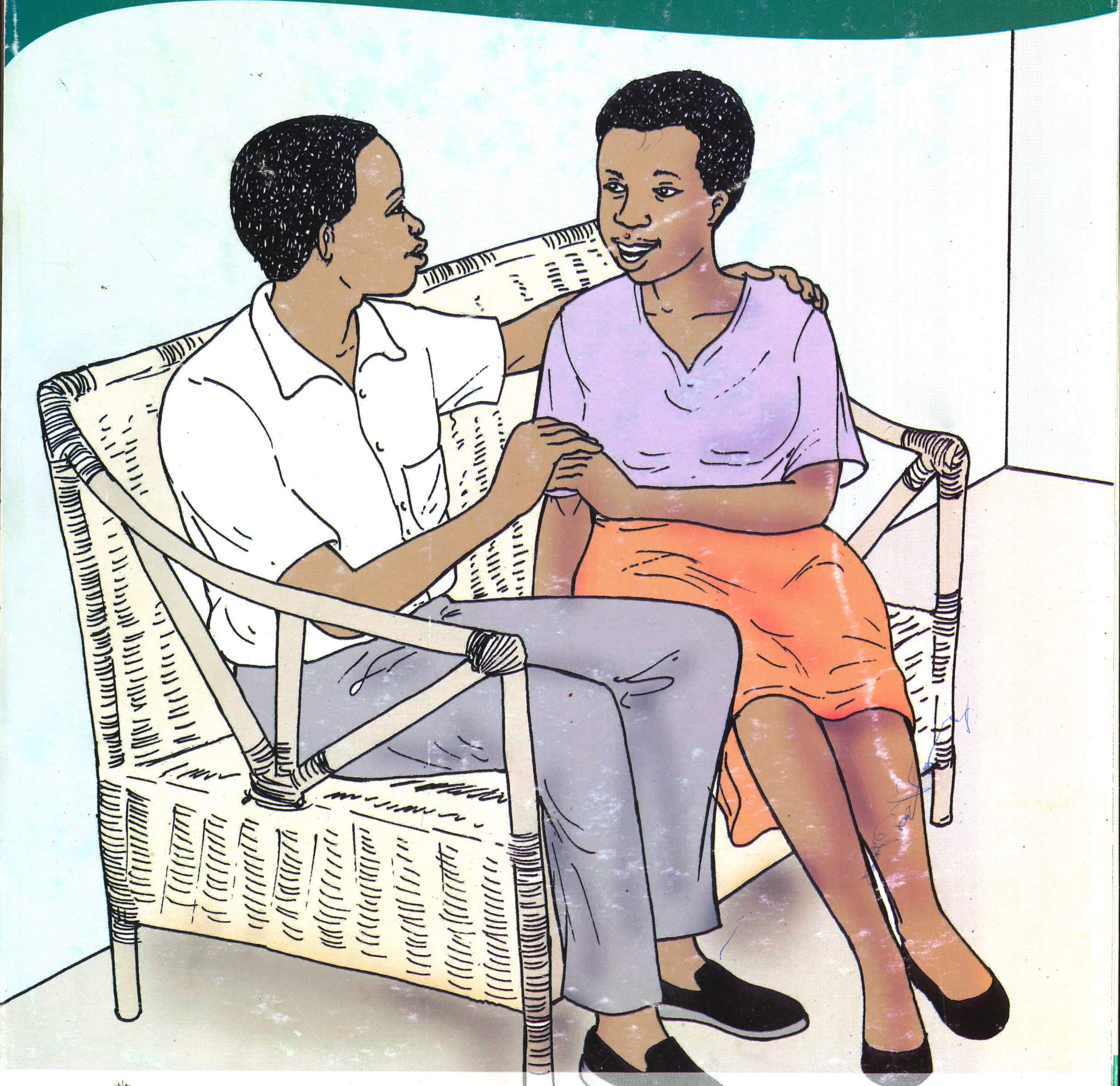

Kulera

Wina aliyense amene ali mumsinkhu wobereka (zaka 15-49) ali omasuka kuganizira za njira imene akhoza kugwiritsa ntchito kupewa mimba yosakonzekera

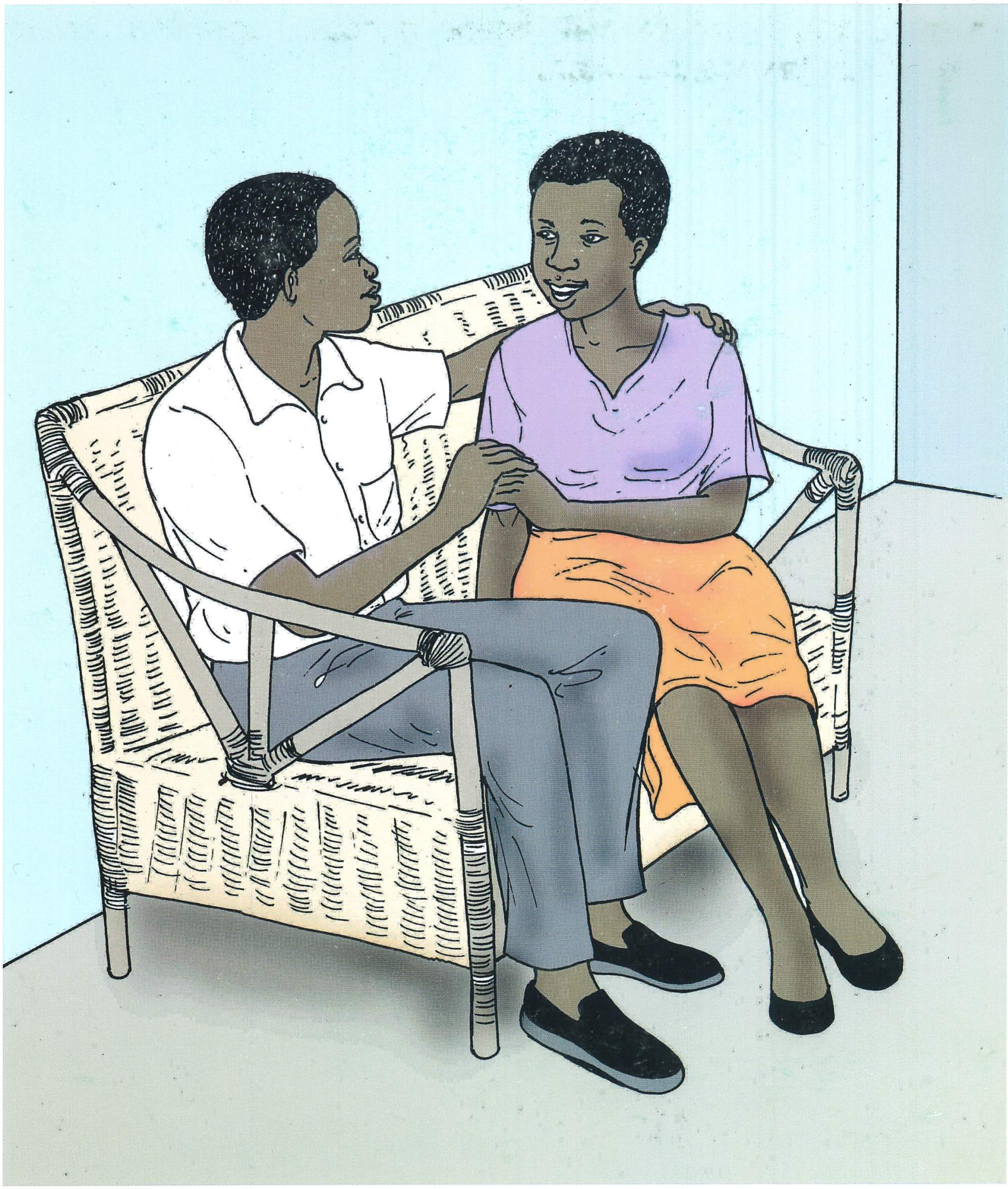

khadi 02

Kulera

A Phiri ndi akazi awo ndiwomasuka ndipo akukambirana za kulera

khadi
02

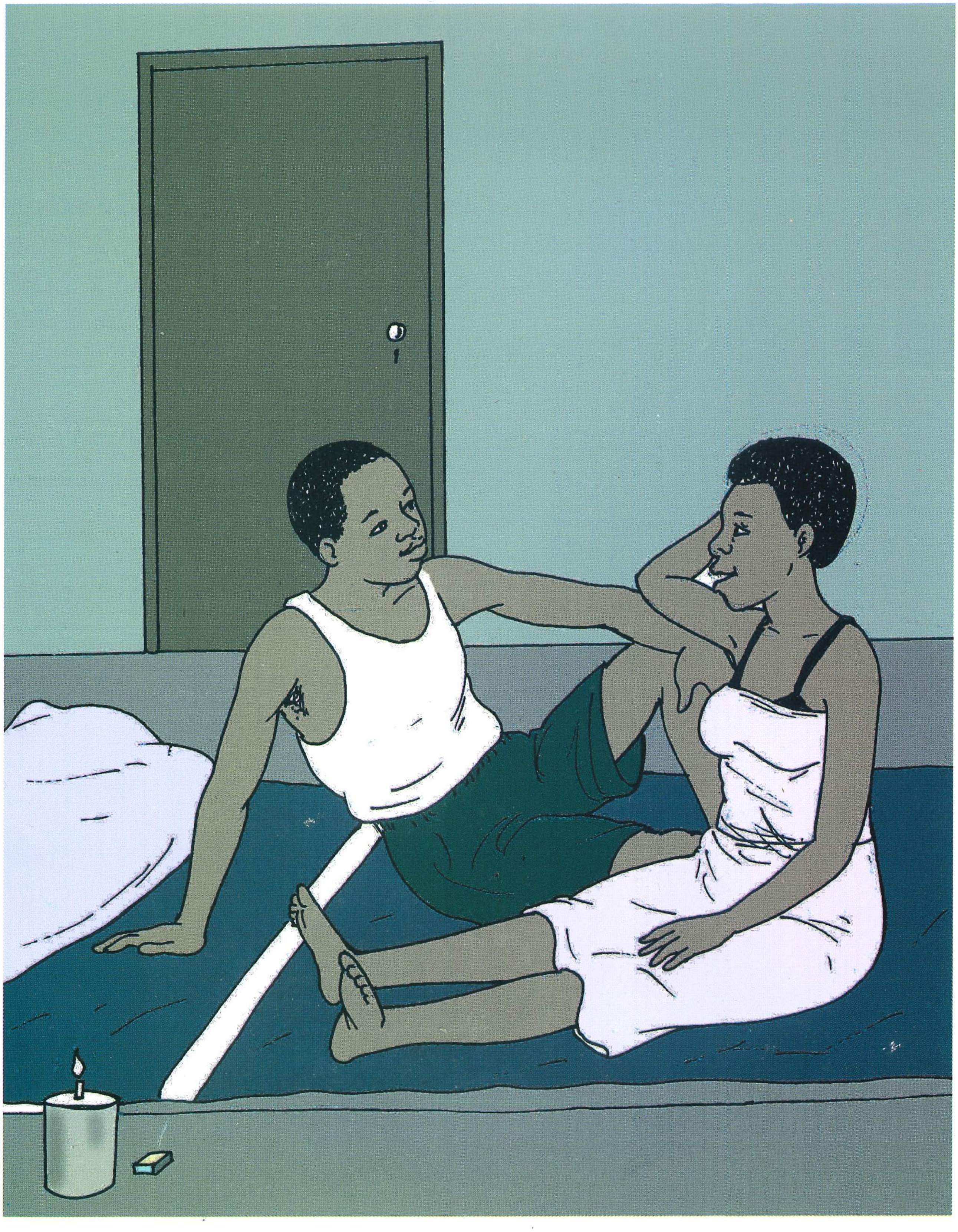

khadi
02

Kulera

Apa tikuona ziwalo za abambo zomwe zimapangitsa amuna kuti apeleke mimba. Ziwalozi ndi machende, mbolo ndi nthumba la mbewu ya abambo. Mbeu za abambo zimapangidwa mu machende. Umuna umapangidwa mu nthumba lo sungira mbewu ya abambo. Pamene bambo ndi mai akugonana mbewuyi imatuluka kuchokera ku machende kudzera mumtsempha ndipo imasakanizika ndi umuna womwe umachokera nthumba losungila mbewu ya abambo. Umuna wosakanikirana ndi mbewu ya abambo ukalowa mchibelekero ndikukumana ndi dzira la mayi, mayi athakutenga mimba pamene dzira lakhwima.

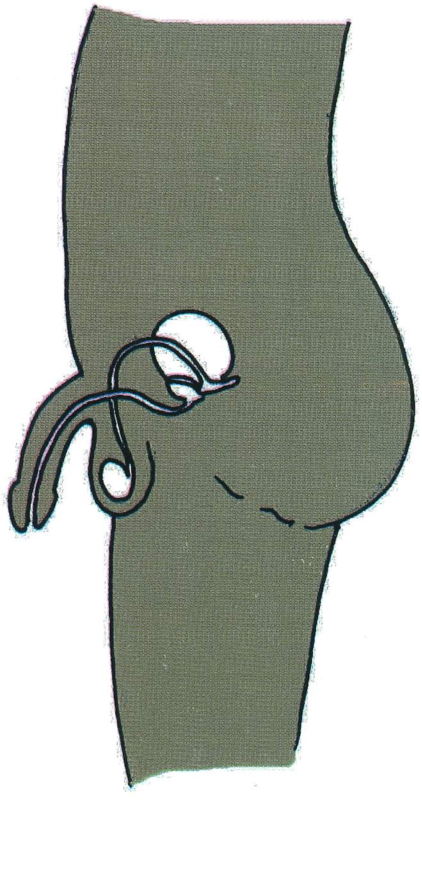

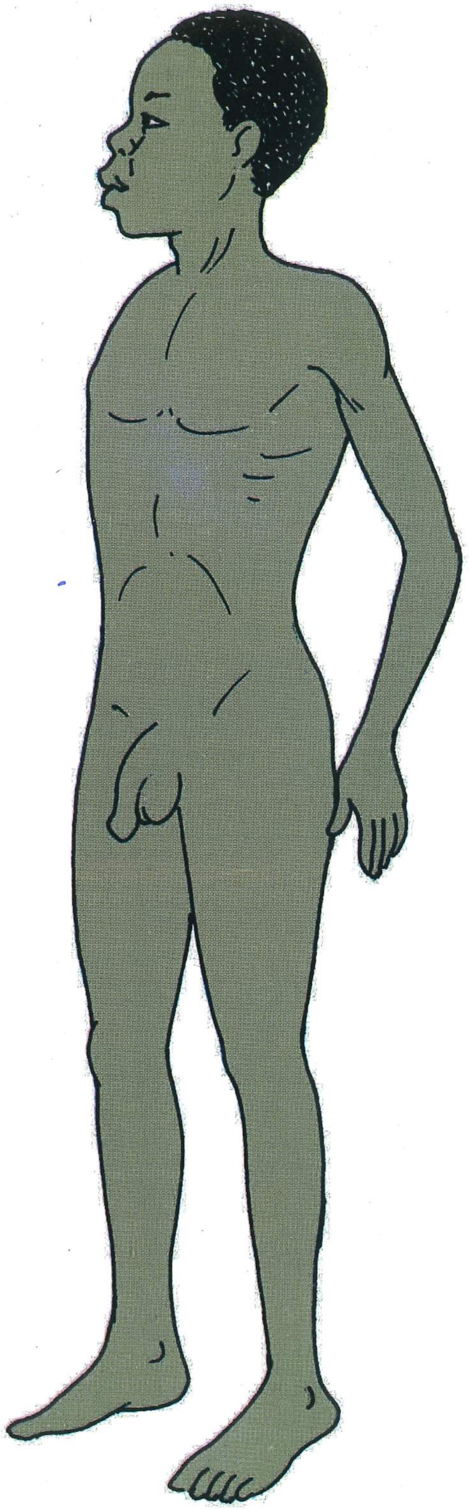

Kulera

Ziwalo za amayi zothandiza pobeleka ndi nyini, khomo la chibelekero, chiberekero, machubu odutsa mazira ndi mabasiketi a mazira.
Mwezi uliwonse basiketi imodzi imatulutsa dzira lokhwima lomwe limadutsa mu chubu kupita mu chibelekero; ndipo ngati dzira silipezana ndi mbewu ya abambo mayi amasamba. Msambo umachokera muchiberekero.

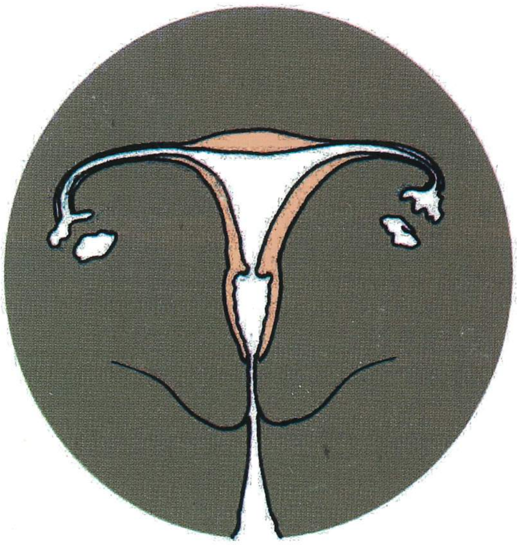

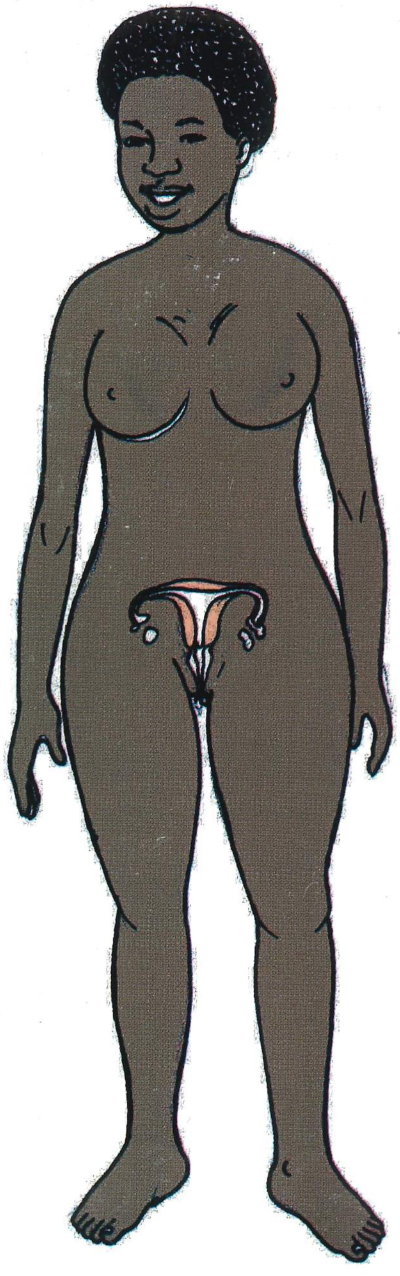

Kulera

Apa tikuwona ziwalo za mwamuna ndi mkazi. Nthawi yogonana, mbewu ya mwamuna imatuluka ndi kulowa muchibelekero ndipo imakafika m'machubu omwe mumadutsa mazira. Ngati mbewu ya mwamuna ilowa mu dzira la mkazi, dziralo limayenda nkukakhazikika muchiberekero m'mene limakula kukhala mwana.

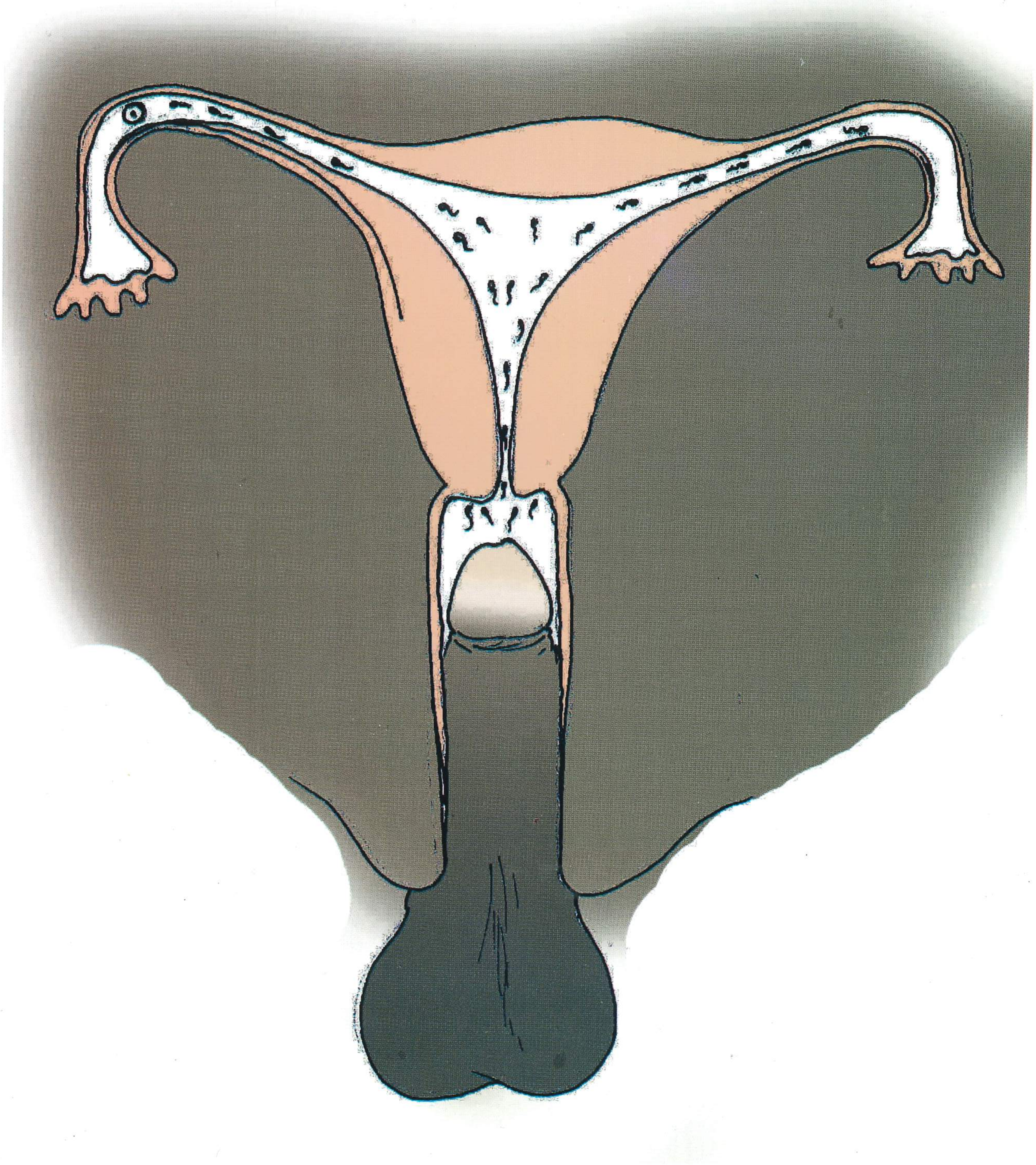

Kulera

Apa tikuona mkazi amene waikidwa lupu mchiberekero chake.

LUPU

Lupu ndi kapulasitiki kamene kamakhala ndi tizingwe tiwiri. Lupu imayikidwa mchibelekero koma zingwe zake zimakhala mu nyini ndipo pakapita masiku angapo zimafewa mwakuti mwamuna sapeza vuto lina lililonse akamagonana ndi amai. Njirayi imateteza amai kuti asatenge mimba kwa zaka khumi ,

Momwe imagwirira ntchito

Lupu imalepheretsa kuti mbewu ya mwamuna ndi dzira la mkazi zikumane choncho mayi sangatenge mimba.

Ubwino wake

Mkazi ndi mwamuna amagonana mosadera nkhawa kuti mkazi atenga mimba.

Mkaka wa m'mawere siuchepa kwa mkazi amene akuyamwitsa.

Kuvuta kwake

Zovuta za njirayi zomwe zimaoneka kwa amayi ena zimasiya pakatha miyezi itatu

- Amai ambiri amamva cham'mimba.
- Amasamba kawirikawiri.
- Msambo wawo umachuluka.,

Malangizo

- Ngati amai afuna kubeleka zaka khumi zisanakwane apite kuchipatala kuti akachotse lupuyo.
- Dziwani kuti palibe njira imene lupu ingadutse kuchoka m'muchibelekelo kupita ku ziwalo zina za mthupi la mkazi.
- Amai akaona vuto apite kuchipatala
- Dziwani kuti lupu simateteza kumatenda opatsirana pogonana ndi Tizirombo toyambitsa matenda a Edzi.

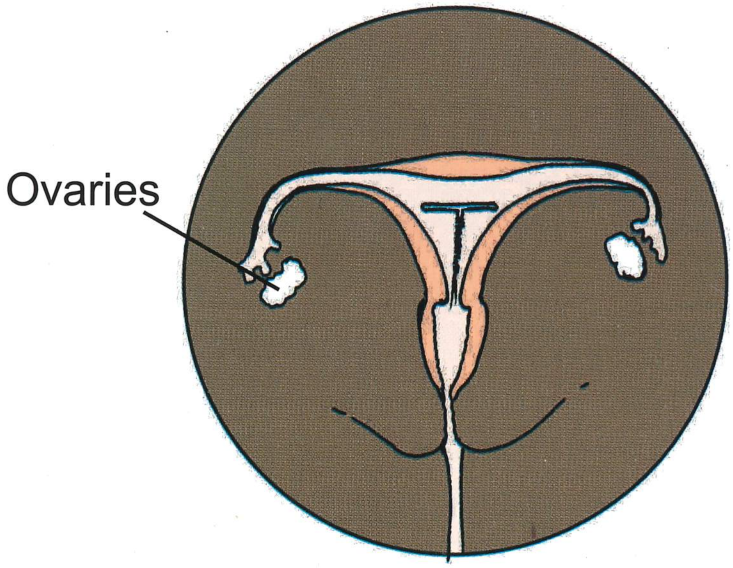
Ovaries

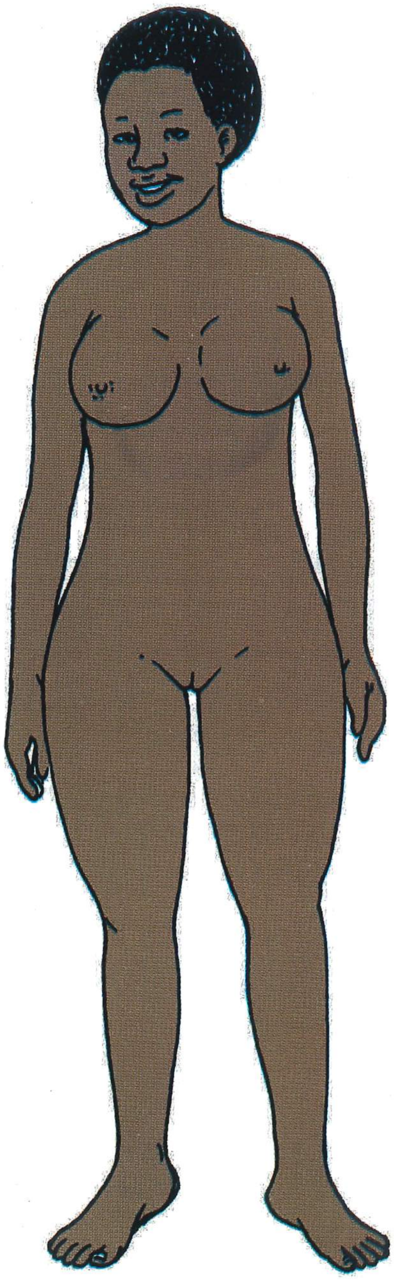

Kulera

Njira ya pa mkono (Impulanoni/Jadele)

Chithuzi choyamba chikuonetsa mkazi amene amuyika Impulanoni pamkono wake.

Chithuzi chachiwiri chikuonetsa mkazi amene amuyika jadele pamkono wake.

Impulanoni ndi jadele ndi njira ya pamkono yomwe imaikidwa pansi pa khungu ndipo timaoneka ngati timitengo ta machesi. Impulanoni ndi kamtengo kamodzi pamene Jadele ndi timitengo tiwiri. Akazi amakhala otetezedwa kutenga mimba kwa zaka zisanu akaikidwa jadele ndipo kwa zaka zitatu akaikidwa impulanoni,

Momwe imagwirira ntchito

Njira za pamkono (Impulanoni kapena Jadele) zimapangitsa kuti

- Dzira la mkazi lisakhwime.
- mkati mwa chibelekero mukhale mosakonzekera kulandira mwana
- Chikazi chikhale cholimba.

Ubwino wake

- Mwamuna ndi mkazi amagonana mosadera nkhawa yotenga mimba
- Mkazi amayamwitsa bwinobwino chifukwa mkaka wa m'mawere siuchepa.

Kuvuta kwake

- Akazi ena amasiya kusamba.
- Ena amasamba mowirikiza
- Ena amasamba modukizadukiza.

Malangizo

- Mkazi akafuna kubereka zaka zitatu kapena zisanu zisanakwane apite kuchipatala kuti akachotse njira ya pankono.
- Mkazi sachedwa kutenga mimba akachotsetsa njira ya pa mkono.
- Mkazi akaona vuto lililonse apite kuchipatala kuti akathandizidwe
- Dziwani kuti njira ya pa mkono simateteza kumatenda opatsirana pogonana ndi tizirombo toyambitsa matenda a Edzi.
- Dziwani kuti mphamvu ya njirayi imachepa ngati mkazi/mzimai akumwa mankhwala a ma ARV (5A ndi Nevirapine)

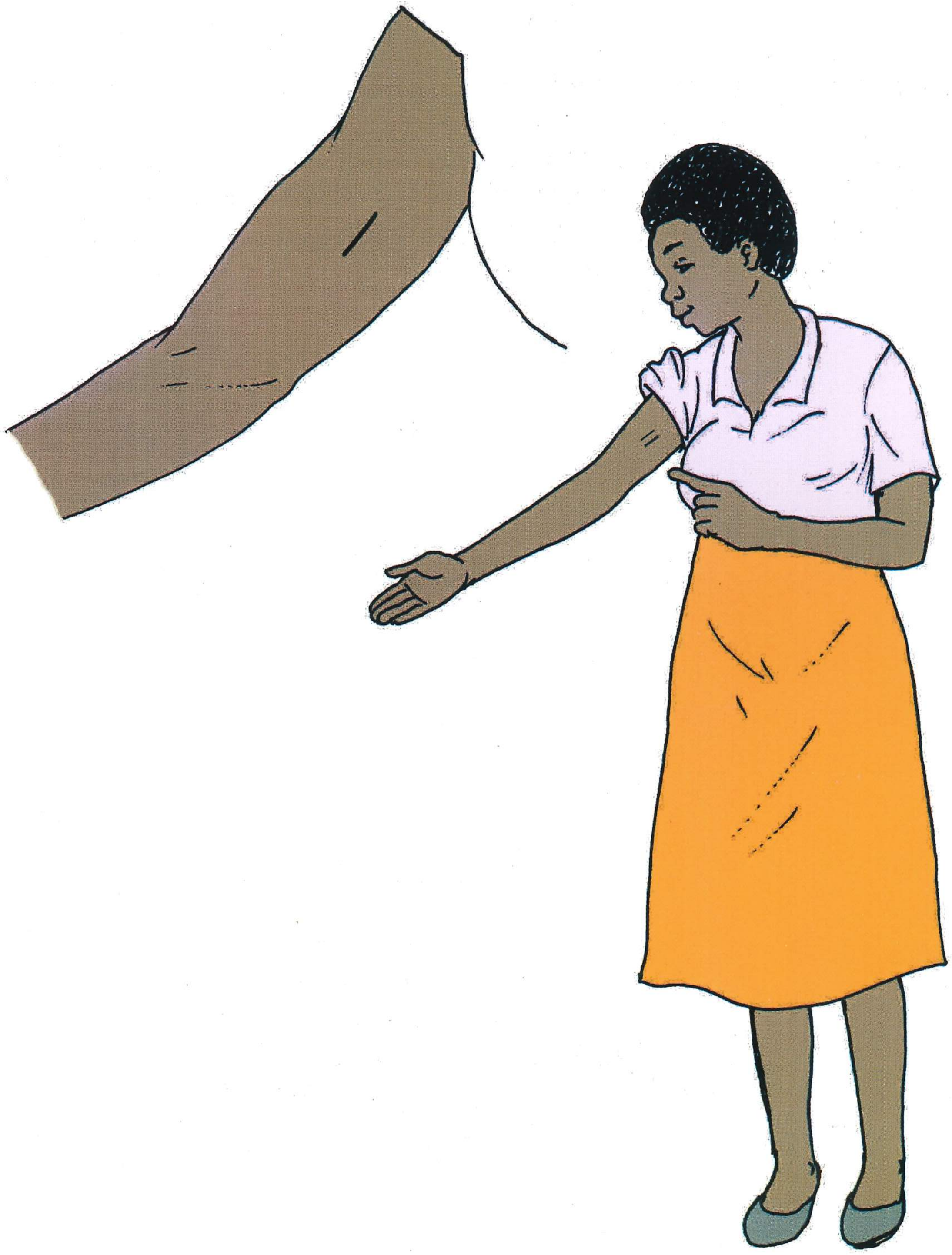

Kulera

KUTSEKA MWAMUNA

Chithunzi ichi chikuonetsa mwamuna amene watseketsa machubu wodutsa mbewu ya abambo.

Kutseka kwake

Kutseka abambo kumachitika ku chipatala. Adokotala amaboola khungu lamachende pang'ono ndikukowa machubu ndipo amawadula. Izi zimachitika mwamuna ali maso ndipo zimatenga mphindi zochepa. Mwamuna amabwerera kunyumba tsiku lomwelo.

Momwe imagwirira ntchito

- ✍ Mwamuna akatseketsa mbewu yake imalephera kudutsa mumachubu kuti ikalowe muchiberekero ndipo nthawi yomwe wagona ndi mkazi, mkaziyo sangatenge mimba.

Ubwino wake

Mwamuna ndi mkazi amagonana mosadera nkhawa kuti mkazi atenga mimba.

Kuvuta kwake

Njirayi ndiyoyenera mwamuna amene sakufuna kuberekanso , chifukwa akatseketsa sangathe kubelekanso.

Malangizo

- ✍ Mwamuna ngati afuna kugonana ndi mkazi akangotseketsa, aonetsetse kuti akugwiritsa ntchito njira yina monga kondomu kwa miyezi itatu kuti asapereke mimba.
- ✍ Mwamuna akatseketsa chilakolako chofuna kugonana ndi mkazi sichisitha ndipo mbolo yake imatota ngati kale.
- ✍ Akamagonana ndi mkazi amanva kukoma ndipo amatulutsa umuna koma simukhala mbewu imene ingapangitse mkazi kutenga mimba.

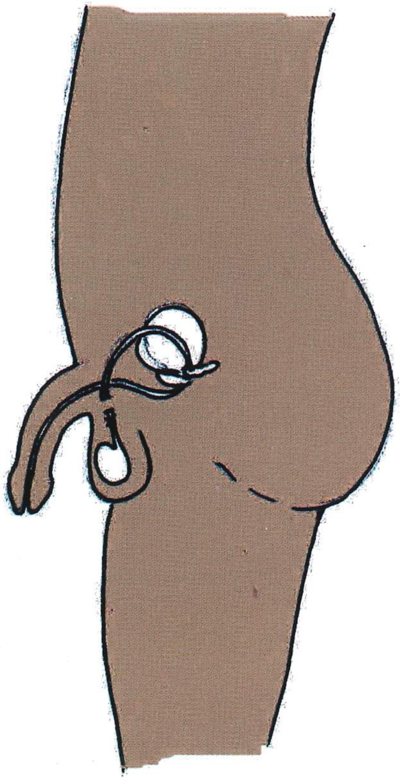

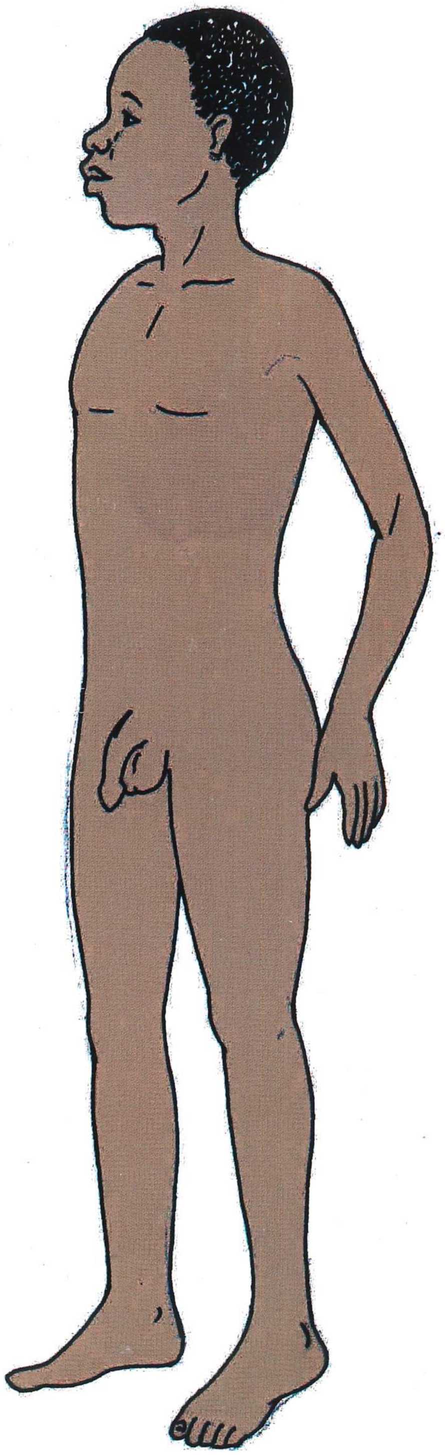

Kulera

KUTSEKA AMAYI
Pa chithunzi apa tikuona mayi amene watseketsa machubu odutsa mazira.

Kutseka kwake
Kutseka mayi kumachitika kuchipatala. Adokotala amacheka pang'ono pamimba ndipo amakowa machubu odzera mazira. Akatero amamanga ndikudula machubuwo. Izi zimachitika mayi ali maso ndipo amabwerera kunyumba tsiku lomwelo. Njirayi ndiyoyenera amayi amene sakufuna kuberekanso .

Momwe imagwirira ntchito
Mbewu ya mwamuna ndi dzira la mayi sizikumana ndipo mayi sangatenge mimba.

Ubwino wake
Mwamuna ndi mayi amagonana mosadera nkhawa kuti mayi atenga mimba.

Kuvuta kwake
Mayi akatseketsa samaberekanso.

Malangizo
Oyenera kugwiritsa ntchito njirayi ndi mayi amene:
- Wakwanitsidwa ndi ana amene ali nawo
- Ali ndi mavuto ena ndipo adokotala anamuuza kuti asadzabelekenso

Chidziwitso
Mayi akatseketsa amasamba mwezi uliwonse ndipo palibe chomwe chimasintha mthupi mwake komanso chilakolako ndi kukoma pogonana sikusintha.

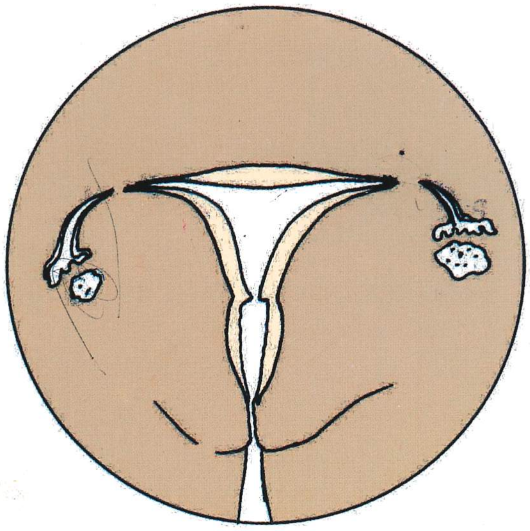

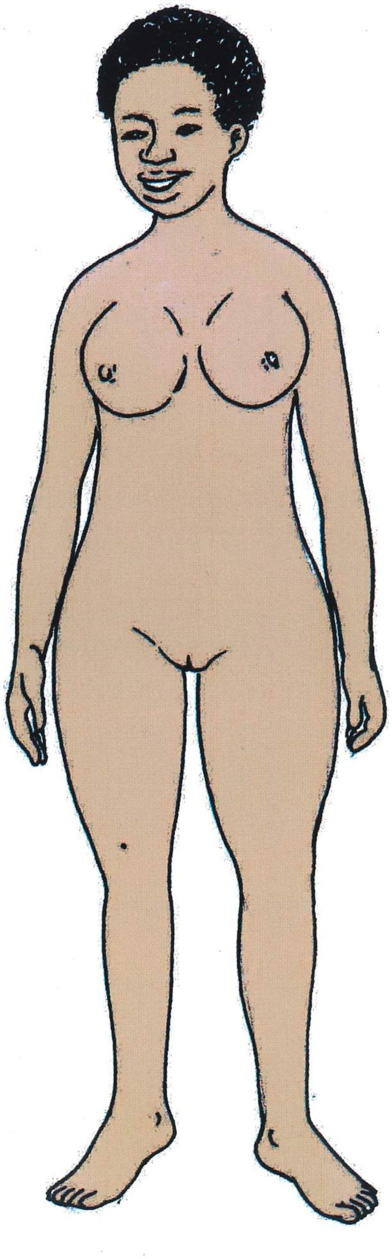

Kulera

Njira yolerera ya pa ngozi
Apa tikuona mkazi amene akugwiritsa ntchito mapilitsi akumwa pamene wagonana ndi mamuna mosaziteteza pofuna kupewa mimba.

Momwe imagwirira ntchito
Mapilitsi amapangitsa kuti
- Ddzira lisakhwime.
- Mkati mwa chiberekero musakonzekere mimba.
- Chikazi chikhale cholimba.

Ubwino wake
- Njirayi ndi yodalilika kwambiri
- Mayi amapewa kutenga mimba pamene wagonana ndi mwamuna
- mosayembekezera monga kugwiriridwa.

Kuvuta kwake
- Ena amamva nseru ndipo nthawi zina ena amasanza
- Imayenera kugwiritsidwa ntchito pasanathe masiku asanu
- Simateteza ku matenda opatsirana poganana kuphatikizipo
- tizilombo toyambitsa matenda a EDZI

Malangizo
- Mayi asankhe njira ina yolerera akagwiritsa ntchito njirayi.
- Mkazi abwerenso kuchipatala ngati:
  - Msambo ukubwela wochepa kwambiri (mwina ali ndi mimba)
  - Msambo wake siukubwera monga mwa masiku onse
  - Ngati msambo siukuyamba mkati mwa masabata anayi (mwina ali ndi Mimba)
  - Akumva kuwawa m'mimba (mwina ali ndi mimba ya muchubu).

CHIDZIWITSO:
Fotokozerani kuti njirayi ndi yolerera pa ngozi osati nthawi zonse
Komanso afotokozereni kuti njirayi siingachotse mimba.

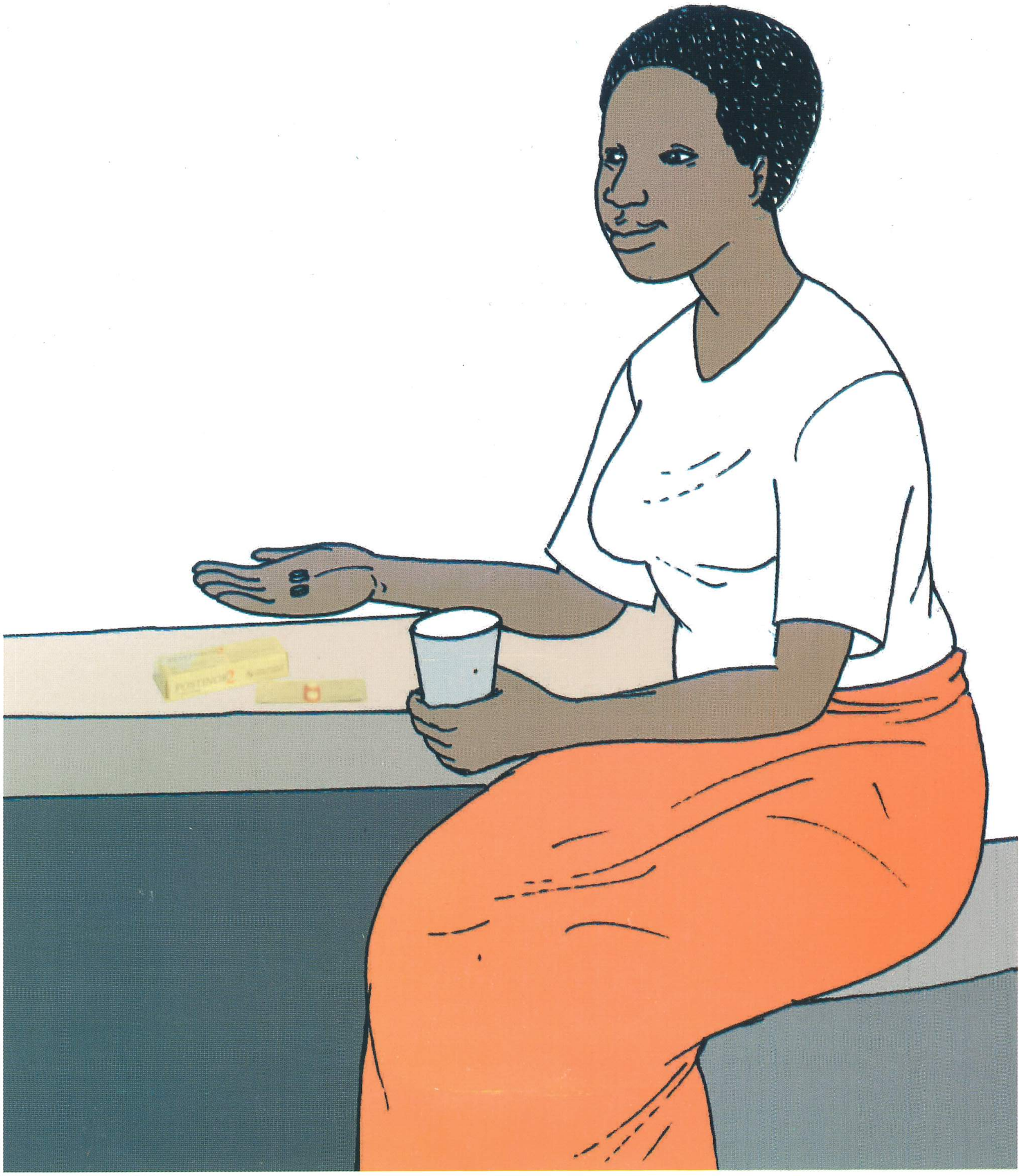

Kulera

Apa tikuona chithunzi cha amayi akumwa mapilitsi.

MAPILITSI

Mapilitsi ndi njira imodzi yomwe akazi amagwiritsa ntchito akafuna kulera. Mapilitsi alipo amitundu iwiri:- mapilitsi a mphamvu imodzi amamwa mayi oyamwitsa mwana amene asanakwane miyezi isanu ndi umodzi chifukwa samachepetsa mkaka wa m'mawere.

Mapilitsi a mphamvu ziwiri amamwa mayi wina aliyense koma mayi oyamwitsa ayambe pamene mwana wakwana miyezi isanu ndi umodzi chifukwa amachepetsa mkaka wa m'mawere.

Momwe amagwirira ntchito

- ✍ Mapilitsi amapangitsa kuti dzira lisakhwime.
- ✍ Mkati mwa Chibelekero mukhale mosakonzekera kulandira mwana.
- ✍ Chikazi chikhale cholimba.

Ubwino wake

- ✍ Amuna ndi akazi amagonana mosadera nkhawa yotenga mimba.
- ✍ Akazi ambiri amasamba masiku ochepa ndipo sakhala ndi vuto lochepa magazi mthupi.
- ✍ Amachepetsa cham'mimba mwa akazi ambiri.

Kuvuta kwake

Akazi ena amamva

- ✍ Nseru.
- ✍ Chizungulire.
- ✍ Kuwawa kwa mutu (litsipa).
- ✍ Kuwawa kwa mawere.

Ena amasamba modukizadukiza, amasiya kusamba, kapena amasamba mowirikiza. Nthawi zambiri izi zimasiya pakatha miyezi itatu.
Imachepetsa mkaka wa m'mawere kwa akazi amene akumwa mapiritsi a mphamvu ziwiri.

Malangizo

- ✍ Mkazi amwe pilitsi tsiku ndi tsiku mpaka nthawi imene akufuna kukhala ndi mwana.
- ✍ Mkazi amwe pilitsi madzulo pogona chifukwa ndi pamene angakumbukire kumwa pilitsilo.
- ✍ Ngati awona zodabwitsa zirizonse apite kuchipatala kuti akaonedwe.
- ✍ Dziwani kuti mapilitsi akulera samateteza ku matenda opatsirana Pogonana ndi tizirombo toyambitsa matenda a Edzi.

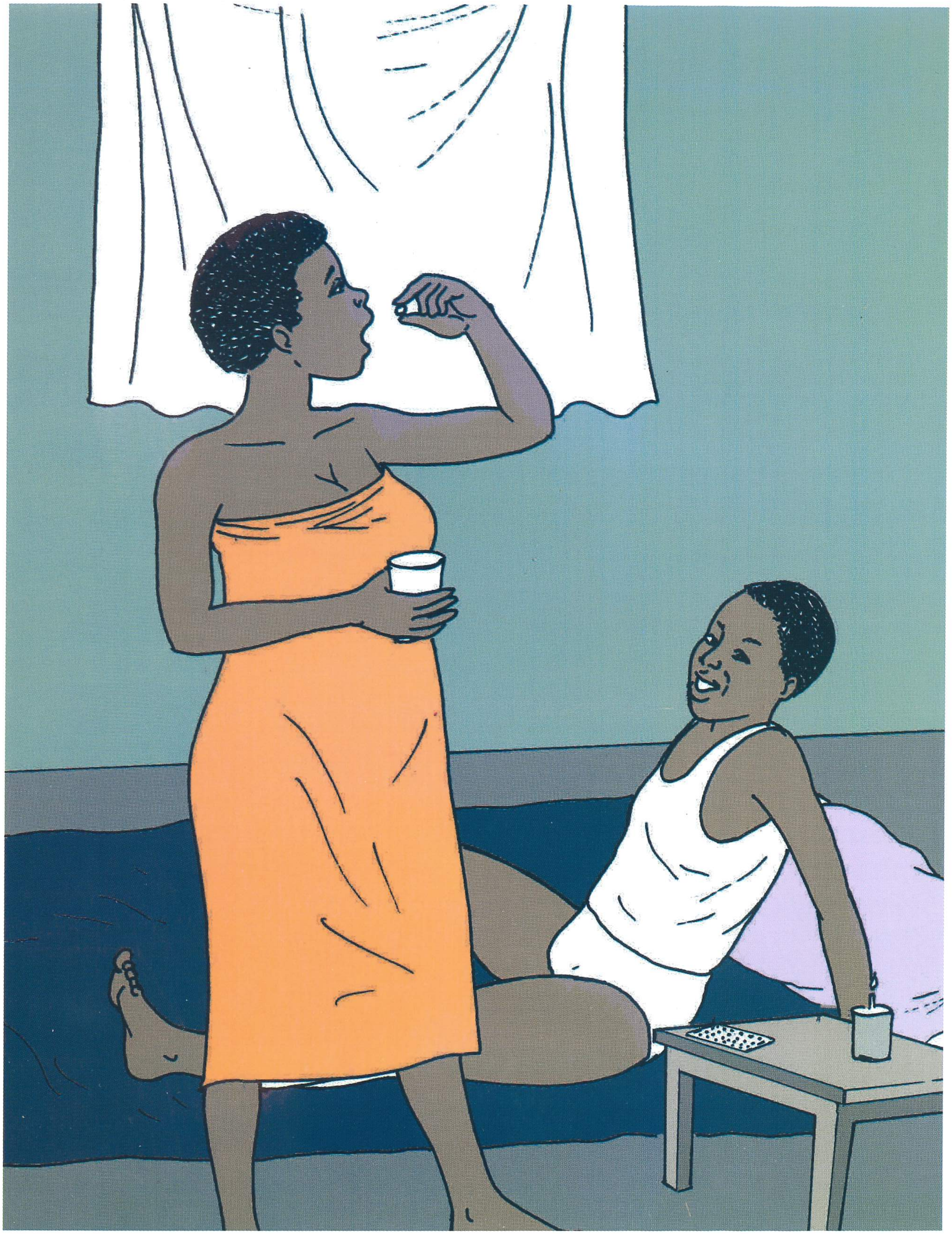

Kulera

Apa tikuona mayi akubayitsa jakisoni yolelera.

JAKISONI

Jakisoni ndi njira imodzi yomwe akazi amagwiritsa ntchito akafuna kulera.

Amayi amalandira njirayi kamodzi pa mwezi,miyezi iwiri kapena miyezi itatu iliyonse molingana ndi mtundu wa mankhala.

Momwe imagwirira ntchito

- Amapangitsa kuti dzira lisakhwime.
- Mkati mwachiberekero mkhale mosakonzekera kulandira mwana
- Chikazi chikhale cholimba.

Ubwino wake

- Amuna ndi akazi amagonana mosadera nkhawa yotenga mimba.
- Kuchuluka ndi kutuluka kwa mkaka wa m'mawere sikumasintha ngati mayi aku bayitsa jakison wa mphambvu imodzi.
- Njirayi ndiyodalirika chifukwa mwa amai 100 amai 98 amatetezedwa ku mimba.

Kuvuta kwake

- Amayi ena amasamba modukizadukiza.
- Ena amasiya kusamba ndipo ena amasamba mowirikiza (izi zimasintha pakapita miyezi itatu).
- Akazi akaleka kubayitsa jakisoni, mphavu yake imapitirira kwa masabata anayi kapena chaka chimodzi osatenga mimba (izi zimachitika mosiyanasiyana malingana ndi kusiyana kwa matupi).

Malangizo

- Akazi aonetsetse kuti akulandira jakisoni kamodzi pa mwezi,pa miyezi iwiri kapena pa miyezi itatu iliyonse.
- Ngati awona zodabwitsa ziri zonse apite kichipatala kukaonana ndi anamwino.
- Dziwani kuti jakisoni simateteza ku matenda opatsirana pogonana ndi Tizirombo toyambitsa matenda a Edzi.

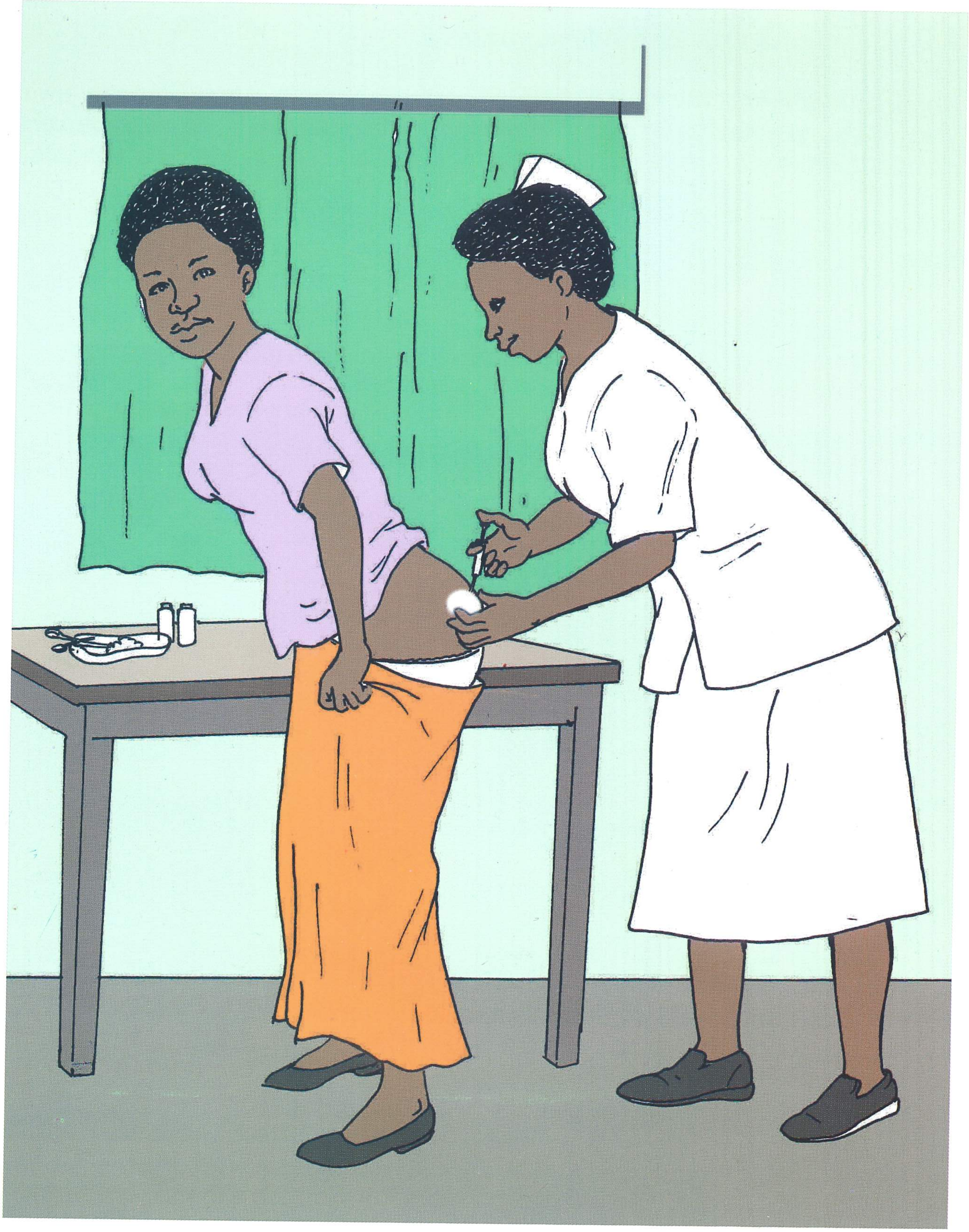

Kulera

KONDOMU YA AMAYI

Apa tikuona amai akuvala kondomu ya amayi

Kondomu ya amayi ndi mphira imene ili ndi mikombero kumbali zonse ziwiri yomwe amayi amavala ku nyini pofuna kudziteteza ku mimba yosakonzekera komanso matenda opatsirana pogonana, kuphatikizapo tizilombo toyambitsa EDZI

Kondomuyi ndi yotsekedwa mbali imodzi. Mbali yotsekedwa imakhala mkati mwa nyini ndipo imatseka khomo la chiberekero. Mbali yotsekula imakhala kunja kwa milomo ya bumbu mkazi akaivala.

Momwe imagwirira ntchito

Imaletsa mbewu ya amuna kulowa muchibelekero ndipo akazi sangatenge mimba.

Ubwino wake

- Abambo ndi amai amagonana mosadera nkhawa kuti mkazi atenga mimba.
- Kondomu ndi njira yokhayo imene ingateteze amai kuti asatenge mimba yosakonzekera, komanso kumatenda, opatsirana pogonana kuphatikiza tizirombo toyambitsa EDZI
- Kondomu ya amayi ndi yolimba, yosavuta kugwiritsa ntchito komanso yodalilika
- Siisokoneza kugonana popeza mayi akhoza kuvaliratu

Kuvuta kwake

- Ena amamva kuyabwa.

Malangizo

- Abambo ndi amai agwiritse ntchito kondomu nthawi zonse asanagonane.
- Gwirani mopinda mkombero wambali yotsekayo ndipo tsekulani nyini ndi dzanja linalo.
- Lowetsani kondomu mu nyini mpakana mumve kuti yalowa mokwanira
- Polowetsa mukhoza kunyonyomala, Kuyima mwendo wina mutaika pa mpando, mutakhala kumapeto kwa mpando miyendo mutakhanyula kapena kugona chagada mutakhanyula miyendo.
- Muonetsetse kuti mkombero wa kunja waphimba milomo ya bumbu lanu.
- Amai athandizire mwamuna polowetsa mbolo mkati mwa kondomu.
- Mukatha kugonana potolozani mkombero wa kunja ndi kutulutsa. Ikulungeni mpepala ndi kuitaya mchimbudzi chokumba kapena kuitentha.
- Gwiritsani ntchito kondomu ina mukafuna kugonananso

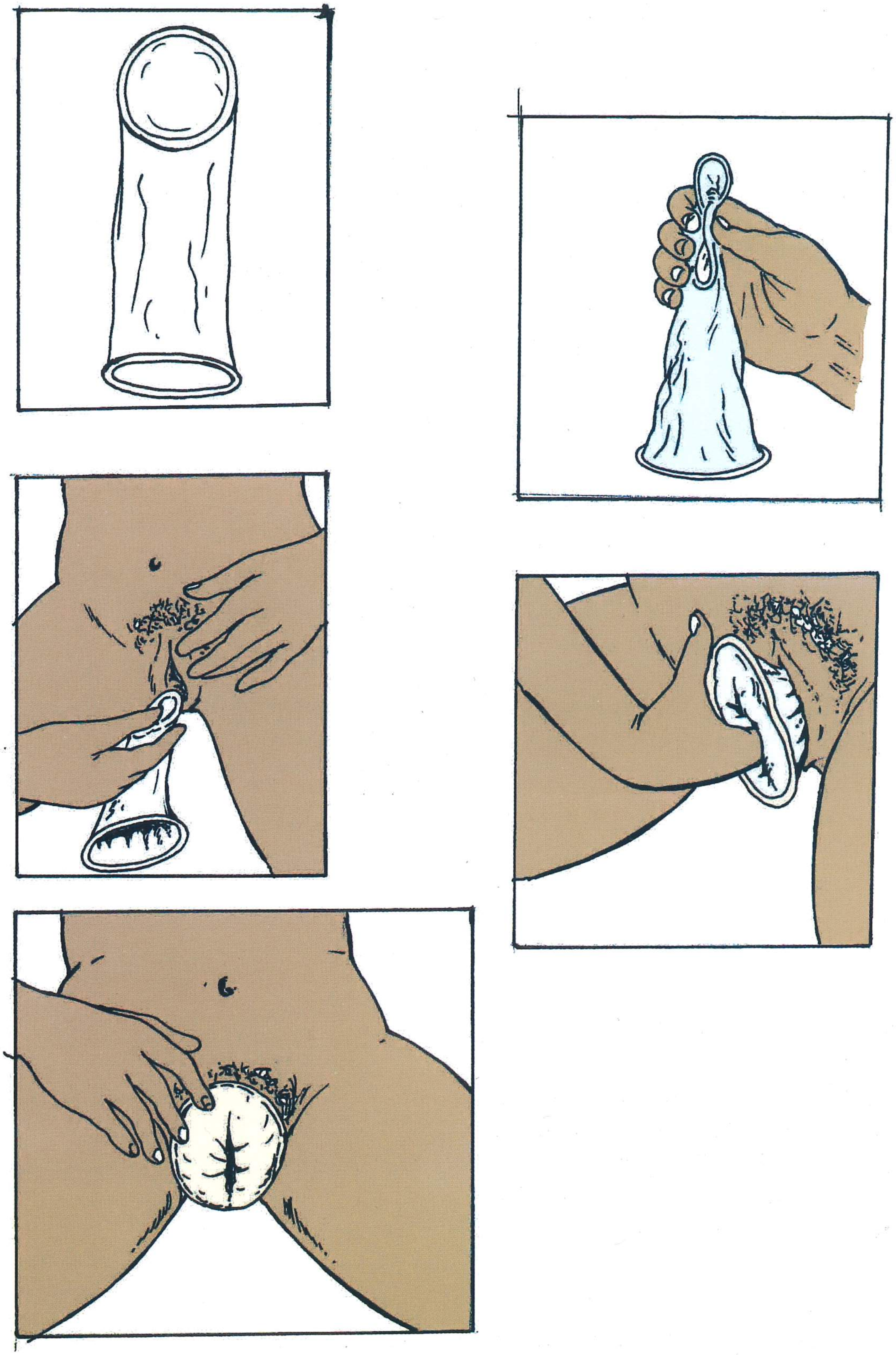

Kulera

KONDOMU YAABAMBO

Apa tikuona mwamuna akuvala kondomu

Kondomu ndi kathumba kamphira komwe amuna amaveka mbolo ikatota, asanayambe kugonana ndi mkazi

Momwe imagwirira ntchito

Imaletsa mbewu ya abambo kulowa muchibelekero ndipo amayi sangatenge mimba.

Ubwino wake

- Abambo ndi amai amagonana mosadera nkhawa kuti mayi atenga mimba.
- Kondomu ndi njira yokhayo immene imateteze mwamuna ndi mkazi kumatenda opatsirana pogonana kuphatikizapo
- tizirombo toyambitsa EDZI komanso imateteza mimba yosayembekezera
- Imapangitsa kuti amuna asathire umuna mwansanga.

Kuvuta kwake

- Ena amamva kuyabwa

Malangizo

- Abambo ndi amai agwiritse ntchito kondomu nthawi zonse akamagonana.
- Kondomu ivalidwe mbolo Itatota.
- Gwirani nsonga ya kondomu povala kuti muchotse mpweya.
- Akatha kugonana mwamuna asadikire kuti mbolo ifote ali mu nyini.
- Pochotsa agwire kukamwa kwa kondomu kupewa kuti mbewu yawo ingakhutukire mu nyini.
- Ayimange mfundo ndikuitaya muchimbudzi chokumba kapena kuitentha.
- Agwiritse ntchito kondomu ina akafuna kugonananso.

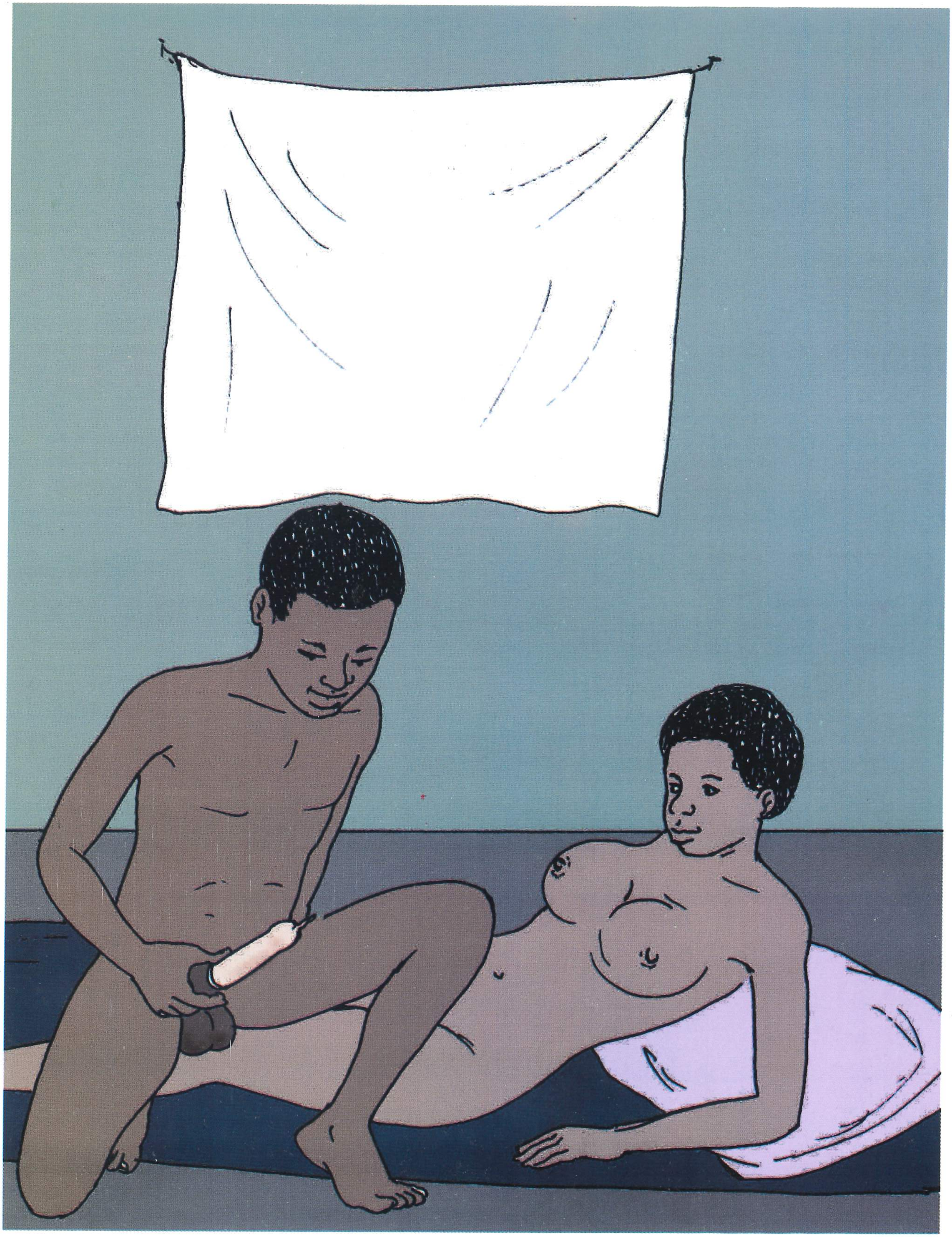

Kulera

NJIRA YOYAMWITSA

Apa tikuona amai amene akugwiritsa ntchito njira yoyamwitsa.
Iyi ndi njira yolerela imene imagwira ntchito pamene mayi akuyamwitsa mwana mwakathithi. Mwanayo akhale ochepera miyezi isanu ndi umodzi ndipo mayiyo akhale asanayambe kumusambira.

Momwe imagwirira ntchito

Mwana akamayamwa mwakathithi dzira lamayi silimakhwima ndipo mayi samasamba choncho sangatenge mimba.

Ubwino wake

- Mayi akamayamwitsa mwakathithi mwana amatetezedwa ku matenda osiyanasiyana monga chimfine, kutsegula m'mimba ndi kukhosomola.
- Njirayi simabweretsa vuto lili lonse m'nthupi mwamayi.

Kuvuta kwake

Njirayi siyodalilika kwenikweni chifukwa amayi ena amatha kutenga mimba akuyamwitsa ngakhale asanasambe.

Malangizo

- Mayi adziyamwitsa mwana mwakathithi kuti njirayi igwire ntchito ndi kumuteteza kutenga mimba.

Chidziwitso

Kuyamwitsa mwakathithi kumatanthauza kuti mwana ayamwe mkaka wa m'mawere okhaokha osampatsa chakudya ndi chakumwa chilichonse ngakhale madzi

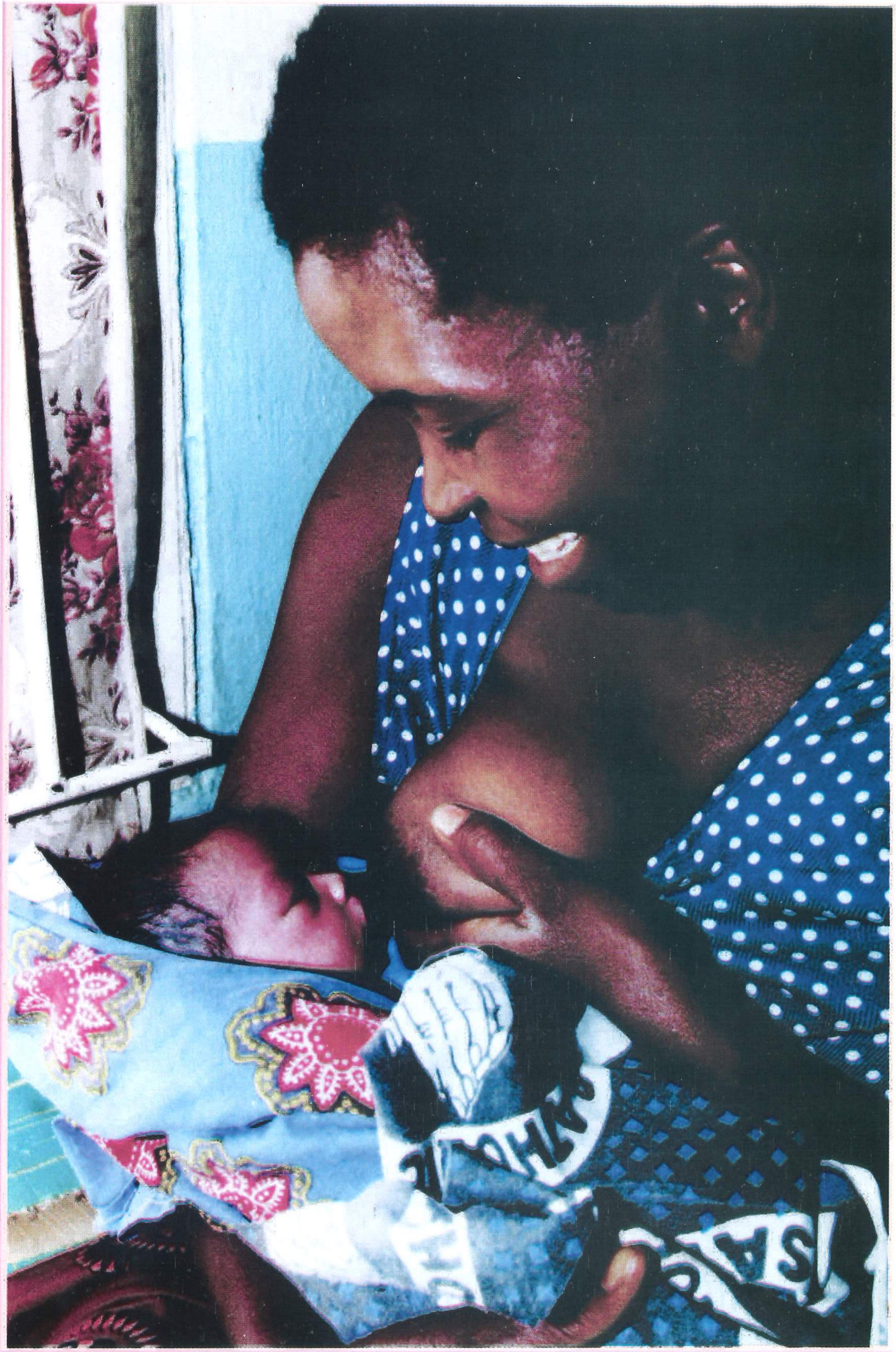

Kulera

NJIRA ZACHILENGEDWE

Njira yachilengedwe ndi njira yomwe mayi amagwiritsa ntchito potsata zomwe zikuchitika mthupi mwake.

NJIRA YOONA M'MENE CHIKAZI CHILILI

Momwe imagwirira ntchito

Nthawi yomwe basiketi lamazira latulutsa dzira lokhwima, chikazi chimakhala chotamuka, chotelera ndiponso choyera ngati dzira losaphika. Nthawi imeneyi mwamuna ndi mkazi asagonane chifukwa mkaziyo angathe kutenga mimba.

Ubwino wake

- Njirayi simabweretsa vuto lililonse mthupi la mayi.
- Akazi amene sakufuna kugwiritsa ntchito njira zina zakulera akhoza kugwiritsa ntchito njirayi.

Kuvuta kwake

- Njirayi ndiyosadalilika kwenikweni
- Mwamuna ndi mkazi amalephela kugonana chilalolako ali nacho.
- Akazi ena sangathe kudziwa bwino mmene chikazi chawo chilili

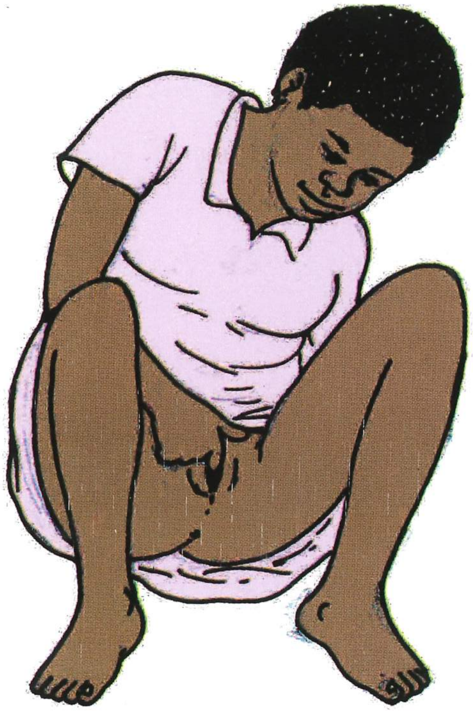

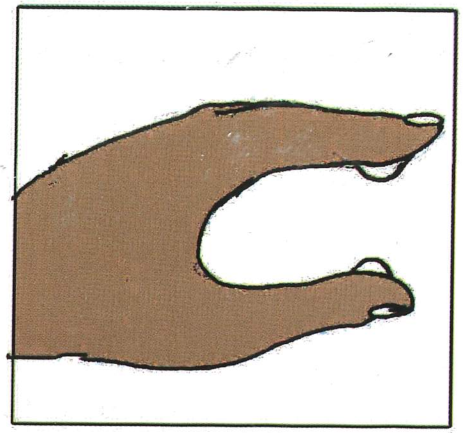

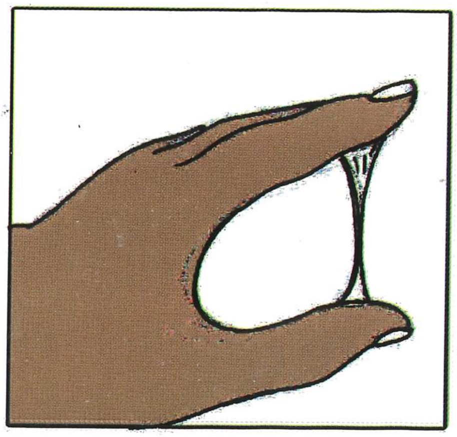

Kulera

NJIRA YOWERENGA MASIKU

Momwe njirayi imagwirira ntchito

Pali masiku omwe amai sangatenge mimba ngakhale atagonana ndi abambo popanda chitetezo china chilli chonse.

Njirayi imagwira ntchito ngati amai ndi abambo sakugonana masiku amene mai dzira lake lakhwima.

Kawerengedwe ka masiku okhwima dzira

- Amai ayenera kuti adziwe bwinobwino m'mene msambo wake umayendera powerenga masiku amene amapyola pakati pa misambo iwiri chifukwa dzira limakhwima pakatikati pa misambo iwiri.
- Amai angadziwe pakatikati pa misambo iwiri pogwiritsa ntchito kalendala motere:
- Amai azichonga pa kalendala tsiku loyamba la msambo wake
- Achite izi kokwana miyezi isanu ndi umodzi
- Asankhe mwezi omwe unali ndi masiku ambiri ndipo achotselepo masiku khumi ndi limodzi(11)
- Asankhe mwezi omwe unali ndi masiku ochepa ndipo achotselepo masiku khumi ndi mphambu zisanu ndi zitatu (18)

Chitsanzo

Mwezi wautali masiku 30 kuchotsela 30-11 = 19

Mwezi waufupi masiku 26 kuchotsela 26 -18 = 8

Izi zitanthauza kuti deti ya 8 pa kalendala ya mwezi ulionse mpaka deti 19 mwezi ulionse ndi nthawi yomwe amai atha kutenga mimba.

Ubwino wake

Njirayi simabweletsa vuto lililonse mthupi la amai.

Amai amene sakufuna kugwiritsa ntchito njira zina zolerera akhoza kugwiritsa ntchito njirayi.

Kuvuta kwake

- Njirayi ndi yosadalilika kwenikweni
- Abambo ndi amai amalephera kugonana chilakolako ali nacho
- Amai ena amalephela kudziwa kachongedwe ndi kuchotsela kwake kwa masiku
- Siyiteteza kumatenda opatsirana pogonana kuphatikizirapo EDZI.

Sun	Mon	Tue	Wed	Thu	Fri	Sat
1	2	3	4	5	6	7
8	9	10	11	12	13	14
15	16	17	18	19	20	21
22	23	24	25	26	27	28
29	30	31				

Sun	Mon	Tue	Wed	Thu	Fri	Sat
			1	2	3	4
5	6	7	8	9	10	11
12	13	14	15	16	17	18
19	20	21	22	23	24	25
26	27	28				

Sun	Mon	Tue	Wed	Thu	Fri	Sat
			1	2	3	4
5	6	7	8	9	10	11
12	13	14	15	16	17	18
19	20	21	22	23	24	25
26	27	28	29	30	31	

Kulera

NJIRA YOGWIRITSA NTCHITO MIKANDA

Iyi ndi njira yatsopano yachirengedwe imene mkazi amaphunzitsidwa kuti asagonane ndi mwamuna kuyambira tsiku la eyiti akangoyamba nsambo mpaka tsiku la nayintini (8-19) kuti apewe kutenga mimba, ndipo imagwira ntchito kwa akazi amene msambo wawo umatenga masiku 26 mpaka 32. Potsatira njirayi, akazi amagwiritsa ntchito nekilesi ya mikanda imene imakhala ndi mitundu yosiyana-siyana kudziwitsa masiku amene mkazi akhoza kutenga mimba kapena ayi.

Momwe imagwirira ntchito

Pali masiku omwe mkazi sangatenge mimba ngakhale atagonana ndi mwamuna popanda chitetezo china chili chonse.

Njirayi imagwira ntchito posagonana masiku amene mkazi dzira lake lakhwima.

Ubwino Wake

- Njirayi simabweletsa vuto lililonse mthupi mwa amai
- Amai amene sakufuna kugwiritsa ntchito njira zina zolerera akhoza kugwiritsa ntchito njirayi.
- Ikhoza kugwiritsidwa ntchito pofuna kupewa mimba ngakhalenso kutenga mimba.
- Siyokwera mtengo
- Abambo amatenga nawo mbali pa nkhani ya kulera

Kuvuta kwake

- Njirayi ndi yosadalilika kwenikweniAbambo ndi amai amalephera kugonana chilakolako ali nacho
- Siyiteteza kumatenda opatsirana kuphatikizirapo tizilombo toyambitsa matenda a EDZI.Amai amene msambo wawo sukwanira masiku 26 kapena kupyola masiku 32 sayenera kugwiritsa ntchito njirayi.

Malangizo

- Amai amene sakufuna kutenga mimba asagonane pamene mulozera ali pa mikanda yoyera.
- Amai sangatenge mimba pamene mulozera ali pa mikanda ya (brown) ngati masiku amsambo kuchokera mwezi wina kufikira mwezi wina akuchepera masiku 26 kapena akupyolerera masiku 32 pamiyezi iwiri
- Ayenera asagwiritsenso njirayi ndipo asankhe yina.

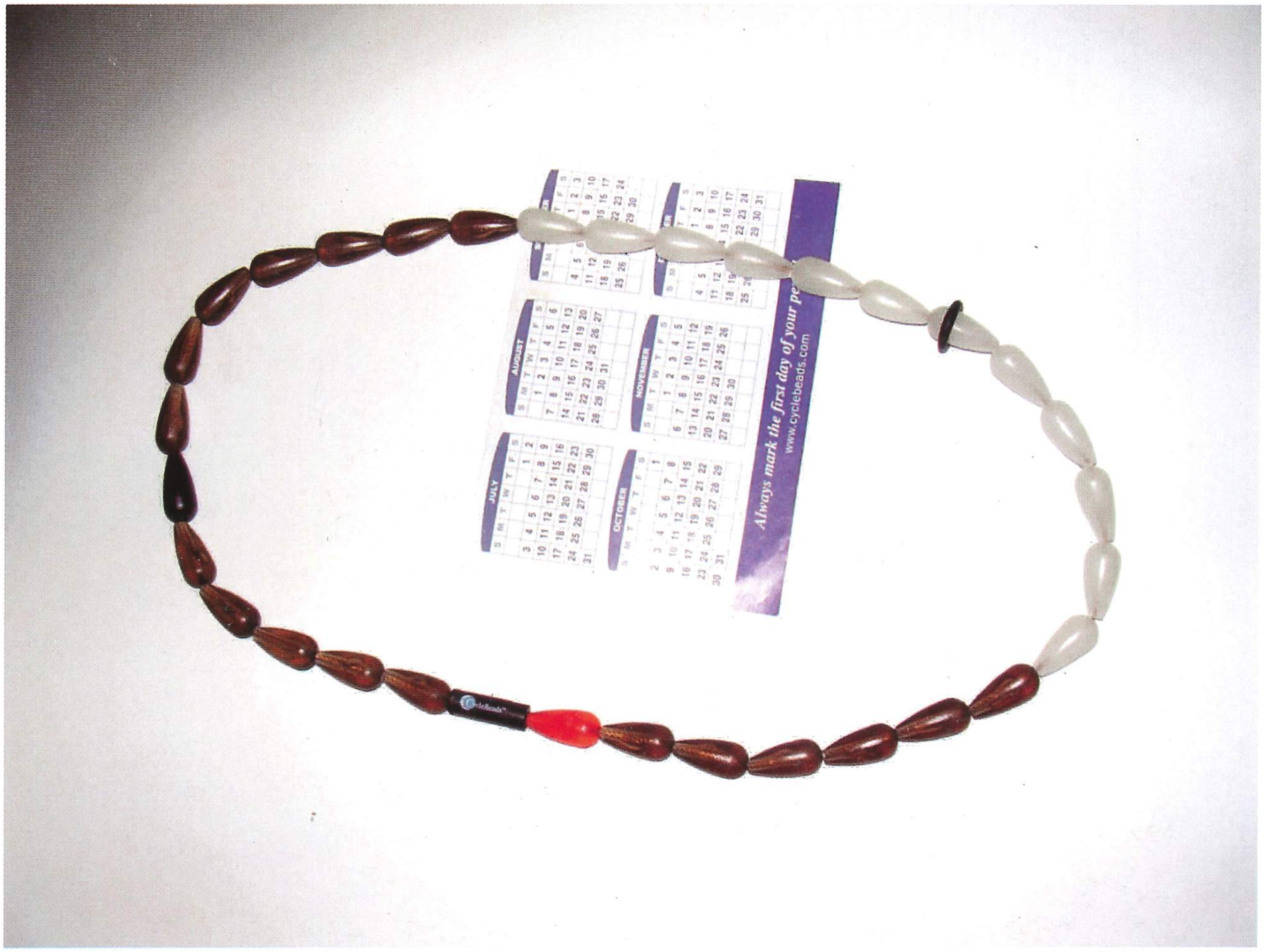
JULY
AUGUST
OCTOBER
NOVEMBER
Always mark the first day of your pe
www.cyclebeads.com

Kulera

Apa tikuona a Phiri ndi akazi awo omwe akhala akugwritsa ntchito njira zina zolelera. Tsopano abereka ana okwana anayi ndipo agwirizana zakuti aleke kubereka chifukwa azindikira kuopsa kobereka ana oposa anayi. Asankha njira yotseka ndipo agwirizana kuti Aphiri ndiwo akatseketse.

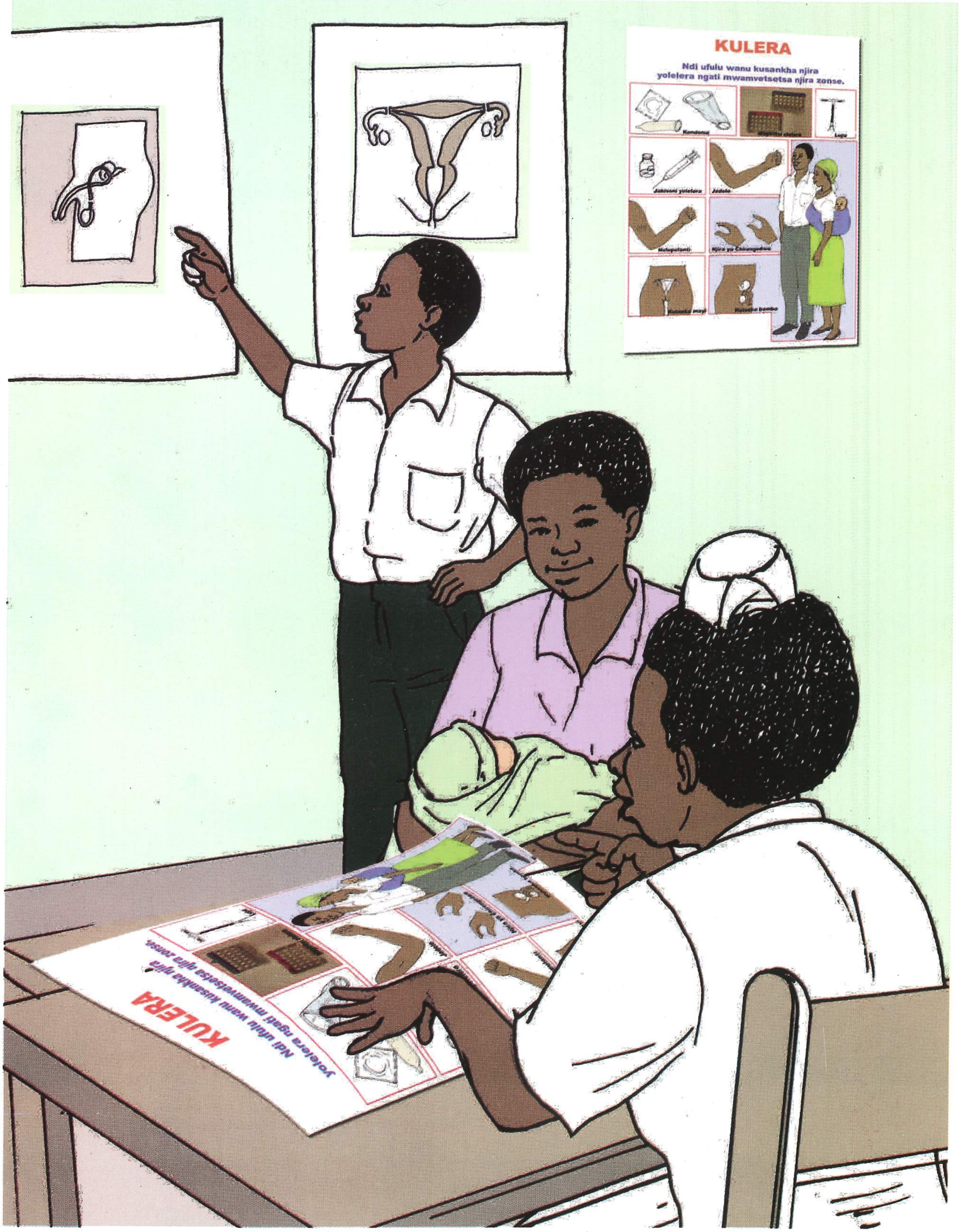
KULERA
Ndi ufulu wanu kusankha njira yolelera ngati mwamvetsetsa njira zonse.
